# Use of repurposed and adjuvant drugs in hospital patients with covid-19: multinational network cohort study

**DOI:** 10.1136/bmj.n1038

**Published:** 2021-05-11

**Authors:** Albert Prats-Uribe, Anthony G Sena, Lana Yin Hui Lai, Waheed-Ul-Rahman Ahmed, Heba Alghoul, Osaid Alser, Thamir M Alshammari, Carlos Areia, William Carter, Paula Casajust, Dalia Dawoud, Asieh Golozar, Jitendra Jonnagaddala, Paras P Mehta, Mengchun Gong, Daniel R Morales, Fredrik Nyberg, Jose D Posada, Martina Recalde, Elena Roel, Karishma Shah, Nigam H Shah, Lisa M Schilling, Vignesh Subbian, David Vizcaya, Lin Zhang, Ying Zhang, Hong Zhu, Li Liu, Jaehyeong Cho, Kristine E Lynch, Michael E Matheny, Seng Chan You, Peter R Rijnbeek, George Hripcsak, Jennifer CE Lane, Edward Burn, Christian Reich, Marc A Suchard, Talita Duarte-Salles, Kristin Kostka, Patrick B Ryan, Daniel Prieto-Alhambra

**Affiliations:** 1 Pharmaco- and Device Epidemiology, Centre for Statistics in Medicine, Nuffield Department of Orthopaedics, Rheumatology, and Musculoskeletal Sciences, University of Oxford, Oxford, UK; 2 Janssen Research and Development, Titusville, NJ, USA; 3 Department of Medical Informatics, Erasmus University Medical Center, Rotterdam, Netherlands; 4 Division of Cancer Sciences, School of Medical Sciences, University of Manchester, Manchester, UK; 5 Nuffield Department of Orthopaedics, Rheumatology, and Musculoskeletal Sciences, University of Oxford, Oxford, UK; 6 College of Medicine and Health, University of Exeter, Exeter, UK; 7 Faculty of Medicine, Islamic University of Gaza, Gaza City, Palestine; 8 Massachusetts General Hospital, Harvard Medical School, Boston, MA, USA; 9 College of Pharmacy, Riyadh Elm University, Riyadh, Saudi Arabia; 10 Critical Care Research Group, Nuffield Department of Clinical Neurosciences, University of Oxford, Oxford, UK; 11 University of Colorado Anschutz Medical Campus, Aurora, CO, USA; 12 Real-World Evidence, Trial Form Support, Barcelona, Spain; 13 Faculty of Pharmacy, Cairo University, Cairo, Egypt; 14 National Institute for Health and Care Excellence, London, UK; 15 Regeneron Pharmaceuticals, Tarrytown, NY, US; 16 Department of Epidemiology, Johns Hopkins Bloomberg School of Public Health, Baltimore, MD, USA; 17 School of Population Health, UNSW Sydney, Sydney, Australia; 18 College of Medicine, University of Arizona, Tucson, AZ, USA; 19 DHC Technologies, Beijing, China; 20 Division of Population Health and Genomics, University of Dundee, Dundee, UK; 21 Department of Public Health, University of Southern Denmark, Odense, Denmark; 22 School of Public Health and Community Medicine, Institute of Medicine, Sahlgrenska Academy, University of Gothenburg, Gothenburg, Sweden; 23 Department of Medicine, Stanford University School of Medicine, Stanford, CA, USA; 24 Fundació Institut Universitari per a la recerca a l’Atenció Primària de Salut Jordi Gol i Gurina (IDIAPJGol), Barcelona, Spain; 25 Universitat Autònoma de Barcelona, Barcelona, Spain; 26 College of Engineering, University of Arizona Tucson, AZ, USA; 27 Bayer Pharmaceuticals, Sant Joan Despí, Spain; 28 School of Population Medicine and Public Health, Chinese Academy of Medical Sciences and Peking Union Medical College, Beijing, China; 29 School of Population and Global Health, University of Melbourne, Melbourne, VIC, Australia; 30 Nanfang Hospital, Southern Medical University, Guangzhou, China; 31 Department of Biomedical Informatics, Ajou University School of Medicine, Suwon, South Korea; 32 VA Informatics and Computing Infrastructure, VA Salt Lake City Healthcare System, Salt Lake City, Utah, USA; Department of Internal Medicine, University of Utah School of Medicine, Salt Lake City, UT, USA; 33 VA Informatics and Computing Infrastructure, Tennessee Valley Healthcare System, VA Medical Center, Nashville, TN, USA; 34 Department of Biomedical Informatics, Vanderbilt University Medical Center, Nashville, TN, USA; 35 Department of Preventive Medicine and Public Health, Yonsei University College of Medicine, Seoul, South Korea; 36 Department of Biomedical Informatics, Columbia University, New York, NY, USA; 37 IQVIA, Cambridge, MA, USA; 38 Department of Biostatistics, UCLA Fielding School of Public Health, University of California, Los Angeles, Los Angeles, CA, USA; 39 OHDSI Center at The Roux Institute, Northeastern University, Portland, ME, USA; 40 Columbia University Irving Medical Center, New York, NY, USA

## Abstract

**Objective:**

To investigate the use of repurposed and adjuvant drugs in patients admitted to hospital with covid-19 across three continents.

**Design:**

Multinational network cohort study.

**Setting:**

Hospital electronic health records from the United States, Spain, and China, and nationwide claims data from South Korea.

**Participants:**

303 264 patients admitted to hospital with covid-19 from January 2020 to December 2020.

**Main outcome measures:**

Prescriptions or dispensations of any drug on or 30 days after the date of hospital admission for covid-19.

**Results:**

Of the 303 264 patients included, 290 131 were from the US, 7599 from South Korea, 5230 from Spain, and 304 from China. 3455 drugs were identified. Common repurposed drugs were hydroxychloroquine (used in from <5 (<2%) patients in China to 2165 (85.1%) in Spain), azithromycin (from 15 (4.9%) in China to 1473 (57.9%) in Spain), combined lopinavir and ritonavir (from 156 (<2%) in the VA-OMOP US to 2,652 (34.9%) in South Korea and 1285 (50.5%) in Spain), and umifenovir (0% in the US, South Korea, and Spain and 238 (78.3%) in China). Use of adjunctive drugs varied greatly, with the five most used treatments being enoxaparin, fluoroquinolones, ceftriaxone, vitamin D, and corticosteroids. Hydroxychloroquine use increased rapidly from March to April 2020 but declined steeply in May to June and remained low for the rest of the year. The use of dexamethasone and corticosteroids increased steadily during 2020.

**Conclusions:**

Multiple drugs were used in the first few months of the covid-19 pandemic, with substantial geographical and temporal variation. Hydroxychloroquine, azithromycin, lopinavir-ritonavir, and umifenovir (in China only) were the most prescribed repurposed drugs. Antithrombotics, antibiotics, H2 receptor antagonists, and corticosteroids were often used as adjunctive treatments. Research is needed on the comparative risk and benefit of these treatments in the management of covid-19.

## Introduction

By the end of 2020, more than 85 million confirmed cases of covid-19 and almost 2 000 000 related deaths occurred worldwide.^1^ Despite a lack of evidence on effectiveness, several medicines were repurposed in the first few months of the pandemic on the basis of in vitro antiviral activity.^2^

For the purpose of illustration, the US Food and Drug Administration gave emergency approval for use of hydroxychloroquine on 28 March 2020 but revoked this on 15 June 2020^3^ and the Recovery and Solidarity trials also found little benefit associated with hydroxychloroquine use.^4^ ^5^ Remdesivir was also proposed as treatment for covid-19 after showing in vitro antiviral activity against SARS-CoV-2.^2^ An international placebo controlled randomised controlled trial showed a decrease in time to recovery.^6^ The Solidarity trial, however, suggested that remdesivir has no benefit on mortality, need for mechanical ventilation, and duration of hospital stay.^5^ Other drugs, such as interferon and lopinavir combined with ritonavir have also been shown to be ineffective.^5^ ^7^

In the absence of approved antivirals for the treatment of covid-19, the cornerstone of management has been supportive care, with adjunctive treatments playing a major role. The two recognised drug classes used for adjunctive treatment are corticosteroids and anticytokines (eg, tocilizumab). A large randomised controlled trial and meta-analysis showed that the glucocorticosteroid dexamethasone and corticosteroids reduced mortality among patients receiving mechanical ventilation or oxygen.^8^ ^9^ Tocilizumab was found to significantly reduce mortality in patients admitted to hospital with covid-19.^10^ Although additional adjunctive treatments are recognised in 2020 guidelines, including antithrombotics, statins, and antihypertensives,^11^ ^12^ ^13^ ^14^ ^15^ recommendations for covid-19 treatment in clinical guidelines have varied both geographically and temporally.^16^

Regulators and public health agencies need to keep up with trends in covid-19 clinical practice. Tweets and press conferences have been shown to influence entire practice patterns but based on little evidence for the utility of treatments. Although attention has shifted to vaccine surveillance since December 2020, there is still a need to understand what treatments are effective for individual patients and at what harm This body of evidence is critical for comparative purposes as more data become available during the pandemic. With known problems in the supply chain for certain drugs, an understanding of what drugs are being used to treat covid-19 at different stages of the disease could help resource constrained environments.

We investigated the use of repurposed and adjunctive drugs among patients admitted to hospital with covid-19 and among patients receiving intensive care in the United States, South Korea, Spain, and China.

## Methods

This multinational network cohort study was based on hospital electronic health records and claims data. We mapped data from different sites to the Observational Medical Outcomes Partnership (OMOP) Common Data Model (CDM).^17^ This approach allowed contributing centres to execute analytical code in a distributed or federated fashion, where each site runs the analyses separately in-house and returns a results dataset without sharing patient level data. The study protocol and analytical package were released on 11 June 2020, and iterative updates are continually released through GitHub.^18^ Our study was also published as a preprint.^19^

### Data sources

Data were obtained from the US, South Korea, Spain, and China. Electronic health record data from the US were obtained from Columbia University Irving Medical Center (CUIMC, February to December 2020), IQVIA Hospital CDM (February to October 2020), STAnford medicine Research data Repository (STARR-OMOP database^20^ from February to May 2020, and Premier database from February to August 2020), Optum (Eden Prairie, MN) deidentified electronic health record dataset (Optum-EHR, February to October 2020), Tufts Medical Center Clinical Academic Research Enterprise Trust (Tufts Research Data Warehouse (TRDW), February to May 2020), and the Department of Veterans Affairs (VA-OMOP, February to June 2020). Data for South Korea came from nationwide claims recorded in the Health Insurance Review and Assessment (HIRA, February to April 2020).^21^ Inpatient electronic health record data from Spain was obtained from HM Hospitales (March to April 2020) and Hospital del Mar (February to August 2020). Data from China was extracted from nine hospitals in Honghu, supported by Nanfang Hospital and Southern Medical University, and contained full electronic health record data (NFHCRD database, January to April 2020). Data on drug use in patients receiving intensive care were available from IQVIA Hospital CDM, Premier, Optum-EHR, VA-OMOP, HM Hospitales, and Hospital del Mar. Supplementary table 1 provides a detailed description of the databases.

### Study participants

Patients admitted to hospital with a recorded diagnosis of covid-19 or a positive polymerase chain reaction test result for SARS-CoV-2 between January and December 2020 were included. A second cohort of patients who received intensive care was identified as a subset of the former, defined by the initiation of mechanical ventilation, extracorporeal membrane oxygenation, or tracheostomy. Index dates for the two cohorts were the date of admission to hospital and the date intensive care started, respectively.

### Drugs of interest

We obtained information on all drugs prescribed or dispensed during hospital admission. For the study of treatments used for covid-19, we assessed all drugs included in at least two randomised controlled trials according to the covid-19 clinical trial tracker.^22^ The resulting list was circulated to stakeholders with a role in drug development and research (eg, key opinion leaders, pharmaceutical industry) and drug regulatory agencies. All their suggestions were added to the final list of medicines under study. We classified the drugs into two groups: repurposed drugs—those with alternative indications but thought to be efficacious as antivirals; and adjuvant drugs—those used to treat pneumonia or prevent or treat complications from covid-19.^23^ Supplementary table 3 lists the drugs considered. For the main results, we focused on drugs covered in the living World Health Organization guideline for drugs—hydroxychloroquine, lopinavir combined with ritonavir, remdesivir, and dexamethasone.^7^

### Statistical analysis

We summarise age, sex, and history of medical conditions as proportions (the number of participants within a category, divided by the total number of participants). Supplementary table 2 shows the clinical codes and time windows used to identify medical conditions.

**Fig 1:**
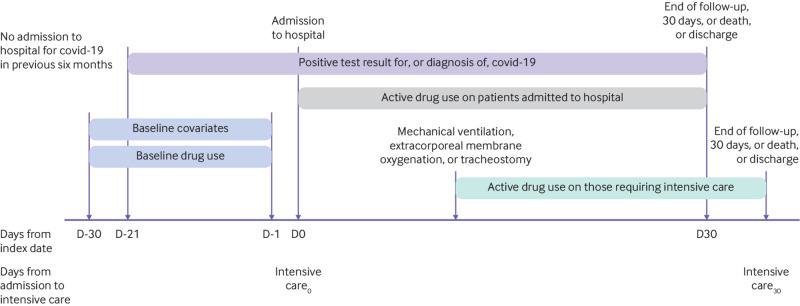
Timeline of study

Drug use was calculated from the index date (admission date or initiation of intensive care) to 30 days after, or discharge, or death, when these dates were available in the database. We calculated use for each drug and major drug class. Prevalence of drug use was determined by the proportion of participants with any active prescription or dispensation of a certain drug or drug during hospital admission or the period of intensive care. Figure 1 provides a timeline of the study. Supplementary figure 1 shows which drugs could potentially have been prescribed in the month before hospital admission.

All drugs and additional time windows (previous year, previous month, and on index date) are reported in full and will be updated in a dedicated interactive website (https://data.ohdsi.org/Covid19CharacterizationCharybdis/) as more data become available. All (aggregated) data can be downloaded from this website.

To better visualise drug use, we generated rainbow plots for each database. These plots display the proportion of users of each drug using Anatomical Therapeutic Chemical groupings. We also created lollipop plots of drug use to show the heterogeneity for all selected repurposed and adjuvant drugs (see supplementary file). On the basis of drug use proportions, we determined the top five most used repurposed drugs and top 10 most used adjuvant drugs for each database and setting; use of the focused medicines are depicted by gauge plots.

We calculated use of the selected drugs by month of index date (admission to hospital or start of intensive care). To ensure enough time points, we selected databases with two or more months of data available for each drug. Drug use was plotted for each calendar month in the study period. A timeline of selected relevant events, such as regulatory decisions or trial results for the selected medicines, was added. The supplementary appendix provides time series graphs for all drugs and groupings.

### Patient and public involvement

No funding was available for patient or public involvement in this project. Urgency because of the covid-19 pandemic and restrictions also prevented us from actively involving patients, although the Observational Health Data Sciences and Informatics community welcomes members of the public to engage with its work. No patients were involved in setting the research question or the outcome measures. Patients were not invited to comment on the study design, not consulted to develop patient relevant outcomes or interpret the results, and not invited to contribute to the writing or editing of this document for readability or accuracy.

## Results

A total of 303 264 patients identified from 11 databases were included: 290 131 participants were from the US (744 from California, 326 from Massachusetts, 7353 from New York, 10 951 from US-wide Veterans Affairs, and 270 757 from US-wide databases: Premier, IQVIA Hospital CDM, and Optum-EHR), 7599 from South Korea, 5230 from Spain, and 304 from China, Of these 303 264 participants, 62 963 (from VA-OMOP, Premier, Optum-EHR, IQVIA Hospital CDM, Hospital del Mar, and HM Hospitales) received intensive care.

The results of this study are available in an interactive website (https://data.ohdsi.org/Covid19CharacterizationCharybdis/). This website contains both the summary results presented here and further details, including all drugs and comorbidities recorded for the two cohorts.

Table 1 presents the baseline characteristics of the patients admitted to hospital with covid-19. Supplementary table 4 shows the results for patients who received intensive care. Age varied slightly across data sources, but most patients were within the age range 50 to 74 years. The proportion of women was 40-50% in all settings except South Korea (59%) and the VA-OMOP (7%).

**Table 1:** Baseline characteristics of patients admitted to hospital with covid-19, stratified by data source. Data are numbers (percentages)

Characteristics	USA: CUIMC (n=7353)	South Korea: HIRA (n=7599)	Spain: HM Hospitales (Spain; n=2544)	Spain: Hospital del Mar (n=2686)	USA: IQVIA Hospital CDM (n=77 853)	China: NFHCRD (n=304)	USA: Optum-EHR (n=36 717)	USA: Premier (n=156 187)	USA: STARR-OMOP (n=744)	USA: TRDW (n=326)	USA: VA-OMOP (n=10 951)
Sex:											
Female	3897 (53)	4483 (59)	1043 (41)	1424 (53)	38 139 (49)	149 (49)	18 359 (50)	74 970 (48)	372 (50)	140 (43)	767 (7)
Age (years):											
0-4	221 (3)	76 (1)	25 (1)	13 (0.5)	2335 (3)	<5	367 (1)	1562 (1)	37 (5)	10 (3)	<5
5-9	74 (1)	76 (1)	<5	<5	<5	<5	367 (1)	<5	7 (1)	<5	<5
10-14	74 (1)	76 (1)	<5	<5	<5	<5	367 (1)	<5	<5	<5	<5
15-19	147 (2)	228 (3)	<5	11 (0.4)	778 (1)	<5	734 (2)	1562 (1)	15 (2)	<5	<5
20-24	221 (3)	988 (13)	76 (3)	91 (3)	1557 (2)	6 (2)	1102 (3)	3124 (2)	15 (2)	<5	<5
25-29	368 (5)	912 (12)	178 (7)	199 (7)	2335 (3)	12 (4)	1469 (4)	4686 (3)	30 (4)	16 (5)	110 (1)
30-34	441 (6)	380 (5)	153 (6)	167 (6)	3113 (4)	24 (8)	1836 (5)	6247 (4)	37 (5)	16 (5)	110 (1)
35-39	441 (6)	380 (5)	127 (5)	145 (5)	3113 (4)	24 (8)	1836 (5)	6247 (4)	37 (5)	20 (6)	219 (2)
40-44	368 (5)	380 (5)	204 (8)	210 (8)	3892 (5)	24 (8)	1836 (5)	7809 (5)	45 (6)	10 (3)	219 (2)
45-49	294 (4)	608 (8)	204 (8)	212 (8)	4670 (6)	36 (12)	2203 (6)	9371 (6)	45 (6)	20 (6)	329 (3)
50-54	441 (6)	760 (10)	229 (9)	242 (9)	6227 (8)	40 (13)	2937 (8)	12 495 (8)	52 (7)	13 (4)	657 (6)
55-59	588 (8)	760 (10)	229 (9)	239 (9)	7784 (10)	46 (15)	3672 (10)	14 057 (9)	82 (11)	36 (11)	876 (8)
60-64	662 (9)	684 (9)	204 (8)	215 (8)	8562 (11)	27 (9)	3672 (10)	15 619 (10)	82 (11)	39 (12)	1314 (12)
65-69	662 (9)	380 (5)	153 (6)	148 (6)	8562 (11)	18 (6)	3672 (10)	15 619 (10)	82 (11)	42 (13)	1424 (13)
70-74	662 (9)	304 (4)	153 (6)	150 (6)	8562 (11)	15 (5)	3305 (9)	15 619 (10)	89 (12)	20 (6)	2300 (21)
75-79	588 (8)	304 (4)	178 (7)	196 (7)	7005 (9)	18 (6)	2570 (7)	14 057 (9)	60 (8)	20 (6)	1424 (13)
80-84	515 (7)	152 (2)	127 (5)	140 (5)	9340 (12)	9 (3)	2203 (6)	12 495 (8)	30 (4)	23 (7)	657 (6)
85-89	294 (4)	152 (2)	153 (6)	172 (6)	<5	<5	2937 (8)	14 057 (9)	15 (2)	16 (5)	657 (6)
90-94	221 (3)	76 (1)	102 (4)	105 (4)	<5	<5	<5	4686 (3)	<5	7 (2)	438 (4)
≥95	74 (1)	<5	25 (1)	31 (1)	<5	<5	<5	<5	<5	13 (4)	219 (2)
Comorbidities:											
Anaemia	294 (4)	304 (4)	—	27 (1)	3113 (4)	—	2203 (6)	3124 (2)	74 (10)	13 (4)	876 (8)
Anxiety disorder	74 (1)	304 (4)	—	<5	1557 (2)	—	1469 (4)	1562 (1)	60 (8)	<5	1205 (11)
Asthma	147 (2)	380 (5)	—	<5	1557 (2)	—	1102 (3)	1562 (1)	52 (7)	7 (2)	219 (2)
Atrial fibrillation	147 (2)	76 (1)	—	27 (1)	1557 (2)	—	1469 (4)	1562 (1)	37 (5)	10 (3)	657 (6)
Chronic liver disease	74 (1)	76 (1)	—	<5	778 (1)	—	367 (1)	<5	15 (2)	<5	219 (2)
Chronic obstructive pulmonary disease	74 (1)	76 (1)	—	27 (1)	1557 (2)	—	1102 (3)	1562 (1)	15 (2)	7 (2)	876 (8)
Dementia	<5	304 (4)	—	<5	778 (1)	—	734 (2)	1562 (1)	<5	<5	657 (6)
Diabetes mellitus	368 (5)	532 (7)	—	27 (1)	5448 (7)	—	3672 (10)	4686 (3)	67 (9)	16 (5)	1971 (18)
Gastroesophageal reflux disease	147 (2)	836 (11)	—	<5	1557 (2)	—	1469 (4)	1562 (1)	89 (12)	10 (3)	548 (5)
Heart disease	735 (10)	380 (5)	—	27 (1)	4670 (6)	—	4039 (11)	4686 (3)	141 (19)	23 (7)	2081 (19)
Heart failure	221 (3)	152 (2)	—	<5	2335 (3)	—	1469 (4)	3124 (2)	37 (5)	7 (2)	767 (7)
Hyperlipidaemia	221 (3)	1216 (16)	—	27 (1)	4670 (6)	—	3672 (10)	4686 (3)	164 (22)	16 (5)	1424 (13)
Hypertensive disorder	588 (8)	1292 (17)	—	27 (1)	6227 (8)	—	5140 (14)	4686 (3)	216 (29)	20 (6)	2300 (21)
Insomnia	74 (1)	152 (2)	—	<5	<5	—	367 (1)	<5	7 (1)	<5	329 (3)
Ischaemic heart disease	147 (2)	152 (2)	—	27 (1)	778 (1)	—	1102 (3)	1562 (1)	22 (3)	<5	329 (3)
Low back pain	74 (1)	532 (7)	—	<5	778 (1)	—	734 (2)	<5	22 (3)	<5	548 (5)
Malignant neoplastic disease	515 (7)	152 (2)	—	27 (1)	1557 (2)	—	1469 (4)	1562 (1)	201 (27)	10 (3)	767 (7)
Osteoarthritis of hip	<5	<5	—	<5	<5	—	<5	<5	22 (3)	<5	<5
Osteoarthritis of knee	74 (1)	152 (2)	—	<5	<5	—	367 (1)	<5	37 (5)	<5	219 (2)
Peripheral vascular disease	74 (1)	228 (3)	—	<5	778 (1)	—	734 (2)	1562 (1)	7 (1)	16 (5)	329 (3)
Renal impairment	294 (4)	76 (1)	—	27 (1)	3113 (4)	—	2570 (7)	3124 (2)	89 (12)	<5	1314 (12)
Venous thrombosis	<5	<5	—	<5	<5	—	367 (1)	<5	15 (2)	13 (4)	110 (1)
Viral hepatitis	<5	76 (1)	—	<5	<5	—	<5	<5	15 (2)	<5	110 (1)
Mortality	735 (10)	228 (3)	356 (14)	—	10897 (14)	<5	1469 (4)	1562 (1)	7 (1)	33 (10)	1643 (15)

CUIMC=Columbia University Irving Medical Center; HIRA=Health Insurance Review and Assessment; OMOP=Observational Medical Outcomes Partnership; Optum-EHR=Optum deidentified electronic health record dataset; STARR=STAnford medicine Research data Repository; TRDW=Tufts Research Data Warehouse; VA=Veterans Affairs.

**Fig 2:**
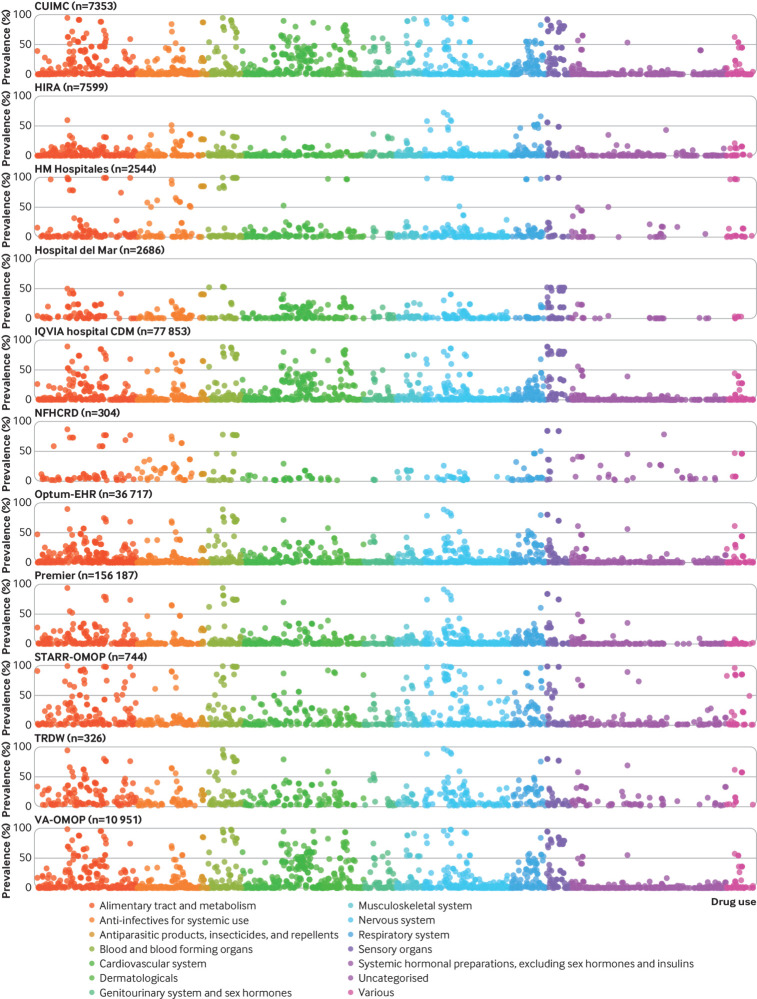
Percentage of any use (one day or more) of all drugs (rainbow plot) on days 0 to 30 after hospital admission in patients with a positive test result for or diagnosis of covid-19. CUIMC=Columbia University Irving Medical Center; HIRA=Health Insurance Review and Assessment; OMOP=Observational Medical Outcomes Partnership; Optum-EHR=Optum deidentified electronic health record dataset; STARR=STAnford medicine Research data Repository; TRDW=Tufts Research Data Warehouse; VA=Veterans Affairs

A total of 3455 different drugs were administered to patients in the month after admission to hospital for covid-19 (fig 2). The Anatomical Therapeutic Chemical groups consistently seen among the most prescribed drugs were anti-infectives for systemic use, treatments for blood and blood forming organs, cardiovascular system therapies, and drugs for the musculoskeletal system.

Table 2 reports the top five most used repurposed drugs and table 3 the top 10 most used adjunctive drugs in each data source among the drugs of interest. Supplementary table 5 shows the results for patients who received intensive care. The most popular antivirals were hydroxychloroquine (from 14% in VA-OMOP, US, to 85% in HM Hospitales, Spain), lopinavir-ritonavir (from 0.3% in VA-OMOP to 50% in HM Hospitales), oseltamivir (0.5% in Optum-EHR, US, to 13% in NFHCRD, China), and remdesivir (7.7% in CUIMC, US, and 7.3% in IQVIA Hospital CDM, US). China used different products: umifenovir, prescribed to 78% of patients admitted to hospital, ribavirin (21%), and chloroquine (12%). Commonly used adjunctive treatments included antithrombotics, corticosteroids, antibiotics, metformin, vitamin supplements (C and D), antihypertensives, H2 receptor antagonists, and interleukin inhibitors.

**Table 2:** Top five most used repurposed drugs in each data source in patients admitted to hospital with covid-19 on days 0 to 30 after hospital admission. Data are treatment (percentage of patients admitted to hospital who received the medicine)

Ranking	USA: CUIMC (n=7353)	South Korea: HIRA (n=7599)	Spain: HM Hospitales (Spain; n=2544)	Spain: Hospital del Mar (n=2686)	USA: IQVIA Hospital CDM (n=77 853)	China: NFHCRD (n=304)	USA: Optum-EHR (n=36 717)	USA: Premier (n=156 187)	USA: STARR-OMOP (n=744)	USA: TRDW (n=326)	USA: VA-OMOP (n=10 951)
1	HCQ (22.3)	L-R (34.9)	HCQ (85.1)	HCQ (40.2)	AZM (47.4)	UMF (78.3)	AZM (37.0)	AZM (46.6)	AZM (8.7)	AZM (28.5)	AZM (33.0)
2	AZM (21.4)	HCQ (27.4)	AZM (57.9)	AZM (7.9)	HCQ (9.3)	RBV (21.1)	HCQ (20.5)	HCQ (22.8)	HCQ (3.2)	HCQ (19.9)	HCQ (13.5)
3	RMD (7.7)	AZM (13.7)	L-R (50.5)	L-R (4.4)	RMD (7.3)	OST (13.2)	L-R (1.2)	OST (0.5)	OST (1.1)	OST (<1.5)	OST (0.7)
4	OST (0.9)	PIFN (0.4)	OST (5.8)	OST (<0.2)	IVM (0.9)	CQ (11.5)	OST (0.5)	L-R (0.4)	ICN (0.7)	L-R (<1.5)	IVM (0.3)
5	IVM (0.3)	OST (0.4)	CQ (0.3)	—	OST (0.5)	L-R (7.2)	IVM (0.2)	—	RBV (<0.7)	—	L-R (0.3)

CUIMC=Columbia University Irving Medical Center; HIRA=Health Insurance Review and Assessment; OMOP=Observational Medical Outcomes Partnership; Optum-EHR=Optum deidentified electronic health record dataset; STARR=STAnford medicine Research data Repository; TRDW=Tufts Research Data Warehouse; VA=Veterans Affairs; HCQ=hydroxychloroquine; AZM=azithromycin; RMD=remdesivir; OST=oseltamivir; IVM=ivermectin; L-R=lopinavir-ritonavir; PIFN=pegylated interferon alfa-2a; CQ=chloroquine; UMF=umifenovir; RBV=ribavirin; ICN=itraconazole.

**Table 3:** Top 10 most used adjunctive drugs in each data source on days 0 to 30 after hospital admission. Data are treatment (percentage of patients admitted to hospital who received the medicine)

Ranking	USA: CUIMC (n=7353)	South Korea: HIRA (n=7599)	Spain: HM Hospitales (Spain; n=2544)	Spain: Hospital del Mar (n=2686)	USA: IQVIA Hospital CDM (n=77 853)	China: NFHCRD (n=304)	USA: Optum-EHR (n=36 717)	USA: Premier (n=156 187)	USA: STARR-OMOP (n=744)	USA: TRDW (n=326)	USA: VA-OMOP (n=10 951)
1	Vit D (88.1)	Fluoro (24.7)	Bemi (82.0)	Enox (52.2)	Vit D (84.8)	Fluoro (63.8)	Enox (53.7)	Enox (62.1)	CS (66.7)	Enox (58.3)	Vit D (95.3)
2	Enox (54.0)	H2RA (16.4)	Ceft (61.5)	Vit D (24.1)	Enox (55.5)	Vit C (58.6)	CS (46.5)	CS (38.2)	Hep (50.7)	Hep (51.2)	Enox (59.7)
3	CS (41.4)	Stat (13.5)	CS (44.4)	CS (23.1)	Ceft (50.2)	CS (40.8)	Ceft (37.6)	Stat (33.3)	α1b (38.3)	Ceft (40.5)	Stat (58.3)
4	Hep (38.1)	ARBs (13.1)	Fluoro (23.8)	Ceft (15.7)	CS (49.3)	Ig (22.0)	Stat (33.0)	Asp (27.2)	Enox (32.8)	Stat (36.2)	CS (40.9)
5	Stat (32.2)	CS (10.4)	Tocil (17.1)	ACEI (9.2)	Hep (28.9)	Amox (15.1)	Vit D (30.4)	H2RA (24.5)	Asp (28.6)	CS (34.0)	Asp (40.7)
6	Asp (27.6)	Vit C (9.7)	Asp (12.8)	ARB (4.5)	Stat (25.7)	ARB (8.9)	Asp (28.1)	Vit C (21.5)	Stat (28.4)	Asp (23.6)	Ceft (34.2)
7	Ceft (26.0)	Met (8.3)	Stat (12.7)	Met (3.0)	H2RA (23.0)	Met (6.9)	Hep (28.0)	DfXaI (12.6)	H2RA (23.8)	Vit D (17.8)	Hep (34.1)
8	H2RA (25.1)	Ceft (8.2)	ARB (12.6)	Stat (0.9)	Asp (21.1)	Stat (4.6)	H2RA (22.5)	α1b (12.0)	Ceft (13.3)	H2RA (17.2)	ACEI (25.9)
9	α1b (12.8)	Vit D (8.1)	ACEI (12.5)	Asp (0.7)	Vit C (19.9)	Ceft (3.6)	Vit C (15.9)	ACEI (11.0)	Tran (12.1)	α1b (16.3)	DfXaI (18.8)
10	ACEI (10.5)	DPP-4I (4.8)	α1b (3.5)	Hep (0.6)	α1b (10.0)	Enox (2.0)	ACEI (15.4)	ARB (8.5)	Fluoro (9.9)	ACEI (9.5)	Met (18.6)

CUIMC=Columbia University Irving Medical Center; HIRA=Health Insurance Review and Assessment; OMOP=Observational Medical Outcomes Partnership; Optum-EHR=Optum deidentified electronic health record dataset; STARR=STAnford medicine Research data Repository; TRDW=Tufts Research Data Warehouse; VA=Veterans Affairs; Vit=vitamin; Enox=enoxaparin; CS=corticosteroids; Hep=heparin; Stat=statins; Asp=aspirin; Ceft=ceftriaxone; H2RA=H2 receptor antagonist; α1b=α1 blockers; ACEI=angiotensin converting enzyme inhibitors; Fluoro=fluoroquinolones; ARB=angiotensin receptor blockers; Met=metformin; DDP-4I=dipeptidyl peptidase-4 inhibitors; Bemi=bemiparin; Tocil=tocilizumab; Ig=immunoglobulins; Amox=amoxicillin; DfXaI=direct factor Xa inhibitors; Tran=tranexamic acid.

**Fig 3:**
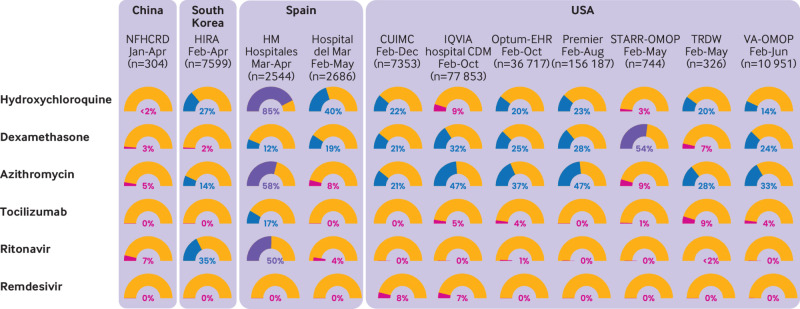
Percentage of any use (one day or more) of selected drugs on days 0 to 30 after hospital admission in patients with a positive test result for or diagnosis of covid-19. CUIMC=Columbia University Irving Medical Center; HIRA=Health Insurance Review and Assessment; OMOP=Observational Medical Outcomes Partnership; Optum-EHR=Optum deidentified electronic health record dataset; STARR=STAnford medicine Research data Repository; TRDW=Tufts Research Data Warehouse; VA=Veterans Affairs

Figure 3 shows the proportion of users of each of the drugs of interest both in patients admitted to hospital and in patients receiving intensive care, for each database (also see supplementary figures 2-5). Hydroxychloroquine was the most used drug, but this varied greatly, ranging from <2% in China to 85% in Spain (HM Hospitales). Chloroquine was used in China (11.5%). Dexamethasone was widely used in the US (20-54%). Both drugs had increased use in patients receiving intensive care services, except for dexamethasone in HM Hospitales. use of azithromycin varied, ranging from 58% in HM Hospitales to 5% in China. Lopinavir-ritonavir was used in South Korea, Spain, and China. Tocilizumab was used in some US settings (5-10% of patients) and in HM Hospitales. The use of adjunctive treatments increased substantially among patients who received intensive care, with the greatest augmentation seen for systemic corticosteroids, famotidine, heparin, and tocilizumab.

Supplementary figure 1 shows drug use before and during hospital admission. All the repurposed drugs were associated with increased drug use during hospital admission. Dexamethasone, corticosteroids, azithromycin, and tocilizumab also showed higher use during hospital admission compared with before hospital admission.

**Fig 4:**
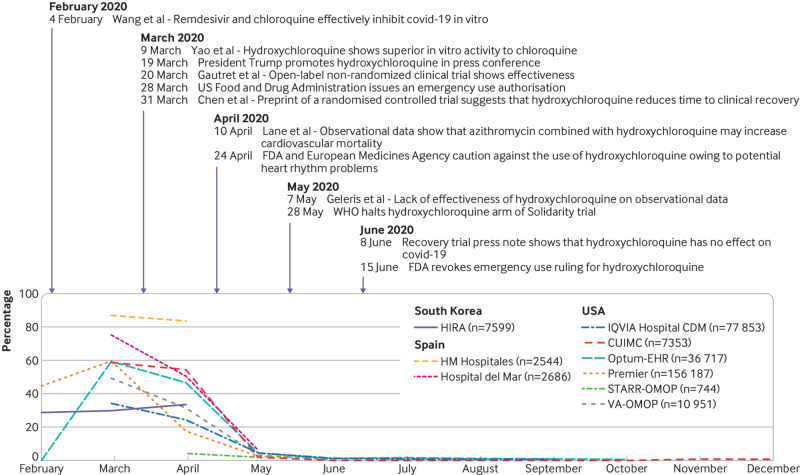
Time trends in hydroxychloroquine use on days 0 to 30 after hospital admission in patients with a positive test result for or diagnosis of covid-19 by month. CUIMC=Columbia University Irving Medical Center; HIRA=Health Insurance Review and Assessment; OMOP=Observational Medical Outcomes Partnership; Optum-EHR=Optum deidentified electronic health record dataset; STARR=STAnford medicine Research data Repository; VA=Veterans Affairs

**Fig 5:**
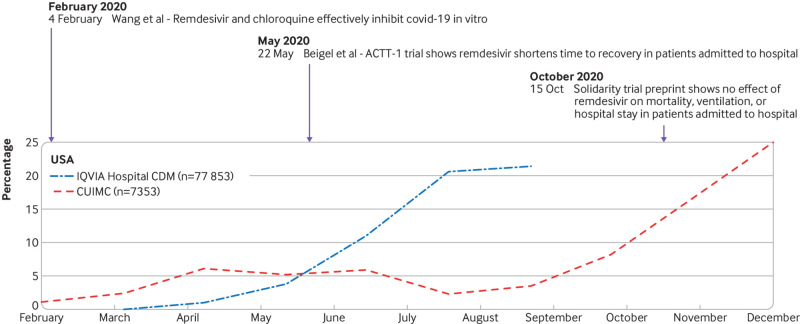
Time trends in remdesivir use on days 0 to 30 after hospital admission in patients with a positive test result for or diagnosis of covid-19 by month. ACTT-1=Adaptive COVID-19 Treatment Trial 1; CUIMC=Columbia University Irving Medical Center

**Fig 6:**
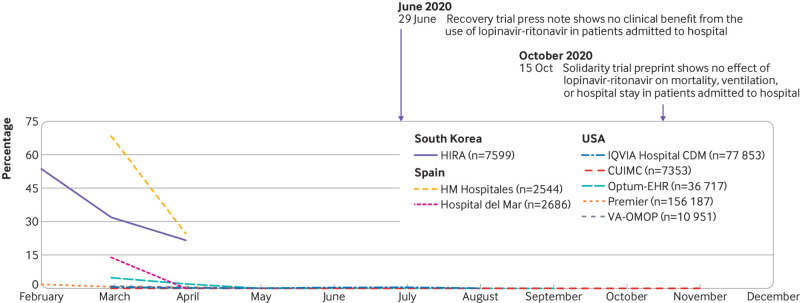
Time trends combined lopinavir and ritonavir use on days 0 to 30 after hospital admission in patients with a positive test result for or diagnosis of covid-19 by month. CUIMC=Columbia University Irving Medical Center; HIRA=Health Insurance Review and Assessment; OMOP=Observational Medical Outcomes Partnership; Optum-EHR=Optum deidentified electronic health record dataset; VA=Veterans Affairs

**Fig 7:**
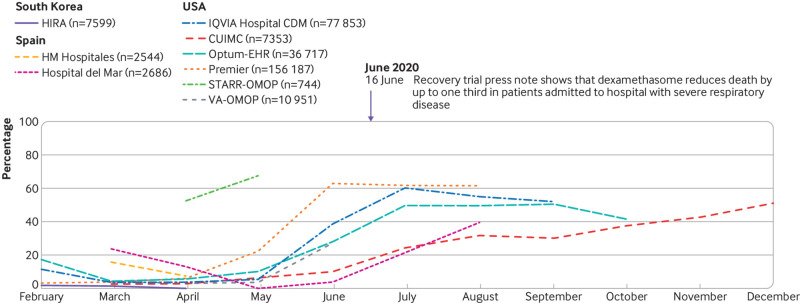
Time trends in dexamethasone use on days 0 to 30 after hospital admission in patients with a positive test result for or diagnosis of covid-19 by month. CUIMC=Columbia University Irving Medical Center; HIRA=Health Insurance Review and Assessment; OMOP=Observational Medical Outcomes Partnership; Optum-EHR=Optum deidentified electronic health record dataset; STARR=STAnford medicine Research data Repository; VA=Veterans Affairs

The management of covid-19 has changed substantially over time (see supplementary figure 6 and fig 4, fig 5, fig 6, and fig 7). The trends in hydroxychloroquine use show a rapid increase during February and March 2020, followed by a similarly rapid decline in May that continued until the end of the year. The upward trend coincided with reports of in vitro and in vivo activity and regulatory approval of hydroxychloroquine (fig 4). The downward trend coincided with reports on safety concerns and of lack of effectiveness. Dexamethasone was scarcely used in the first few months of the pandemic, except in the US (STARR-OMOP database). After the Recovery trial report in June 2020 showed a reduction in mortality associated with dexamethasone, use increased rapidly and then stabilised. Lopinavir-ritonavir was only used at the start of the pandemic in South Korea and Spain, with a downward trend over time. Remdesivir was only recorded in CUIMC and IQVIA Hospital CDM, and it showed a slight upward trend from June onwards.

## Discussion

This study reports on the use of repurposed and adjunctive drugs for the treatment of patients admitted to hospital with covid-19, including those who received intensive care, as recorded in electronic medical records and claims data across three continents. A total of 303 264 patients were admitted to hospital of whom 62 963 received intensive care for covid-19 in the US, South Korea, Spain, and China.

We observed high heterogeneity in the use of repurposed drugs, with great variability in the use of hydroxychloroquine both geographically and temporally. Similar trends were observed for azithromycin. Use of antiretrovirals also varied greatly, with lopinavir-ritonavir use ranging from 0% in the US (VA-OMOP) to 35% in South Korea (HIRA), and highest at 50% in Spain (HM Hospitales).

Adjunctive treatments have been extensively used for the prevention of or treatment for complications from covid-19, including antibiotics, anticoagulants, corticosteroids, vitamin D supplements, and, to a lesser degree, antihypertensives, antacids, statins, and metformin. The use of adjunctive drugs increased among patients who required intensive care.

Hydroxychloroquine has been given much publicity since the start of the pandemic. Its use has been supported or endorsed on the basis of misleading evidence from flawed but heavily publicised studies.^24^ ^25^ ^26^ Numerous randomised controlled trials have, however, shown no benefit. The Recovery trial of 1542 hospital patients with covid-19 treated with hydroxychloroquine showed no effects on 28 day mortality compared with usual care.^27^ Another randomised controlled trial studied the efficacy of hydroxychloroquine as post-exposure prophylaxis in 821 asymptomatic participants but was found not to prevent covid-19 illness after high or moderate exposure to covid-19.^28^ Hydroxychloroquine use increased rapidly when these studies appeared and were heavily publicised and politically endorsed. During March and April 2020, more than 50% of patients admitted to hospital with covid-19 were prescribed hydroxychloroquine. After several papers and regulatory agencies warned about potential side effects, especially when hydroxychloroquine was combined with azithromycin, the use of hydroxychloroquine began to decline. Finally, after the Solidarity trial halted its hydroxychloroquine arm and the Recovery trial presented definitive evidence against the use of hydroxychloroquine, the FDA revoked its approval for emergency use and prescribing decreased to almost 0% in all settings.^29^ ^30^

We found that azithromycin, a macrolide antibiotic with alleged antiviral efficacy against covid-19, was also widely prescribed. Although several guidelines in 2020 recommended the use of empirical antimicrobial treatment, not all advocated its use.^31^ In mid-December the Recovery trial showed no benefit from azithromycin in patients admitted to hospital with covid-19.^32^ We were not able to see the impact in trends as we only had data until December 2020.

Combined use of the protease inhibitors lopinavir and ritonavir was high in South Korea and Spain, with the other databases showing a much lower use. This was consistent with Korean and Spanish guidelines at the time of our study, which recommended protease inhibitors as antiviral treatments,^11^ ^12^ probably based on in vitro studies.^33^ The Recovery and Solidarity trials confirmed the lack of efficacy of lopinavir-ritonavir compared with usual care.^29^ ^30^

Remdesivir, another highly publicised antiviral, was only used in two databases, and in less than 25% of patients. Umifenovir in China was the most prescribed repurposed drug, consistent with Chinese guidelines and research.^34^ ^35^

Adjunctive drugs used to prevent covid-19 or treat complications differed noticeably worldwide. Use of corticosteroids ranged from about 10% of admitted patients in South Korea (HIRA) to 67% of patients in Stanford (California, US).

Before results were available from the Recovery trial, there was a wide debate on whether corticosteroids have a role in mitigating inflammatory organ injury.^36^ ^37^ Most clinical guidelines did not recommend the use of corticosteroids to treat covid-19,^31^ with notable exceptions.^24^ ^38^ The use of dexamethasone was low in almost all settings in our study until June 2020, when the Recovery trial showed its efficacy in reducing death in patients admitted to hospital with severe covid-19 related disease receiving respiratory support.^39^ Corticosteroid use in general appeared to increase slowly during the study period.

The use of anticoagulants in our study was higher than expected. Heparin use was widely prescribed in the US and Spain, but not in China or South Korea. Severe covid-19 has been associated with a coagulopathy, which when untreated leads to poor clinical outcomes.^40^ Although several randomised controlled trials are ongoing to evaluate the value of anticoagulation in patients with covid-19, interim guidelines recommend the use of anticoagulants for prophylaxis against thromboembolism.^12^ ^41^ The use of antibiotics also varied widely, as did the use of statins. Traditional Chinese medicines were not widely prescribed (<10% recorded in NFHCRD; see supplementary table 6).

The observed heterogeneity and rapid changes in drug use go hand in hand with the infodemic associated with covid-19. We have shown how the timings of bad science reporting, tweets, and political endorsements are aligned with changes in practice patterns and potentially influence the decisions of regulators.^42^ Retrospective evaluation of management and treatment practices during the pandemic are necessary^43^ to safeguard against the increase in use of unproven and potentially harmful treatments, during future waves of the pandemic and public health crises.

### Limitations of this study

Our study was based on routinely collected real world data (electronic health records and claims data), with the potential for misclassification of disease and treatments. We only included patients with a clinical diagnosis of covid-19 or a positive polymerase chain reaction test result during hospital admissions or 21 days previously; therefore, patients without a coded diagnosis would have been excluded even if they were suspected of having covid-19. The number of patients with covid-19 might also be underreported in clinical settings with scarce testing resources, especially when volumes of patients are high. In addition, medical conditions might be underreported because the absence of a medical code for the disease is interpreted as absence of the disease. Exposure misclassification is also possible; participating data sources varied in how drugs were captured (eg, hospital billing records, prescription orders, dispensing data).

Estimates for drug use on the date of hospital admission are particularly sensitive to misclassification and could conflate baseline concomitant drug history with immediate treatment on admission. We further explored this (see supplemental figure 1) and found that the drugs we focused on were not typically used before hospital admission according to the data sources.

We did not differentiate between drugs prescribed on the day of hospital admission from those in the following days or in the context of worsening disease. This could also mean that some drugs used at discharge (or those prescribed after discharge) could have appeared as being prescribed to patients during hospital admission. To avoid this, we censored on discharge when this information was available. Additionally, in most of the databases where this date was not available, only inpatient data were provided, so these drugs would not be recorded.

Another limitation of our study was the lack of information on dose and duration of drug treatments. These are important factors that would have added value to our understanding of the trends in prescribing, especially among those in high risk groups or those with greater susceptibility to drug related adverse events.

Although our study adds valuable information to the understanding of prescribing patterns for covid-19 in 2020, it only provides a snapshot of drug use in clinical practice. As new evidence continually emerges over time, drug use in covid-19 is likely to evolve rapidly. Although possibly not representative of global treatment patterns, our data provide a good oversight of inpatient treatment for covid-19 in real world practice settings during 2020. Our study cohorts included both academic hospitals (eg, at Columbia University and Stanford University) as well as nationwide data sources and including other less specialised treatment centres (eg, HIRA, IQVIA Hospital CDM). Owing to the varied settings we decided not to provide drug use by country or overall because it would not be representative of the underlying populations.

### Conclusions

Great interest has been shown in the safety and efficacy of drugs used to treat covid-19, but little evidence exists on the prescribing patterns for repurposed and adjuvant drugs in routine clinical practice. Our study shows how unproven drug treatments were used during the first months of the pandemic, with great heterogeneity between centres, and that they were quickly replaced by proven treatments.

**What is already known on this topic**

Repurposed drugs are commonly used to manage novel diseases and conditions with no available treatments
Hydroxychloroquine was widely used to treat patients with covid-19 during the early phases of the pandemic
At the start of the pandemic, guidelines recommended concomitant treatments, including immune based drugs, antithrombotics, and antibiotics

**What this study adds**

The use of repurposed drugs to manage patients with covid-19 varied widely in the US, South Korea, Spain, and China during 2020 and a wide range of adjunctive treatments were used
Emerging clinical data highlighting concerns about the safety and efficacy of hydroxychloroquine and azithromycin affected use both geographically and temporally
The use of corticosteroids during 2020, however, steadily increased, with little use in the early stages of the pandemic (February to April)

## Web extra

Extra material supplied by authors

Web appendix: Supplementary material

Web appendix: Supplementary figure 4

Web appendix: Supplementary figure 5

Web appendix: Supplementary figure 6

Web appendix: Supplementary table 6

## Notes

Contributors: All authors designed the study, interpreted the results, and reviewed the manuscript. AGS designed and prepared the statistical package and set up the web app. AP-U, LYHL, AGS, and DP-A wrote the first draft of the manuscript. AP-U produced and designed the figures and tables. AP-U and AGS are joint first authors. The corresponding author attests that all listed authors meet authorship criteria and that no others meeting the criteria have been omitted. AGS, GH, SCY, TDS, CR, KK, MG, PBR, JDP, KEL, and MM are the guarantors.

Ethical approval: All the data partners received institutional review board (IRB) approval or exemption. STARR-OMOP had approval from IRB panel #8 (RB-53248) registered to Leland Stanford Junior University under the Stanford Human Research Protection Program (HRPP). The use of Veterans Affairs data was reviewed by the Department of Veterans Affairs Central IRB and was determined to meet the criteria for exemption under Exemption Category 4(3) and approved the request for waiver of the Health Insurance Portability and Accountability Act of 1996 authorisation. The research was approved by the Columbia University IRB as an Observational Health Data Sciences and Informatics network study. The IRB number for use of HIRA data was AJIB-MED-EXP-20-065). The use of HM Hospitales data as approved by the Clinical Research Ethics Committee of the IDIAPJGol (project code: 20/070-PCV). The collection and usage of the data for clinical research in NFHCRD was approved by the IRB of Nanfang Hospital. The use of data from the Hospital del Mar database (HMAR) was approved by the Parc de Salut Mar Clinical Research Ethics Committee.

Data sharing: Analyses were performed locally, and the patient level data are not readily available to be shared. The analytic code is, however, available at: https://github.com/ohdsi-studies/Covid19CharacterizationCharybdis and results are available at https://data.ohdsi.org/Covid19CharacterizationCharybdis/

The lead authors (AP-U and AGS) affirm that the manuscript is an honest, accurate, and transparent account of the study being reported; that no important aspects of the study have been omitted; and that any discrepancies from the study as planned have been explained.

Dissemination to participants and related patient and public communities: No public or lay dissemination activities have been organised to date. The Observational Health Data Sciences and Informatics community and contributing academic institutions, including the University of Oxford, will issue a press release for general media and the lay audience once the manuscript is available to the public.

Provenance and peer review: Not commissioned; externally peer reviewed.
