# Use of repurposed and adjuvant drugs in hospital patients with covid-19: multinational network cohort study

**DOI:** 10.1136/bmj.n1277

**Published:** 2021-05-21

A few changes have been made to this paper by Prats-Uribe and colleagues (*BMJ* 2021;373:n1038, doi:10.1136/bmj.n1038, published 11 May 2021). Authors Hong Zhu and Li Lui should be affiliated with Nanfang Hospital [not Nanfang University], Southern Medical University, Guangzhou, China. In the fourth sentence of the data sources paragraph of the Methods, the period during which inpatient electronic health record data was collected from Hospital del Mar in Spain should have been February to August [not May] 2020. In table 1, the age distribution for participants from Hospital del Mar, Spain, has been updated. The ethical approval statement has been amended to acknowledge that use of data from the Hospital del Mar database (HMAR) was approved by the Parc de Salut Mar Clinical Research Ethics Committee. Finally, table 1 in the supplementary file has been amended to include a description of the HMAR database.

