# Supplementary material for: Use of repurposed and adjuvant drugs in hospital patients with covid-19: multinational network cohort study
Source: BMJ. 2021 May 11;373:n1038. doi: 10.1136/bmj.n1038 (PMC8111167; doi:10.1136/bmj.n1038)
Supplement: Supplementary file 1 — Web appendix: Supplementary material [file praa062143.ww.pdf]

Supplementary Table 1. Description of databases used

ID	Database Name	Setting	Patient Count	Months	Description
CUIMC	Columbia University Irving Medical Center	Hospital EHR – New York, USA	>6M	Feb-Dec	The clinical data warehouse of NewYork-Presbyterian Hospital/Columbia University Irving Medical Center, New York, NY (a major academic medical center) based on its current and previous electronic health record systems, with data spanning over 30 years and including over 6 million patients
HIRA	Health Insurance Review & Assessment Service	Claims – South Korea	0.24M	Feb-Apr	National administrative claims data from a single insurance service from South Korea. It contains the observational medical records (including both inpatient and outpatient) of a patient while they are qualified to get the national medical insurance.
HM	HM Hospitales	Hospital EHR – Spain	>2300 COVID cases	Mar-Apr	Hospital de Madrid (HM) Hospitals data are made available through partnership with IDIAPJGol. The HM Hospitals database covers in-patient care delivered across a network of 17 private hospitals in Spain between 1st of March and 24th of April 2020. HM Hospitals database covers more than 2300 confirmed COVID-19 cases, and all in-patient hospital care, including the data of admission, conditions, procedures and medicines dispensed in hospital, date of discharge, and date of known death or date of end of follow-up in the database.
HMAR	Hospital del Mar	Hospital EHR – Barcelona, Spain	0.6M	2000-Late 2020	Anonymized data from the Electronic Medical Records from Hospital del Mar (Barcelona, Spain). Hospital belonging to the Spanish National Health System (public), attending the Eastern area of Barcelona City. Includes hospital data collected routinely in the clinical practice, both structured and unstructured information, extracted using a free text analysis tool (with natural language processing): Inpatient (hospital) care, Outpatient specialist care, Emergency Room Visits and partial information from other settings like primary care and pharmacy care present in free text notes from EMRs. All subjects with at least one healthcare encounter with the Hospital within approximately the last 20 years are included (approximately 0.6 M subjects, with more than 5 M hospitalizations/visits). Hospital del Mar data are made available through collaboration with TFS / IOMED.

IQVIA Hospital CDM	IQVIA Hospital Charge Datamaster (CDM)	National hospital billing – United States	>86M	Feb-Oct	IQVIA Hospital CDM are data from hospital charge data masters (CDM) and collected from resource management software within short-term, acute-care and non-federal hospitals. Hospital charge masters are not a true EHR. It is an in-patient specific view of all things that are ordered in a hospital, captured for revenue purposes. The data covers over 86 million patients with 530 million medical events from January 2009 through early October 2020, derived from both public and private hospitals. These data do not overlap with federally funded hospitals, such as the Department of Veterans Affairs.
Nanfang	Nanfang Hospital COVID-19 Research Database (NFHCRD)	Hospital EHR – Hubei, China	>400 COVID cases	Jan-Apr	The clinical data warehouse of The People's Hospital of HongHuï Hubei China based on its current and previous electronic health record systems, with data spanning over 4 months and including over 400 patients.
OPTUM EHR	Optum EHR	National EHR – USA	>95M	Feb-Oct	Optum®de-identified Electronic Health Record Dataset is derived from dozens of healthcare provider organizations in the United States (that include more than 700 hospitals and 7,000 Clinics treating more than 103 million patients) receiving care in the United States. The medical record data includes clinical information, inclusive of prescriptions as prescribed and administered, lab results, vital signs, body measurements, diagnoses, procedures, and information derived from clinical Notes using Natural Language Processing (NLP).
PREMIER	Premier	National hospital billing – USA	227M	Feb-Aug	The Premier Healthcare Database contains complete clinical coding, hospital cost, and patient billing data from approximately 700 hospitals throughout the United States representing 20% of inpatient hospital stays. Premier collects data from participating hospitals in its health care alliance. The Premier health care alliance was formed for hospitals to share knowledge, improve patient safety, and reduce risks. Participation in the Premier health care alliance is voluntary. Although the database excludes federally funded hospitals (e.g., Veterans Affairs), the hospitals included are nationally representative based on bed size, geographic region, location (urban/rural) and teaching hospital status. The database contains a date-stamped log of all billed items by cost-accounting department including

					medications; laboratory, diagnostic, and therapeutic services; and primary and secondary diagnoses for each patient’s hospitalization.
STARR-OMOP	STARR-OMOP	Hospital EHR – California, USA	>2.7M	Feb-May	STAnford medicine Research data Repository, a clinical data warehouse containing live Epic data from Stanford Health Care (a major academic medical center), the Stanford Children’s Hospital, the University Healthcare Alliance and Packard Children's Health Alliance clinics and other auxiliary data from Hospital applications such as radiology PACS. STARR platform is developed and operated by Stanford Medicine Research IT team and is made possible by Stanford School of Medicine Research Office. https://arxiv.org/abs/2003.10534
TRDW	Tufts Medical Center Research Data Warehouse (TRDW)	Hospital EHR - Massachusetts, USA	>1M	Feb-May	Electronic medical record data on approximately 1 million patients who received care beginning in 2006 at Tufts Medical Center (TMC). TMC is an academic medical center that includes Tuft Medical Center's main downtown Boston hospital for adult patients, the Floating Hospital for Children, and associated primary and specialty care clinics. TRDW contains TMC's EHR data fused with data on the same patients from TMC's CoC accredited tumor registry, its oncology EHR, and death data from the Massachusetts State Registry of Vital Statistics. EHR data streams ingested into TRDW include controlled vocabulary data on all domains except cost and select free text sources and devices.
VA OMOP	Department of Veterans Affairs (VA OMOP)	Hospital EHR - USA	>9M	Feb-Jun	VA OMOP data reflects the national Department of Veterans Affairs health care system (a federally funded system), which is the largest integrated provider of medical and mental health services in the United States. Care is provided at 170 VA Medical Centers and 1,063 outpatient sites serving more than 9 million enrolled Veterans each year.

Supplementary Table 2. Codes used for Baseline characteristics

Condition	Window	OMOP Concept ID	SNOMED Code
Anemia	day -30 to -1	439777	271737000
Anxiety disorder	day -30 to -1	442077	197480006
Asthma	day -30 to -1	317009	195967001
Atrial fibrillation	day -30 to -1	313217	49436004
Chronic liver disease	day -30 to -1	4212540	328383001
Chronic obstructive lung disease	day -30 to -1	255573	13645005
Dementia	day -30 to -1	4182210	52448006
Diabetes mellitus	day -30 to -1	201820	73211009
Gastroesophageal reflux disease	day -30 to -1	318800	235595009
Heart disease	day -30 to -1	321588	56265001
Heart failure	day -30 to -1	316139	84114007
Hyperlipidemia	day -30 to -1	432867	55822004
Hypertensive disorder	day -30 to -1	316866	38341003
Insomnia	day -30 to -1	436962	193462001
Ischemic heart disease	day -30 to -1	4185932	3738000
Low back pain	day -30 to -1	194133	279039007
Malignant neoplastic disease	day -30 to -1	443392	363346000
Osteoarthritis of hip	day -30 to -1	4079749	239872002
Osteoarthritis of knee	day -30 to -1	4079750	239873007
Peripheral vascular disease	day -30 to -1	321052	400047006
Renal impairment	day -30 to -1	4030518	236423003
Venous thrombosis	day -30 to -1	444247	111293003
Viral hepatitis	day -30 to -1	4291005	414545008

Supplementary Table 3. List of drugs of interest and codes used to identify

Type	Name	OMOP Concept ID	Vocabulary	Vocabulary Name	Vocabulary Code
**Repurposed**	Hydroxychloroquine	1777087	RxNorm	hydroxychloroquine	5521
**Repurposed**	Chloroquine	1792515	RxNorm	chloroquine	2393
**Repurposed**	Azithromycin	1734104	RxNorm	azithromycin	18631
**Repurposed**	Lopinavir	1738170	RxNorm	lopinavir	195088
**Repurposed**	Ritonavir	1748921	RxNorm	ritonavir	85762
**Repurposed**	Interferon beta	19109079	RxNorm	interferon-beta	4381
**Repurposed**	Peginterferon alfa-2a	1714165	RxNorm	peginterferon alfa-2a	120608
**Repurposed**	Peginterferon alfa-2b	1797155	RxNorm	peginterferon alfa-2b	253453
**Repurposed**	Favipiravir	32765	RxNorm Extension	Favipiravir	OMOP4873976
**Repurposed**	Remdesivir	37499271	RxNorm	remdesivir	2284718
**Repurposed**	Oseltamivir	1799139	RxNorm	oseltamivir	260101
**Repurposed**	Oseltamivir carboxylate	45775353	RxNorm	oseltamivir carboxylate	1546279
**Repurposed**	Ribavirin	1762711	RxNorm	ribavirin	9344
**Repurposed**	Ivermectin	1784444	RxNorm	ivermectin	6069
**Repurposed**	Itraconazole	1703653	RxNorm	itraconazole	28031
**Repurposed**	Umifenovir	32764	RxNorm Extension	Umifenovir	OMOP4873975
**Adjunctive therapies**	Fluoroquinolones	21603007	ATC	Fluoroquinolones	J01MA
**Adjunctive therapies**	Amoxicillin	1713332	RxNorm	amoxicillin	723
**Adjunctive therapies**	Ceftriaxone	1777806	RxNorm	ceftriaxone	2193
**Adjunctive therapies**	Interleukin inhibitors	21603914	ATC	Interleukin inhibitors	L04AC
**Adjunctive therapies**	Tocilizumab	40171288	RxNorm	tocilizumab	612865
**Adjunctive therapies**	Siltuximab	44818461	RxNorm	siltuximab	1535218
**Adjunctive therapies**	Sarilumab	1594587	RxNorm	sarilumab	1923319
**Adjunctive therapies**	Ustekinumab	40161532	RxNorm	ustekinumab	847083
**Adjunctive therapies**	Anakinra	1114375	RxNorm	anakinra	72435
**Adjunctive therapies**	Baricitinib	1510627	RxNorm	baricitinib	2047232
**Adjunctive therapies**	Tofacitinib	42904205	RxNorm	tofacitinib	1357536
**Adjunctive therapies**	TNF inhibitors	21603907	ATC	Tumor necrosis factor alpha (TNF-a) inhibitors	L04AB
**Adjunctive therapies**	Adalimumab	1119119	RxNorm	adalimumab	327361
**Adjunctive therapies**	Etanercept	1151789	RxNorm	etanercept	214555
**Adjunctive therapies**	Infliximab	937368	RxNorm	infliximab	191831
**Adjunctive therapies**	Warfarin	1310149	RxNorm	warfarin	11289
**Adjunctive therapies**	Acenocoumarol	19024063	RxNorm	acenocoumarol	154
**Adjunctive therapies**	Dicumarol	1325124	RxNorm	dicumarol	1598
**Adjunctive therapies**	Heparin	1367571	RxNorm	heparin	5224
**Adjunctive therapies**	Heparinoids	19113045	RxNorm	heparinoids	5228
**Adjunctive therapies**	Enoxaparin	1301025	RxNorm	enoxaparin	67108
**Adjunctive therapies**	Reviparin	19129274	RxNorm	reviparin	75960
**Adjunctive therapies**	Dalteparin	1301065	RxNorm	dalteparin	67109
**Adjunctive therapies**	Bemiparin	19069137	RxNorm	bemiparin	280611

**Adjunctive therapies**	Edoxaban	45892847	RxNorm	edoxaban	1599538
**Adjunctive therapies**	Direct factor Xa inhibitors	43534760	ATC	Direct factor Xa inhibitors	B01AF
**Adjunctive therapies**	Apixaban	43013024	RxNorm	apixaban	1364430
**Adjunctive therapies**	Rivaroxaban	40241331	RxNorm	rivaroxaban	1114195
**Adjunctive therapies**	Dabigatran	45775372	RxNorm	dabigatran	1546356
**Adjunctive therapies**	Dabigatran etexilate	40228152	RxNorm	dabigatran etexilate	1037042
**Adjunctive therapies**	Clopidogrel	1322184	RxNorm	clopidogrel	32968
**Adjunctive therapies**	Aspirin	1112807	RxNorm	aspirin	1191
**Adjunctive therapies**	Triflusal	19042778	RxNorm	triflusal	38655
**Adjunctive therapies**	Cangrelor	46275677	RxNorm	cangrelor	1656052
**Adjunctive therapies**	Ticagrelor	40241186	RxNorm	ticagrelor	1116632
**Adjunctive therapies**	Prasugrel	40163718	RxNorm	prasugrel	613391
**Adjunctive therapies**	Ticlopidine	1302398	RxNorm	ticlopidine	10594
**Adjunctive therapies**	Cilostazol	1350310	RxNorm	cilostazol	21107
**Adjunctive therapies**	ACE inhibitors	21601783	ATC	ACE INHIBITORS, PLAIN	C09A
**Adjunctive therapies**	ARBs	21601822	ATC	ANGIOTENSIN II RECEPTOR BLOCKERS (ARBs), PLAIN	C09C
**Adjunctive therapies**	Losartan	1367500	RxNorm	losartan	52175
**Adjunctive therapies**	Corticosteroids	21602723	ATC	CORTICOSTEROIDS FOR SYSTEMIC USE, PLAIN	H02A
**Adjunctive therapies**	Dexamethasone	1518254	RxNorm	dexamethasone	3264
**Adjunctive therapies**	Statins	21601855	ATC	HMG CoA reductase inhibitors	C10AA
**Adjunctive therapies**	Metformin	1503297	RxNorm	metformin	6809
**Adjunctive therapies**	DPP-4 inhibitors	21600783	ATC	Dipeptidyl peptidase 4 (DPP-4) inhibitors	A10BH
**Adjunctive therapies**	Linagliptin	40239216	RxNorm	linagliptin	1100699
**Adjunctive therapies**	Sitagliptin	1580747	RxNorm	sitagliptin	593411
**Adjunctive therapies**	SGLT2 inhibitors	1123627	ATC	Sodium-glucose co-transporter 2 (SGLT2) inhibitors	A10BK
**Adjunctive therapies**	Dapagliflozin	44785829	RxNorm	dapagliflozin	1488564
**Adjunctive therapies**	GLP1 inhibitors	44785829	RxNorm	dapagliflozin	1488564
**Adjunctive therapies**	H2 receptor antagonist	21600081	ATC	H2-receptor antagonists	A02BA
**Adjunctive therapies**	Famotidine	953076	RxNorm	famotidine	4278
**Adjunctive therapies**	Alpha-1 blockers	21601698	ATC	Alpha and beta blocking agents	C07AG
**Adjunctive therapies**	Prazosin	1350489	RxNorm	prazosin	8629
**Adjunctive therapies**	Immunoglobulins	21601254	ATC	IMMUNOGLOBULINS	J06B
**Adjunctive therapies**	BCG vaccine	19086176	RxNorm	BCG, live, Tice strain	221050
**Adjunctive therapies**	Colchicine	1101554	RxNorm	colchicine	2683

**Adjunctive therapies**	Nitazoxanide	1715315	RxNorm	nitazoxanide	31819
**Adjunctive therapies**	Nitric oxide	19020068	RxNorm	nitric oxide	7442
**Adjunctive therapies**	Pirfenidone	45775206	RxNorm	pirfenidone	1592254
**Adjunctive therapies**	Defibrotide	42898933	RxNorm	defibrotide	1311089
**Adjunctive therapies**	Camostat	43009043	RxNorm Extension	camostat mesilate	OMOP4700469
**Adjunctive therapies**	Sargramostim	1308432	RxNorm	sargramostim	69634
**Adjunctive therapies**	Thalidomide	19137042	RxNorm	thalidomide	10432
**Adjunctive therapies**	Vitamin C	19011773	RxNorm	ascorbic acid	1151
**Adjunctive therapies**	Vitamin D	21600815	ATC	Vitamin D and analogues	A11CC
**Adjunctive therapies**	Ibrutinib	44507848	RxNorm	ibrutinib	1442981
**Adjunctive therapies**	Tranexamic acid	1303425	RxNorm	tranexamic acid	10691
**Adjunctive therapies**	Ruxolitinib	40244464	RxNorm	ruxolitinib	1193326
**Adjunctive therapies**	Siltuximab	44818461	RxNorm	siltuximab	1535218
**Adjunctive therapies**	Bevacizumab	1397141	RxNorm	bevacizumab	253337
**Adjunctive therapies**	Fingolimod	40226579	RxNorm	fingolimod	1012892
**Adjunctive therapies**	Eculizumab	19080458	RxNorm	eculizumab	591781

Supplementary Table 4. Baseline Characteristics of patients hospitalised with COVID-19 and requiring intensive care services, stratified by data source

	Spain	Spain	USA	USA	USA	USA
	HM-Hospitales	HMAR	IQVIA Hospital CDM	OPTUM - EHR	PREMIER	VA-OMOP
	n=1,397	n=228	n=18,274	n=4,425	n=36,735	n=1,904
**Sex**						
Female	39%	34%	41%	38%	40%	6%
**Age**						
00-04			2%	1%	1%	0%
05-09			0%	0%	0%	0%
10-14			0%	0%	0%	0%
15-19			0%	1%	0%	1%
20-24			1%	1%	1%	2%
25-29	1%	<2%	1%	2%	1%	3%
30-34	1%	4%	2%	2%	2%	5%
35-39	1%	<2%	3%	3%	3%	7%
40-44	3%	4%	4%	4%	4%	12%
45-49	4%	4%	5%	5%	5%	14%
50-54	5%	9%	7%	8%	7%	26%
55-59	8%	9%	10%	11%	10%	15%
60-64	10%	11%	13%	14%	12%	7%
65-69	11%	12%	14%	14%	14%	5%
70-74	12%	15%	14%	13%	13%	3%
75-79	13%	17%	12%	9%	11%	1%
80-84	10%	8%	13%	7%	8%	0%
85-89	12%	4%		7%	7%	0%
90-94	8%	<2%			2%	0%
≥95	3%	<2%				1%
**Comorbidities**						
Anemia		<2%	2%	8%	6%	13%
Anxiety disorder		<2%	1%	2%	1%	13%
Asthma		<2%	10%	30%	30%	3%
Atrial fibrillation		<2%	5%	13%	11%	12%
Chronic liver disease		<2%	3%	16%	15%	3%
COPD		<2%	5%	16%	14%	19%
Dementia		<2%	9%	40%	25%	5%
Diabetes mellitus		3%	3%	14%	12%	30%
Gastroesophageal reflux disease		<2%	1%	6%	4%	9%
Heart disease		3%	10%	45%	37%	34%
Heart failure		<2%	8%	34%	30%	14%
Hyperlipidemia		3%	0%	3%	2%	21%
Hypertensive disorder		4%	5%	27%	25%	34%
Insomnia		<2%	2%	12%	10%	3%
Ischemic heart disease		<2%	2%	6%	4%	6%
Low back pain		<2%	0%	4%	3%	6%
Malignant neoplastic disease		<2%	8%	35%	32%	9%
Osteoarthritis of hip		<2%	0%	1%	0%	1%
Osteoarthritis of knee		<2%	0%	2%	1%	2%
Peripheral vascular disease		<2%	2%	5%	7%	5%
Renal impairment		<2%	2%	16%	10%	28%
Venous thrombosis		<2%	1%	2%	2%	2%
Viral hepatitis		<2%	1%	2%	2%	2%
**Mortality**	18%		45%	17%	6%	45%

Supplementary Table 5. Top 5 most used repurposed drugs and top 10 most used adjunctive drugs in each data source in patients hospitalised with COVID-19 and requiring intensive care services on day 0 to 30 after hospitalization. Percentage (%) of patients receiving intensive services who received each medicine.

Repurposed

	HM		HMAR		IQVIAHospitalCDM		Optum		Premier		VA	
	Intensive Services		Intensive Services		Intensive Services		Intensive Services		Intensive Services		Intensive Services	
N	Treatment	%	Treatment	%	Treatment	%	Treatment	%	Treatment	%	Treatment	%
1	Hydroxychloroquine	87.6%	Hydroxychloroquine	80.7%	Azithromycin	43.7%	Azithromycin	39.3%	Azithromycin	42.6%	Azithromycin	36.1%
2	Azithromycin	62.8%	Azithromycin	42.5%	Hydroxychloroquine	12.1%	Hydroxychloroquine	26.2%	Hydroxychloroquine	28.8%	Hydroxychloroquine	21.2%
3	Ritonavir	52.0%	Ritonavir	15.4%	Remdesivir	9.7%	Ritonavir	2.1%	Ritonavir	0.7%	Ritonavir	0.7%
4	Oseltamivir	8.7%			Ivermectin	1.0%	Ivermectin	0.5%	Oseltamivir	0.5%	Ivermectin	0.6%
5	Chloroquine	0.4%			Oseltamivir	0.5%					Lopinavir	0.5%

Adjunctive drugs

	HM		HMAR		IQVIAHospitalCDM		Optum		Premier		VA	
	Intensive Services		Intensive Services		Intensive Services		Intensive Services		Intensive Services		Intensive Services	
N	Treatment	%	Treatment	%	Treatment	%	Treatment	%	Treatment	%	Treatment	%
1	Bemiparin	89.3%	Enoxaparin	96.5%	Vitamin D	91.8%	Corticosteroids	67.5%	Enoxaparin	63.3%	Vitamin D	98.3%
2	Ceftriaxone	68.6%	Vitamin D	69.3%	Corticosteroids	68.8%	Heparin	62.1%	Corticosteroids	49.4%	Statins	63.2%
3	Corticosteroids	55.4%	Corticosteroids	67.1%	Enoxaparin	59.0%	Enoxaparin	61.9%	H2 receptor antagonist	43.0%	Enoxaparin	61.4%
4	Fluoroquinolones	27.8%	Ceftriaxone	46.1%	Heparin	46.7%	H2 receptor antagonist	56.5%	Statins	34.2%	Corticosteroids	59.3%
5	Interleukin inhibitors	25.8%	ACE inhibitors	14.0%	Ceftriaxone	44.2%	Ceftriaxone	42.6%	Aspirin	29.8%	Heparin	54.8%
6	Tocilizumab	25.7%	ARBs	7.0%	H2 receptor antagonist	38.8%	Vitamin D	42.3%	Vitamin C	23.1%	Aspirin	44.4%
7	Statins	15.0%	Metformin	6.1%	Statins	28.9%	Statins	33.5%	Alpha-1 blockers	17.8%	Ceftriaxone	36.3%
8	Aspirin	15.0%	Heparin	3.1%	Aspirin	24.4%	Aspirin	31.4%	Direct factor Xa inhibitors	14.4%	H2 receptor antagonist	31.7%
9	ACE inhibitors	14.7%	Aspirin	2.2%	Vitamin C	21.6%	Alpha-1 blockers	23.2%	Heparin	13.2%	ACE inhibitors	23.5%
10	ARBs	13.8%	Statins	<2.2%	Alpha-1 blockers	15.4%	Vitamin C	18.9%	ACE inhibitors	8.7%	Alpha-1 blockers	23.2%

Supplementary Figure 1. Standardized mean differences (SMD) of prevalence of use of adjuvant therapies before and after hospital admission with a positive test or diagnosis of COVID-19.

To assess which drugs could have been already being used before hospitalisation, we compared the prevalence of use of therapies on -30 to -1 days before diagnosis and 0 to 30 days after (for all databases in which pre-index data was available). For this, we computed standardized mean differences (SMD), a widely used method to detect differences, and we consider different those with SMD>0.1; or those without use before diagnosis:

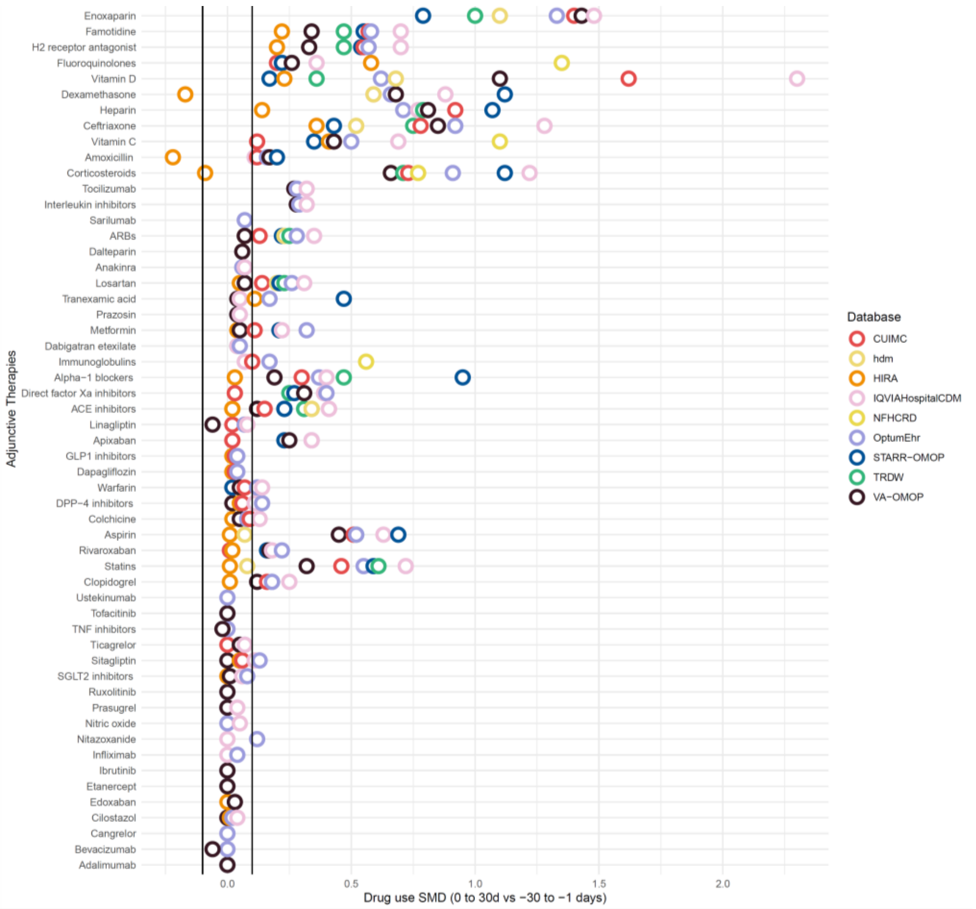

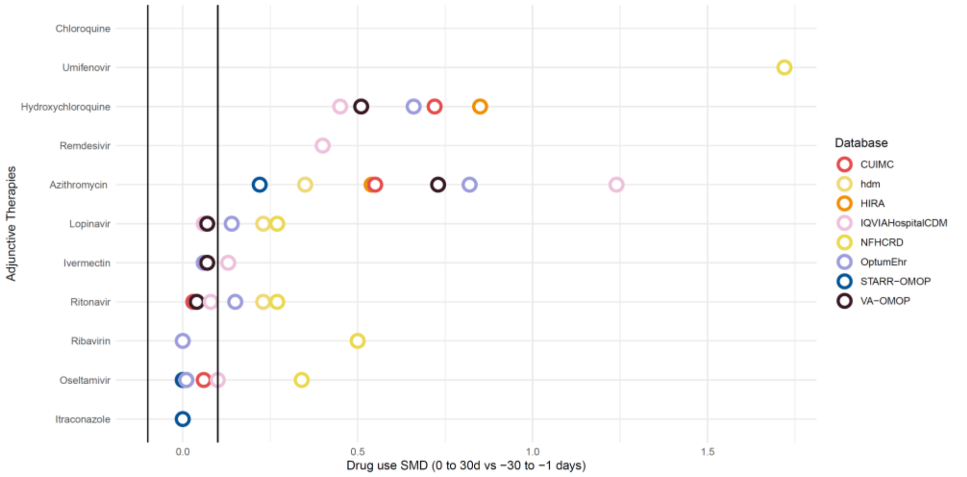

Supplementary Figure 2. Lollipop plot showing proportion of patients receiving adjunctive (a) and repurposed (b) therapies in hospitalized or intensive services settings with a positive test or diagnosis of COVID-19.

a)

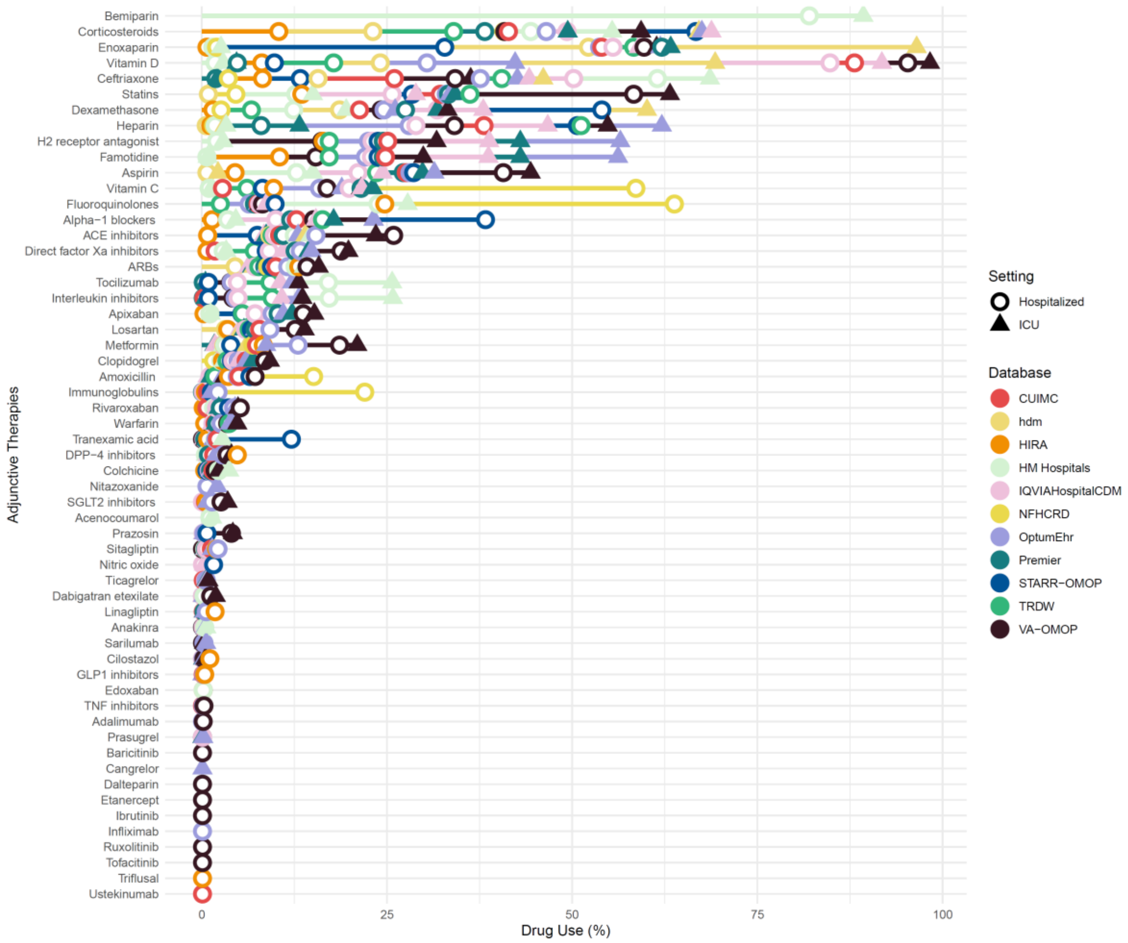

b)

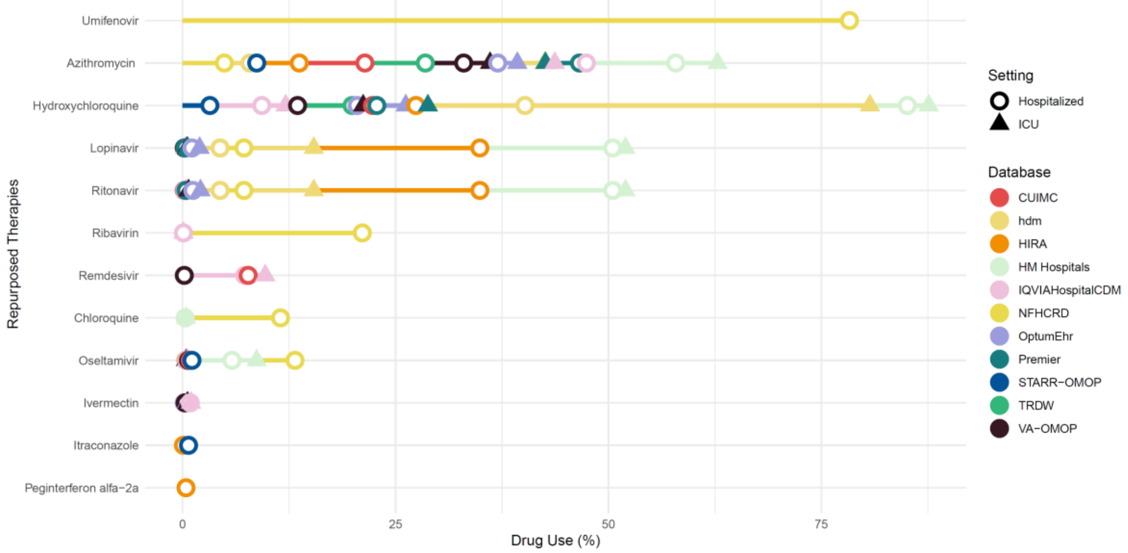

Supplementary Figure 3. Gauge Plots of selected drugs prescribed to patients hospitalized with COVID-19 requiring intensive care Feb-Dec 2020

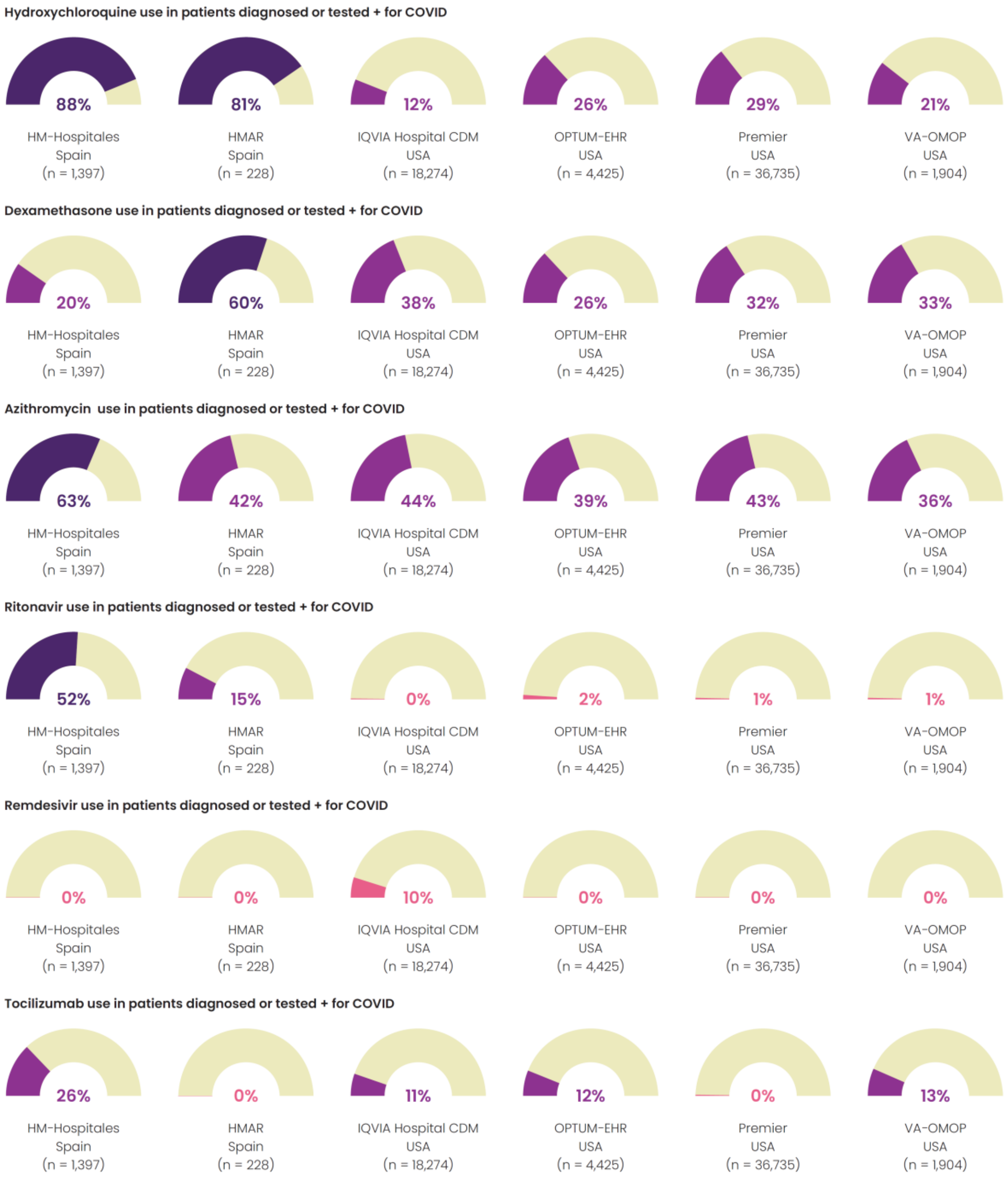

Supplementary Table 6. Drug use (%) for all drugs present in the NFHCRD database

See attached xlsx (Supplementary_Table_6_NFHCRD_Drugs.xlsx)

Supplementary Figure 4. Gauge Plots of all studied drugs prescribed to patients hospitalised with COVID-19 Feb-Dec 2020

See attached pdf (Supp_Figure_4_Gauges_Hospitalised.pdf)

Supplementary Figure 5. Gauge Plots of all studied drugs prescribed to patients hospitalised with COVID-19 requiring intensive care Feb-Dec 2020

See attached pdf (Supp_Figure_5_Gauges_ICU.pdf)

Supplementary Figure 6. Time trends of all studied drugs prescribed to patients hospitalised with COVID-19 Feb-Dec 2020

See attached pdf (Supp_Figure_6_Trends.pdf)
