# Supplementary material for: Use of repurposed and adjuvant drugs in hospital patients with covid-19: multinational network cohort study
Source: BMJ. 2021 May 11;373:n1038. doi: 10.1136/bmj.n1038 (PMC8111167; doi:10.1136/bmj.n1038)

ACE inhibitors use in patients diagnosed or tested + for COVID

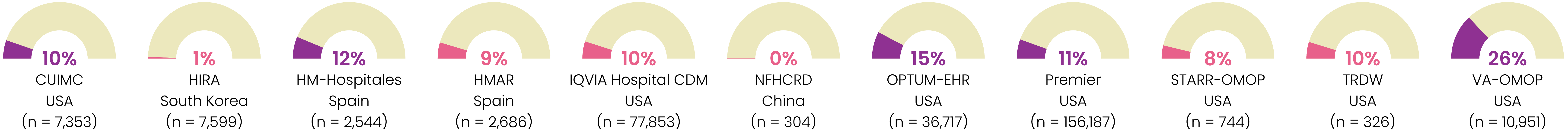

Acenocoumarol use in patients diagnosed or tested + for COVID

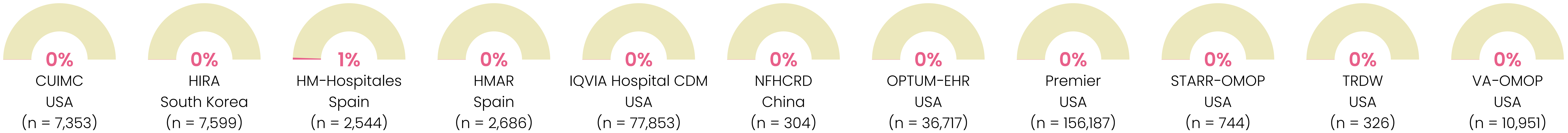

Adalimumab use in patients diagnosed or tested + for COVID

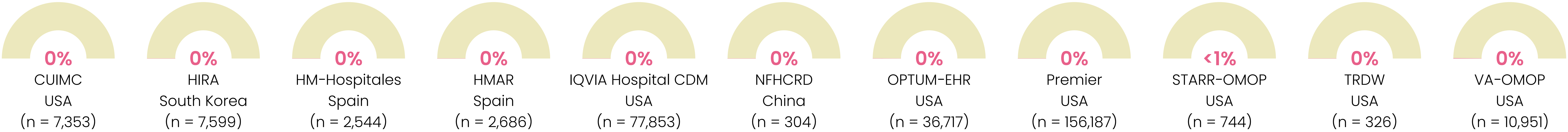

Alpha-1 blockers use in patients diagnosed or tested + for COVID

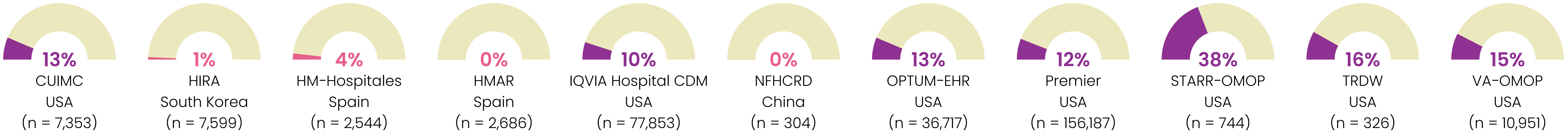

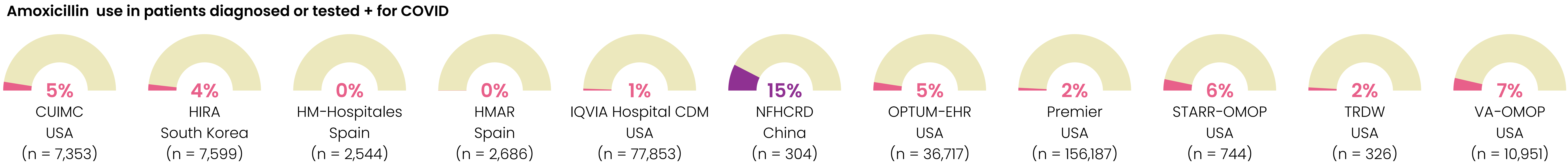
Amoxicillin use in patients diagnosed or tested + for COVID
5%
CUIMC
USA
(n = 7,353)
4%
HIRA
South Korea
(n = 7,599)
0%
HM-Hospitales
Spain
(n = 2,544)
0%
HMAR
Spain
(n = 2,686)
1%
IQVIA Hospital CDM
USA
(n = 77,853)
15%
NFHCRD
China
(n = 304)
5%
OPTUM-EHR
USA
(n = 36,717)
2%
Premier
USA
(n = 156,187)
6%
STARR-OMOP
USA
(n = 744)
2%
TRDW
USA
(n = 326)
7%
VA-OMOP
USA
(n = 10,951)

Anakinra use in patients diagnosed or tested + for COVID

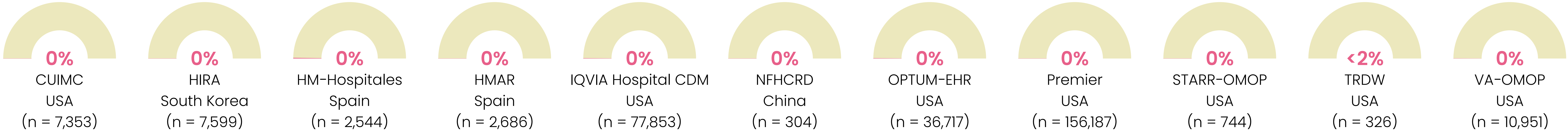

Apixaban use in patients diagnosed or tested + for COVID

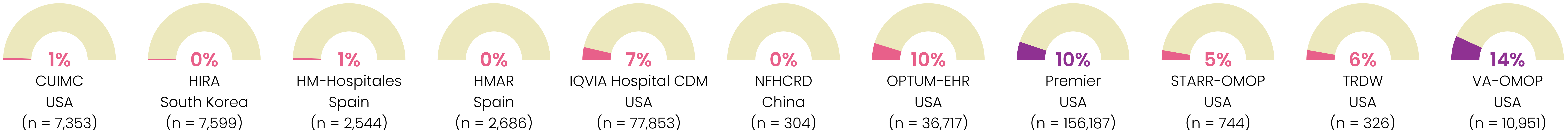

ARBs use in patients diagnosed or tested + for COVID

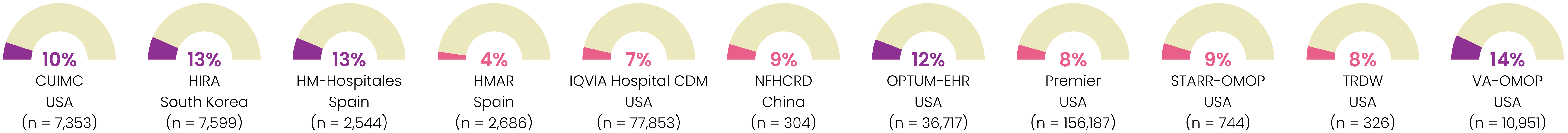

Aspirin use in patients diagnosed or tested + for COVID

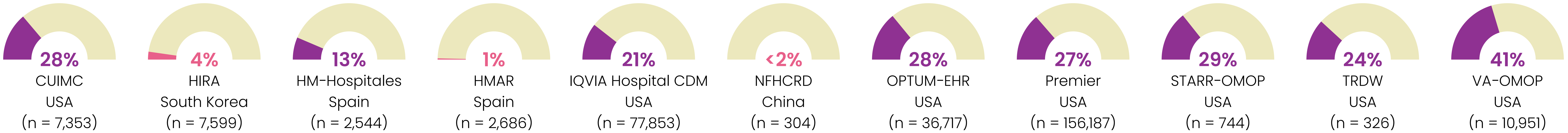

Azithromycin use in patients diagnosed or tested + for COVID

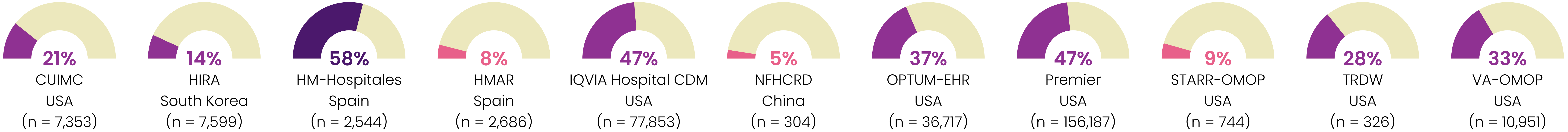

Baricitinib use in patients diagnosed or tested + for COVID

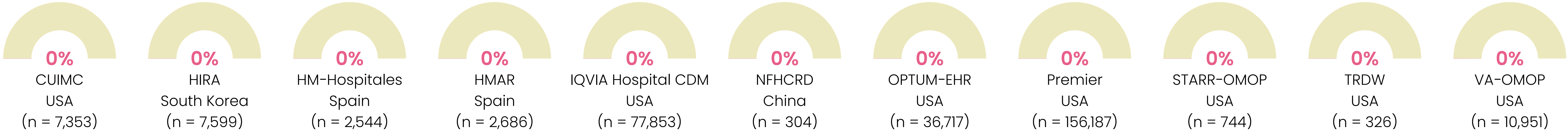

BCG vaccine use in patients diagnosed or tested + for COVID

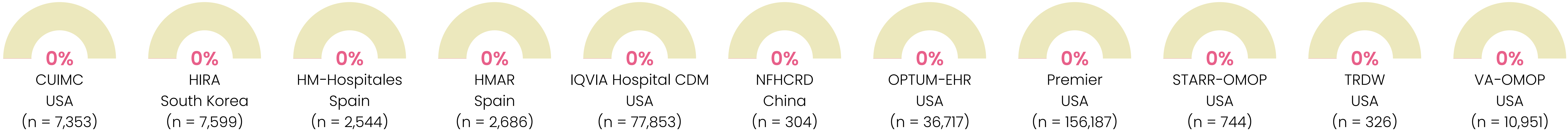

Bemiparin use in patients diagnosed or tested + for COVID

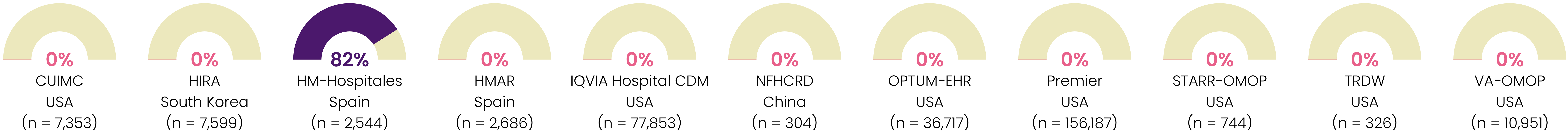

Bevacizumab use in patients diagnosed or tested + for COVID

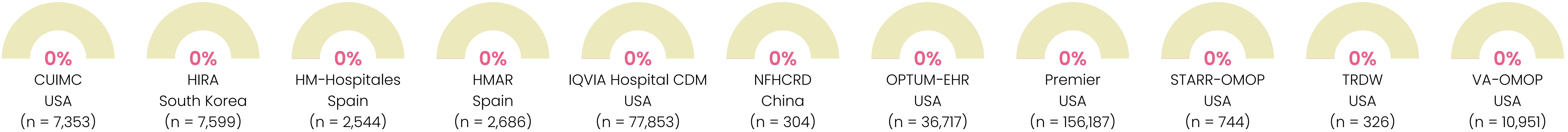

Cangrelor use in patients diagnosed or tested + for COVID

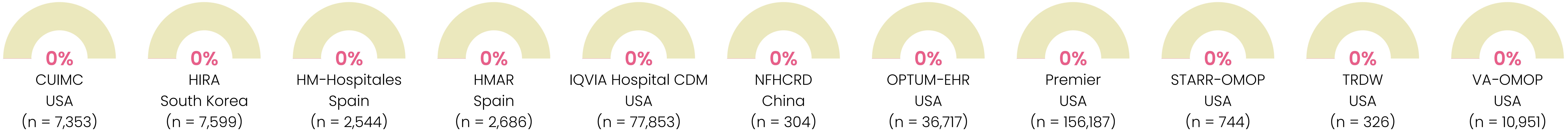

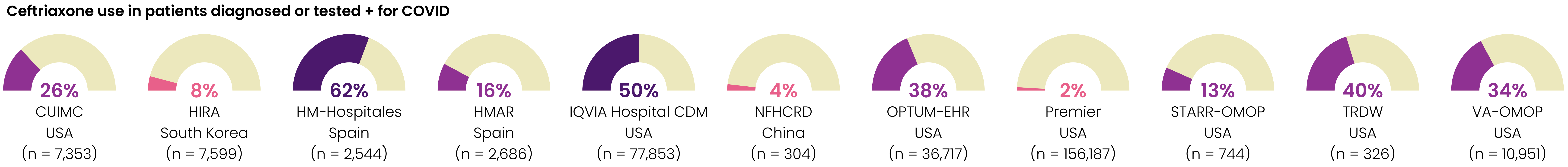
Ceftriaxone use in patients diagnosed or tested + for COVID
26%
CUIMC
USA
(n = 7,353)
8%
HIRA
South Korea
(n = 7,599)
62%
HM-Hospitales
Spain
(n = 2,544)
16%
HMAR
Spain
(n = 2,686)
50%
IQVIA Hospital CDM
USA
(n = 77,853)
4%
NFHCRD
China
(n = 304)
38%
OPTUM-EHR
USA
(n = 36,717)
2%
Premier
USA
(n = 156,187)
13%
STARR-OMOP
USA
(n = 744)
40%
TRDW
USA
(n = 326)
34%
VA-OMOP
USA
(n = 10,951)

Chloroquine use in patients diagnosed or tested + for COVID

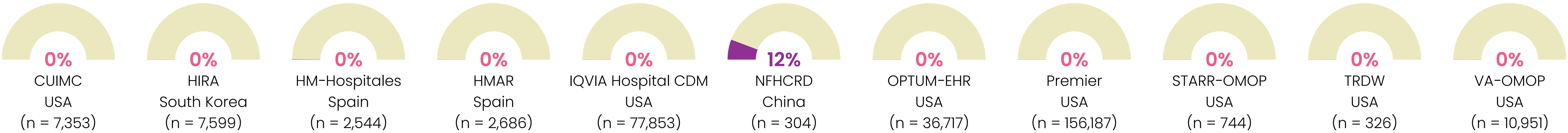

Cilostazol use in patients diagnosed or tested + for COVID

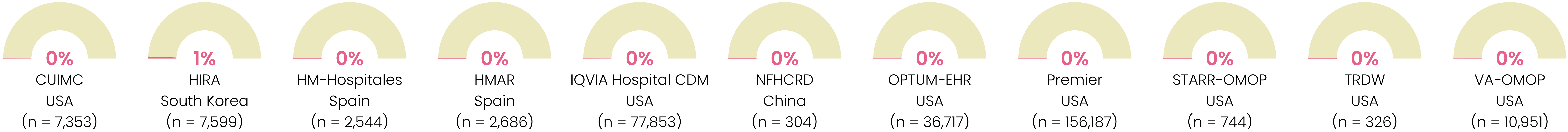

Clopidogrel use in patients diagnosed or tested + for COVID

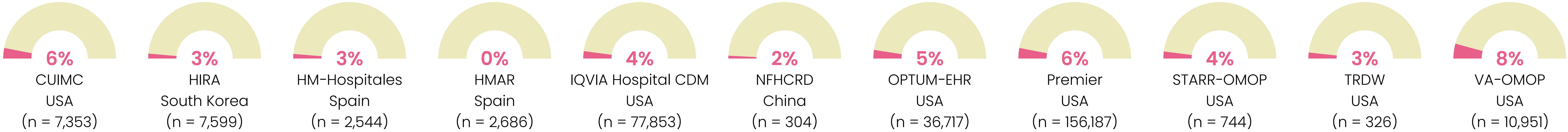

Colchicine use in patients diagnosed or tested + for COVID

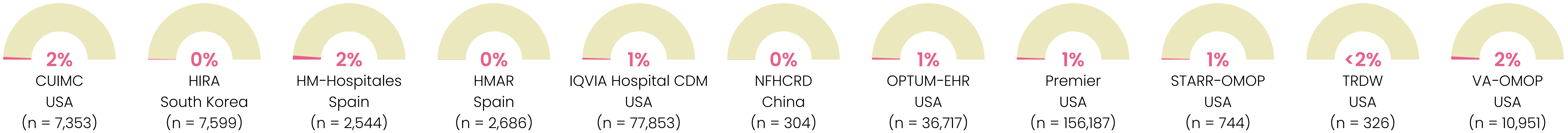

Corticosteroids use in patients diagnosed or tested + for COVID

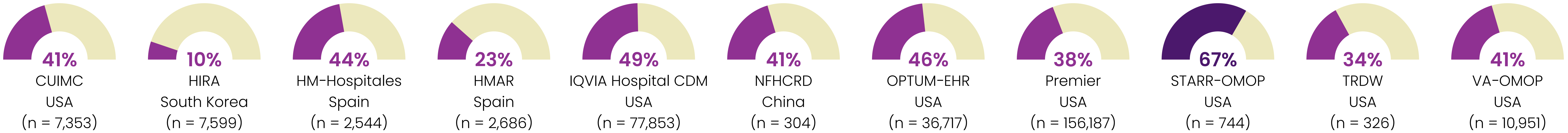

Dabigatran use in patients diagnosed or tested + for COVID

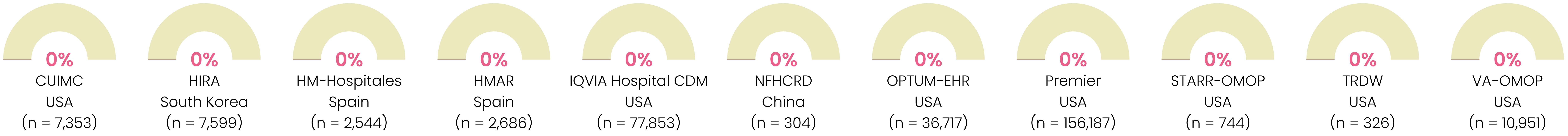

Dabigatran etexilate use in patients diagnosed or tested + for COVID

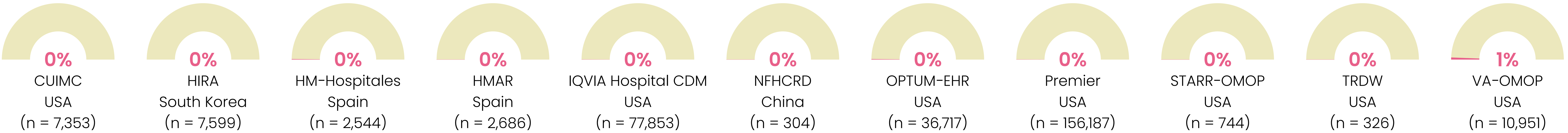

Dalteparin use in patients diagnosed or tested + for COVID

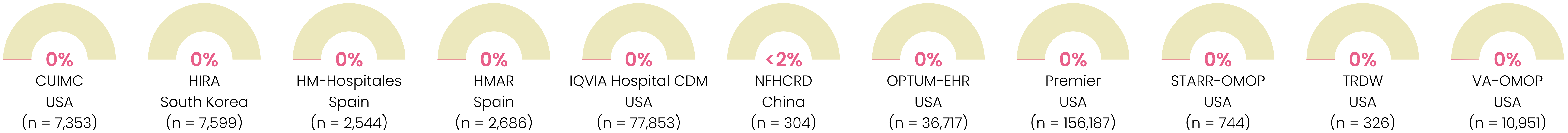

Dapagliflozin use in patients diagnosed or tested + for COVID

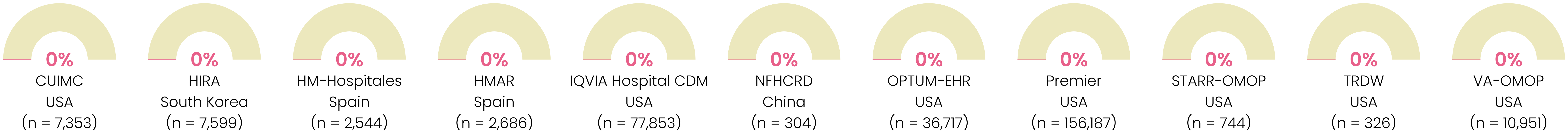

Dexamethasone use in patients diagnosed or tested + for COVID

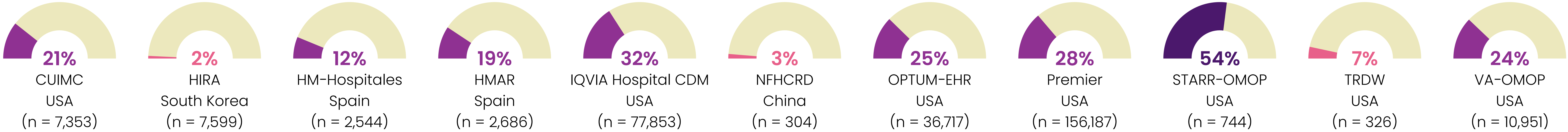

Direct factor Xa inhibitors use in patients diagnosed or tested + for COVID

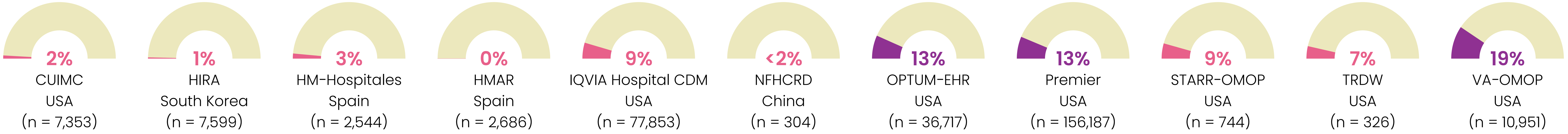

DPP-4 inhibitors use in patients diagnosed or tested + for COVID

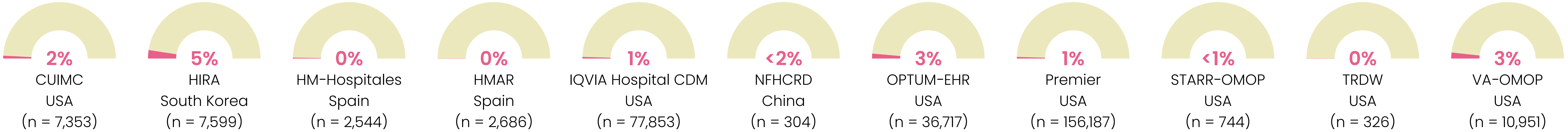

Eculizumab use in patients diagnosed or tested + for COVID

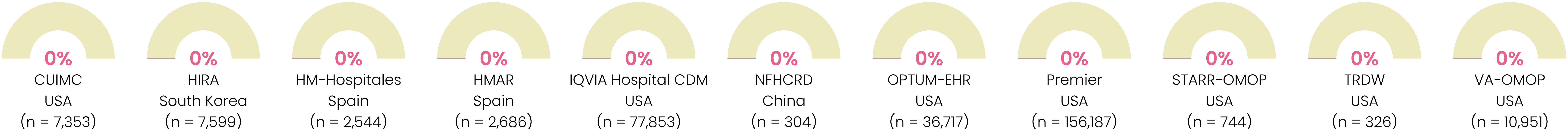

Edoxaban use in patients diagnosed or tested + for COVID

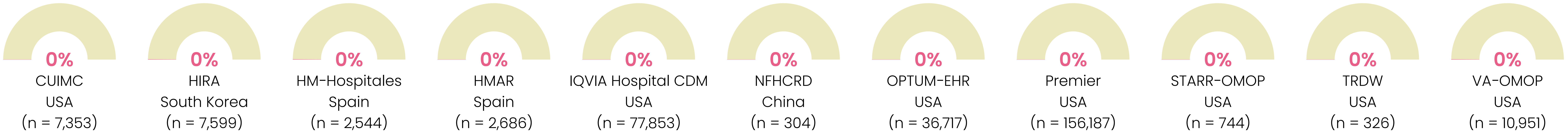

Enoxaparin use in patients diagnosed or tested + for COVID

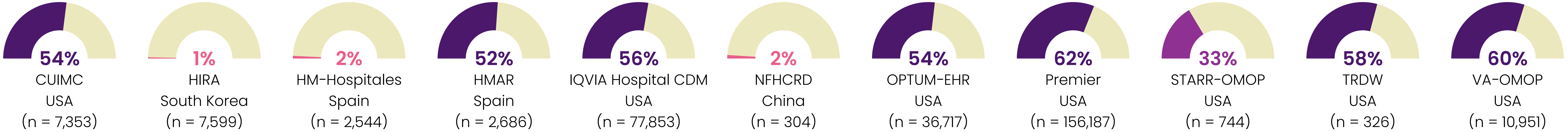

Etanercept use in patients diagnosed or tested + for COVID

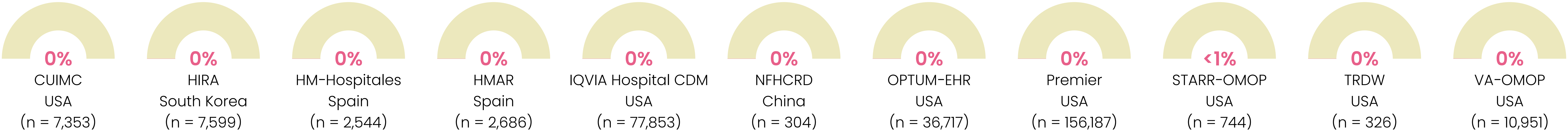

Famotidine use in patients diagnosed or tested + for COVID

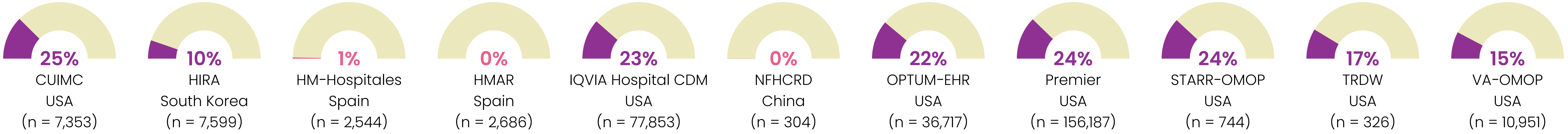

Fingolimod use in patients diagnosed or tested + for COVID

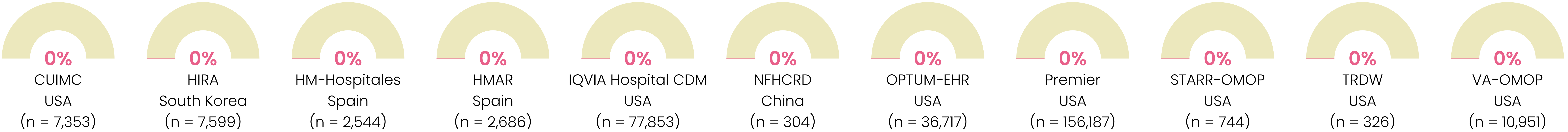

Fluoroquinolones use in patients diagnosed or tested + for COVID

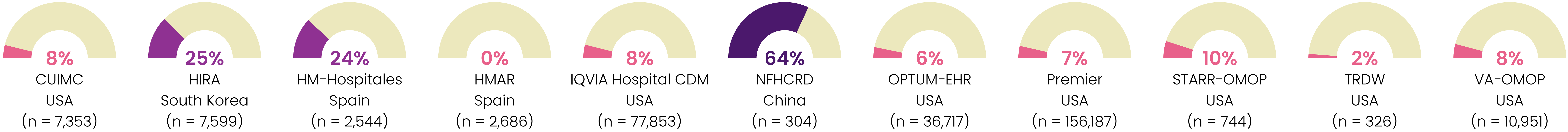

GLP1 inhibitors use in patients diagnosed or tested + for COVID

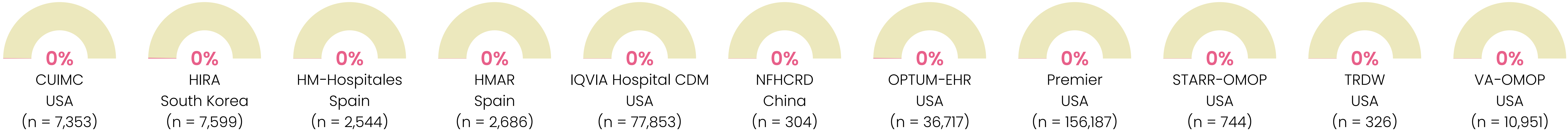

H2 receptor antagonist use in patients diagnosed or tested + for COVID

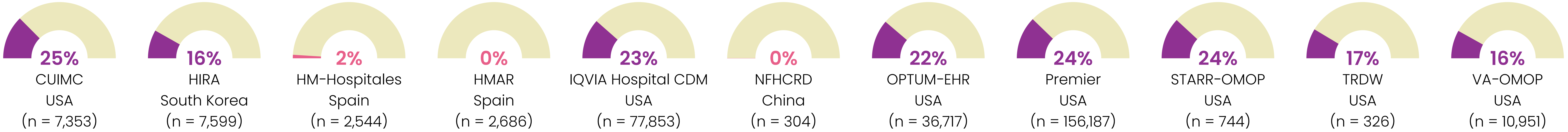

Heparin use in patients diagnosed or tested + for COVID

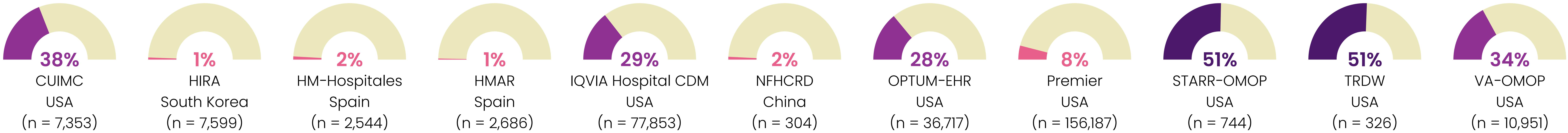

Hydroxychloroquine use in patients diagnosed or tested + for COVID

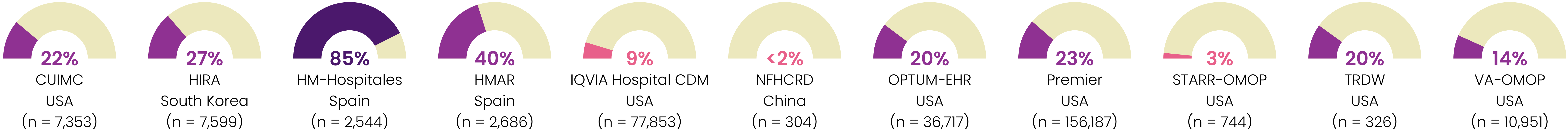

Ibrutinib use in patients diagnosed or tested + for COVID

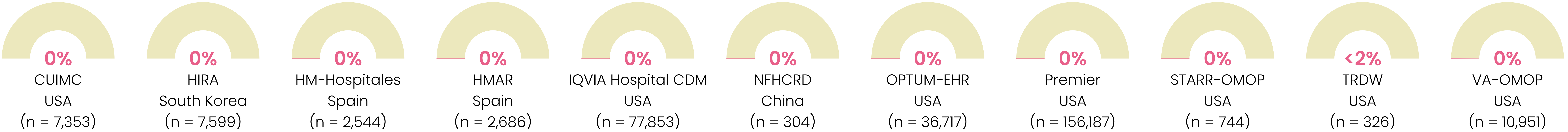

Immunoglobulins use in patients diagnosed or tested + for COVID

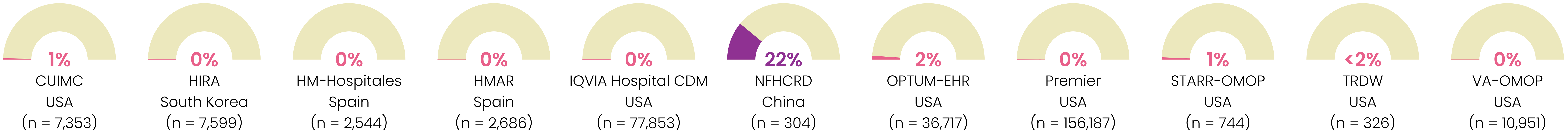

Infliximab use in patients diagnosed or tested + for COVID

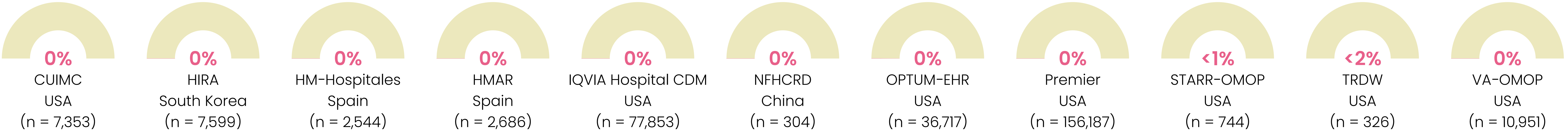

Interleukin inhibitors use in patients diagnosed or tested + for COVID

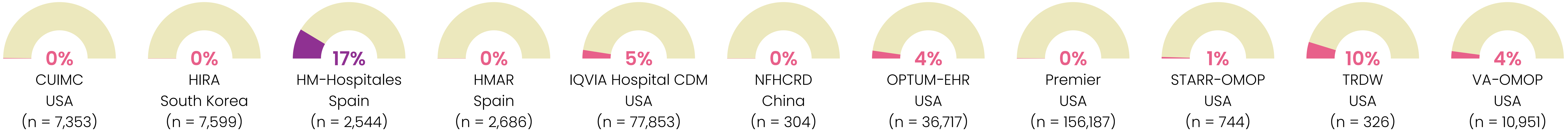

Itraconazole use in patients diagnosed or tested + for COVID

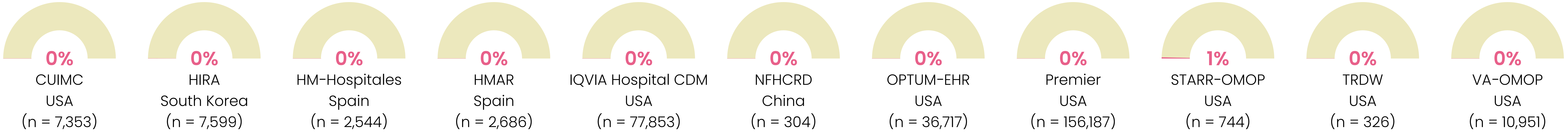

Ivermectin use in patients diagnosed or tested + for COVID

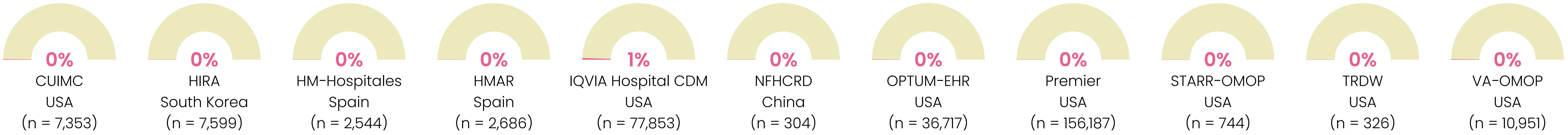

Linagliptin use in patients diagnosed or tested + for COVID

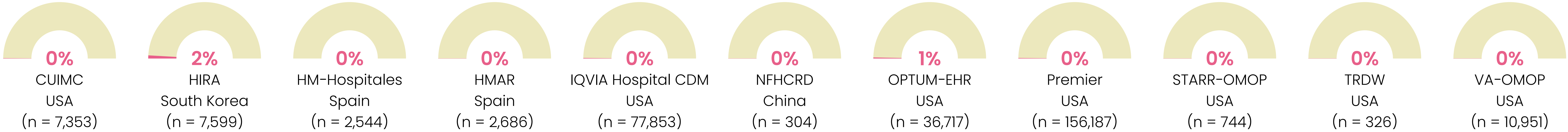

Lopinavir use in patients diagnosed or tested + for COVID

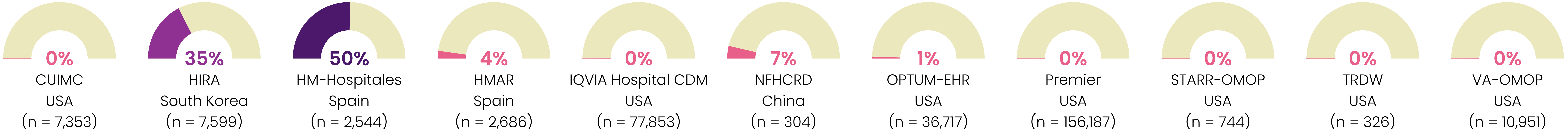

Losartan use in patients diagnosed or tested + for COVID

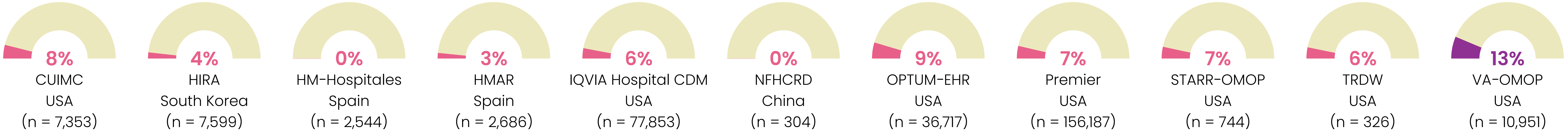

Metformin use in patients diagnosed or tested + for COVID

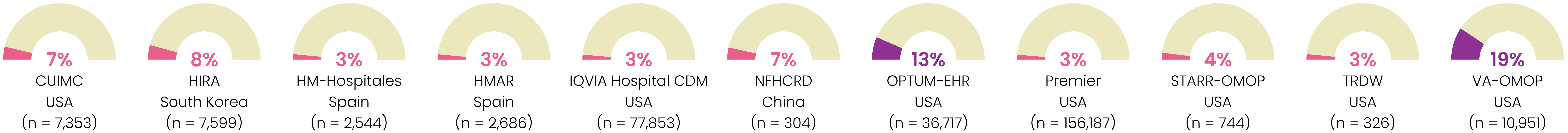

Nitazoxanide use in patients diagnosed or tested + for COVID

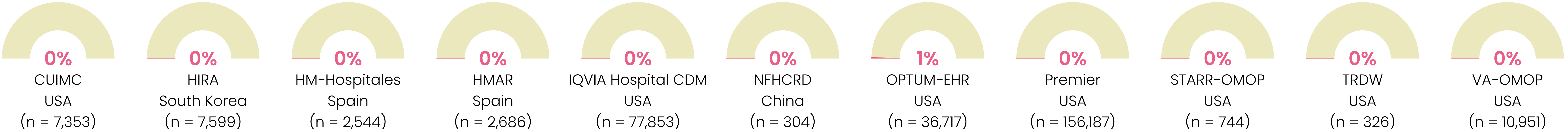

Nitric oxide use in patients diagnosed or tested + for COVID

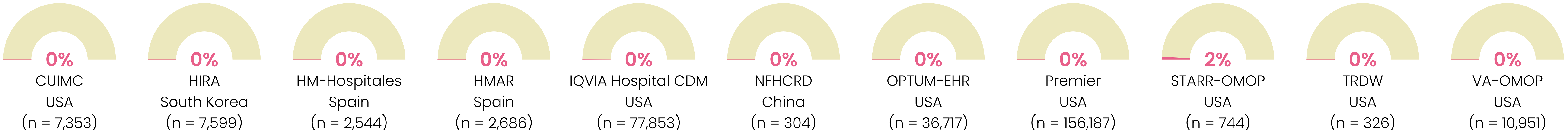

Oseltamivir use in patients diagnosed or tested + for COVID

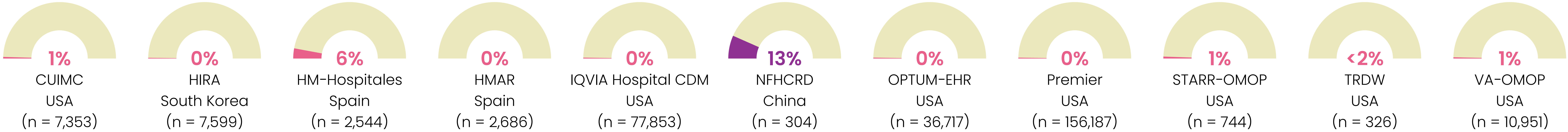

Peginterferon alfa-2a use in patients diagnosed or tested + for COVID

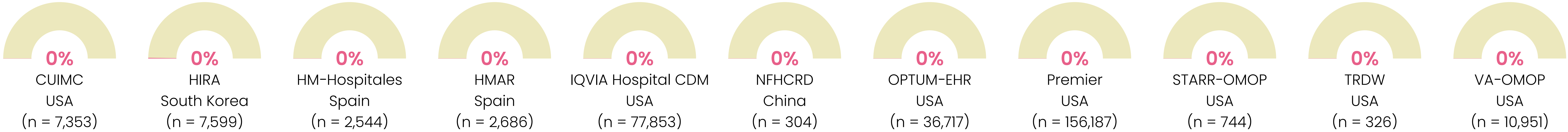

Pirfenidone use in patients diagnosed or tested + for COVID

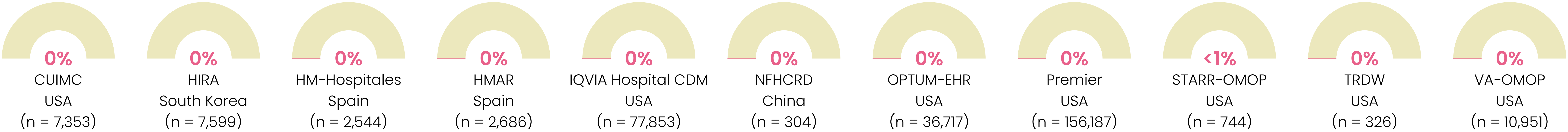

Prasugrel use in patients diagnosed or tested + for COVID

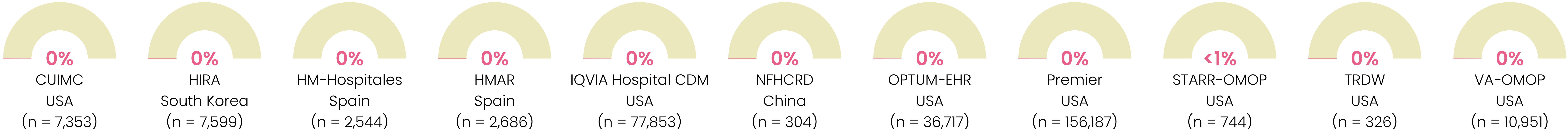

Prazosin use in patients diagnosed or tested + for COVID

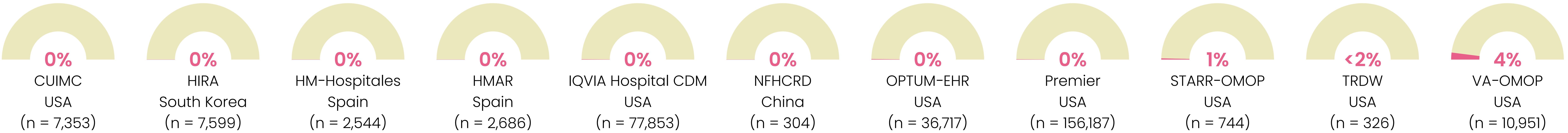

Remdesivir use in patients diagnosed or tested + for COVID

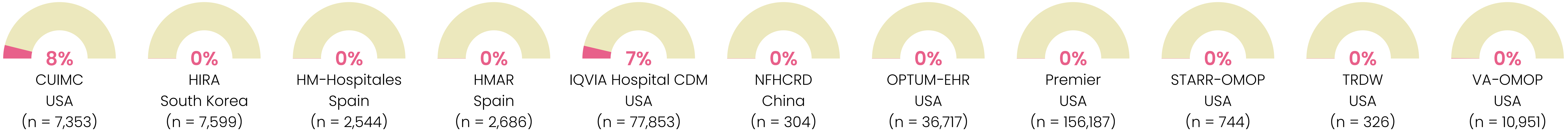

Ribavirin use in patients diagnosed or tested + for COVID

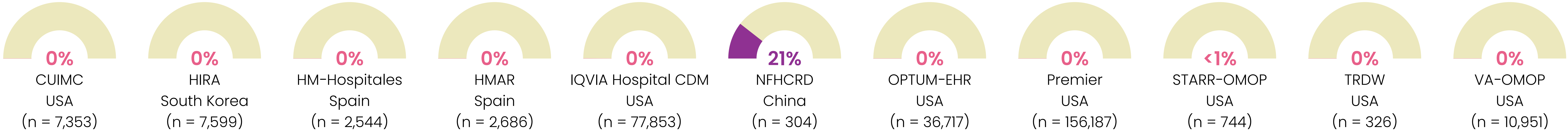

Ritonavir use in patients diagnosed or tested + for COVID

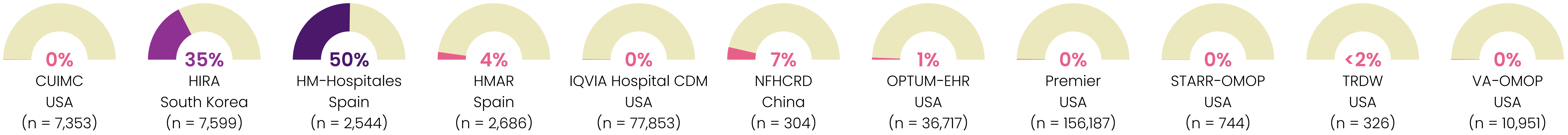

Rivaroxaban use in patients diagnosed or tested + for COVID

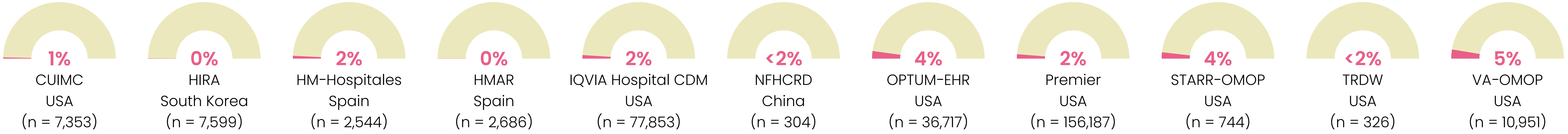

Ruxolitinib use in patients diagnosed or tested + for COVID

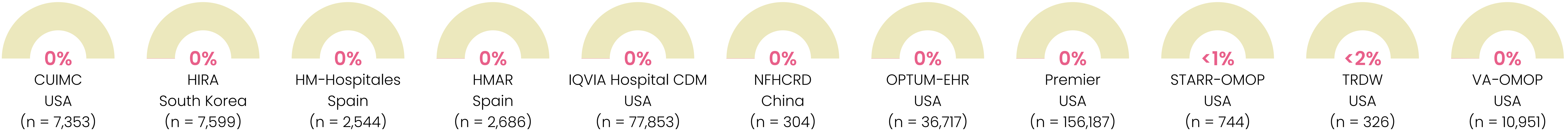

Sargramostim use in patients diagnosed or tested + for COVID

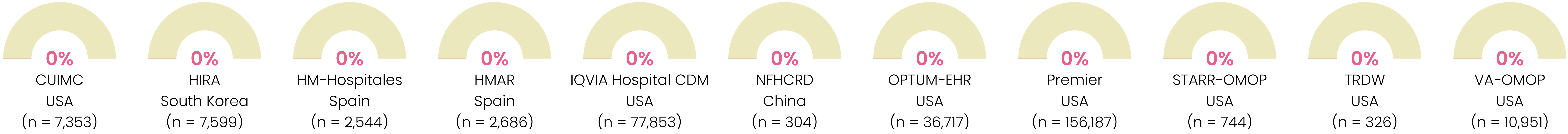

Sarilumab use in patients diagnosed or tested + for COVID

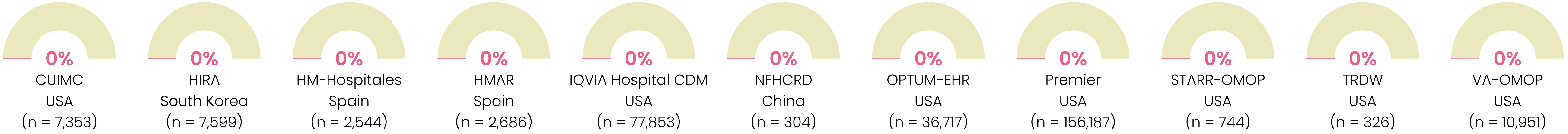

SGLT2 inhibitors use in patients diagnosed or tested + for COVID

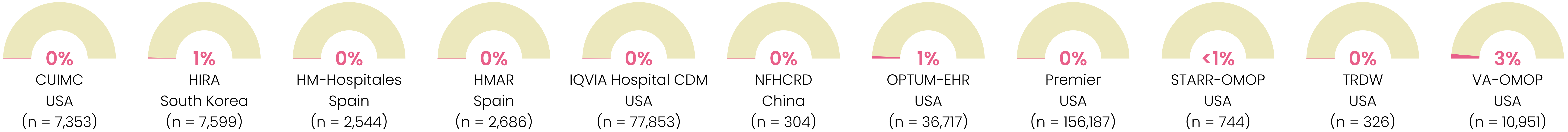

Siltuximab use in patients diagnosed or tested + for COVID

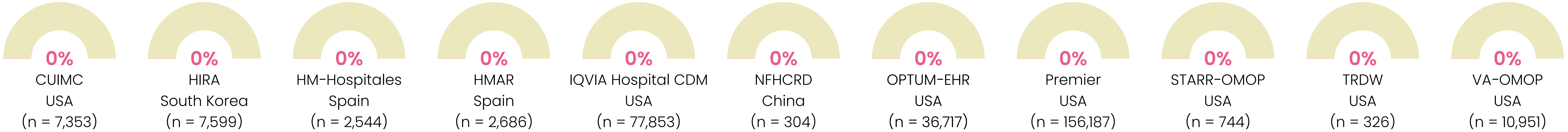

Sitagliptin use in patients diagnosed or tested + for COVID

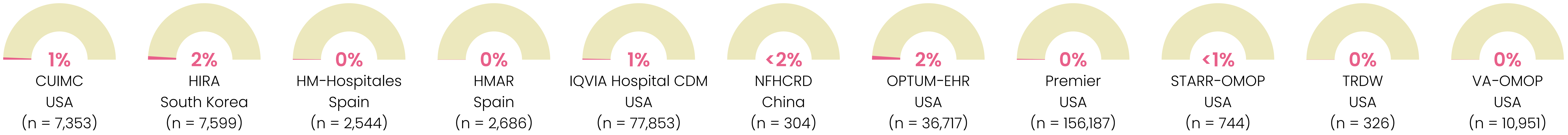

Statins use in patients diagnosed or tested + for COVID

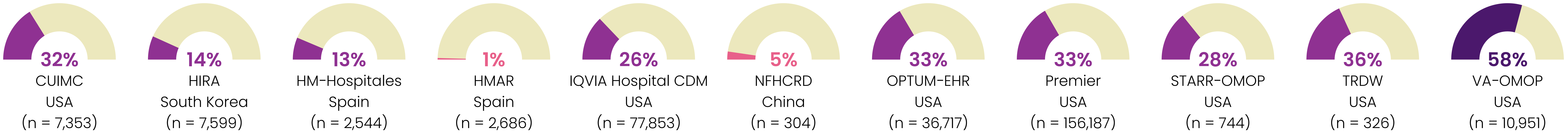

Ticagrelor use in patients diagnosed or tested + for COVID

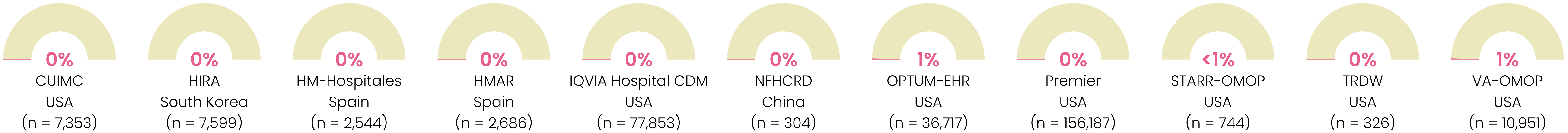

Ticlopidine use in patients diagnosed or tested + for COVID

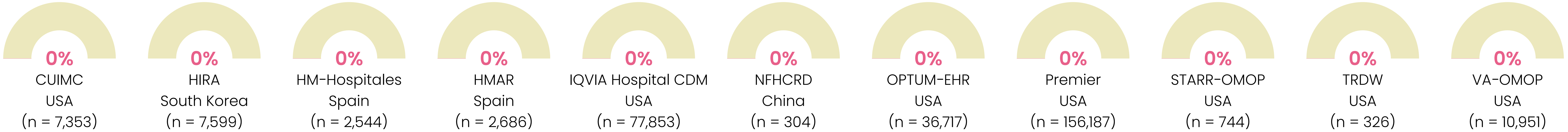

TNF inhibitors use in patients diagnosed or tested + for COVID

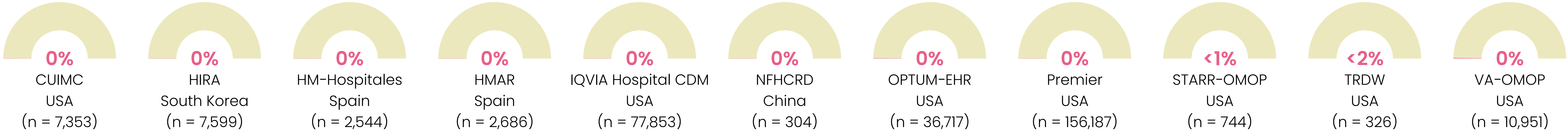

Tocilizumab use in patients diagnosed or tested + for COVID

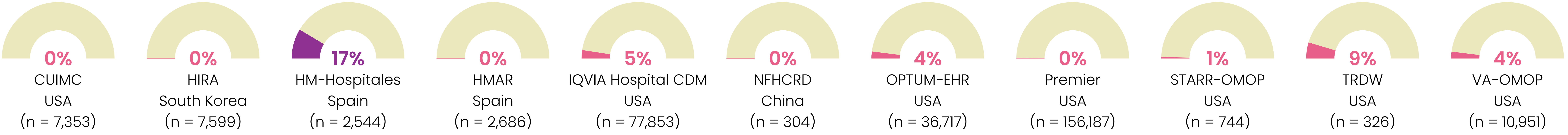

Tofacitinib use in patients diagnosed or tested + for COVID

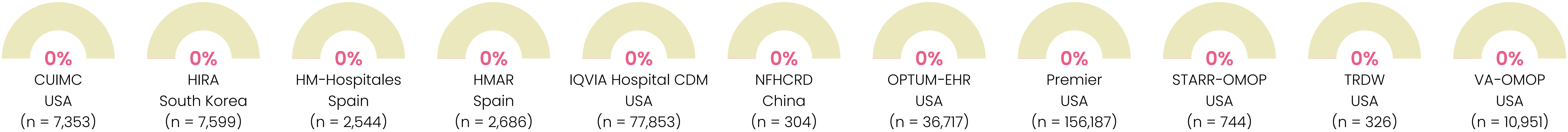

Tranexamic acid use in patients diagnosed or tested + for COVID

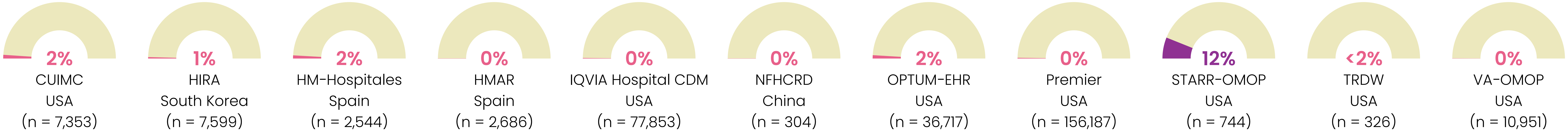

Triflusal use in patients diagnosed or tested + for COVID

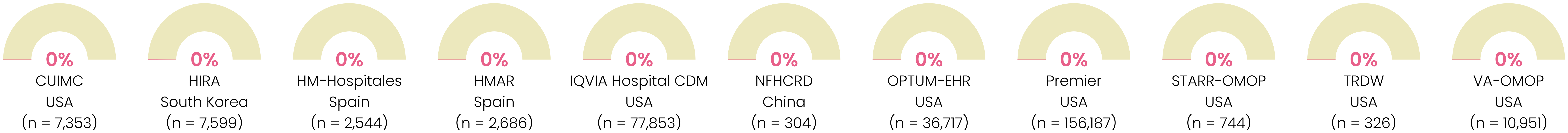

Umifenovir use in patients diagnosed or tested + for COVID

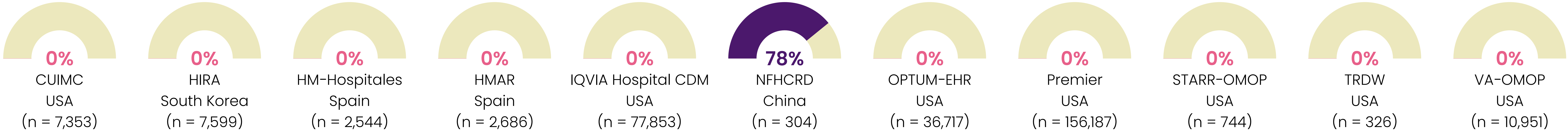

Ustekinumab use in patients diagnosed or tested + for COVID

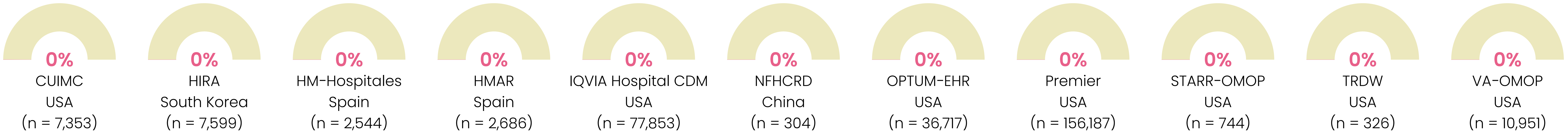

Vitamin C use in patients diagnosed or tested + for COVID

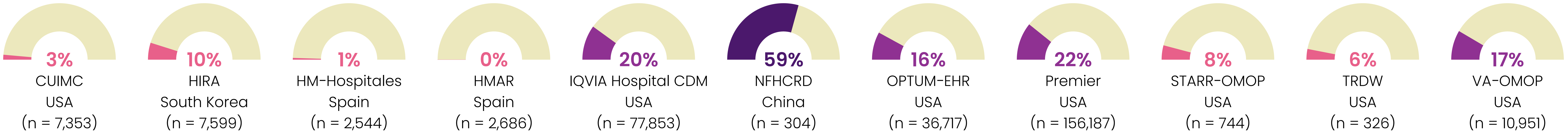

Vitamin D use in patients diagnosed or tested + for COVID

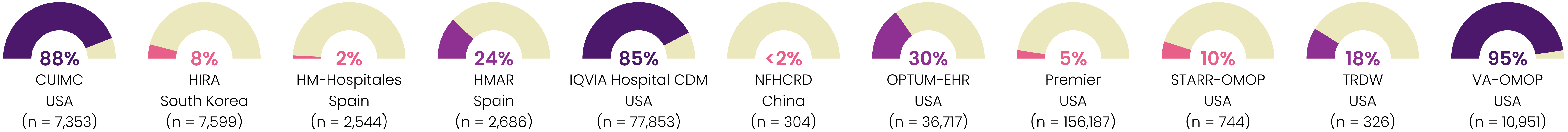

Warfarin use in patients diagnosed or tested + for COVID

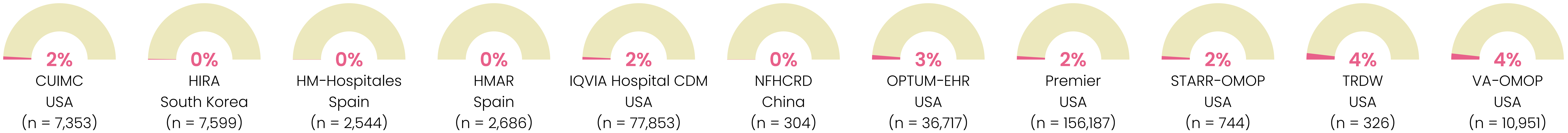

Supplement: Supplementary file 2 — Web appendix: Supplementary figure 4 [file praa062143.wf4.pdf]
