# Supplementary material for: Use of repurposed and adjuvant drugs in hospital patients with covid-19: multinational network cohort study
Source: BMJ. 2021 May 11;373:n1038. doi: 10.1136/bmj.n1038 (PMC8111167; doi:10.1136/bmj.n1038)

ACE inhibitors use in patients diagnosed or tested + for COVID

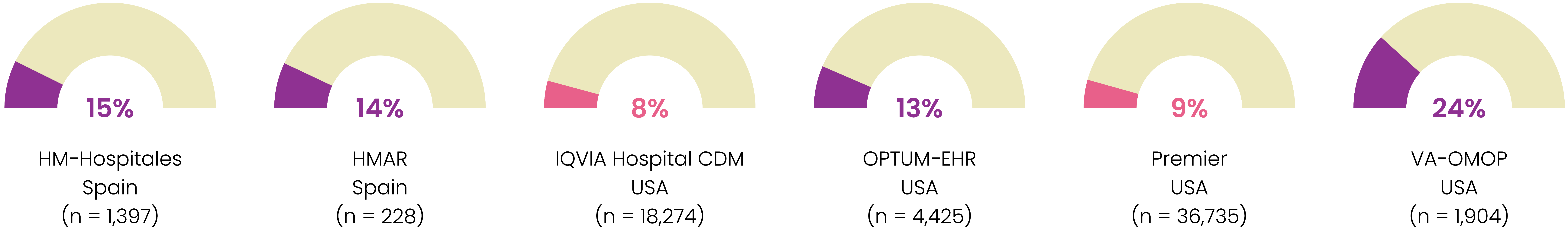

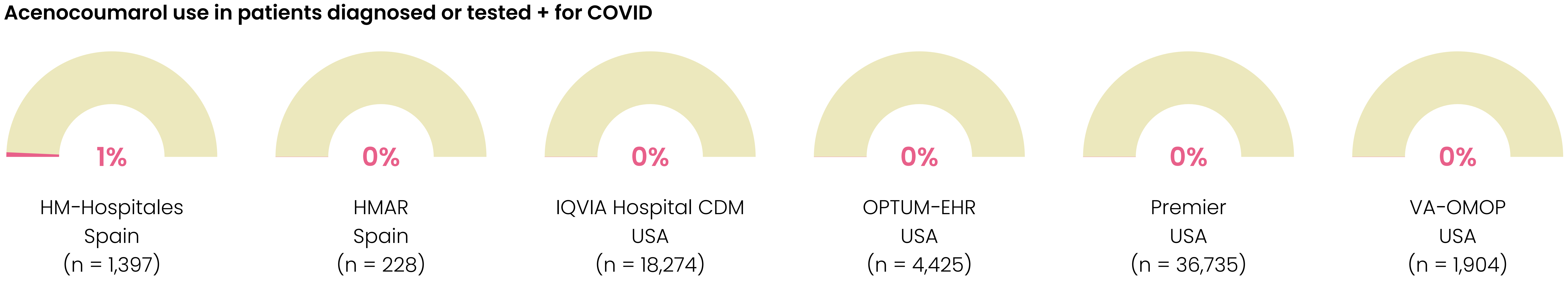
Acenocoumarol use in patients diagnosed or tested + for COVID
1%
HM-Hospitales
Spain
(n = 1,397)
0%
HMAR
Spain
(n = 228)
0%
IQVIA Hospital CDM
USA
(n = 18,274)
0%
OPTUM-EHR
USA
(n = 4,425)
0%
Premier
USA
(n = 36,735)
0%
VA-OMOP
USA
(n = 1,904)

Adalimumab use in patients diagnosed or tested + for COVID

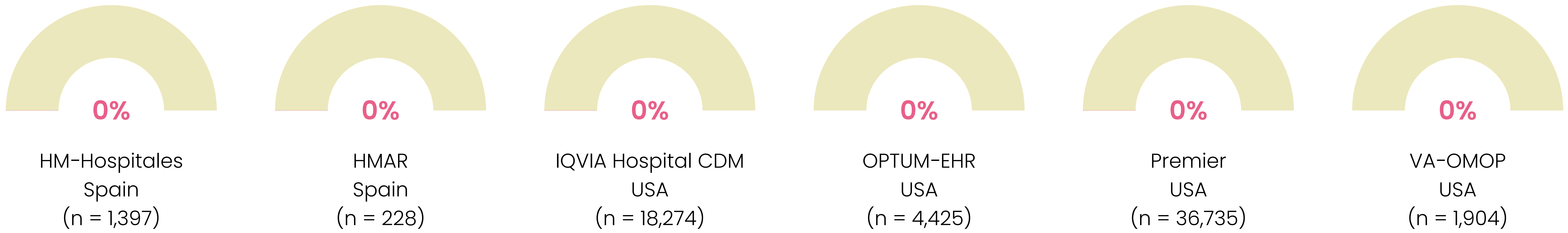

Alpha-1 blockers use in patients diagnosed or tested + for COVID

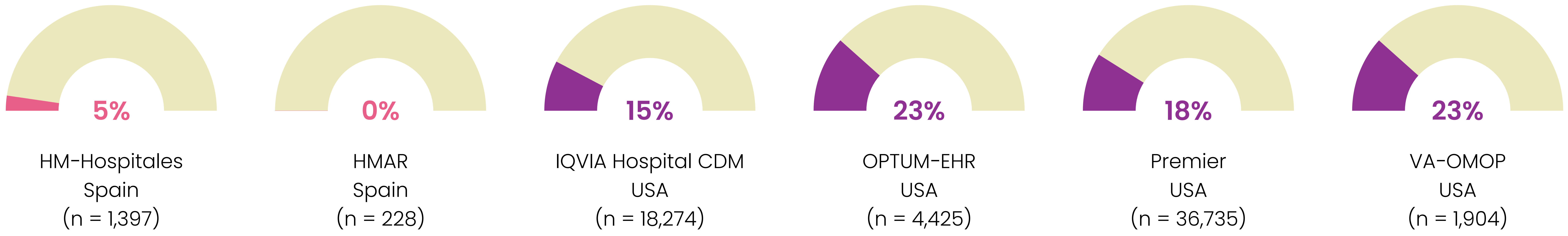

Amoxicillin use in patients diagnosed or tested + for COVID

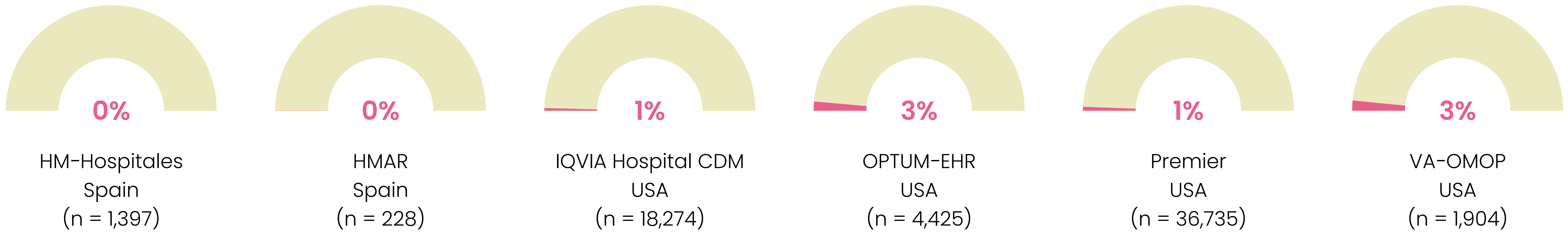

Anakinra use in patients diagnosed or tested + for COVID

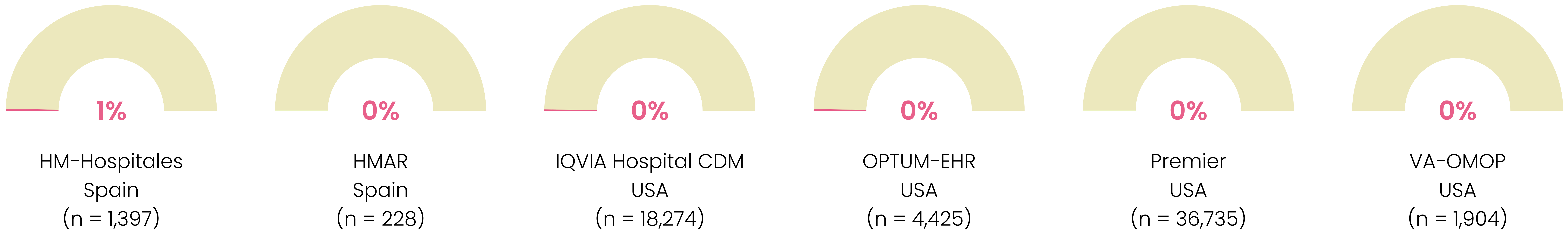

Apixaban use in patients diagnosed or tested + for COVID

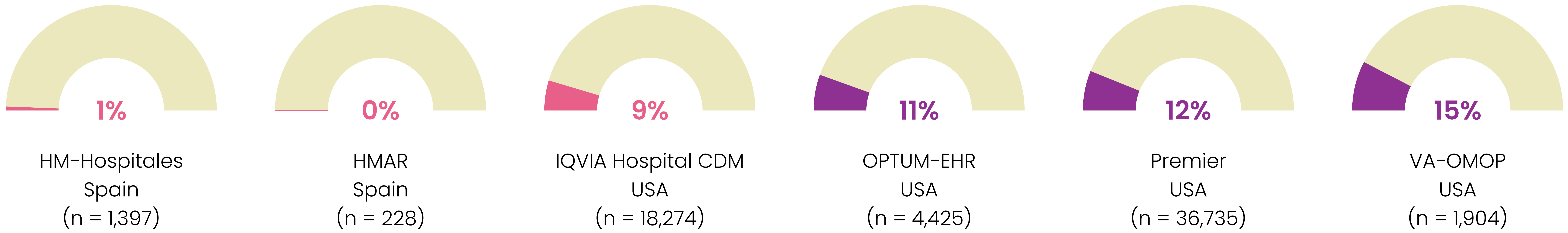

ARBs use in patients diagnosed or tested + for COVID

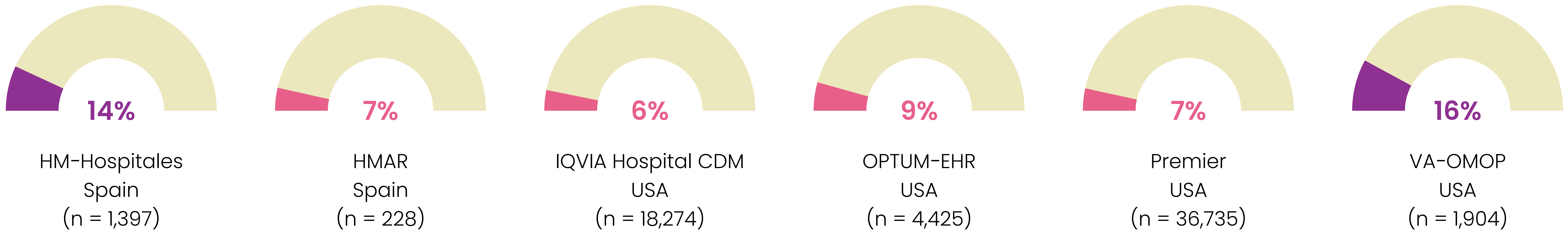

Aspirin use in patients diagnosed or tested + for COVID

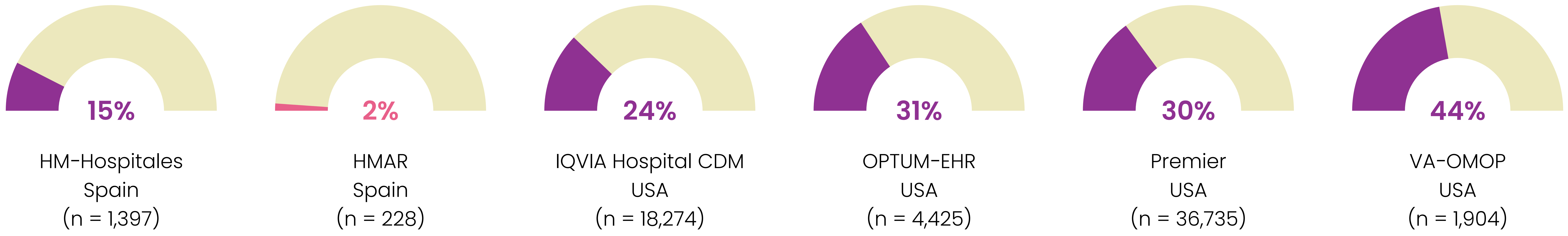

Azithromycin use in patients diagnosed or tested + for COVID

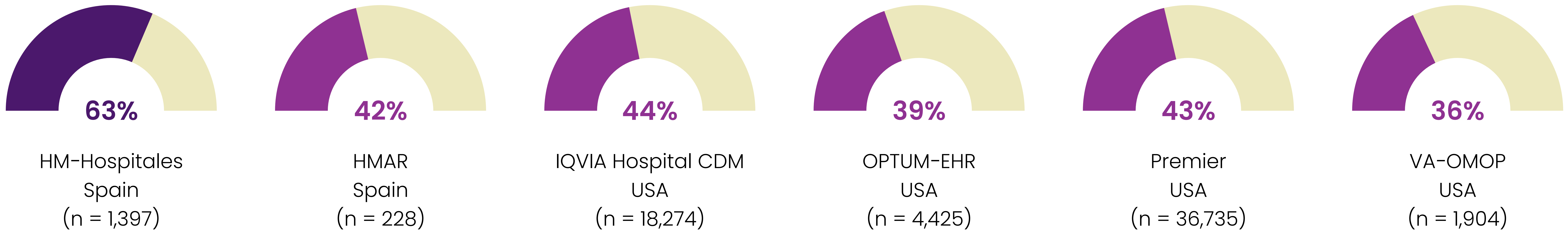

Baricitinib use in patients diagnosed or tested + for COVID

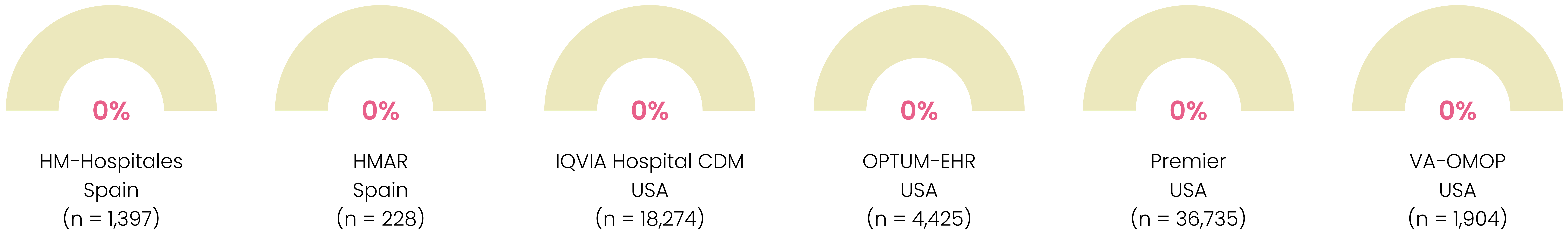

Bemiparin use in patients diagnosed or tested + for COVID

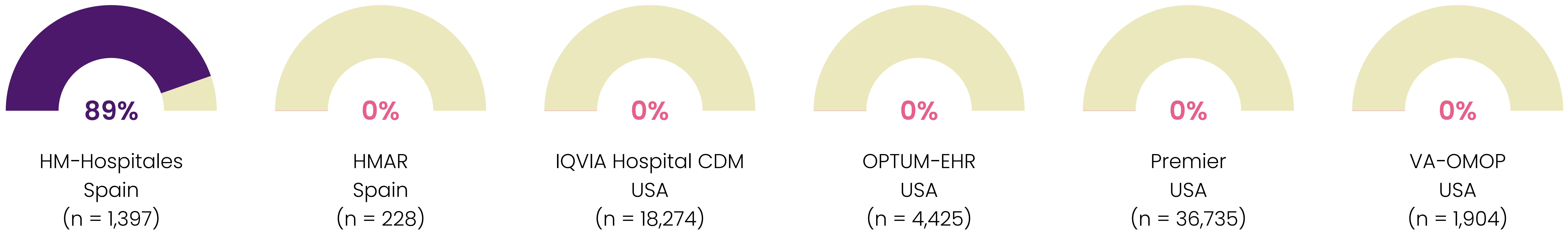

Bevacizumab use in patients diagnosed or tested + for COVID

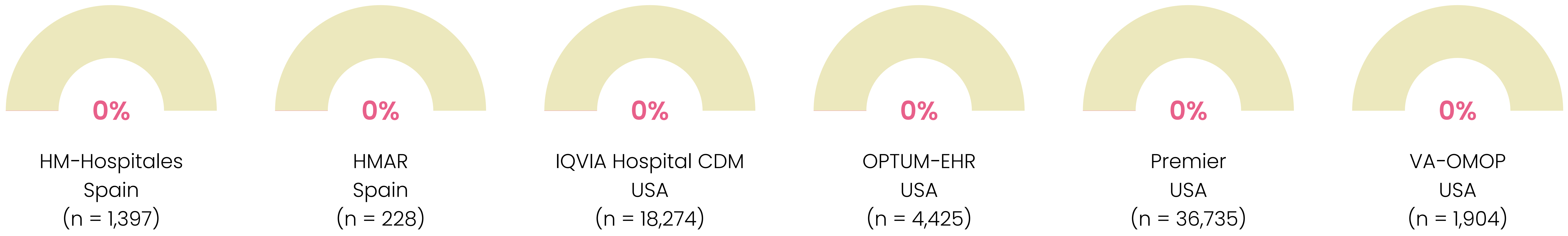

Cangrelor use in patients diagnosed or tested + for COVID

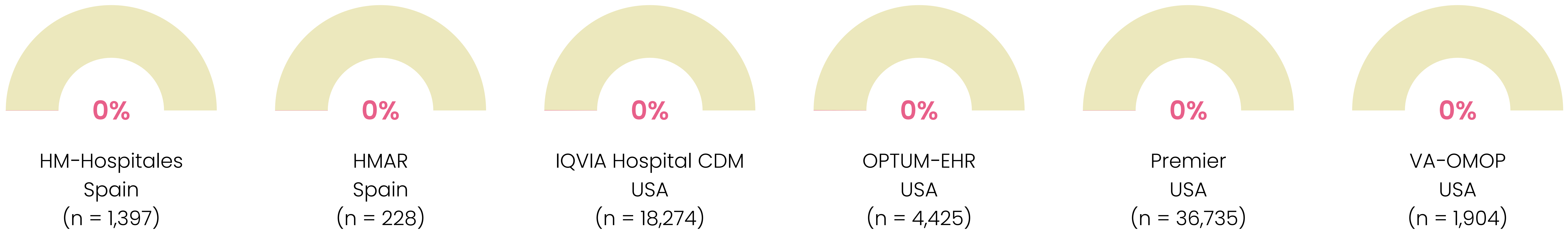

Ceftriaxone use in patients diagnosed or tested + for COVID

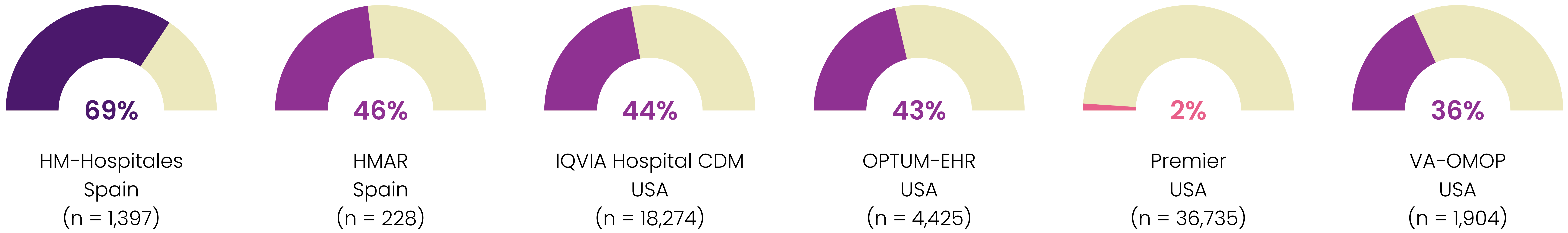

Chloroquine use in patients diagnosed or tested + for COVID

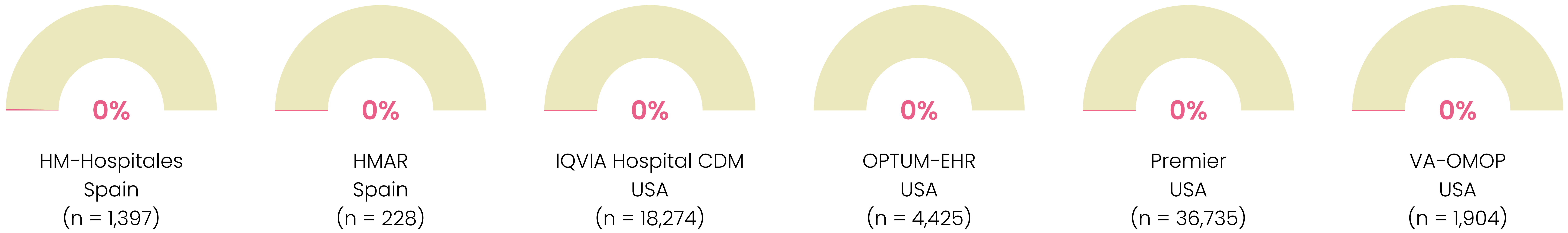

Cilostazol use in patients diagnosed or tested + for COVID

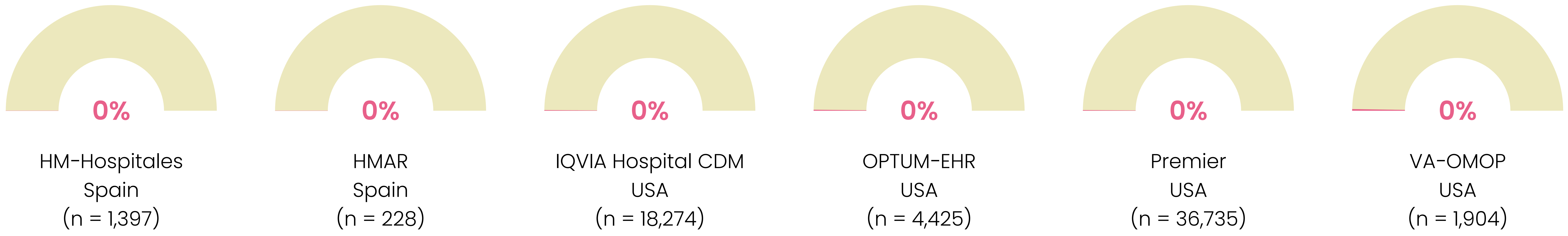

Clopidogrel use in patients diagnosed or tested + for COVID

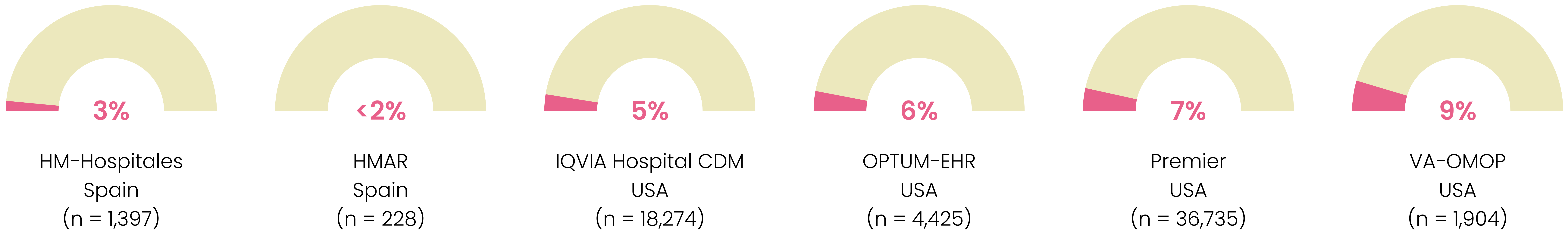

Colchicine use in patients diagnosed or tested + for COVID

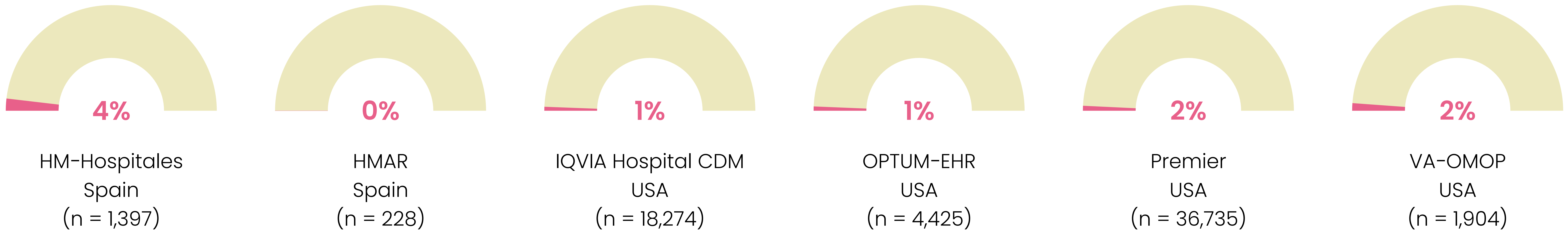

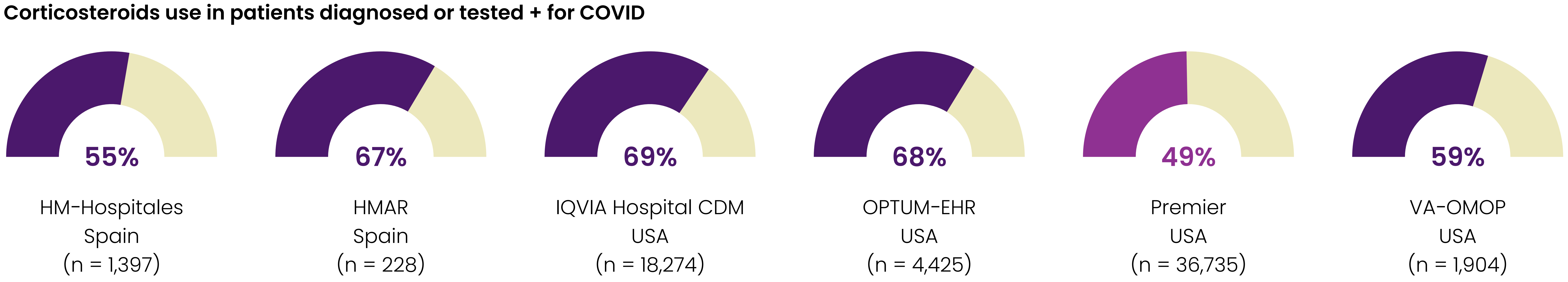
Corticosteroids use in patients diagnosed or tested + for COVID
55%
HM-Hospitales
Spain
(n = 1,397)
67%
HMAR
Spain
(n = 228)
69%
IQVIA Hospital CDM
USA
(n = 18,274)
68%
OPTUM-EHR
USA
(n = 4,425)
49%
Premier
USA
(n = 36,735)
59%
VA-OMOP
USA
(n = 1,904)

Dabigatran use in patients diagnosed or tested + for COVID

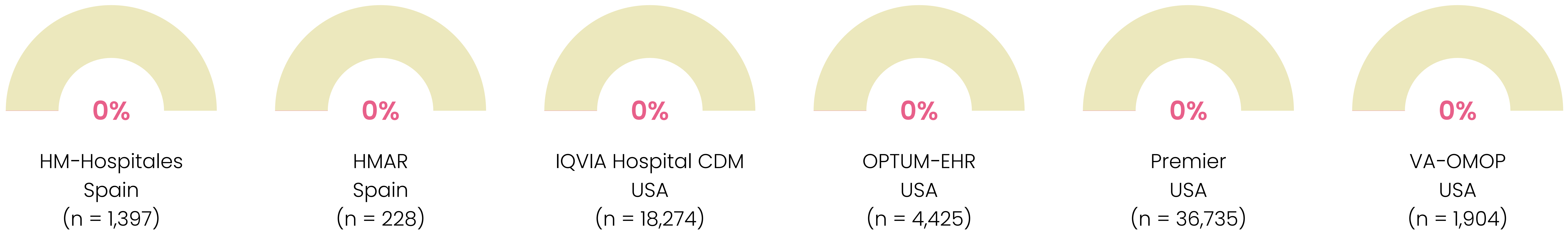

Dabigatran etexilate use in patients diagnosed or tested + for COVID

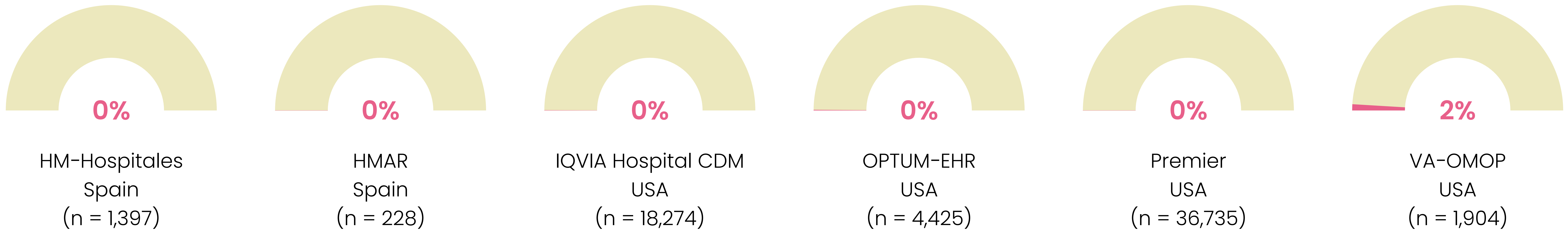

Dalteparin use in patients diagnosed or tested + for COVID

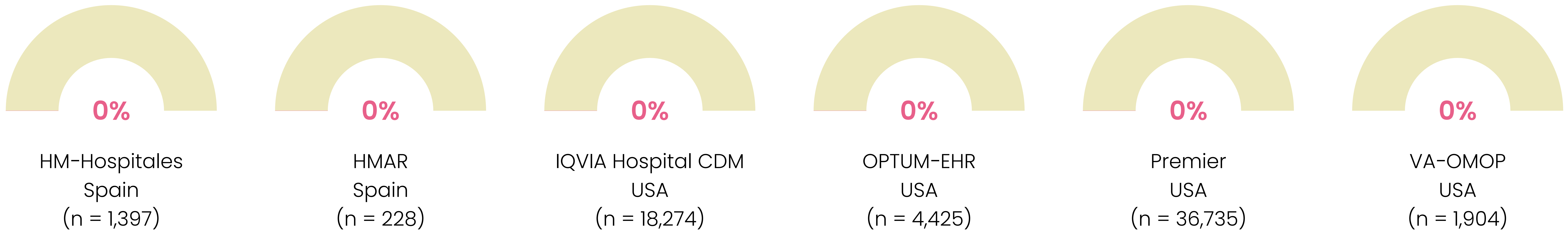

Dapagliflozin use in patients diagnosed or tested + for COVID

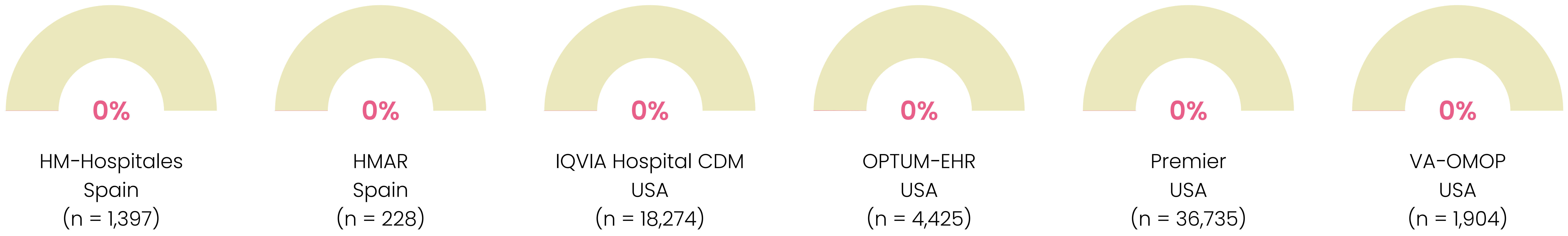

Dexamethasone use in patients diagnosed or tested + for COVID

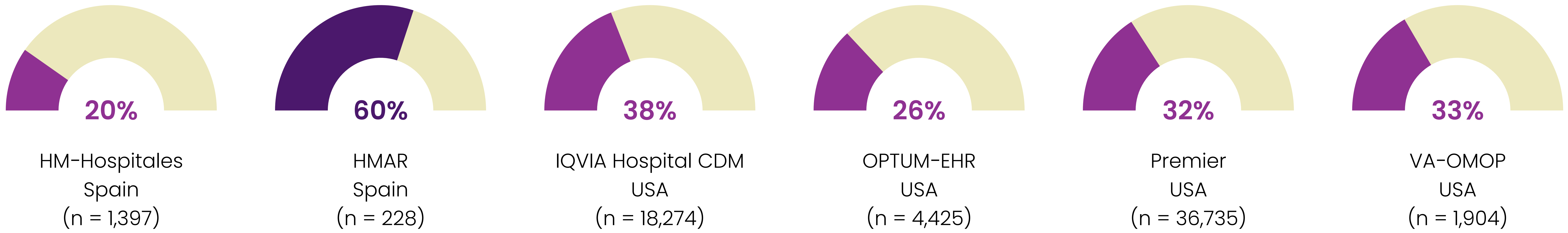

Direct factor Xa inhibitors use in patients diagnosed or tested + for COVID

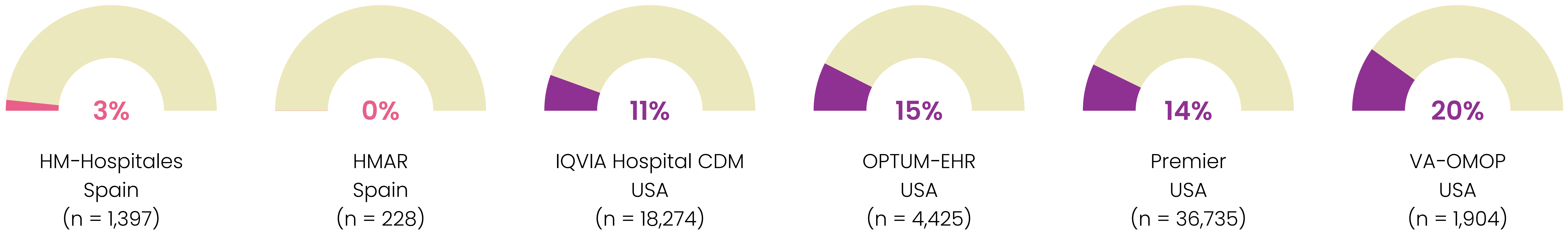

DPP-4 inhibitors use in patients diagnosed or tested + for COVID

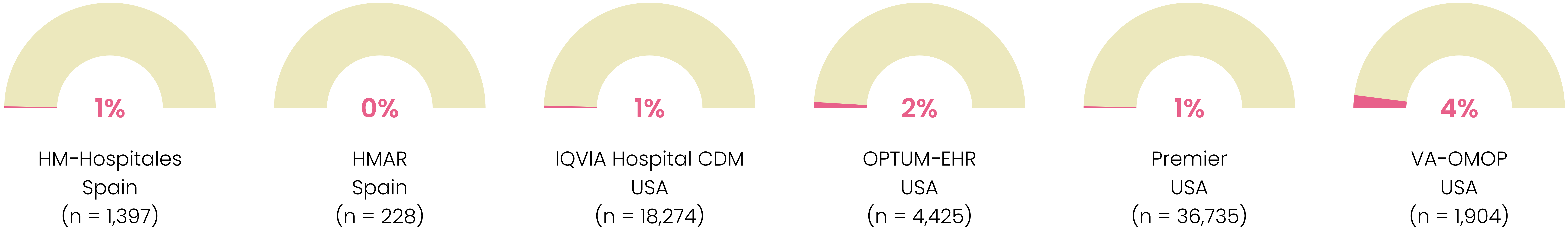

Edoxaban use in patients diagnosed or tested + for COVID

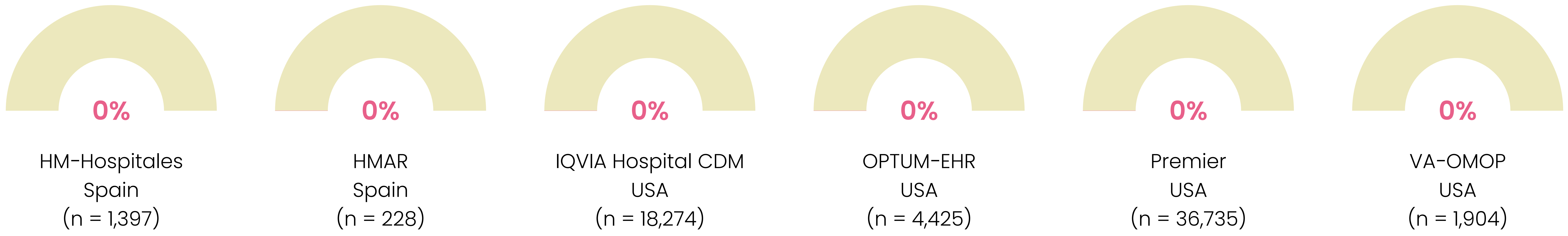

Enoxaparin use in patients diagnosed or tested + for COVID

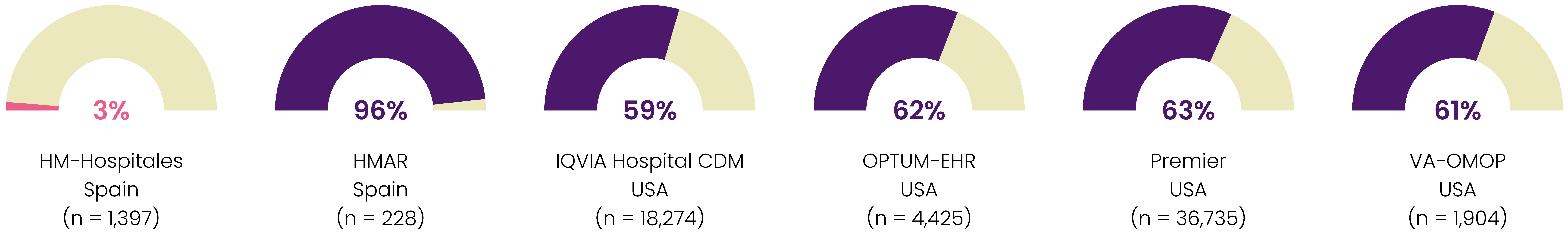

Etanercept use in patients diagnosed or tested + for COVID

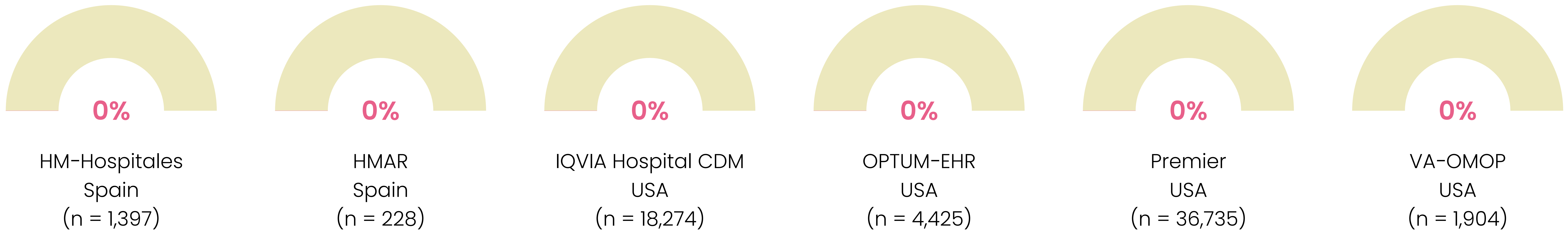

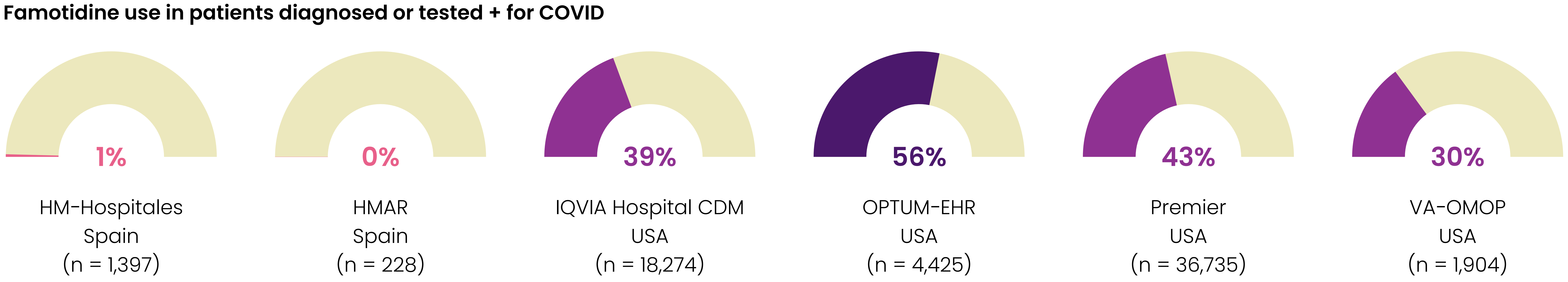
Famotidine use in patients diagnosed or tested + for COVID
1%
HM-Hospitales
Spain
(n = 1,397)
0%
HMAR
Spain
(n = 228)
39%
IQVIA Hospital CDM
USA
(n = 18,274)
56%
OPTUM-EHR
USA
(n = 4,425)
43%
Premier
USA
(n = 36,735)
30%
VA-OMOP
USA
(n = 1,904)

Fingolimod use in patients diagnosed or tested + for COVID

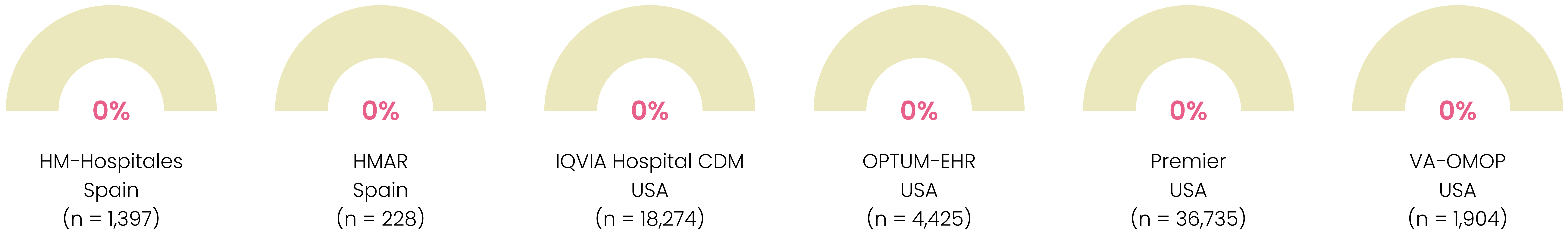

Fluoroquinolones use in patients diagnosed or tested + for COVID

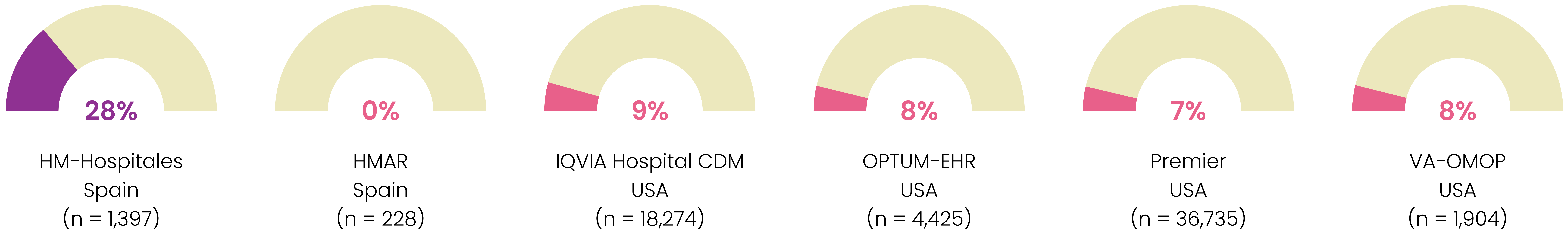

GLP1 inhibitors use in patients diagnosed or tested + for COVID

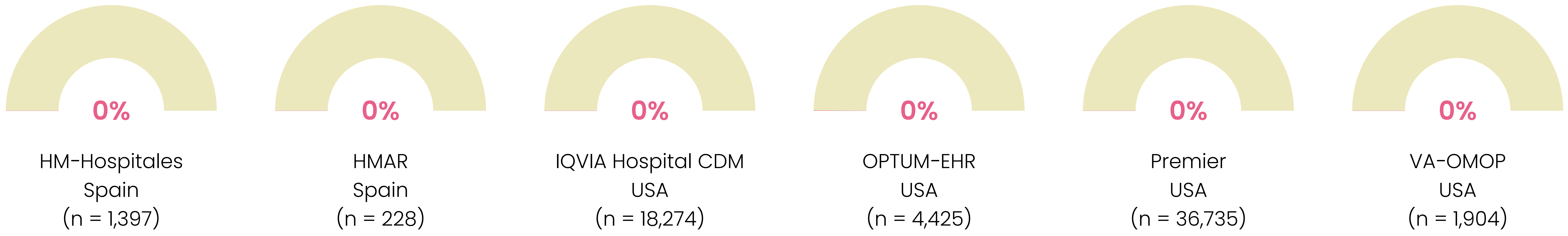

H2 receptor antagonist use in patients diagnosed or tested + for COVID

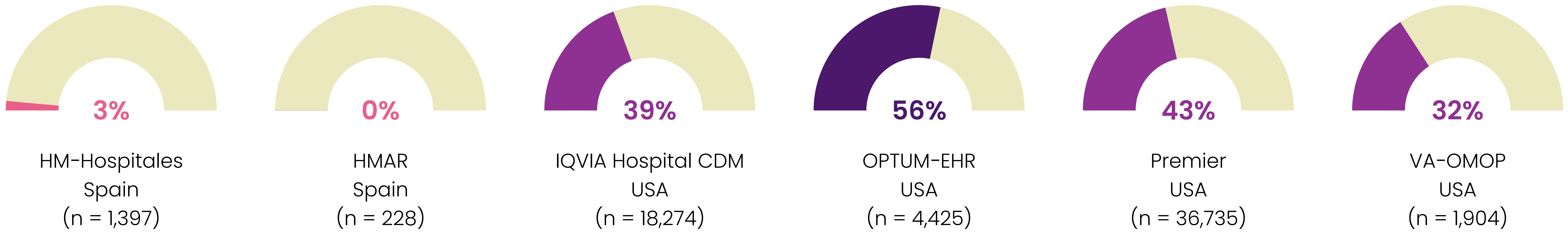

Heparin use in patients diagnosed or tested + for COVID

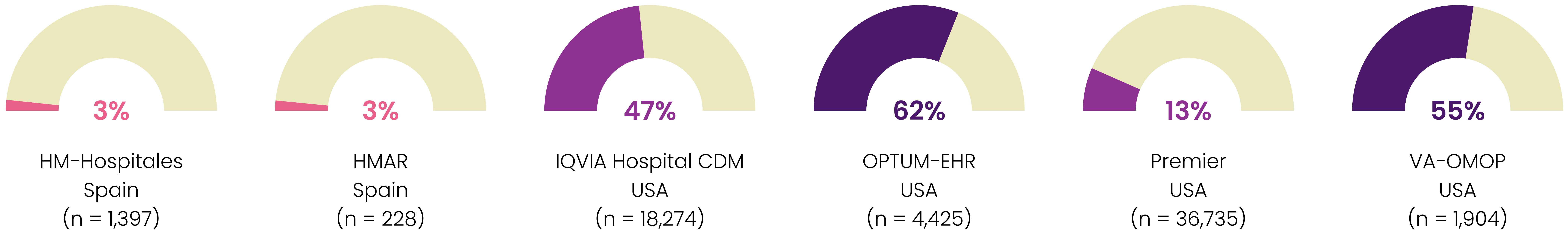

Hydroxychloroquine use in patients diagnosed or tested + for COVID

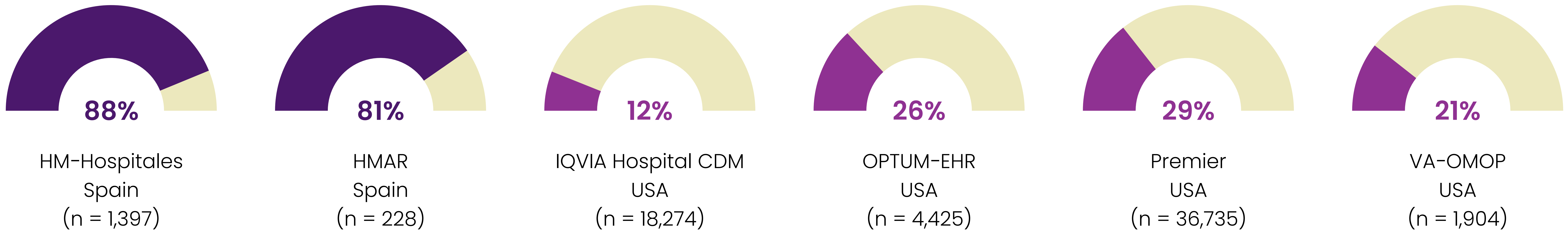

Ibrutinib use in patients diagnosed or tested + for COVID

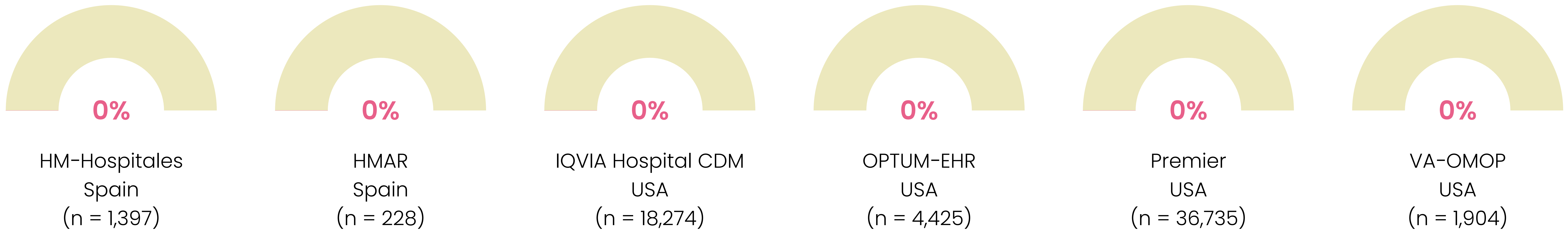

Immunoglobulins use in patients diagnosed or tested + for COVID

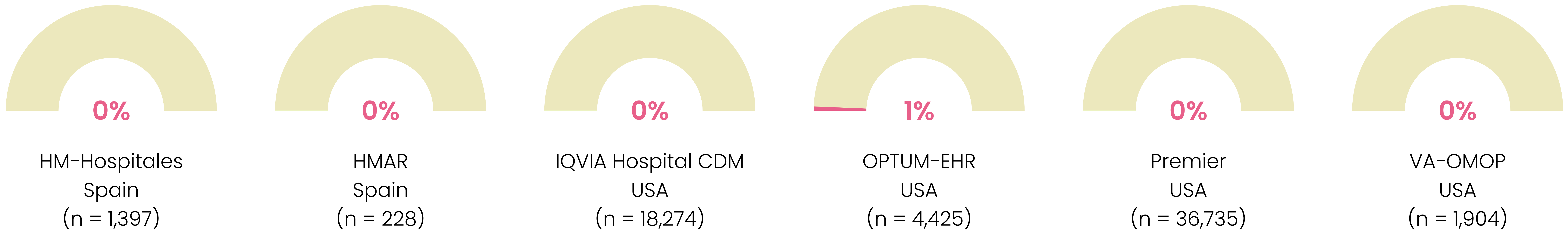

Infliximab use in patients diagnosed or tested + for COVID

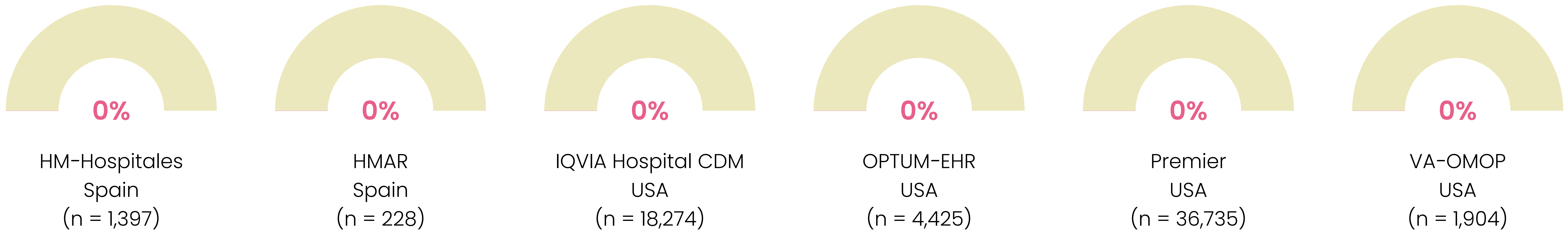

Interleukin inhibitors use in patients diagnosed or tested + for COVID

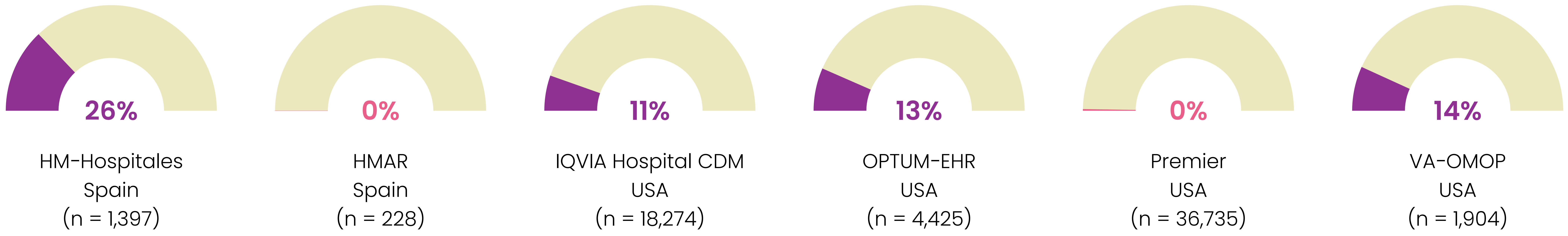

Itraconazole use in patients diagnosed or tested + for COVID

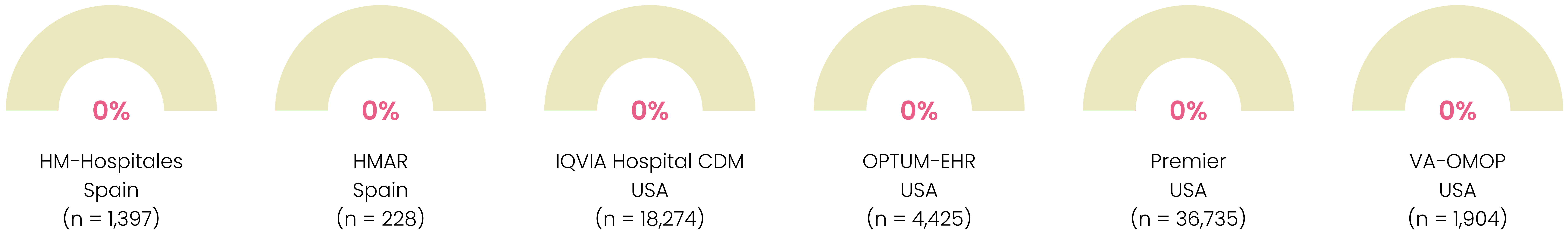

Ivermectin use in patients diagnosed or tested + for COVID

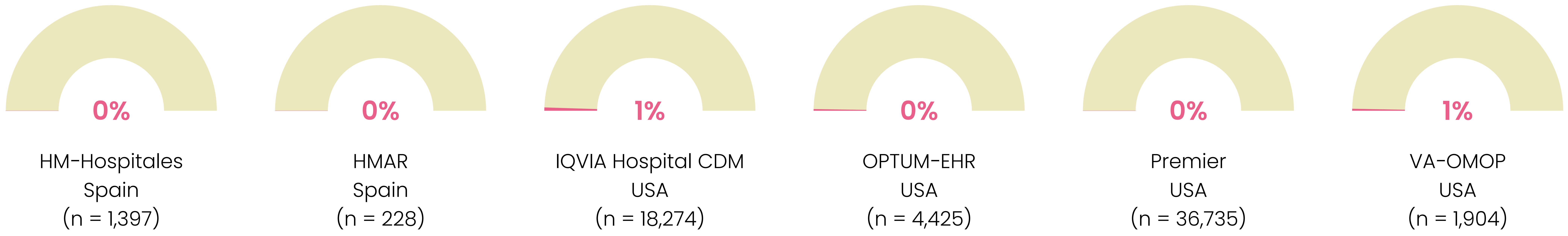

Linagliptin use in patients diagnosed or tested + for COVID

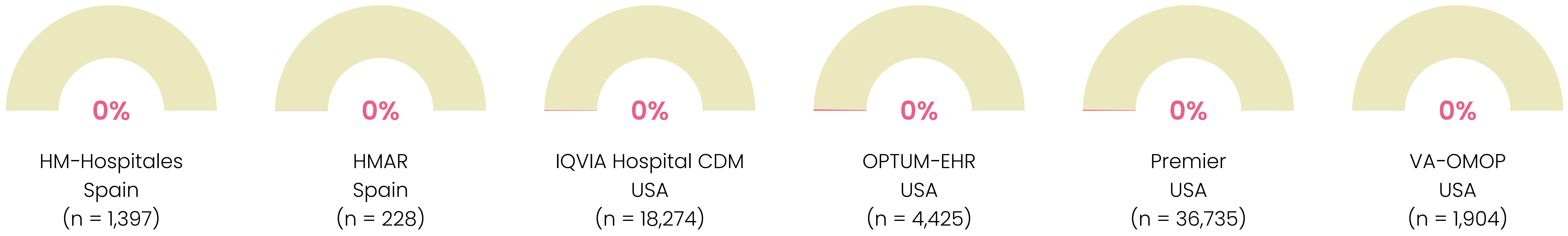

Lopinavir use in patients diagnosed or tested + for COVID

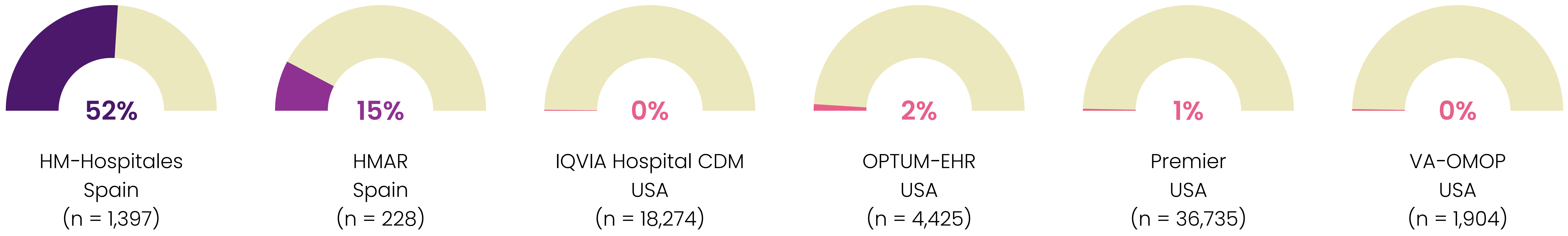

Losartan use in patients diagnosed or tested + for COVID

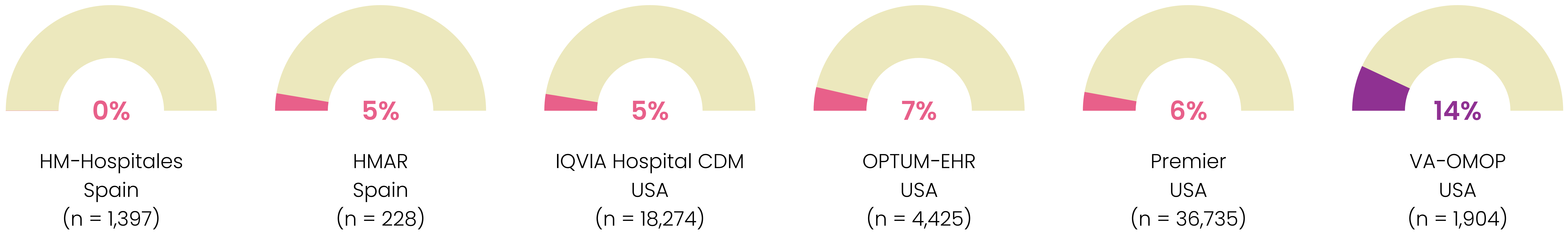

Metformin use in patients diagnosed or tested + for COVID

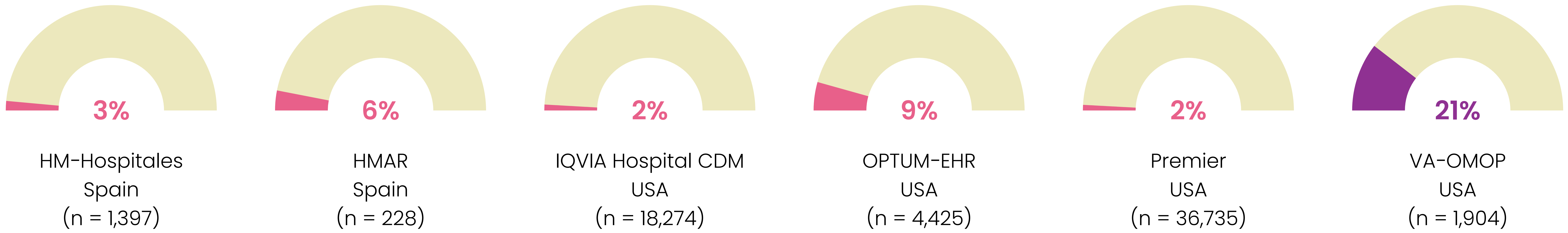

Nitazoxanide use in patients diagnosed or tested + for COVID

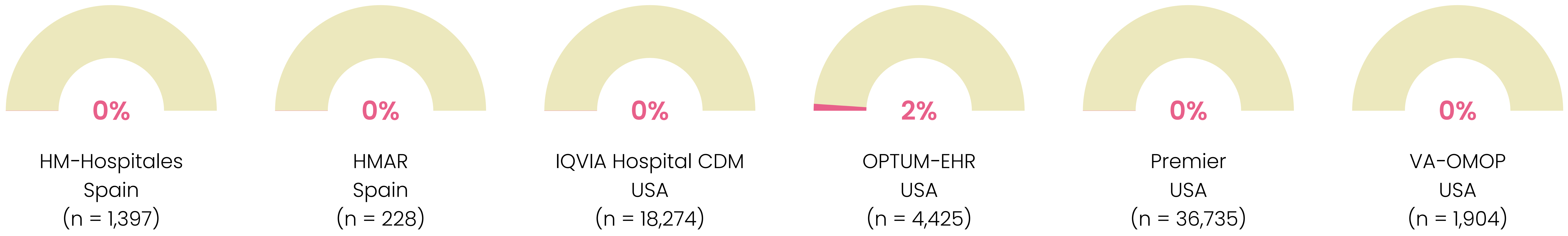

Nitric oxide use in patients diagnosed or tested + for COVID

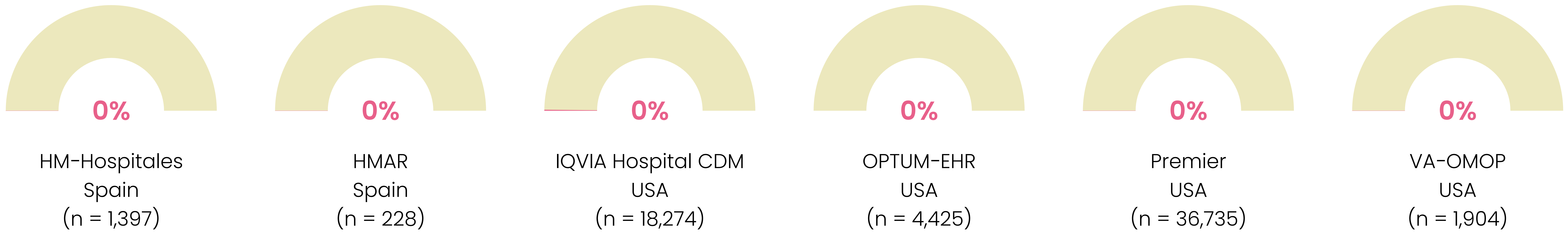

Oseltamivir use in patients diagnosed or tested + for COVID

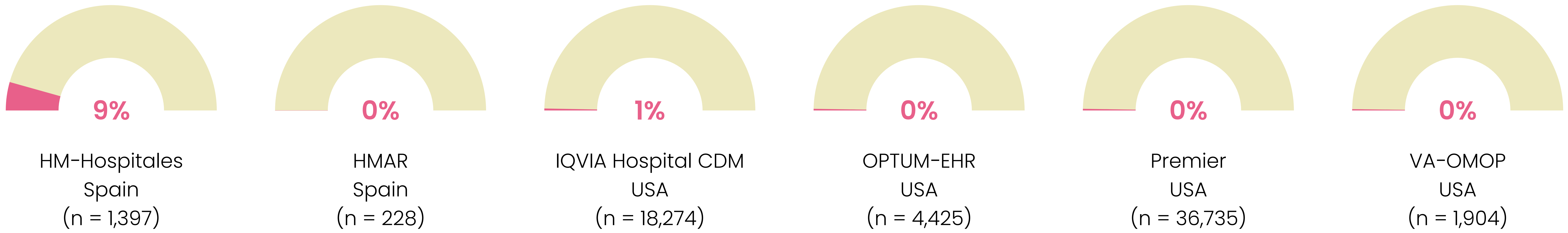

Pirfenidone use in patients diagnosed or tested + for COVID

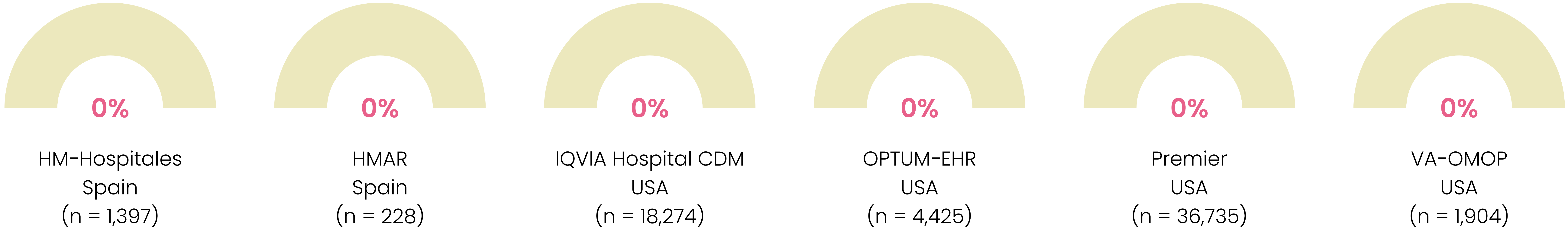

Prasugrel use in patients diagnosed or tested + for COVID

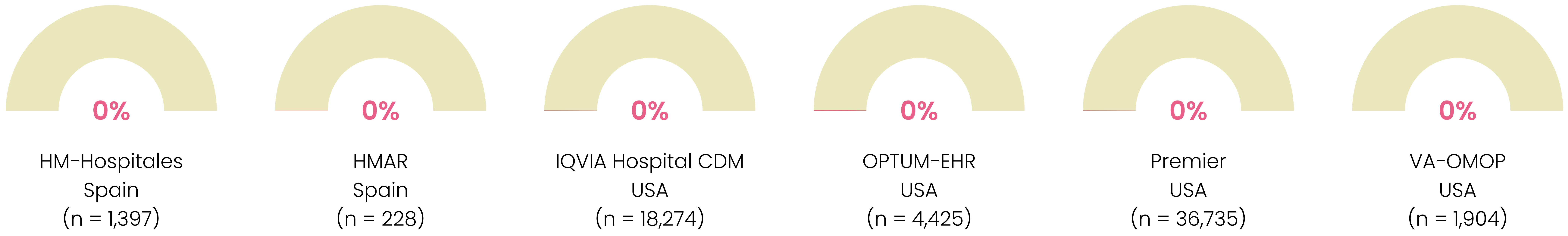

Prazosin use in patients diagnosed or tested + for COVID

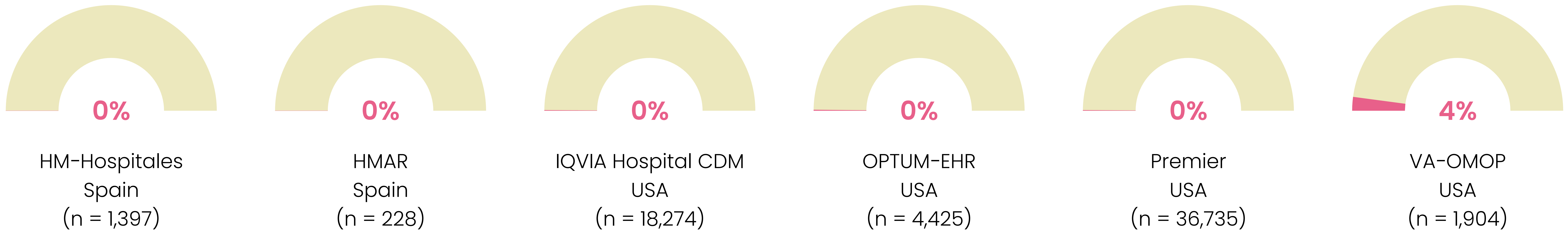

Remdesivir use in patients diagnosed or tested + for COVID

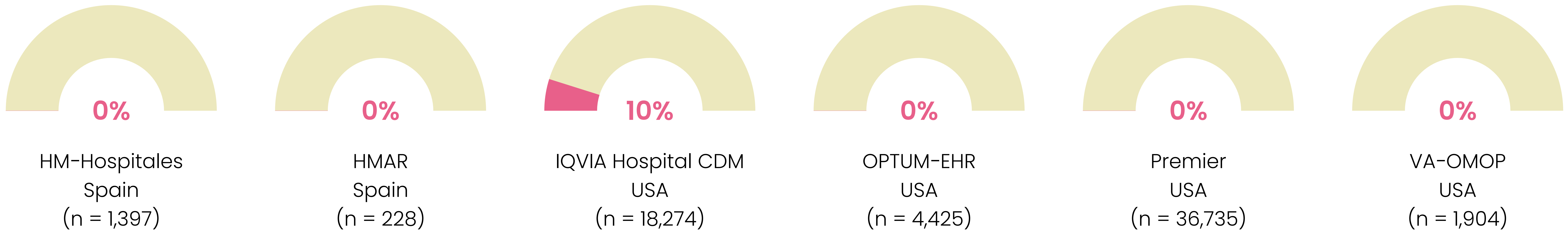

Ribavirin use in patients diagnosed or tested + for COVID

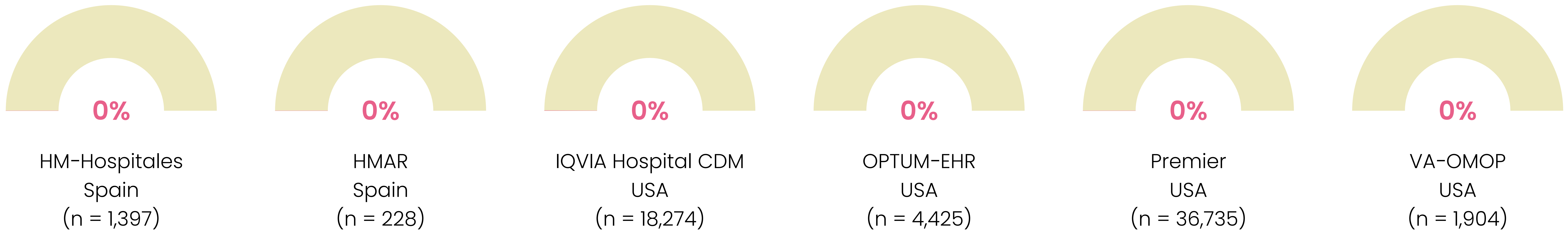

Ritonavir use in patients diagnosed or tested + for COVID

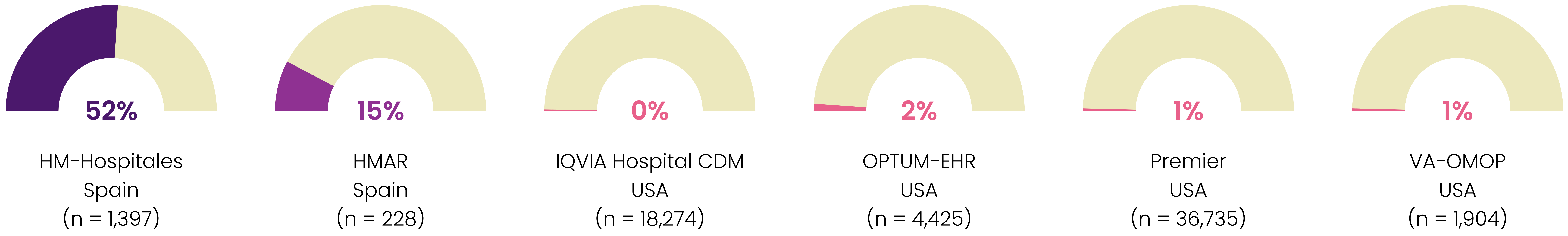

Rivaroxaban use in patients diagnosed or tested + for COVID

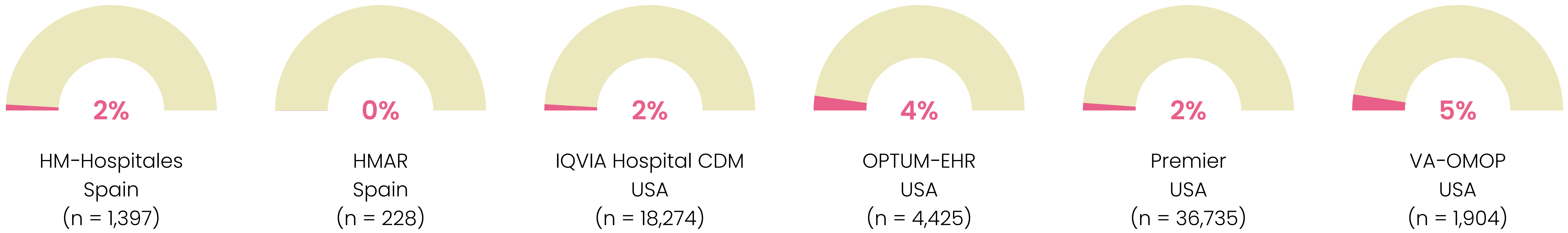

Ruxolitinib use in patients diagnosed or tested + for COVID

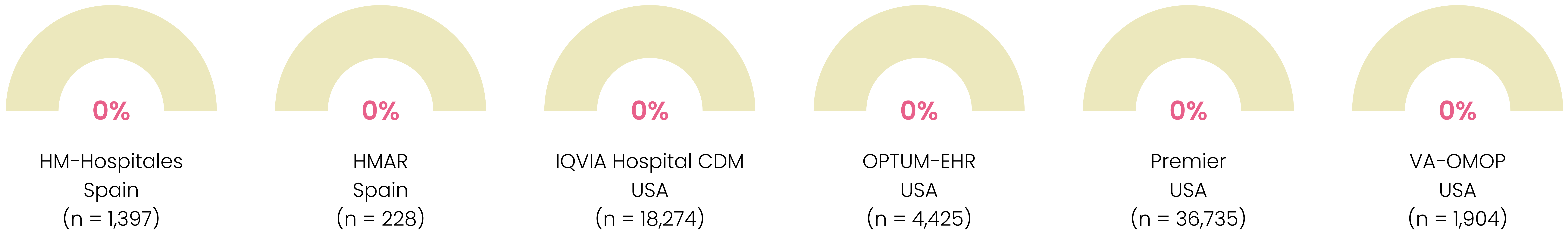

Sargramostim use in patients diagnosed or tested + for COVID

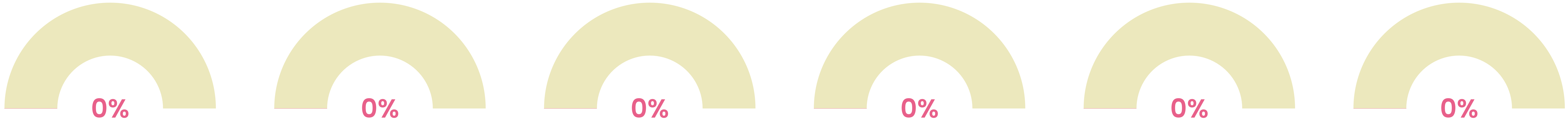

HM-Hospitales Spain (n = 1,397)

HMAR Spain (n = 228)

IQVIA Hospital CDM USA (n = 18,274)

OPTUM-EHR USA (n = 4,425)

Premier USA (n = 36,735)

VA-OMOP USA (n = 1,904)

Sarilumab use in patients diagnosed or tested + for COVID

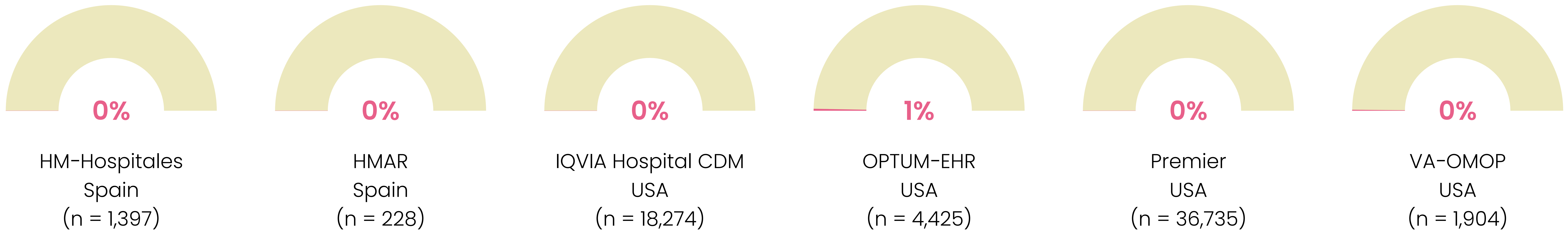

SGLT2 inhibitors use in patients diagnosed or tested + for COVID

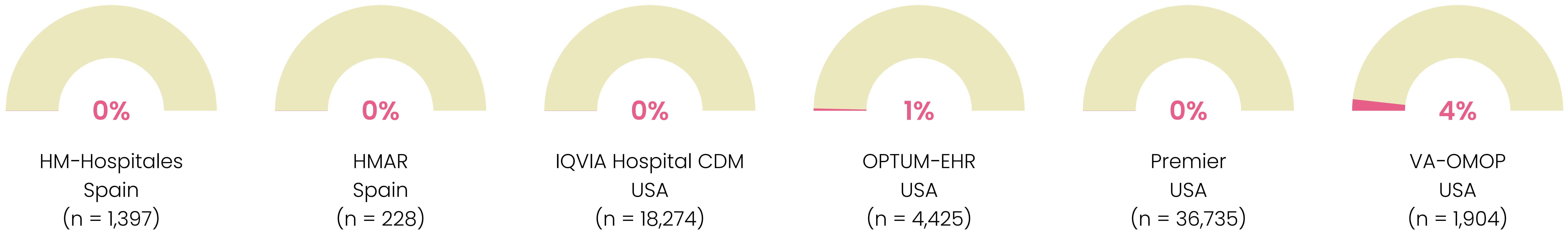

Siltuximab use in patients diagnosed or tested + for COVID

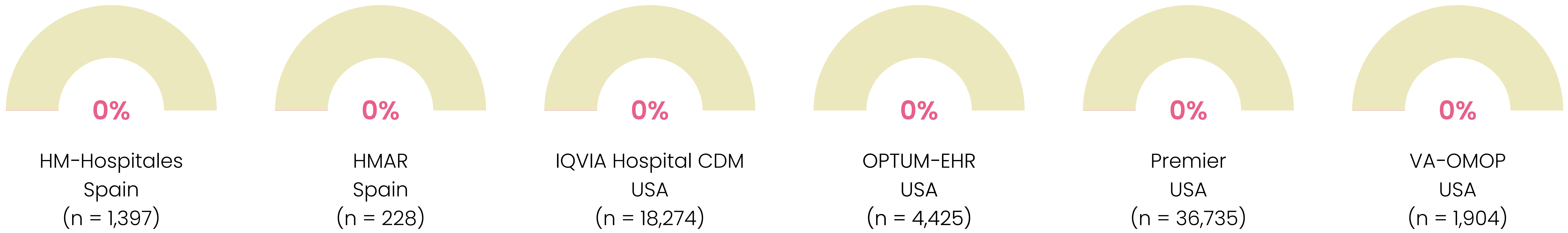

Sitagliptin use in patients diagnosed or tested + for COVID

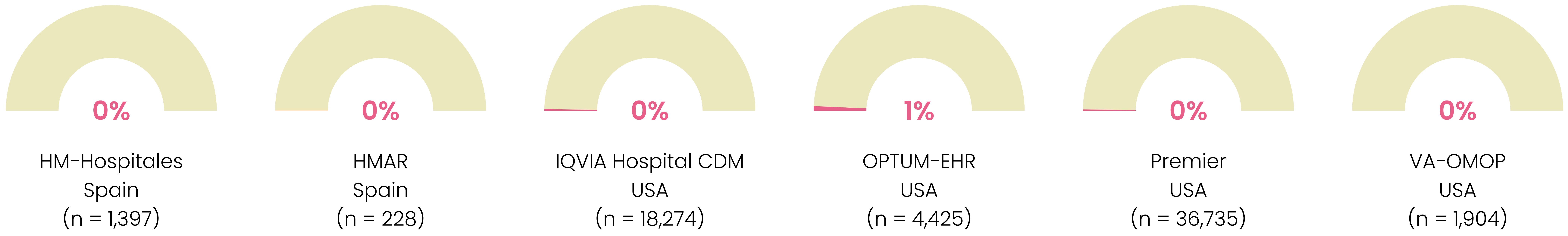

Statins use in patients diagnosed or tested + for COVID

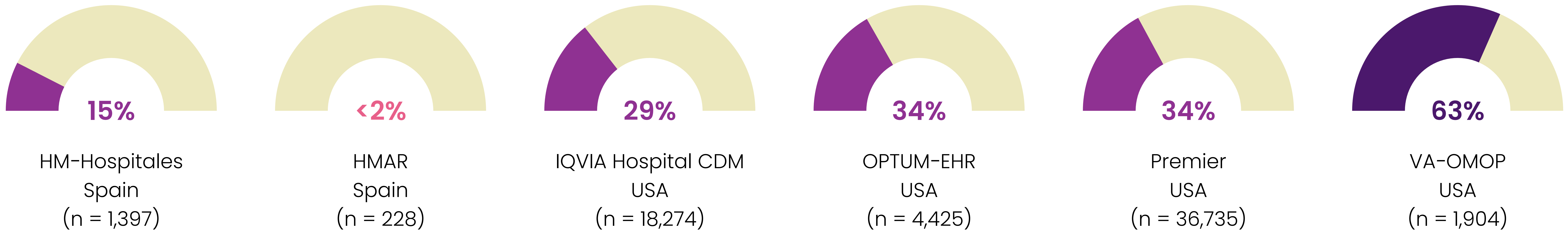

Ticagrelor use in patients diagnosed or tested + for COVID

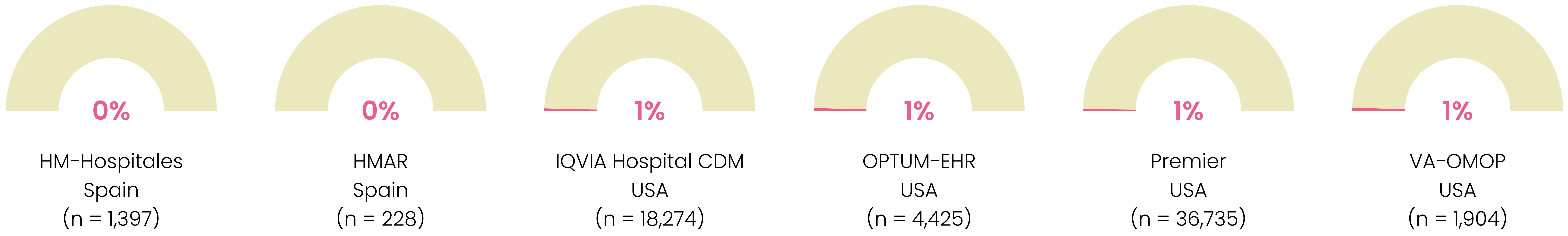

TNF inhibitors use in patients diagnosed or tested + for COVID

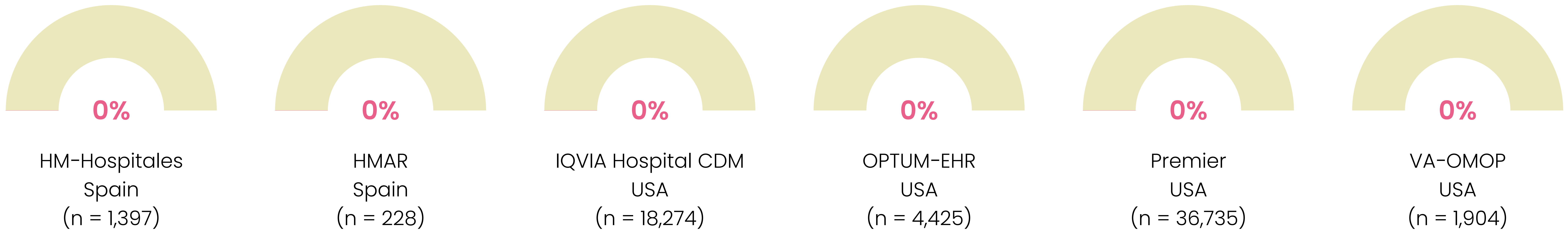

Tocilizumab use in patients diagnosed or tested + for COVID

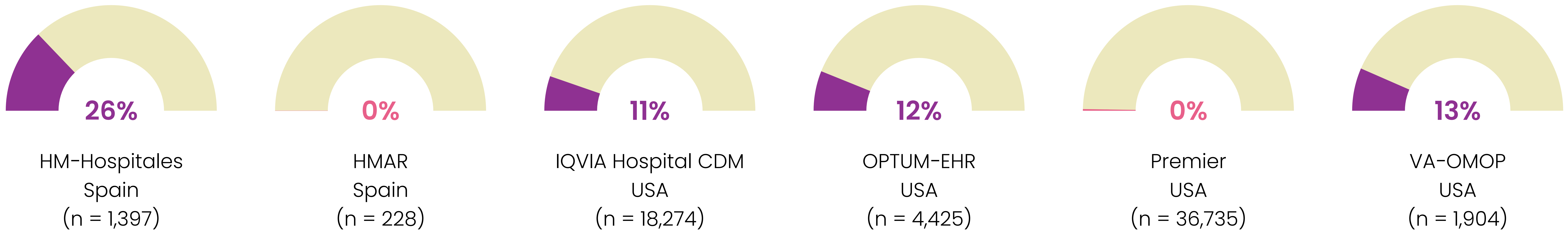

Tofacitinib use in patients diagnosed or tested + for COVID

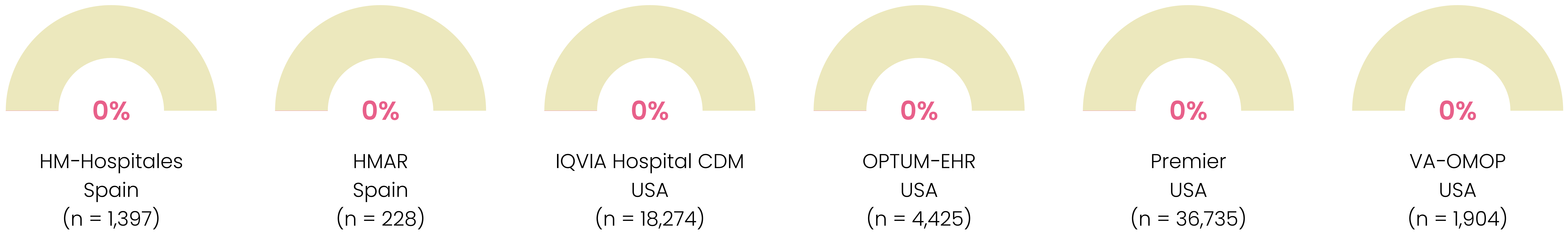

Tranexamic acid use in patients diagnosed or tested + for COVID

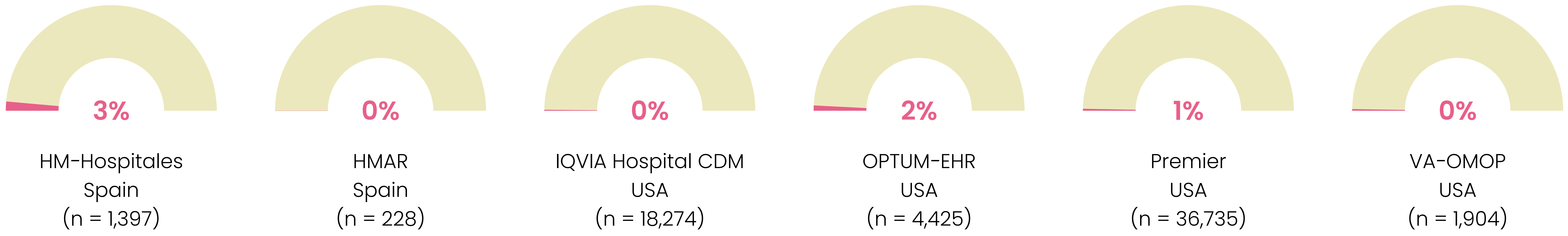

Ustekinumab use in patients diagnosed or tested + for COVID

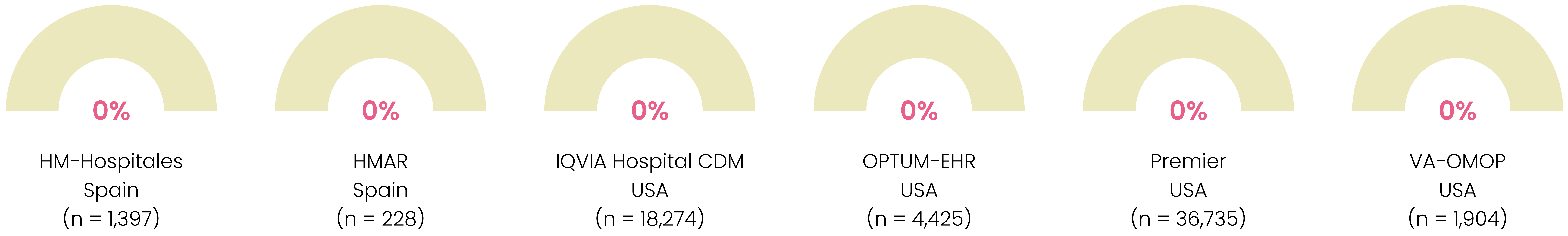

Vitamin C use in patients diagnosed or tested + for COVID

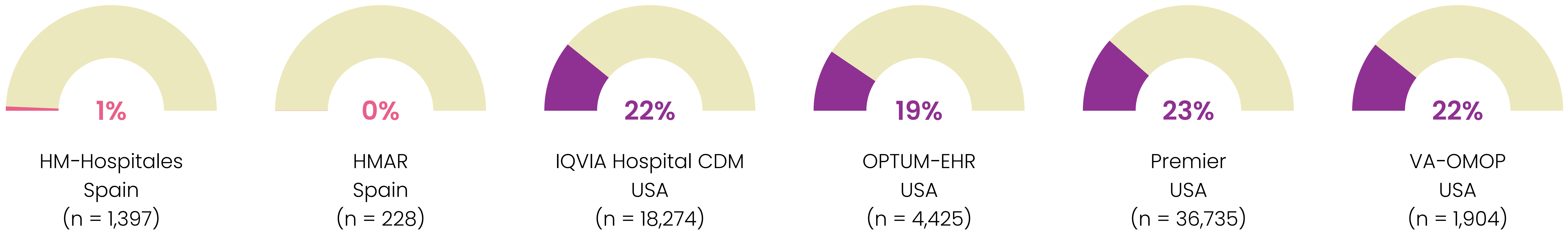

Vitamin D use in patients diagnosed or tested + for COVID

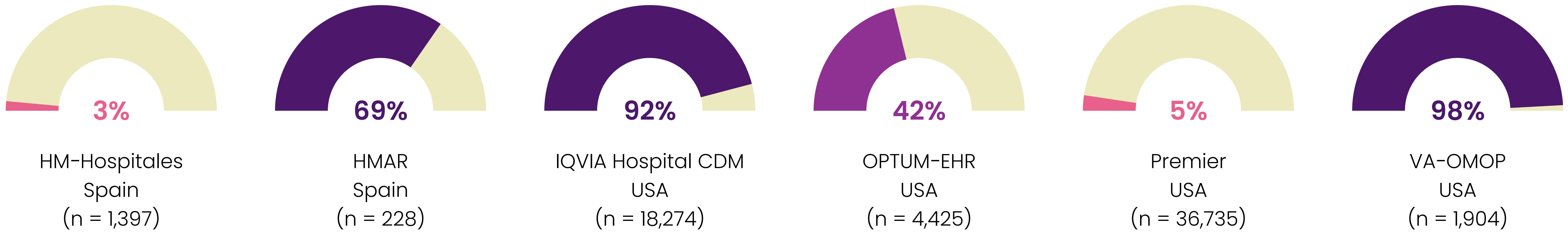

Warfarin use in patients diagnosed or tested + for COVID

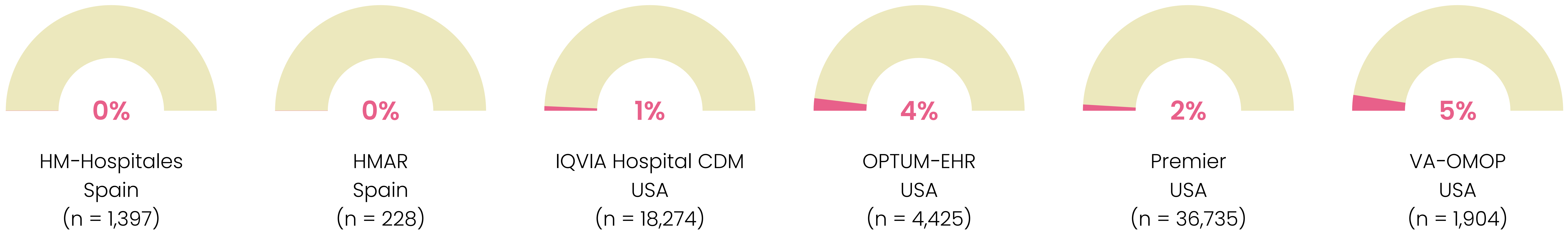

Supplement: Supplementary file 3 — Web appendix: Supplementary figure 5 [file praa062143.wf5.pdf]
