# Supplementary material for: Use of repurposed and adjuvant drugs in hospital patients with covid-19: multinational network cohort study
Source: BMJ. 2021 May 11;373:n1038. doi: 10.1136/bmj.n1038 (PMC8111167; doi:10.1136/bmj.n1038)
Supplement: Supplementary file 4 — Web appendix: Supplementary figure 6 [file praa062143.wf6.pdf]

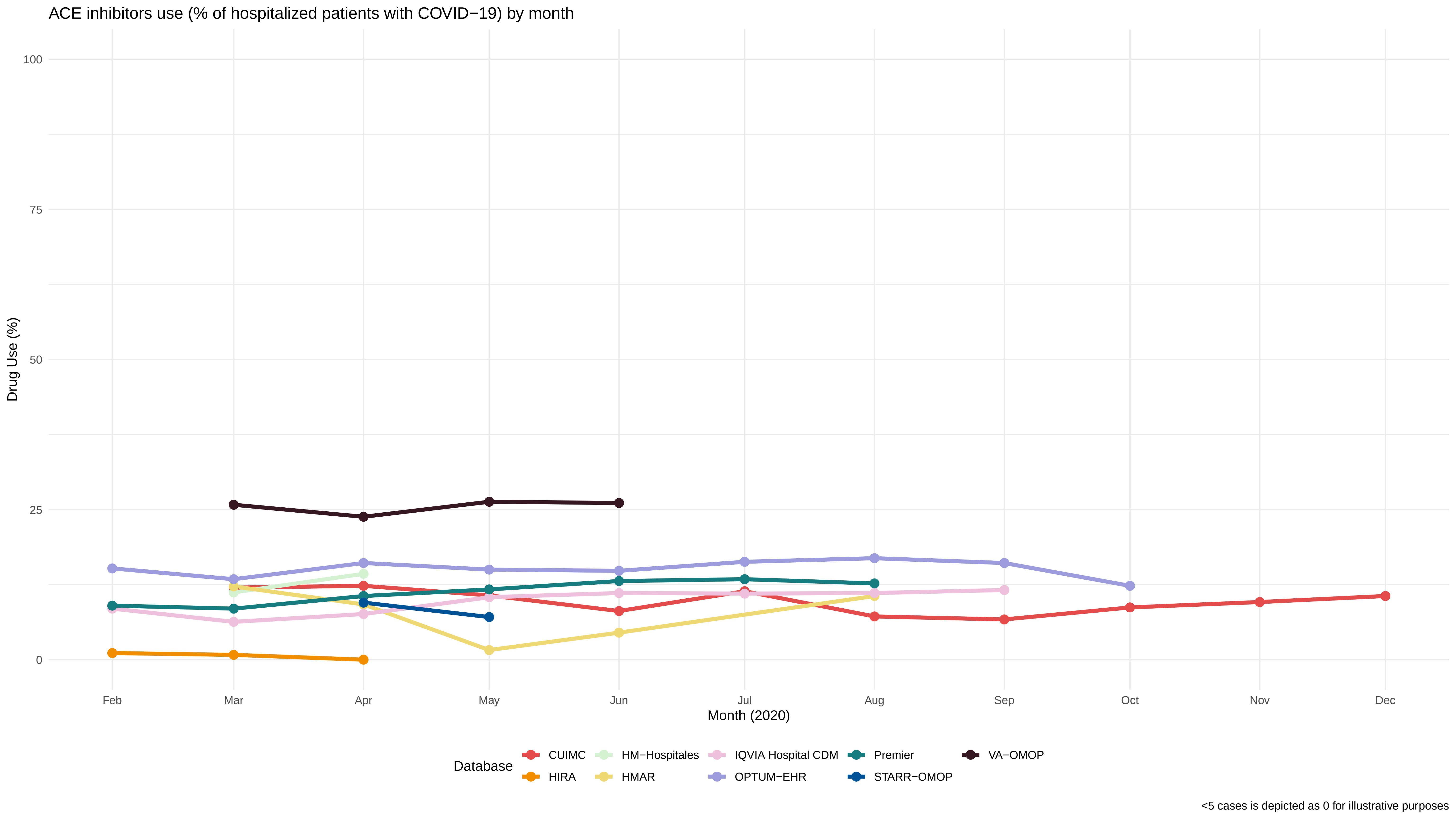
ACE inhibitors use (% of hospitalized patients with COVID−19) by month
Drug Use (%)
100
75
50
25
0
Feb
Mar
Apr
May
Jun
Jul
Aug
Sep
Oct
Nov
Dec
Month (2020)
Database
CUIMC
HIRA
HM−Hospitales
HMAR
IQVIA Hospital CDM
OPTUM−EHR
Premier
STARR−OMOP
VA−OMOP
<5 cases is depicted as 0 for illustrative purposes

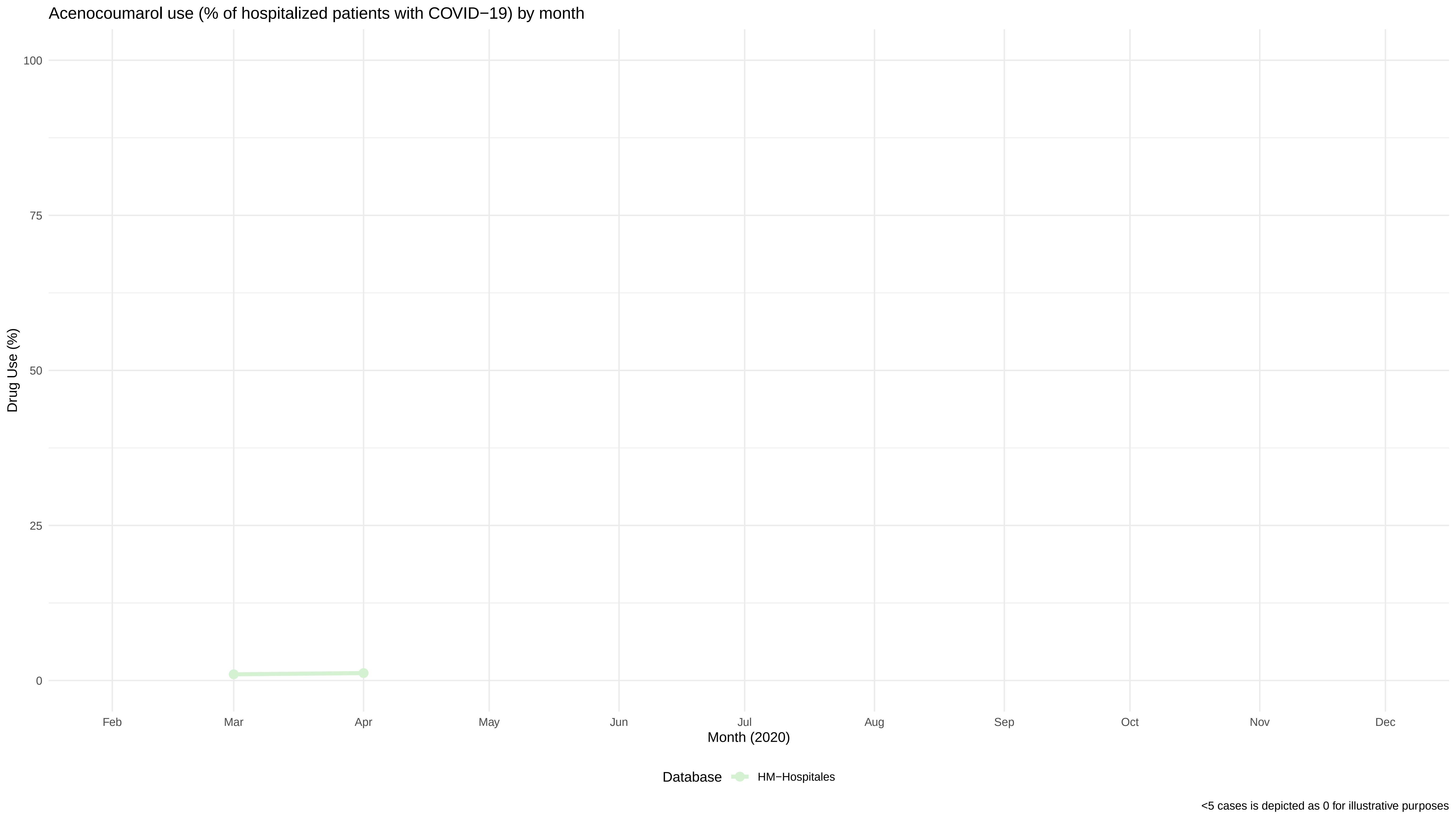
Acenocoumarol use (% of hospitalized patients with COVID−19) by month
Drug Use (%)
100
75
50
25
0
Feb
Mar
Apr
May
Jun
Jul
Aug
Sep
Oct
Nov
Dec
Month (2020)
Database
HM−Hospitales
<5 cases is depicted as 0 for illustrative purposes

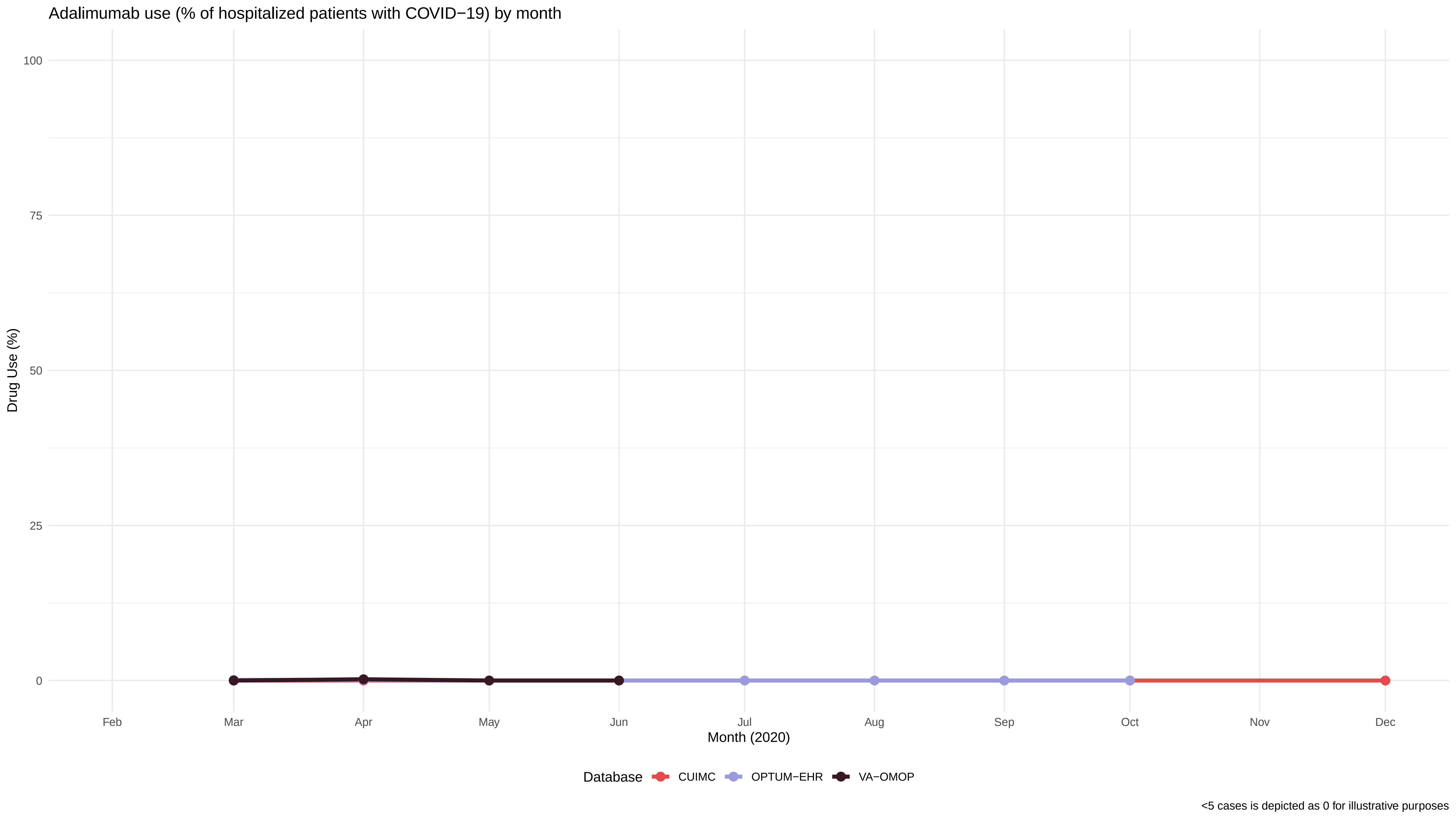
Adalimumab use (% of hospitalized patients with COVID−19) by month
Drug Use (%)
100
75
50
25
0
Feb
Mar
Apr
May
Jun
Jul
Aug
Sep
Oct
Nov
Dec
Month (2020)
Database
CUIMC
OPTUM−EHR
VA−OMOP
<5 cases is depicted as 0 for illustrative purposes

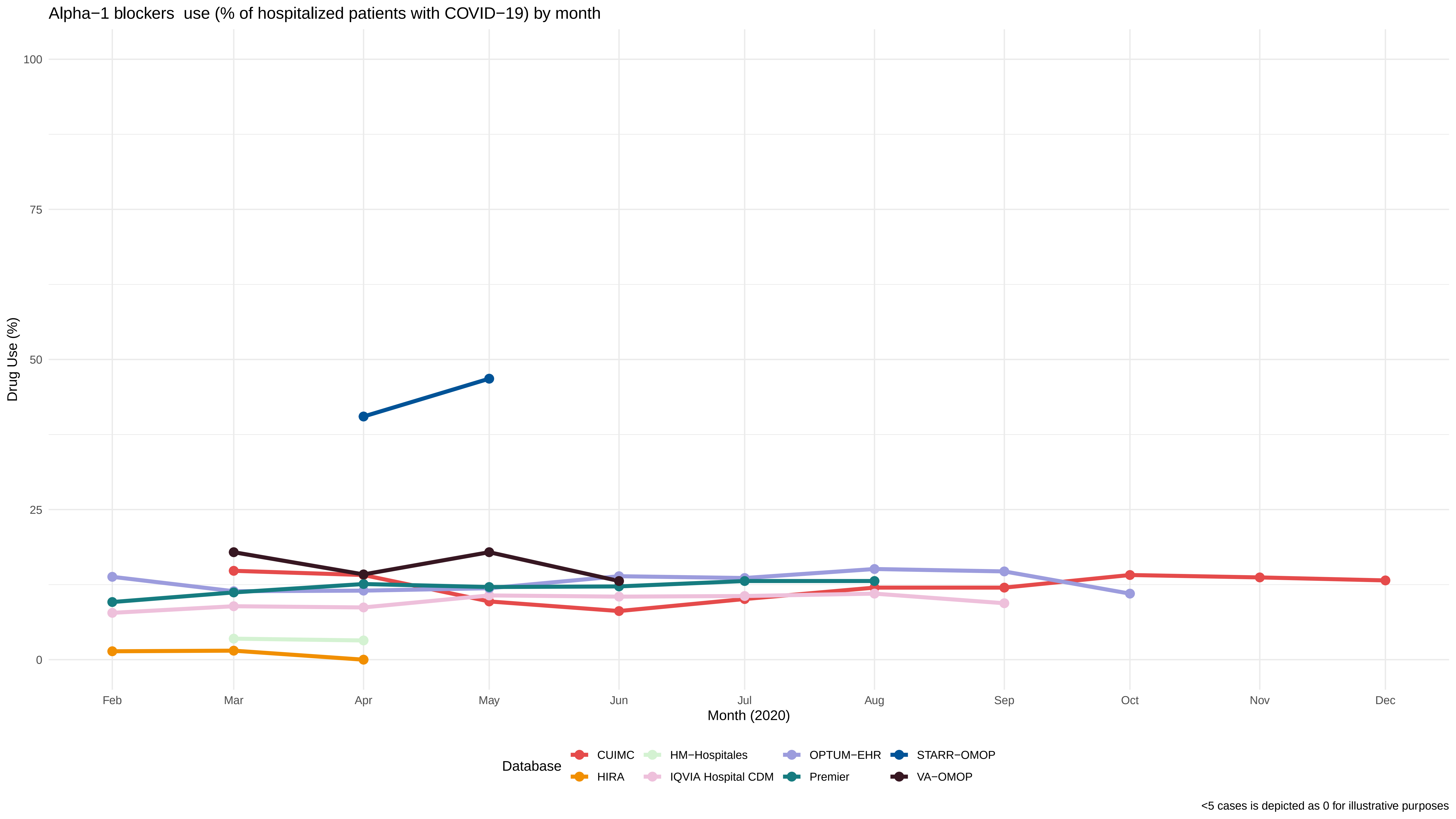
Alpha−1 blockers use (% of hospitalized patients with COVID−19) by month
Drug Use (%)
100
75
50
25
0
Feb
Mar
Apr
May
Jun
Jul
Aug
Sep
Oct
Nov
Dec
Month (2020)
Database
CUIMC
HIRA
HM−Hospitales
IQVIA Hospital CDM
OPTUM−EHR
Premier
STARR−OMOP
VA−OMOP
<5 cases is depicted as 0 for illustrative purposes

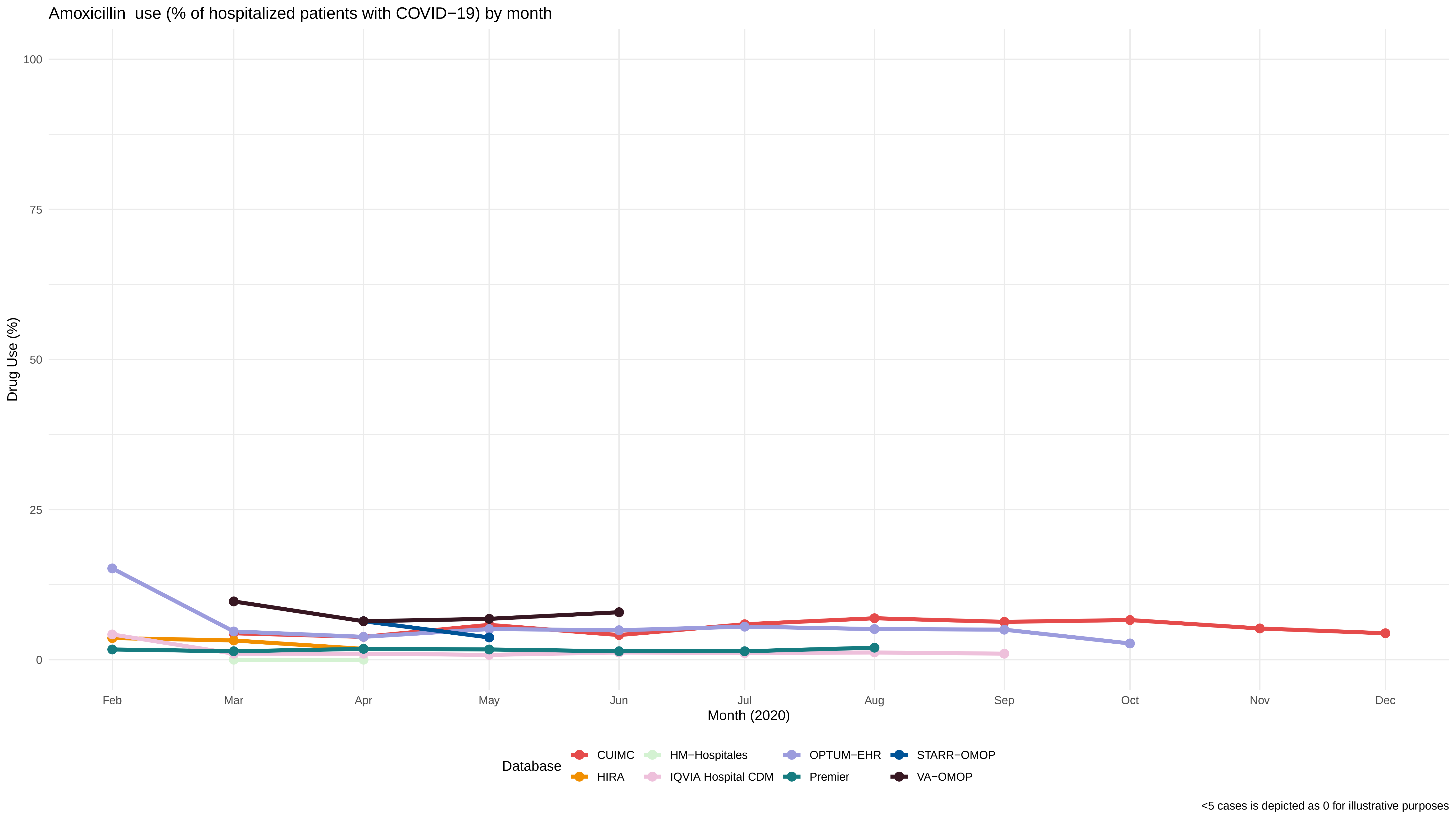
Amoxicillin use (% of hospitalized patients with COVID−19) by month
Drug Use (%)
100
75
50
25
0
Feb
Mar
Apr
May
Jun
Jul
Aug
Sep
Oct
Nov
Dec
Month (2020)
Database
CUIMC
HIRA
HM−Hospitales
IQVIA Hospital CDM
OPTUM−EHR
Premier
STARR−OMOP
VA−OMOP
<5 cases is depicted as 0 for illustrative purposes

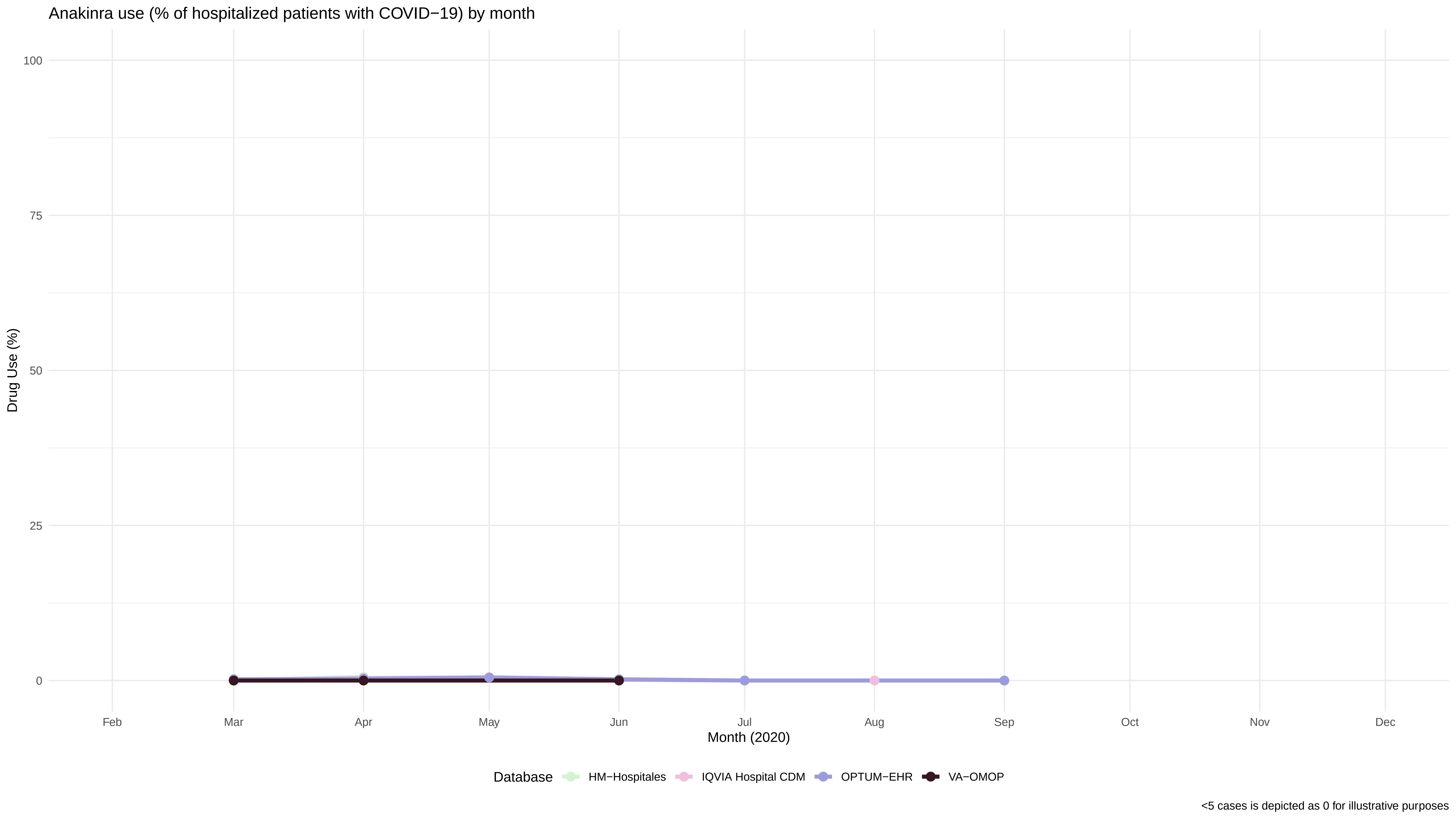
Anakinra use (% of hospitalized patients with COVID−19) by month
Drug Use (%)
100
75
50
25
0
Feb
Mar
Apr
May
Jun
Jul
Aug
Sep
Oct
Nov
Dec
Month (2020)
Database
HM−Hospitales
IQVIA Hospital CDM
OPTUM−EHR
VA−OMOP
<5 cases is depicted as 0 for illustrative purposes

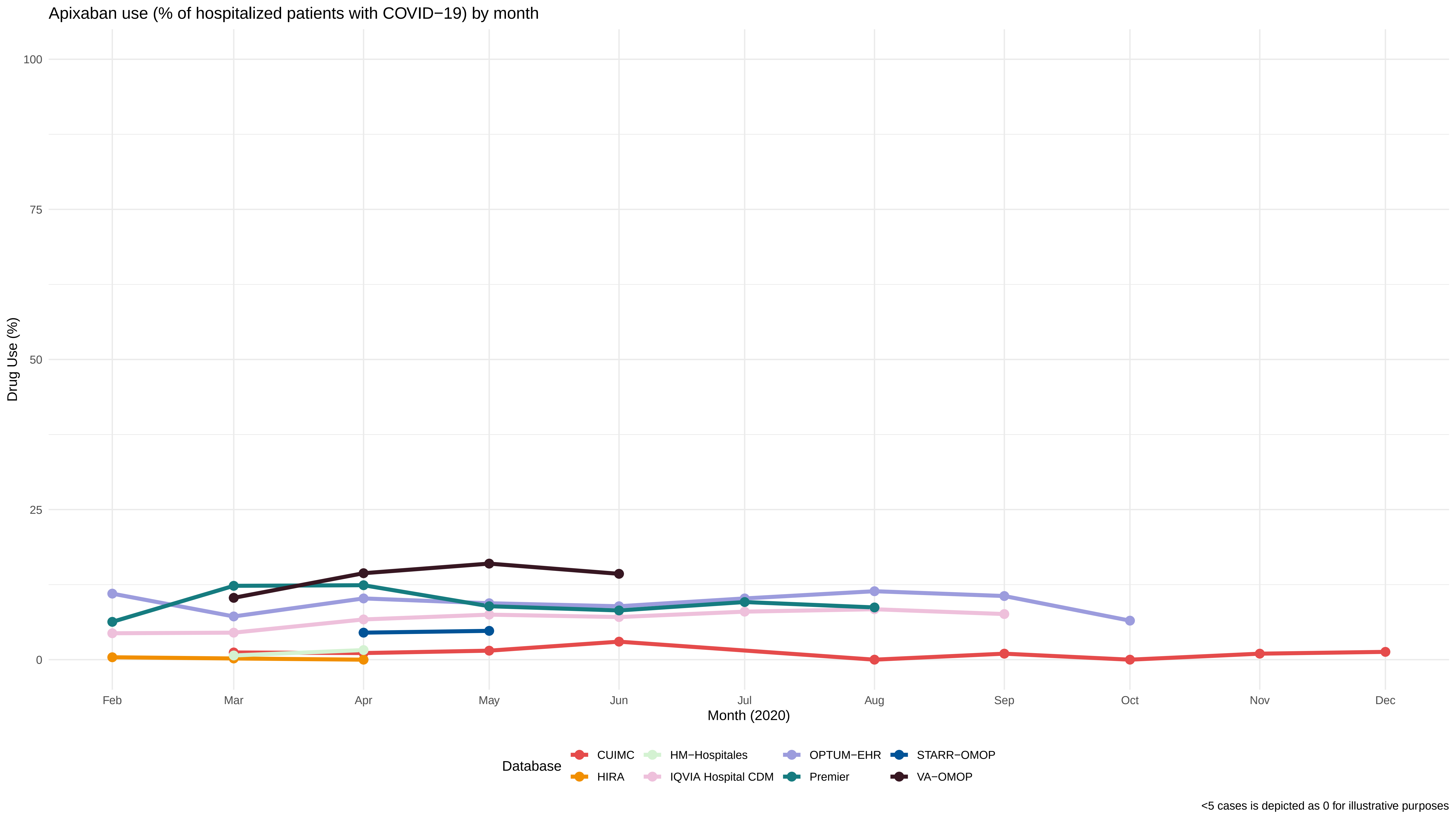
Apixaban use (% of hospitalized patients with COVID−19) by month
Drug Use (%)
100
75
50
25
0
Feb
Mar
Apr
May
Jun
Jul
Aug
Sep
Oct
Nov
Dec
Month (2020)
Database
CUIMC
HIRA
HM−Hospitales
IQVIA Hospital CDM
OPTUM−EHR
Premier
STARR−OMOP
VA−OMOP
<5 cases is depicted as 0 for illustrative purposes

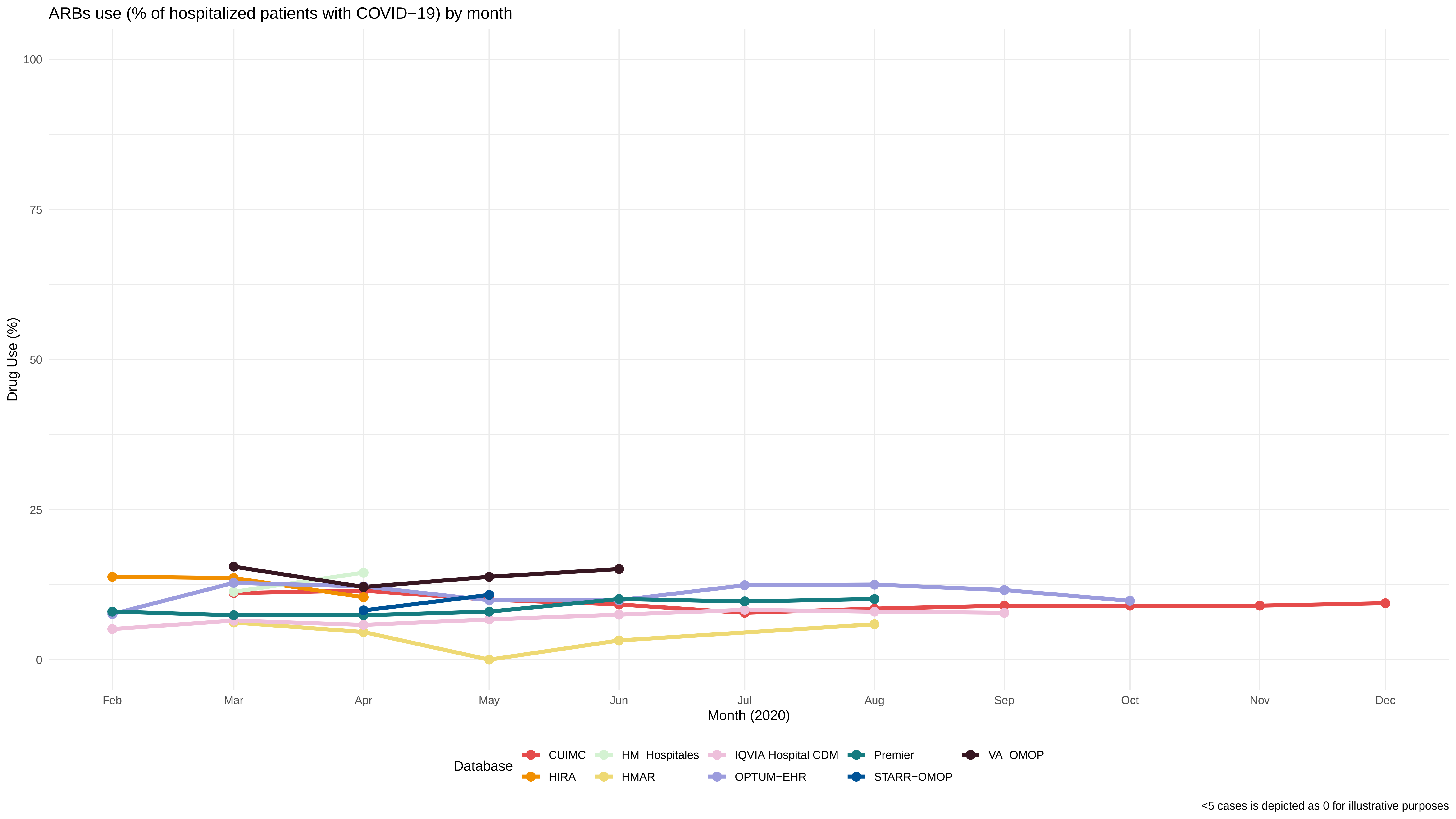
ARBs use (% of hospitalized patients with COVID−19) by month
100
75
50
25
0
Drug Use (%)
Feb
Mar
Apr
May
Jun
Jul
Aug
Sep
Oct
Nov
Dec
Month (2020)
Database
CUIMC
HIRA
HM−Hospitales
HMAR
IQVIA Hospital CDM
OPTUM−EHR
Premier
STARR−OMOP
VA−OMOP
<5 cases is depicted as 0 for illustrative purposes

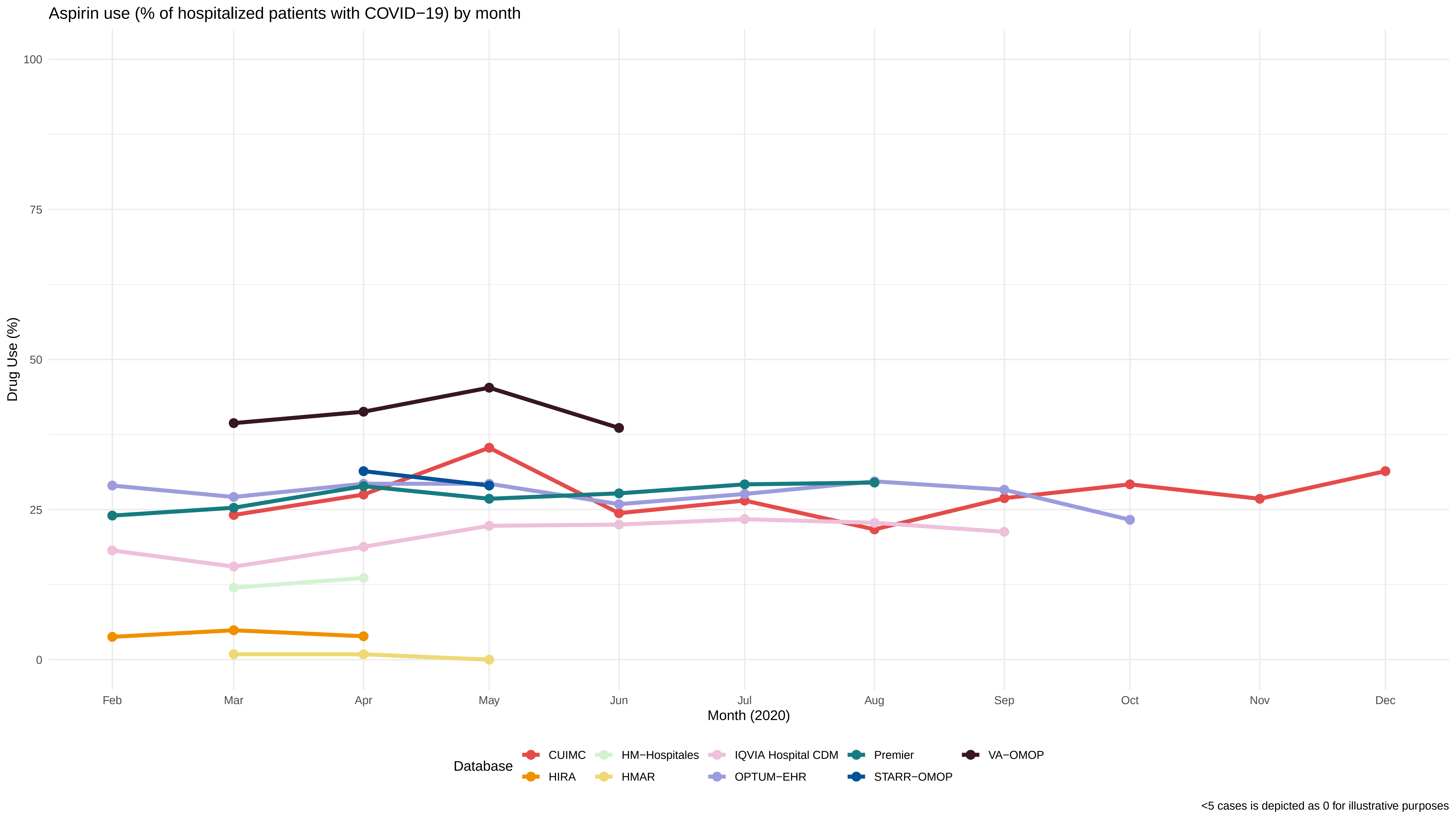
Aspirin use (% of hospitalized patients with COVID−19) by month
100
75
50
25
0
Drug Use (%)
Feb
Mar
Apr
May
Jun
Jul
Aug
Sep
Oct
Nov
Dec
Month (2020)
Database
CUIMC
HIRA
HM−Hospitales
HMAR
IQVIA Hospital CDM
OPTUM−EHR
Premier
STARR−OMOP
VA−OMOP
<5 cases is depicted as 0 for illustrative purposes

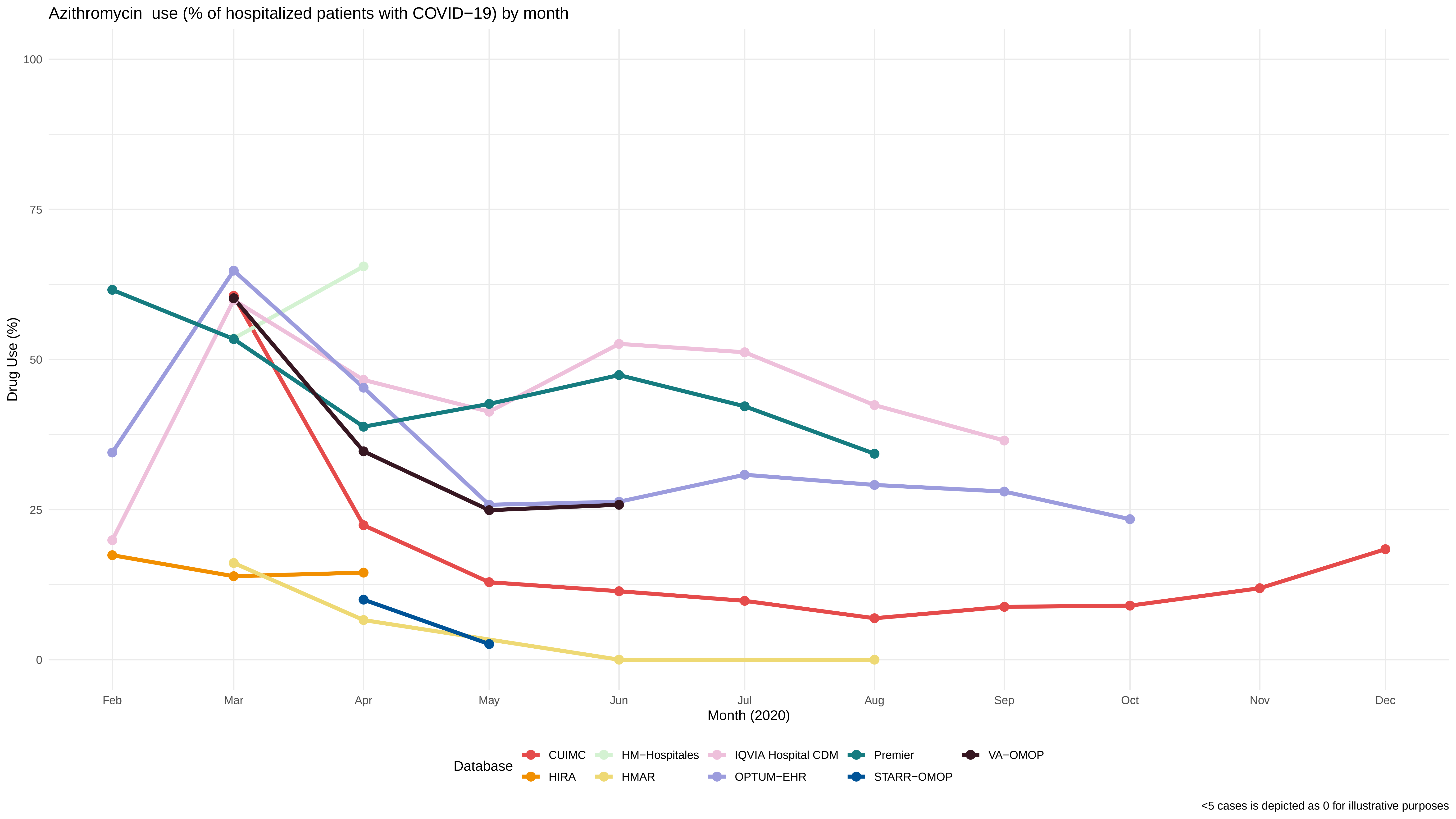
Azithromycin use (% of hospitalized patients with COVID−19) by month
Drug Use (%)
100
75
50
25
0
Feb
Mar
Apr
May
Jun
Jul
Aug
Sep
Oct
Nov
Dec
Month (2020)
Database
CUIMC
HIRA
HM−Hospitales
HMAR
IQVIA Hospital CDM
OPTUM−EHR
Premier
STARR−OMOP
VA−OMOP
<5 cases is depicted as 0 for illustrative purposes

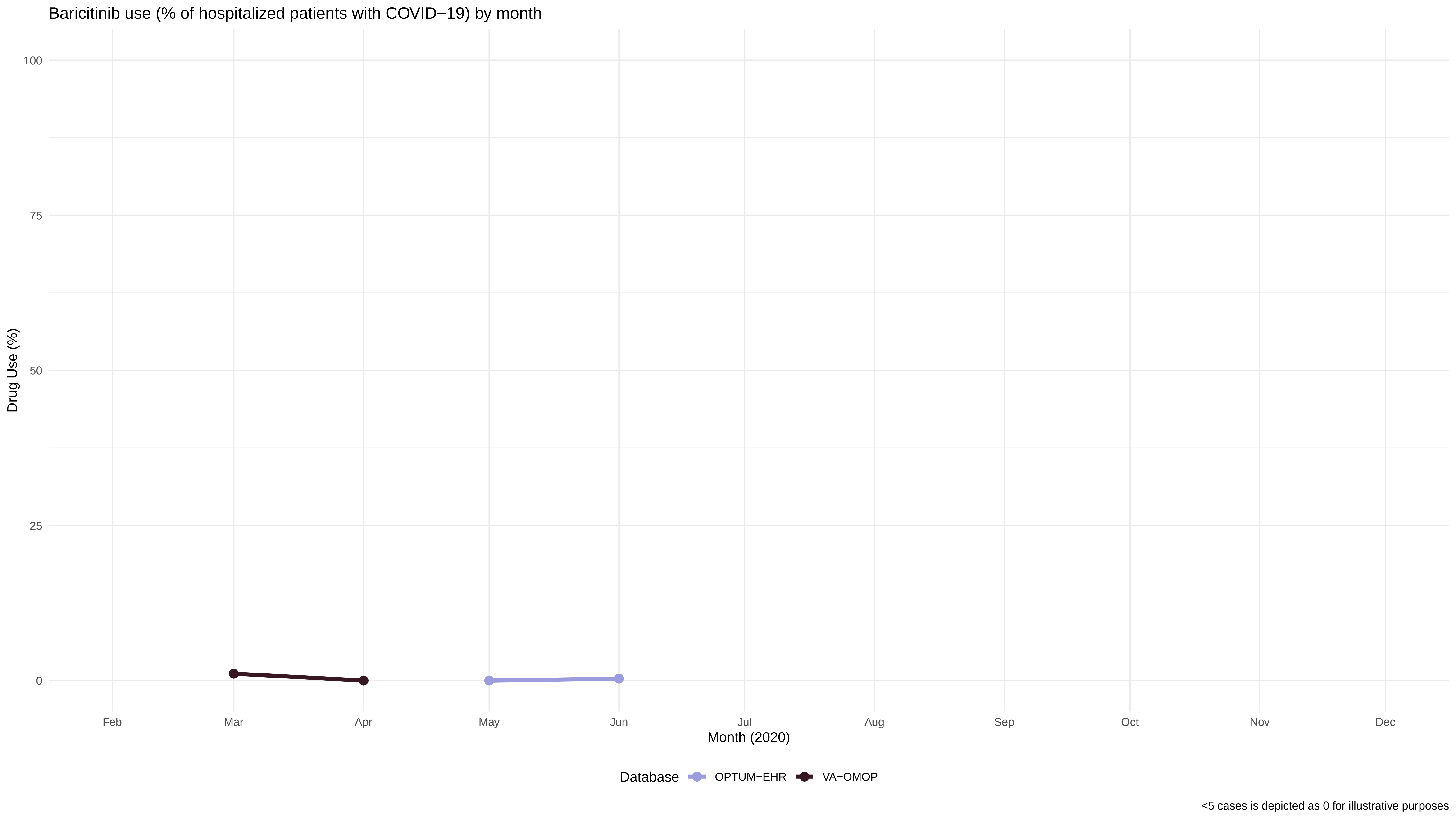
Baricitinib use (% of hospitalized patients with COVID−19) by month
Drug Use (%)
100
75
50
25
0
Feb
Mar
Apr
May
Jun
Jul
Aug
Sep
Oct
Nov
Dec
Month (2020)
Database
OPTUM−EHR
VA−OMOP
<5 cases is depicted as 0 for illustrative purposes

Bemiparin use (% of hospitalized patients with COVID−19) by month

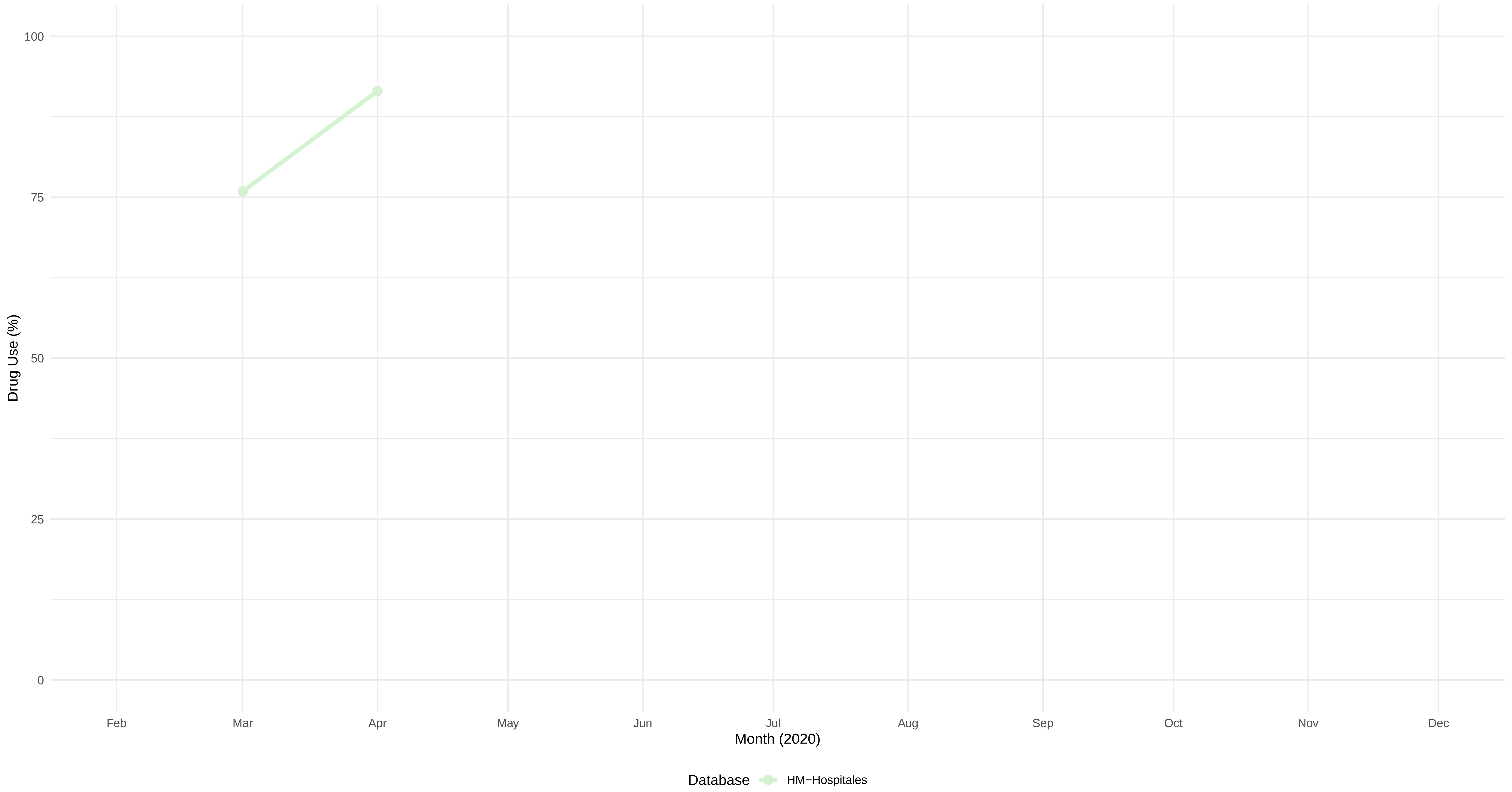

<5 cases is depicted as 0 for illustrative purposes

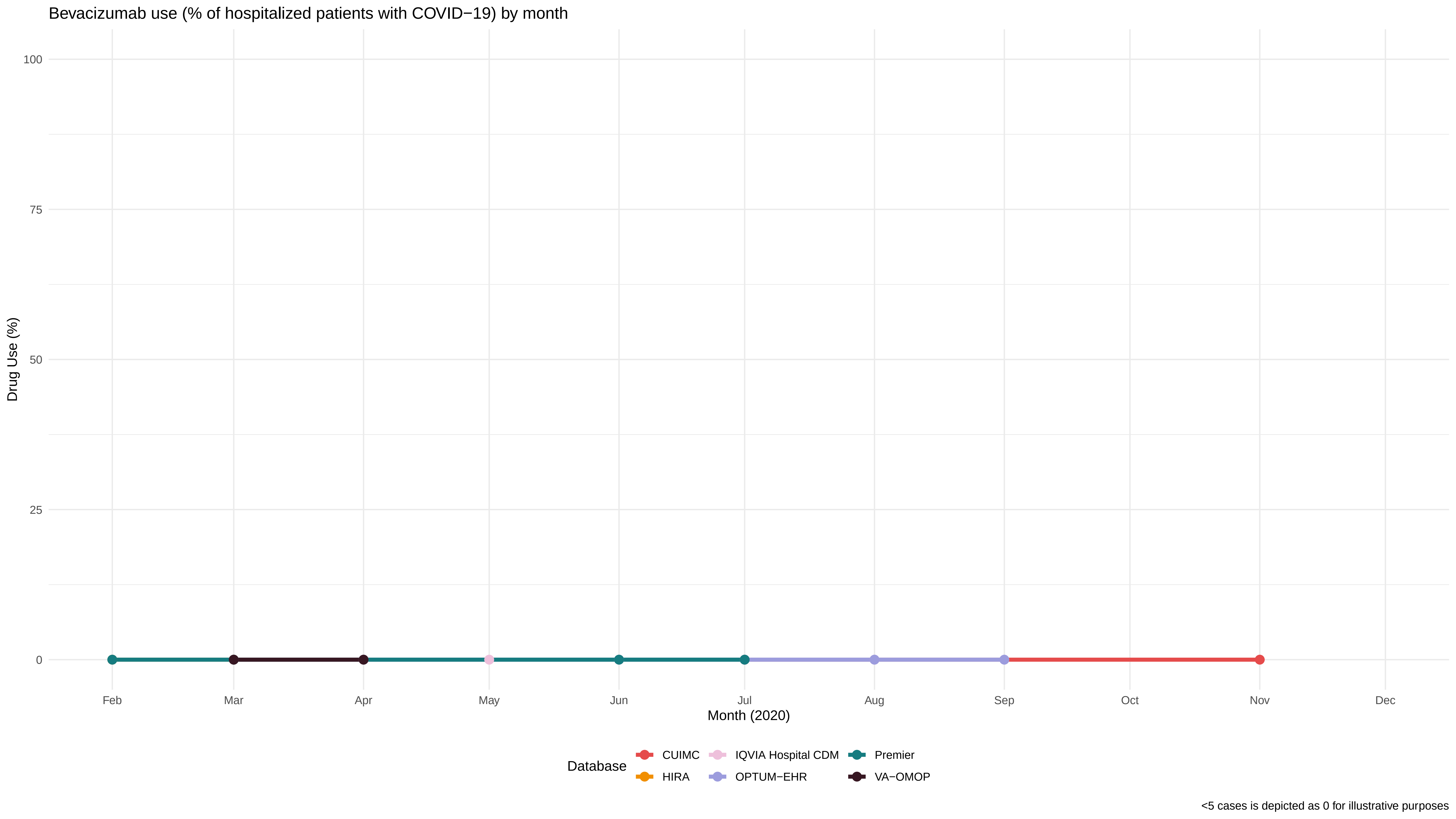
Bevacizumab use (% of hospitalized patients with COVID−19) by month
Drug Use (%)
100
75
50
25
0
Feb
Mar
Apr
May
Jun
Jul
Aug
Sep
Oct
Nov
Dec
Month (2020)
Database
CUIMC
HIRA
IQVIA Hospital CDM
OPTUM−EHR
Premier
VA−OMOP
<5 cases is depicted as 0 for illustrative purposes

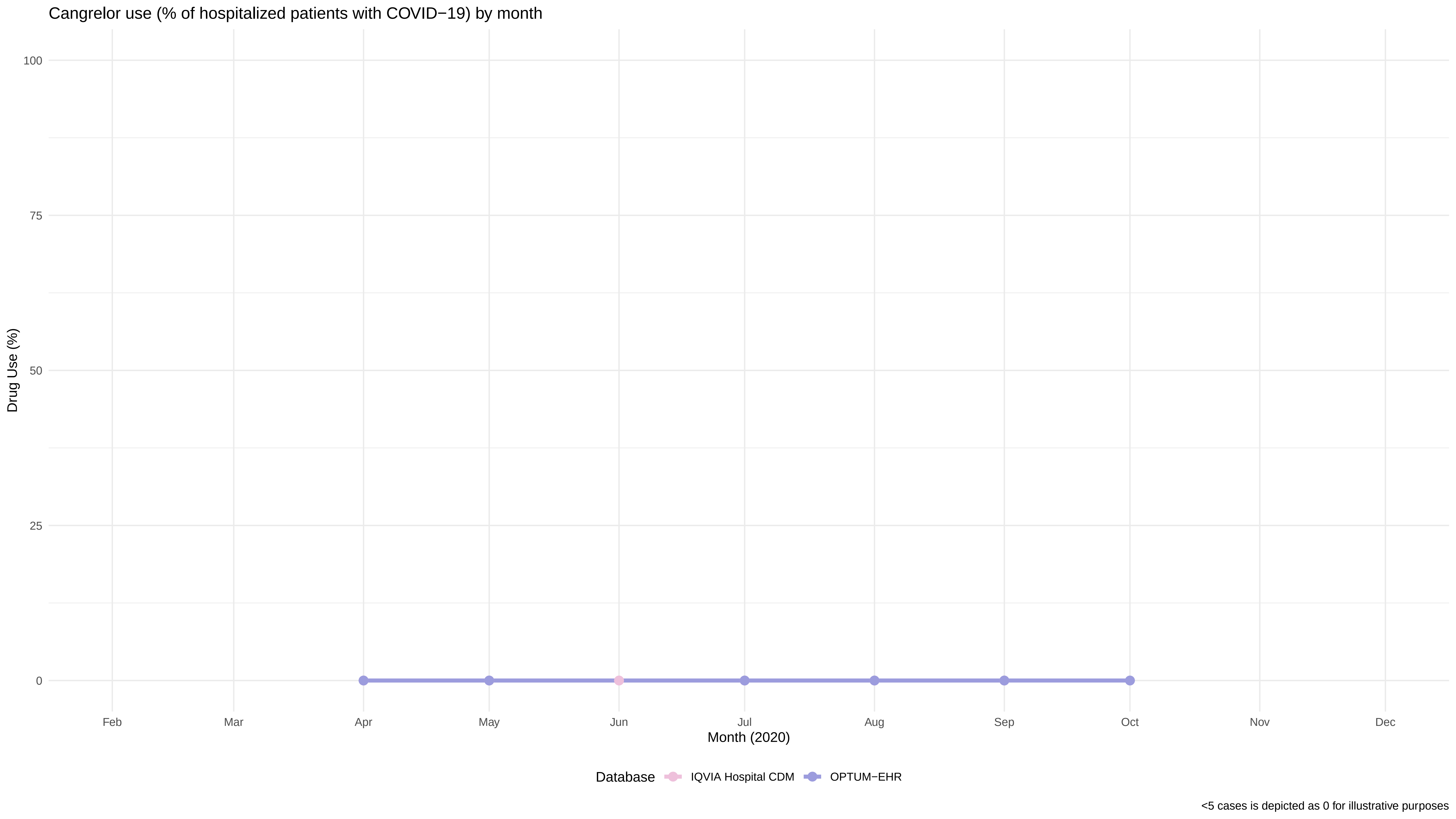

Cangrelor use (% of hospitalized patients with COVID−19) by month
Drug Use (%)
100
75
50
25
0
Feb
Mar
Apr
May
Jun
Jul
Aug
Sep
Oct
Nov
Dec
Month (2020)
Database
IQVIA Hospital CDM
OPTUM−EHR
<5 cases is depicted as 0 for illustrative purposes

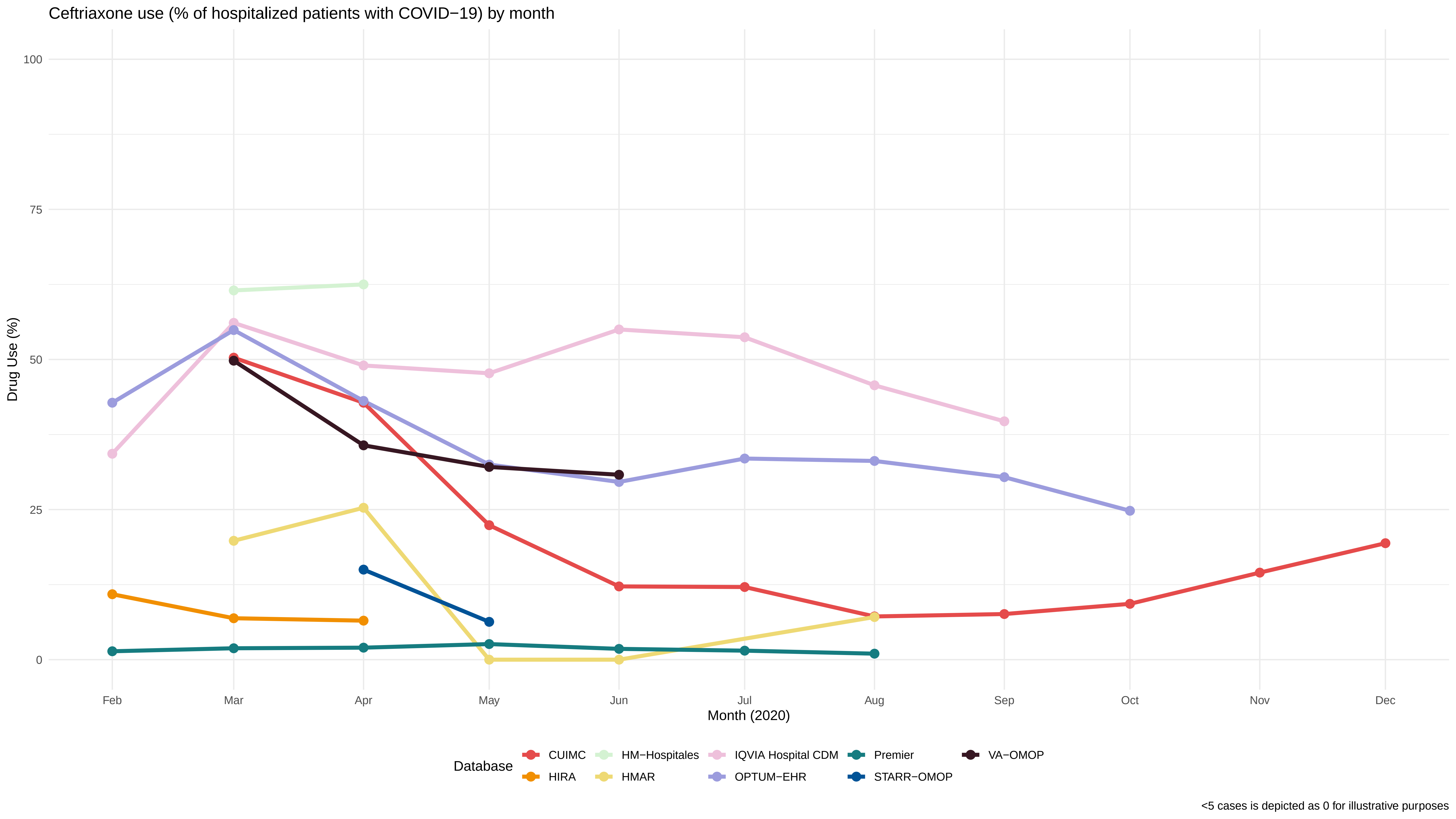

Ceftriaxone use (% of hospitalized patients with COVID−19) by month
Drug Use (%)
100
75
50
25
0
Feb
Mar
Apr
May
Jun
Jul
Aug
Sep
Oct
Nov
Dec
Month (2020)
Database
CUIMC
HIRA
HM−Hospitales
HMAR
IQVIA Hospital CDM
OPTUM−EHR
Premier
STARR−OMOP
VA−OMOP
<5 cases is depicted as 0 for illustrative purposes

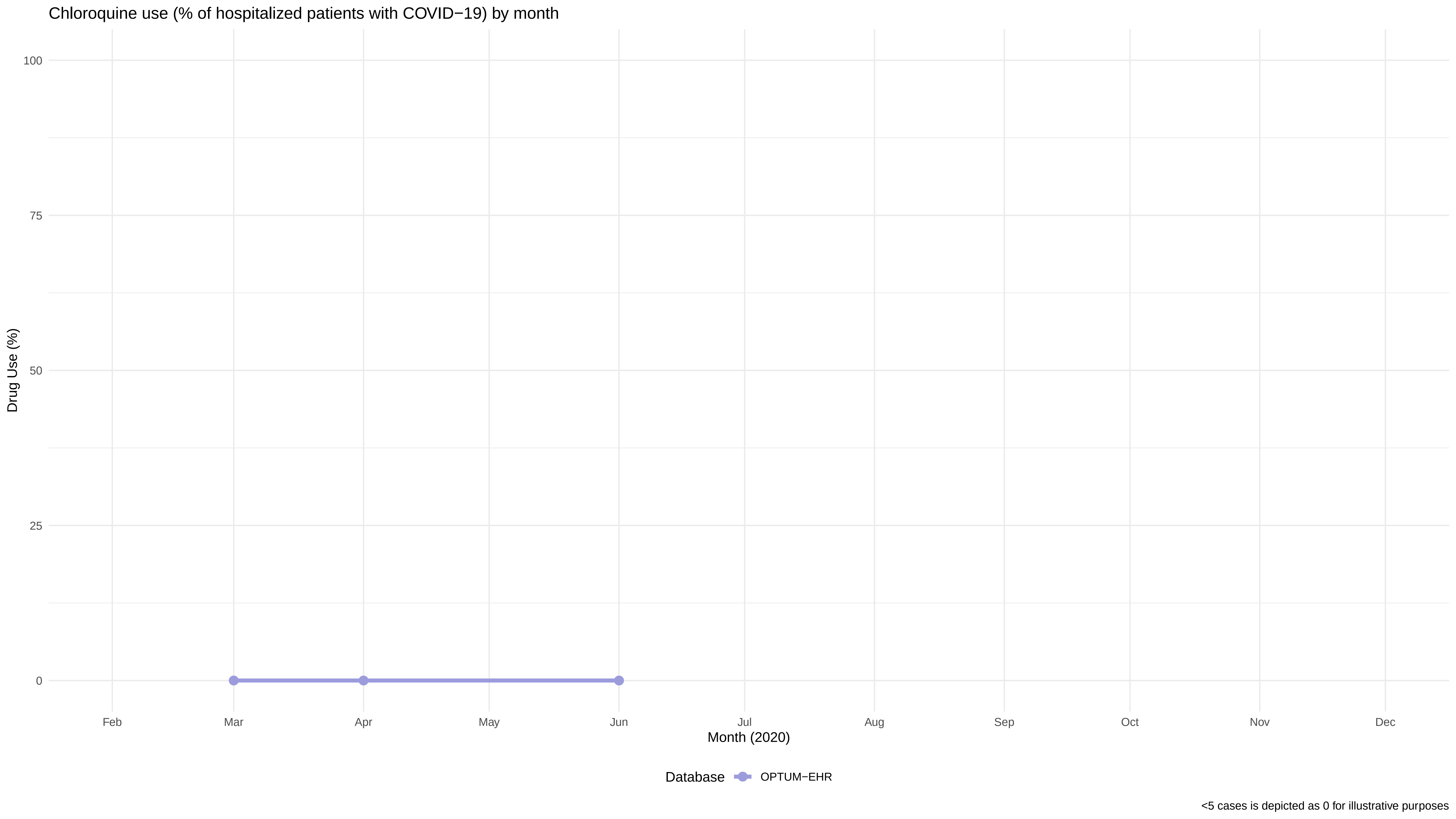
Chloroquine use (% of hospitalized patients with COVID−19) by month
100
75
50
25
0
Drug Use (%)
Feb
Mar
Apr
May
Jun
Jul
Aug
Sep
Oct
Nov
Dec
Month (2020)
Database
OPTUM−EHR
<5 cases is depicted as 0 for illustrative purposes

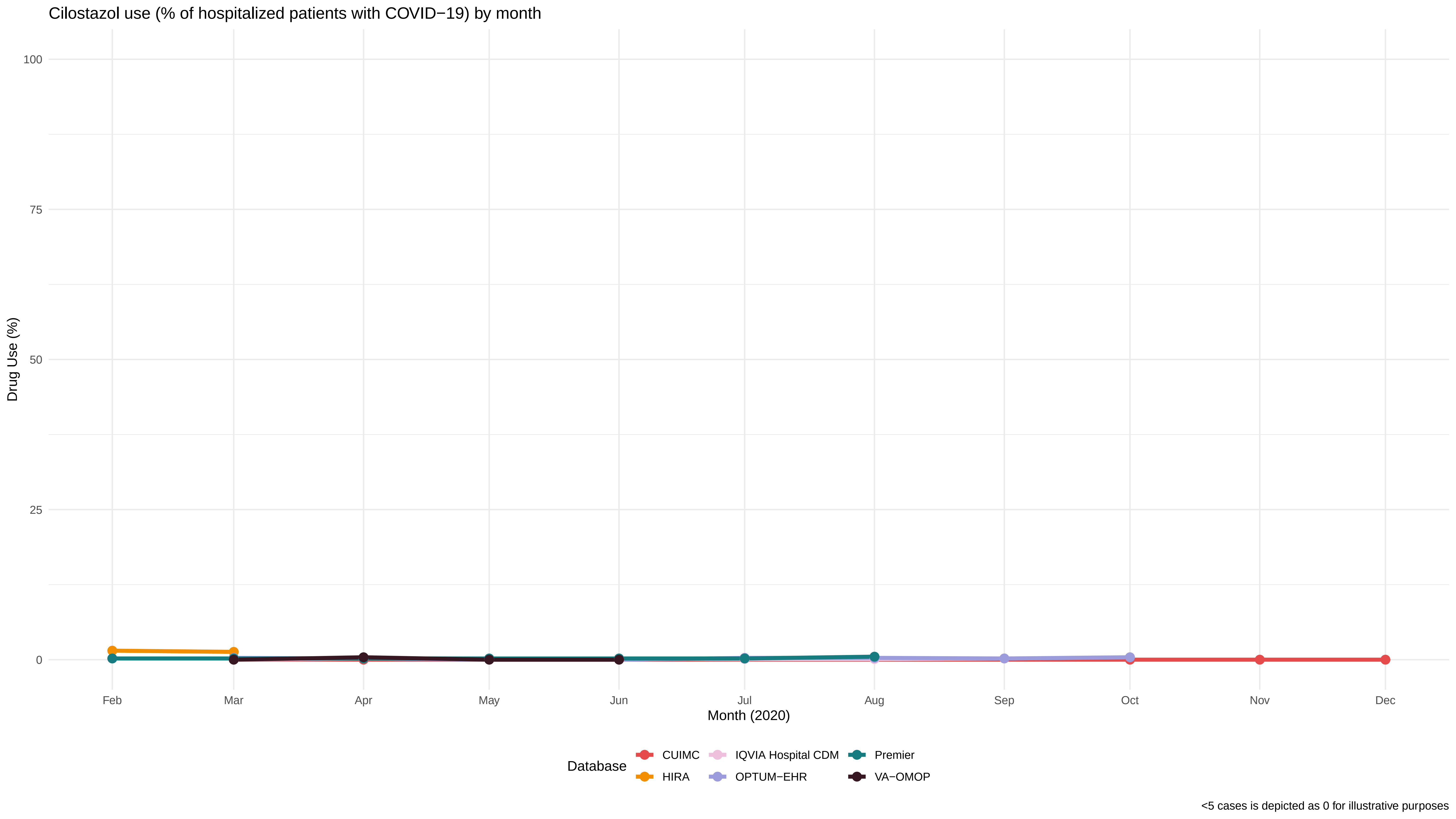

Cilostazol use (% of hospitalized patients with COVID−19) by month
Drug Use (%)
100
75
50
25
0
Feb
Mar
Apr
May
Jun
Jul
Aug
Sep
Oct
Nov
Dec
Month (2020)
Database
CUIMC
HIRA
IQVIA Hospital CDM
OPTUM−EHR
Premier
VA−OMOP
<5 cases is depicted as 0 for illustrative purposes

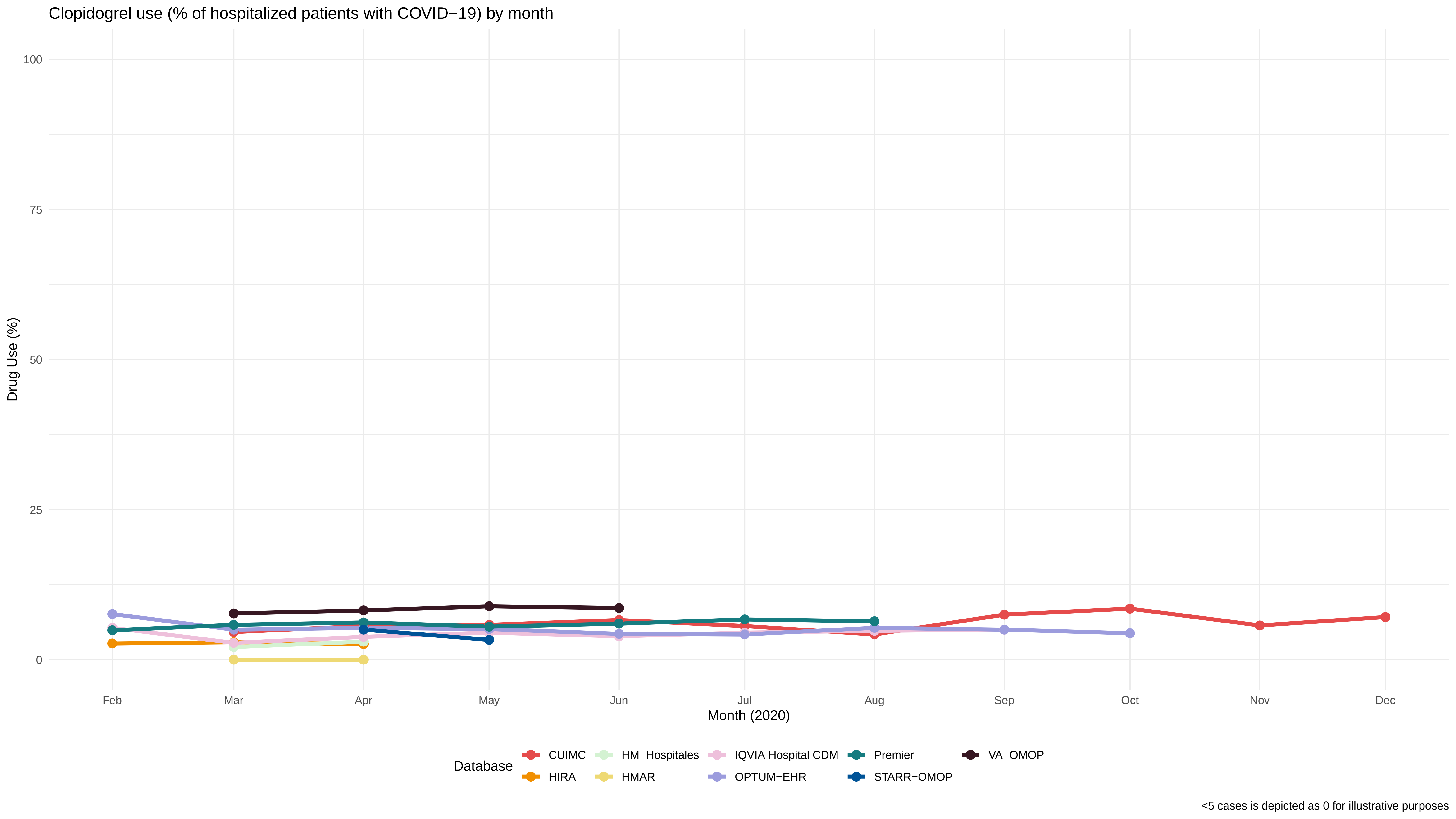
Clopidogrel use (% of hospitalized patients with COVID−19) by month
Drug Use (%)
100
75
50
25
0
Feb
Mar
Apr
May
Jun
Jul
Aug
Sep
Oct
Nov
Dec
Month (2020)
Database
CUIMC
HIRA
HM−Hospitales
HMAR
IQVIA Hospital CDM
OPTUM−EHR
Premier
STARR−OMOP
VA−OMOP
<5 cases is depicted as 0 for illustrative purposes

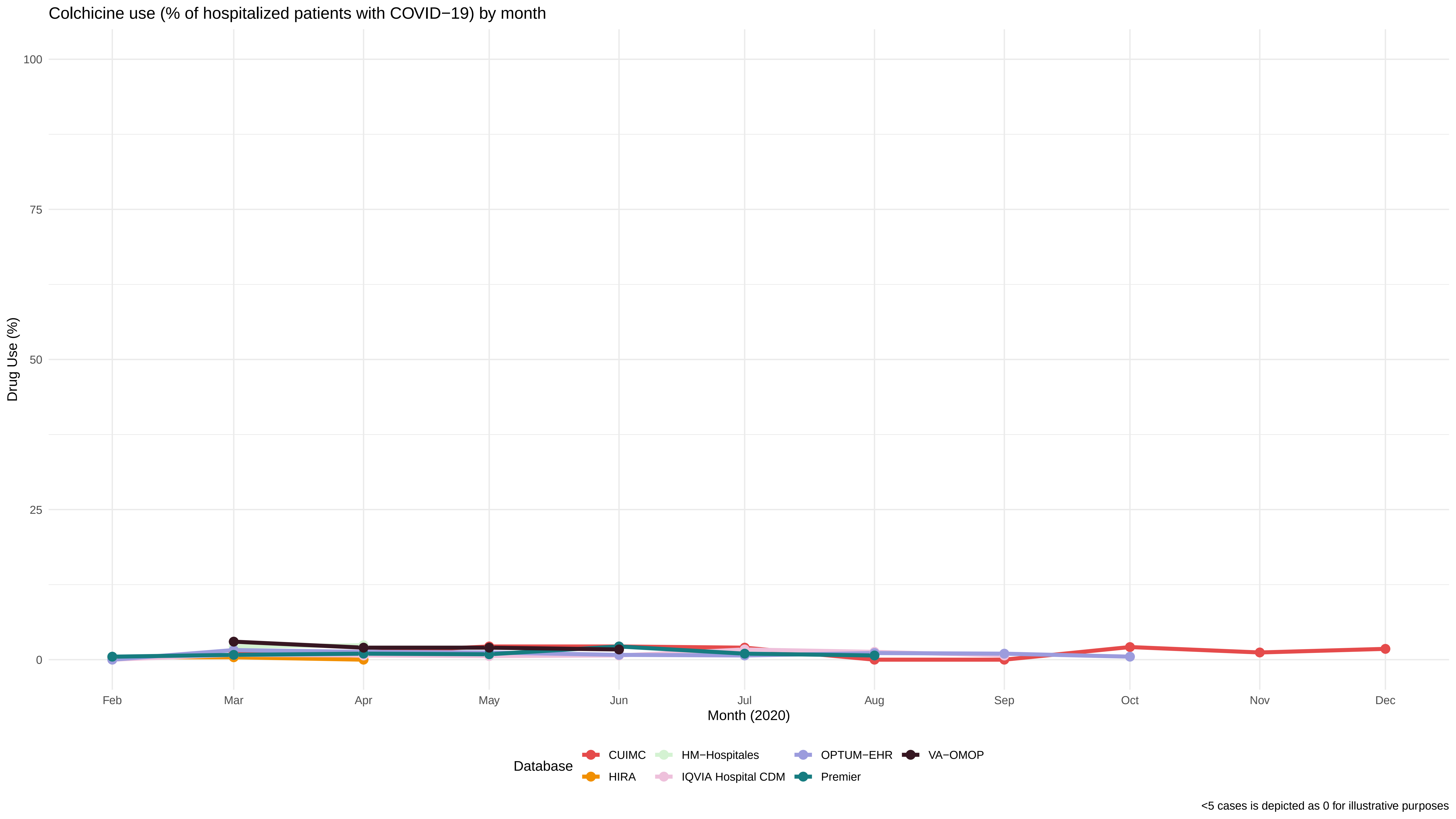

Colchicine use (% of hospitalized patients with COVID−19) by month
Drug Use (%)
100
75
50
25
0
Feb
Mar
Apr
May
Jun
Jul
Aug
Sep
Oct
Nov
Dec
Month (2020)
Database
CUIMC
HIRA
HM−Hospitales
IQVIA Hospital CDM
OPTUM−EHR
Premier
VA−OMOP
<5 cases is depicted as 0 for illustrative purposes

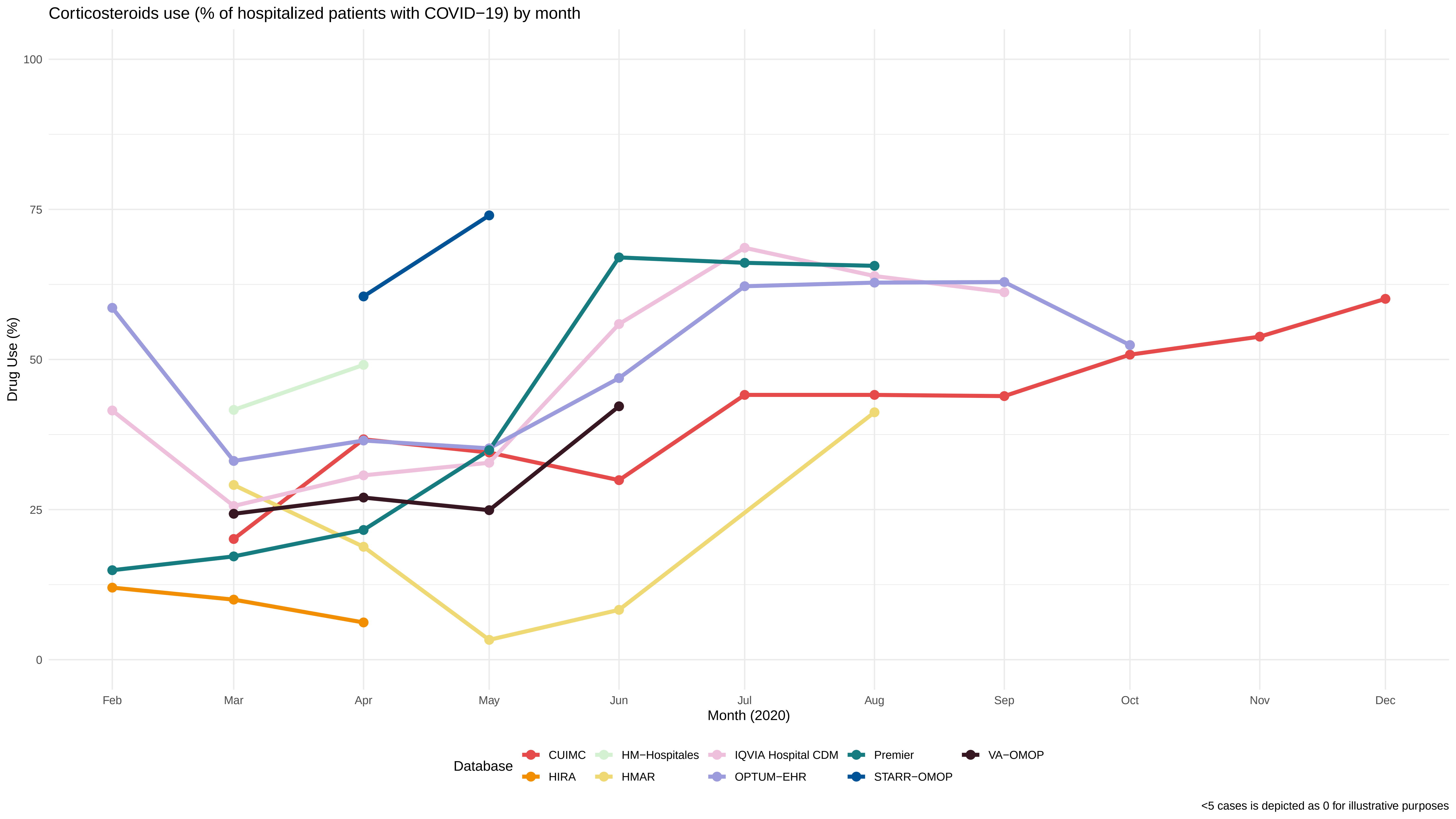

Corticosteroids use (% of hospitalized patients with COVID−19) by month
Drug Use (%)
100
75
50
25
0
Feb
Mar
Apr
May
Jun
Jul
Aug
Sep
Oct
Nov
Dec
Month (2020)
Database
CUIMC
HIRA
HM−Hospitales
HMAR
IQVIA Hospital CDM
OPTUM−EHR
Premier
STARR−OMOP
VA−OMOP
<5 cases is depicted as 0 for illustrative purposes

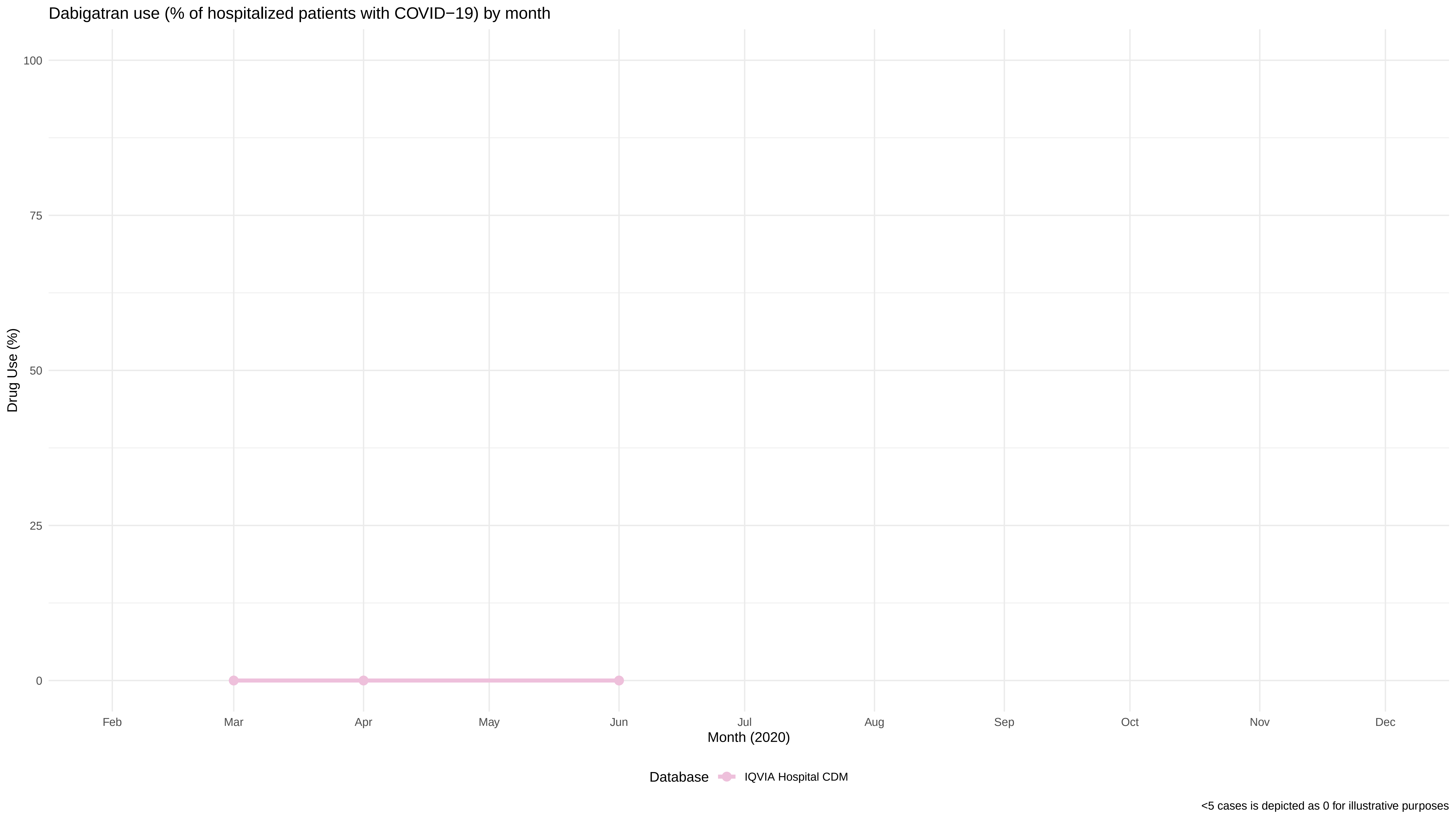
Dabigatran use (% of hospitalized patients with COVID−19) by month
100
75
50
25
0
Drug Use (%)
Feb
Mar
Apr
May
Jun
Jul
Aug
Sep
Oct
Nov
Dec
Month (2020)
Database
IQVIA Hospital CDM
<5 cases is depicted as 0 for illustrative purposes

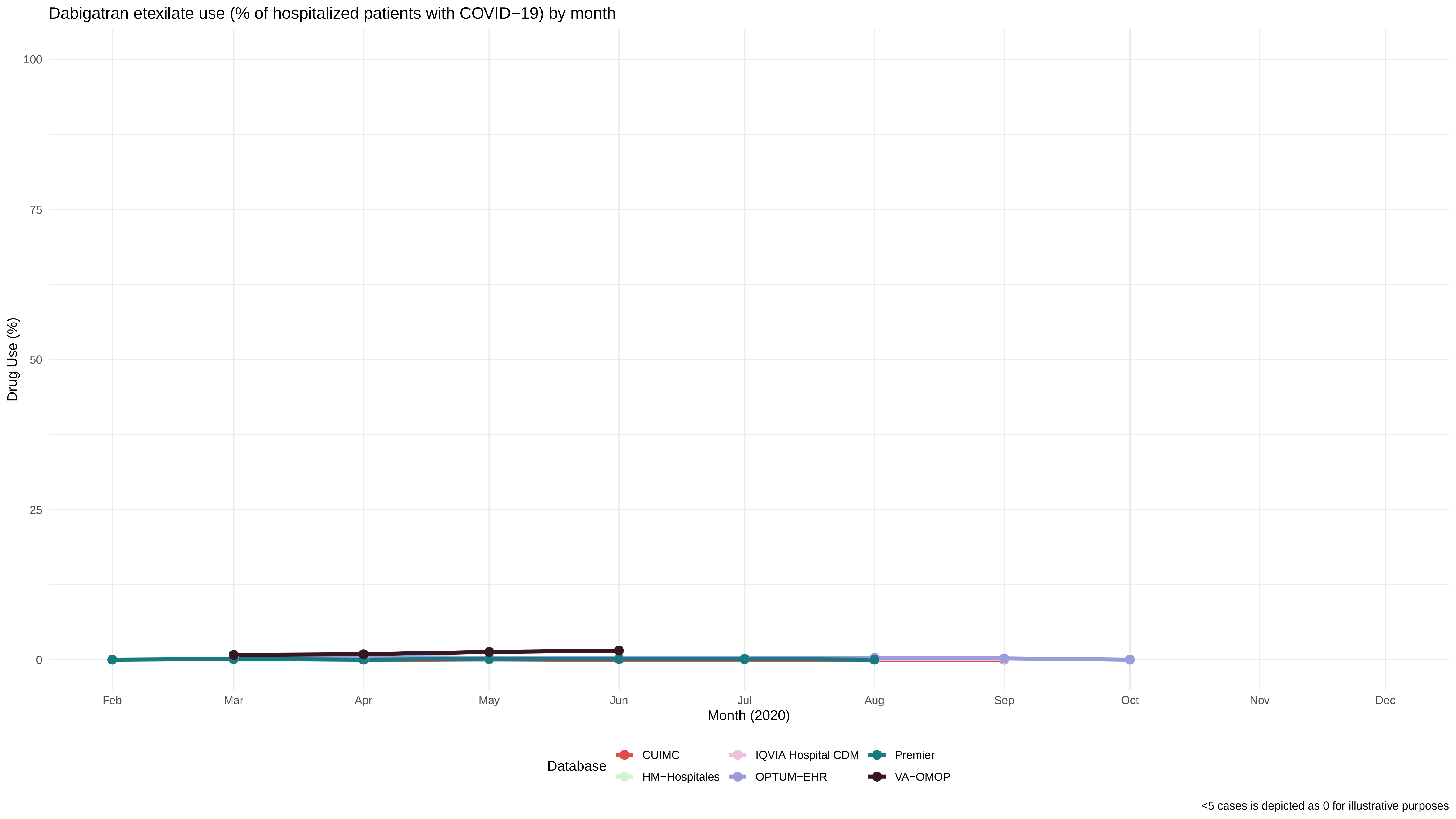

Dabigatran etexilate use (% of hospitalized patients with COVID−19) by month
Drug Use (%)
100
75
50
25
0
Feb
Mar
Apr
May
Jun
Jul
Aug
Sep
Oct
Nov
Dec
Month (2020)
Database
CUIMC
HM−Hospitales
IQVIA Hospital CDM
OPTUM−EHR
Premier
VA−OMOP
<5 cases is depicted as 0 for illustrative purposes

Dalteparin use (% of hospitalized patients with COVID−19) by month

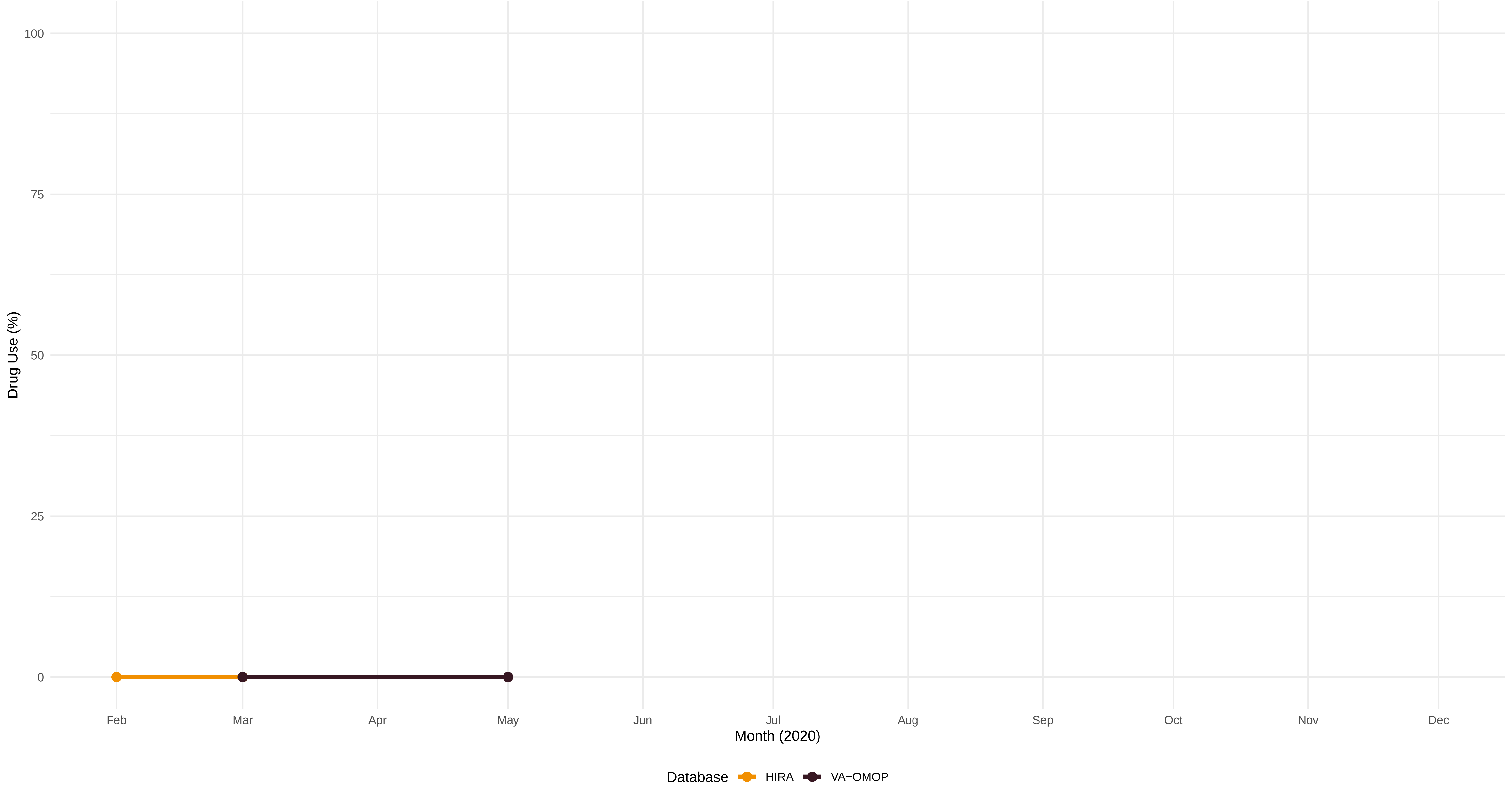

<5 cases is depicted as 0 for illustrative purposes

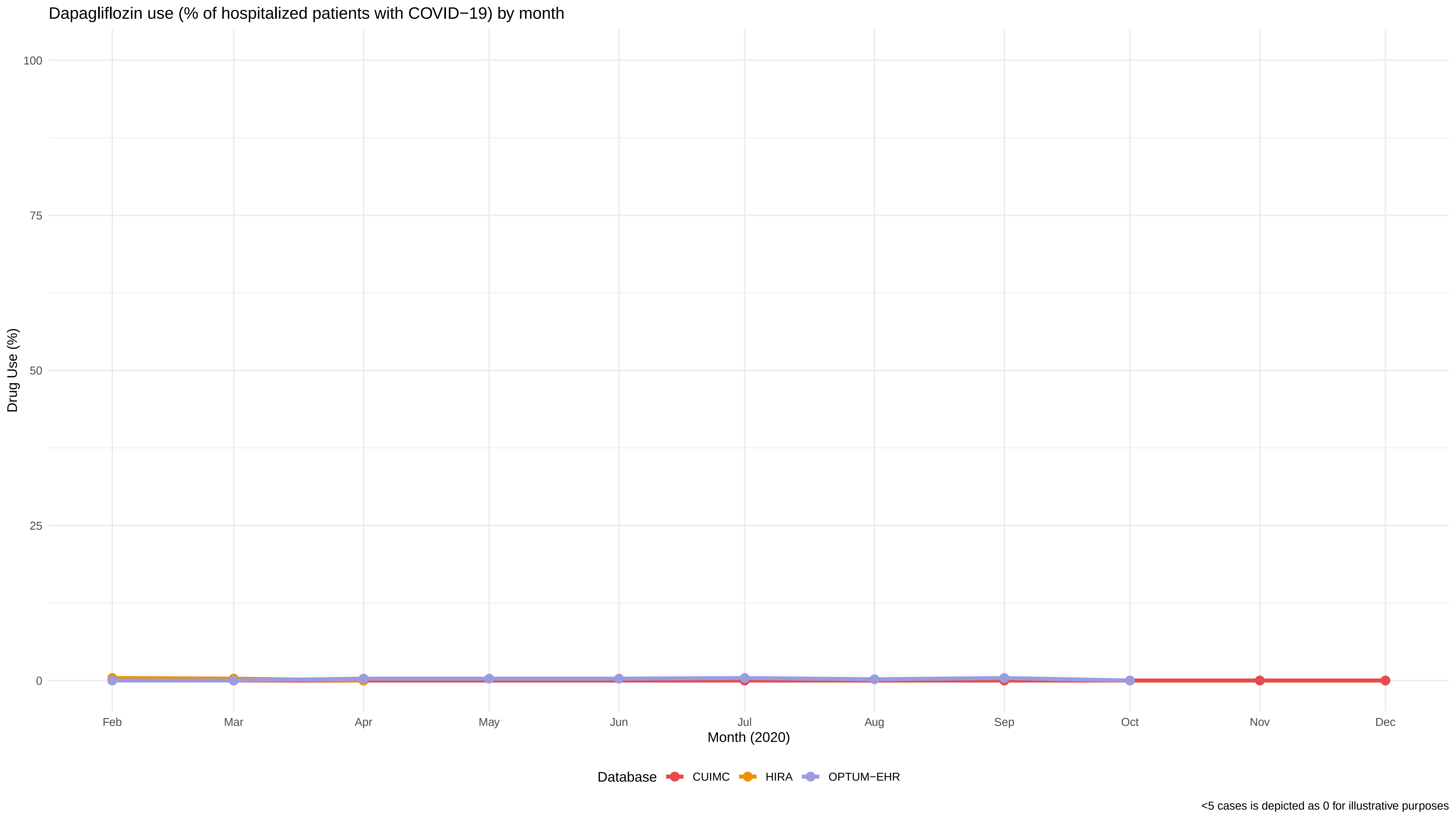

Dapagliflozin use (% of hospitalized patients with COVID−19) by month
Drug Use (%)
100
75
50
25
0
Feb
Mar
Apr
May
Jun
Jul
Aug
Sep
Oct
Nov
Dec
Month (2020)
Database
CUIMC
HIRA
OPTUM−EHR
<5 cases is depicted as 0 for illustrative purposes

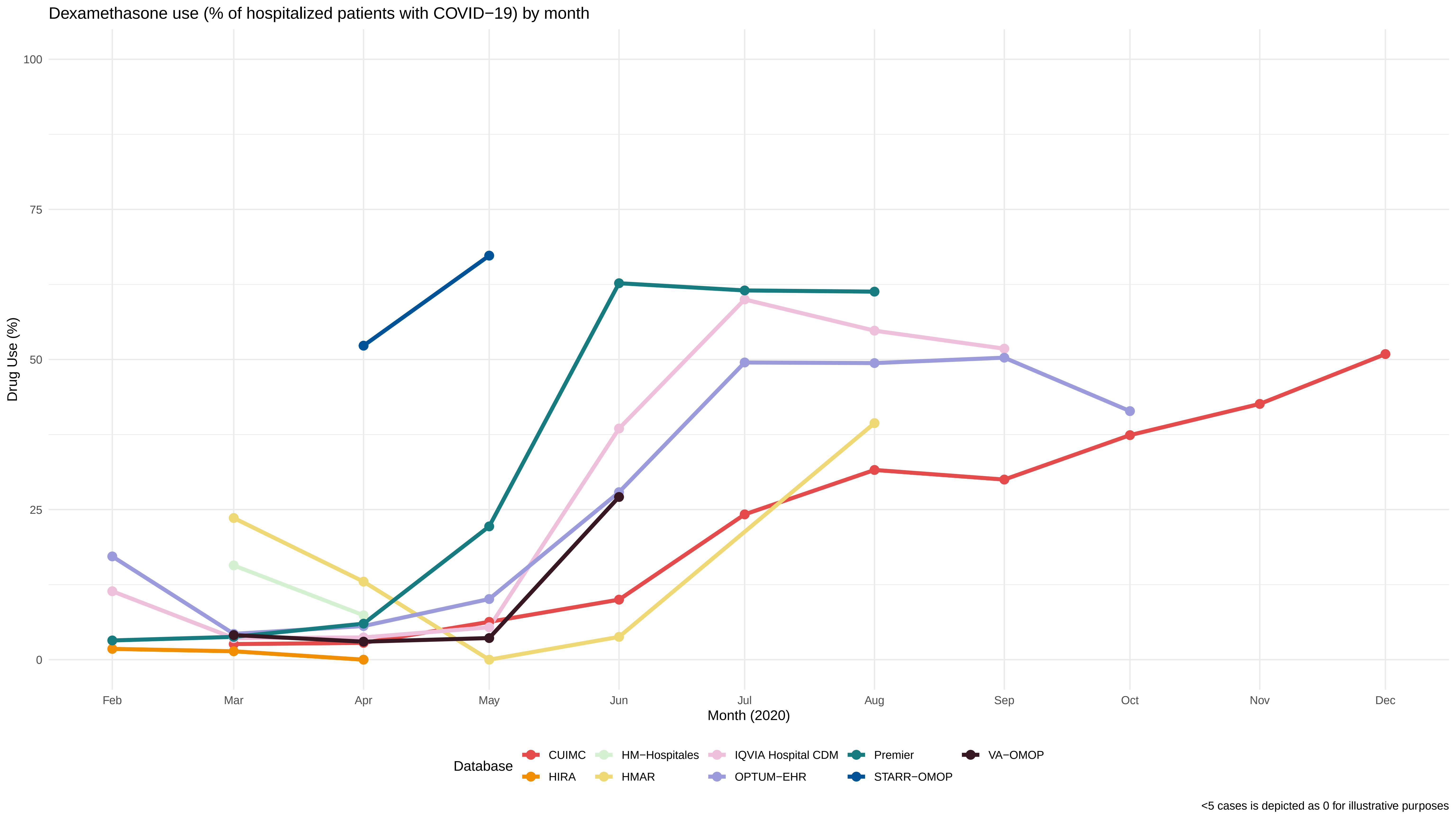

Dexamethasone use (% of hospitalized patients with COVID−19) by month
Drug Use (%)
100
75
50
25
0
Feb
Mar
Apr
May
Jun
Jul
Aug
Sep
Oct
Nov
Dec
Month (2020)
Database
CUIMC
HIRA
HM−Hospitales
HMAR
IQVIA Hospital CDM
OPTUM−EHR
Premier
STARR−OMOP
VA−OMOP
<5 cases is depicted as 0 for illustrative purposes

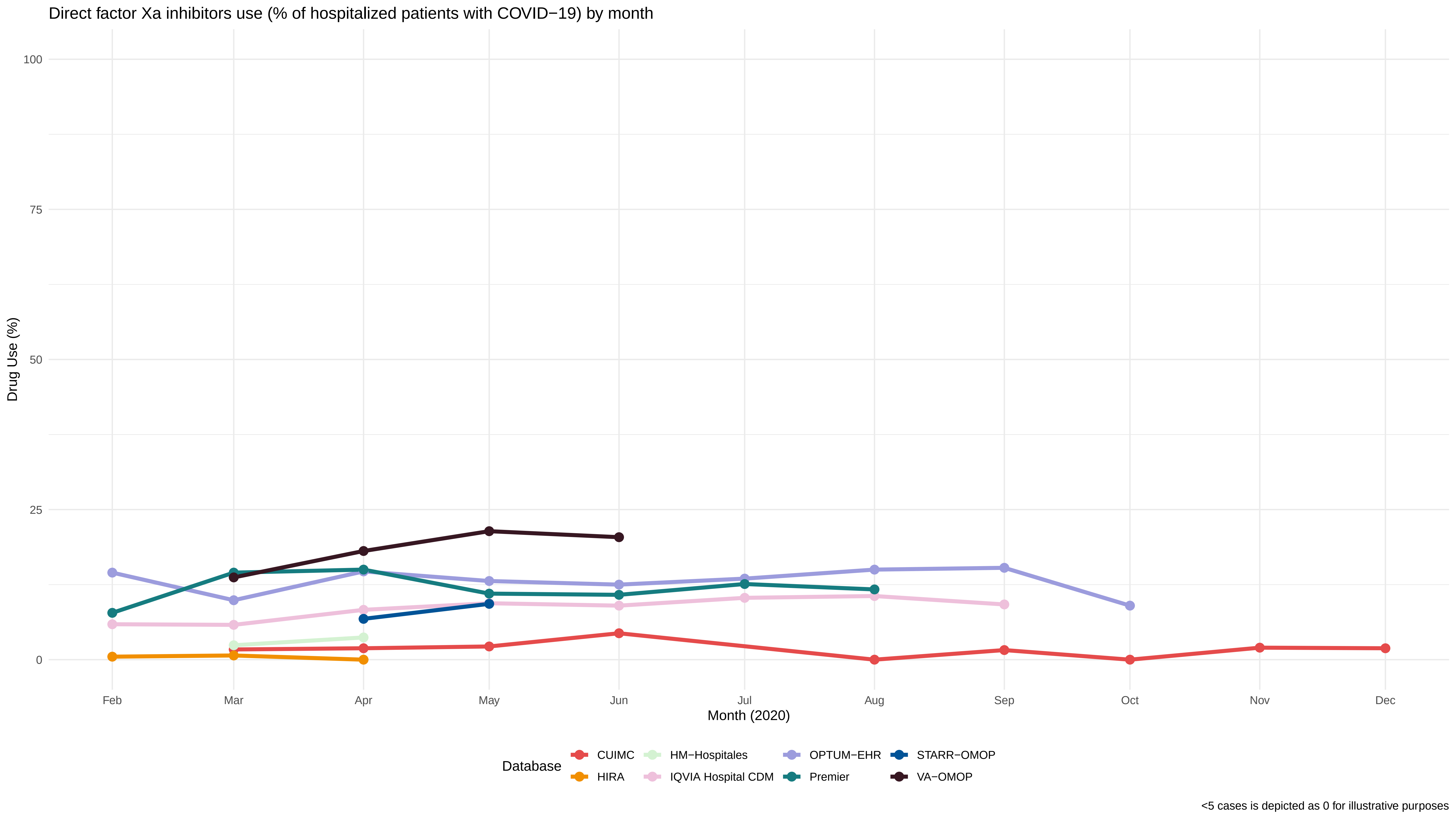
Direct factor Xa inhibitors use (% of hospitalized patients with COVID−19) by month
Drug Use (%)
100
75
50
25
0
Feb
Mar
Apr
May
Jun
Jul
Aug
Sep
Oct
Nov
Dec
Month (2020)
Database
CUIMC
HIRA
HM−Hospitales
IQVIA Hospital CDM
OPTUM−EHR
Premier
STARR−OMOP
VA−OMOP
<5 cases is depicted as 0 for illustrative purposes

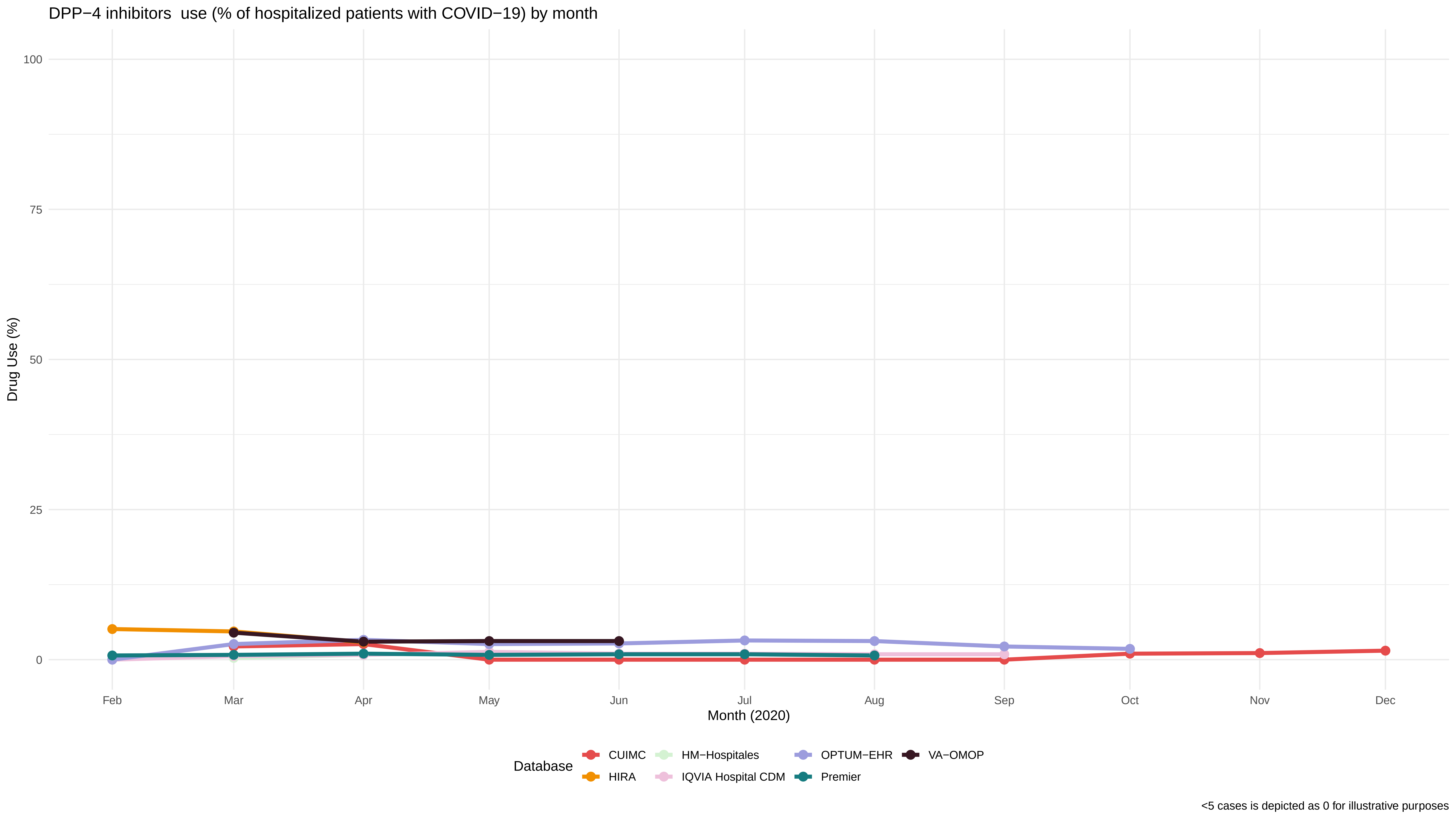
DPP−4 inhibitors use (% of hospitalized patients with COVID−19) by month
Drug Use (%)
100
75
50
25
0
Feb
Mar
Apr
May
Jun
Jul
Aug
Sep
Oct
Nov
Dec
Month (2020)
Database
CUIMC
HIRA
HM−Hospitales
IQVIA Hospital CDM
OPTUM−EHR
Premier
VA−OMOP
<5 cases is depicted as 0 for illustrative purposes

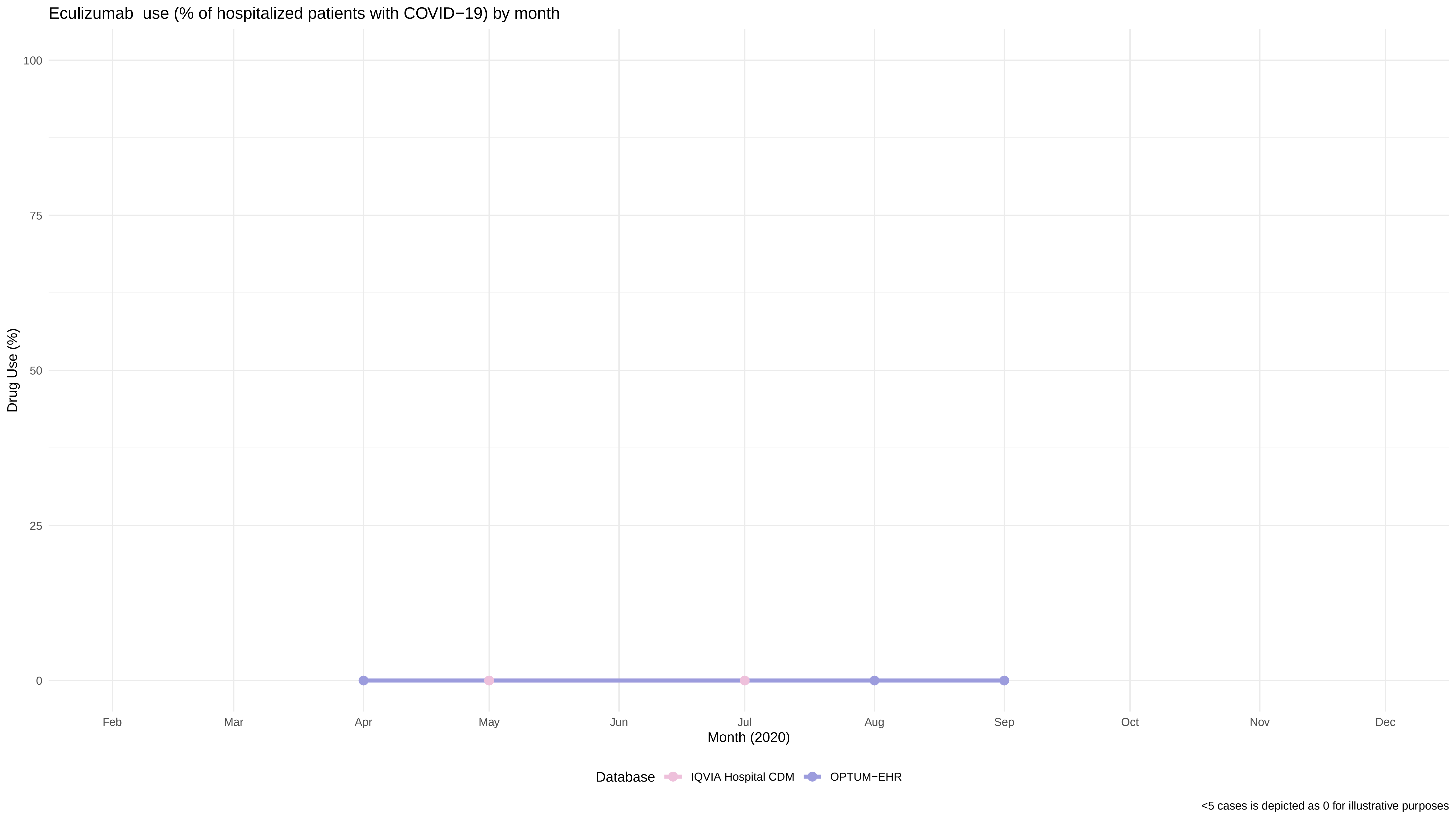
Eculizumab use (% of hospitalized patients with COVID−19) by month
100
75
50
25
0
Drug Use (%)
Feb
Mar
Apr
May
Jun
Jul
Aug
Sep
Oct
Nov
Dec
Month (2020)
Database
IQVIA Hospital CDM
OPTUM−EHR
<5 cases is depicted as 0 for illustrative purposes

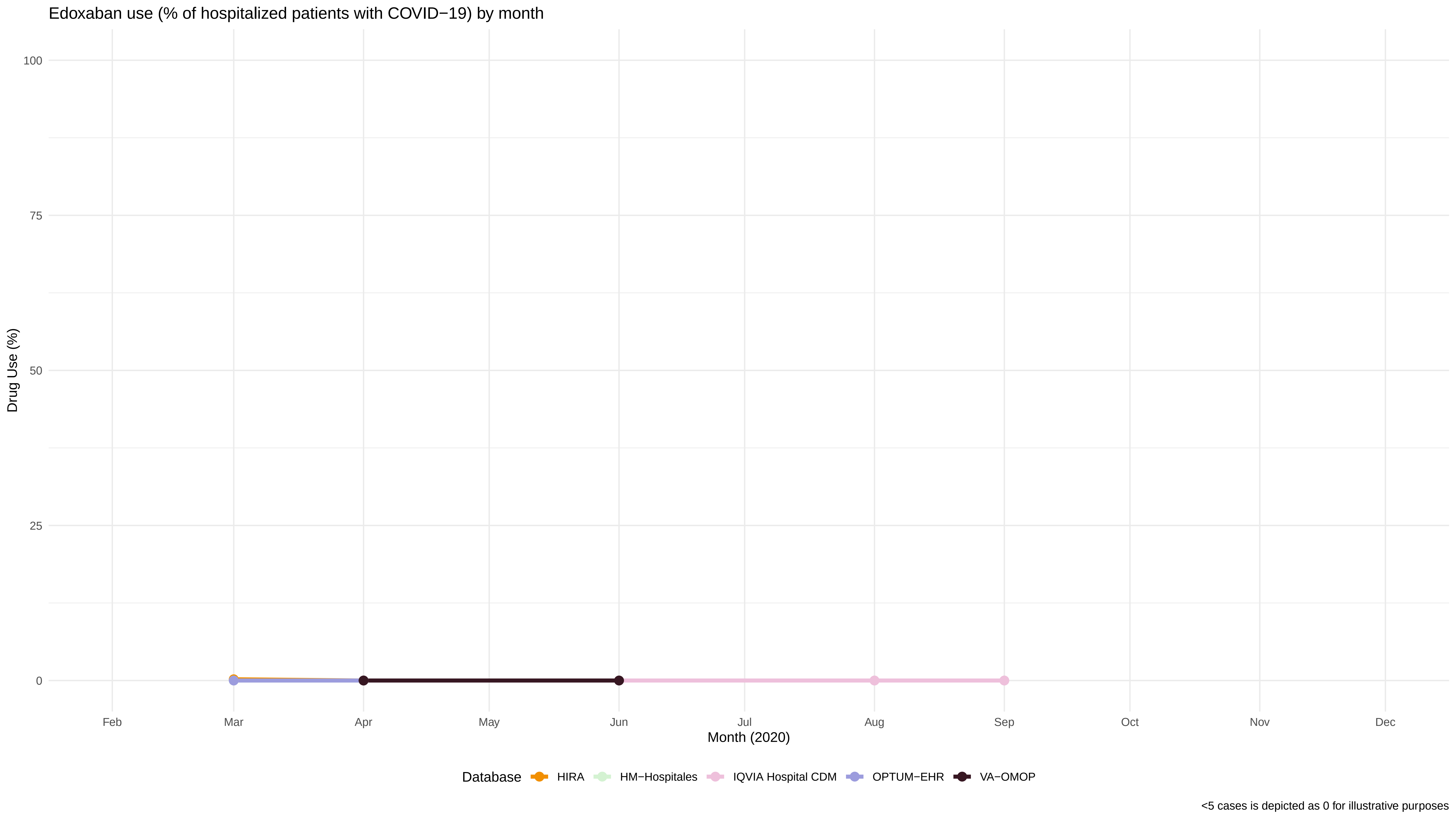
Edoxaban use (% of hospitalized patients with COVID−19) by month
Drug Use (%)
100
75
50
25
0
Feb
Mar
Apr
May
Jun
Jul
Aug
Sep
Oct
Nov
Dec
Month (2020)
Database
HIRA
HM−Hospitales
IQVIA Hospital CDM
OPTUM−EHR
VA−OMOP
<5 cases is depicted as 0 for illustrative purposes

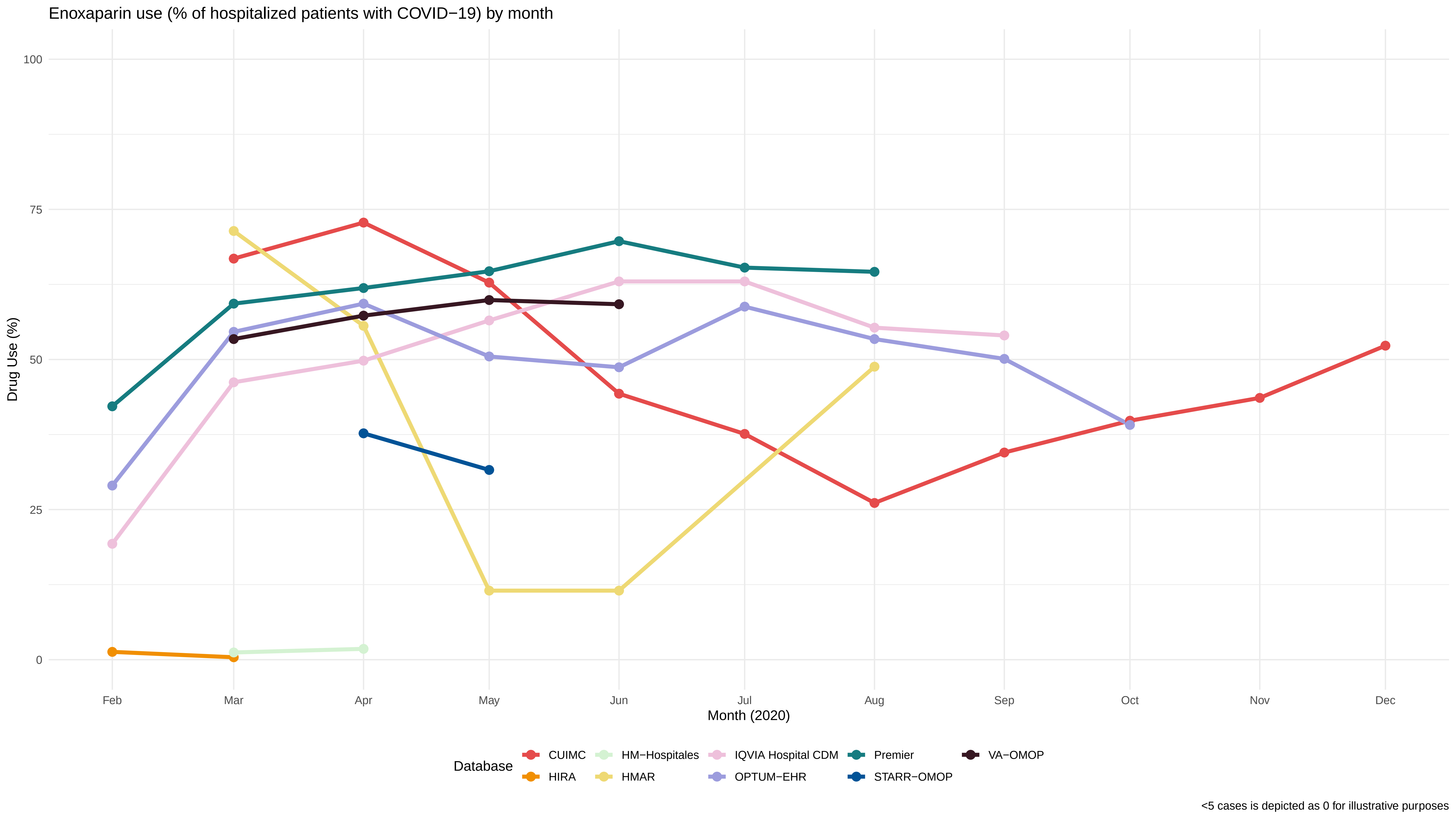
Enoxaparin use (% of hospitalized patients with COVID−19) by month
Drug Use (%)
100
75
50
25
0
Feb
Mar
Apr
May
Jun
Jul
Aug
Sep
Oct
Nov
Dec
Month (2020)
Database
CUIMC
HIRA
HM−Hospitales
HMAR
IQVIA Hospital CDM
OPTUM−EHR
Premier
STARR−OMOP
VA−OMOP
<5 cases is depicted as 0 for illustrative purposes

Etanercept use (% of hospitalized patients with COVID−19) by month

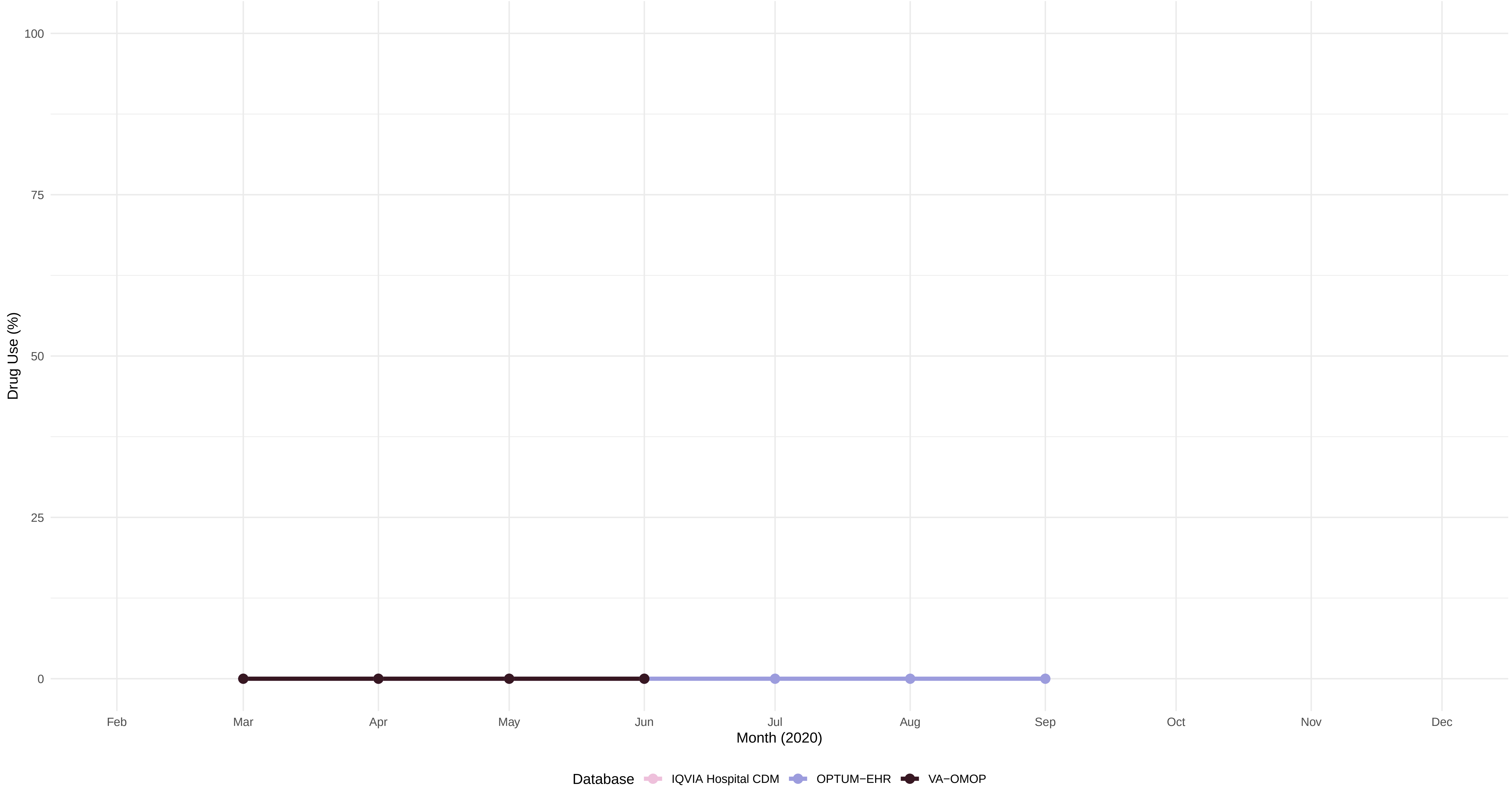

<5 cases is depicted as 0 for illustrative purposes

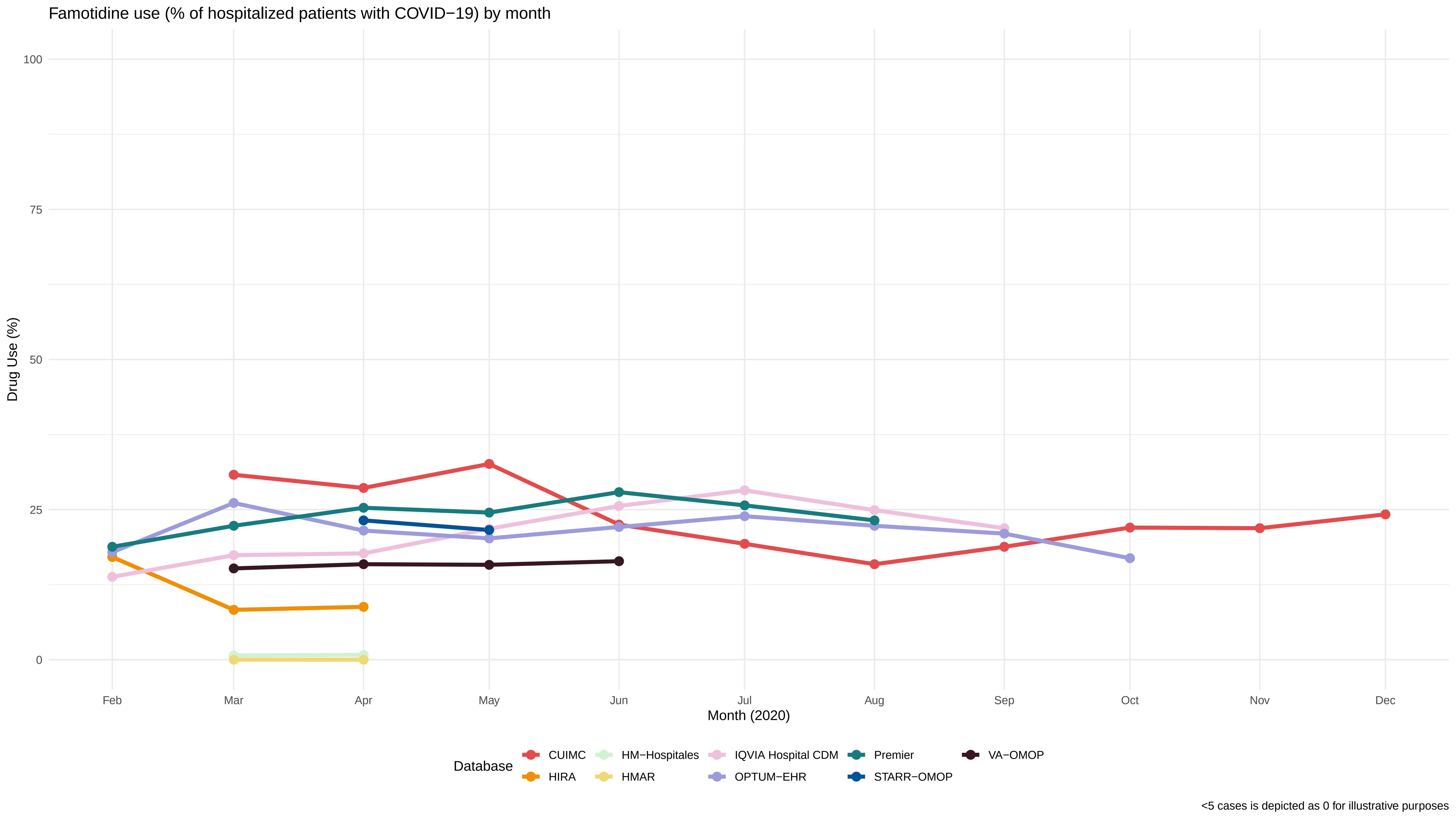

Famotidine use (% of hospitalized patients with COVID−19) by month
Drug Use (%)
100
75
50
25
0
Feb
Mar
Apr
May
Jun
Jul
Aug
Sep
Oct
Nov
Dec
Month (2020)
Database
CUIMC
HIRA
HM−Hospitales
HMAR
IQVIA Hospital CDM
OPTUM−EHR
Premier
STARR−OMOP
VA−OMOP
<5 cases is depicted as 0 for illustrative purposes

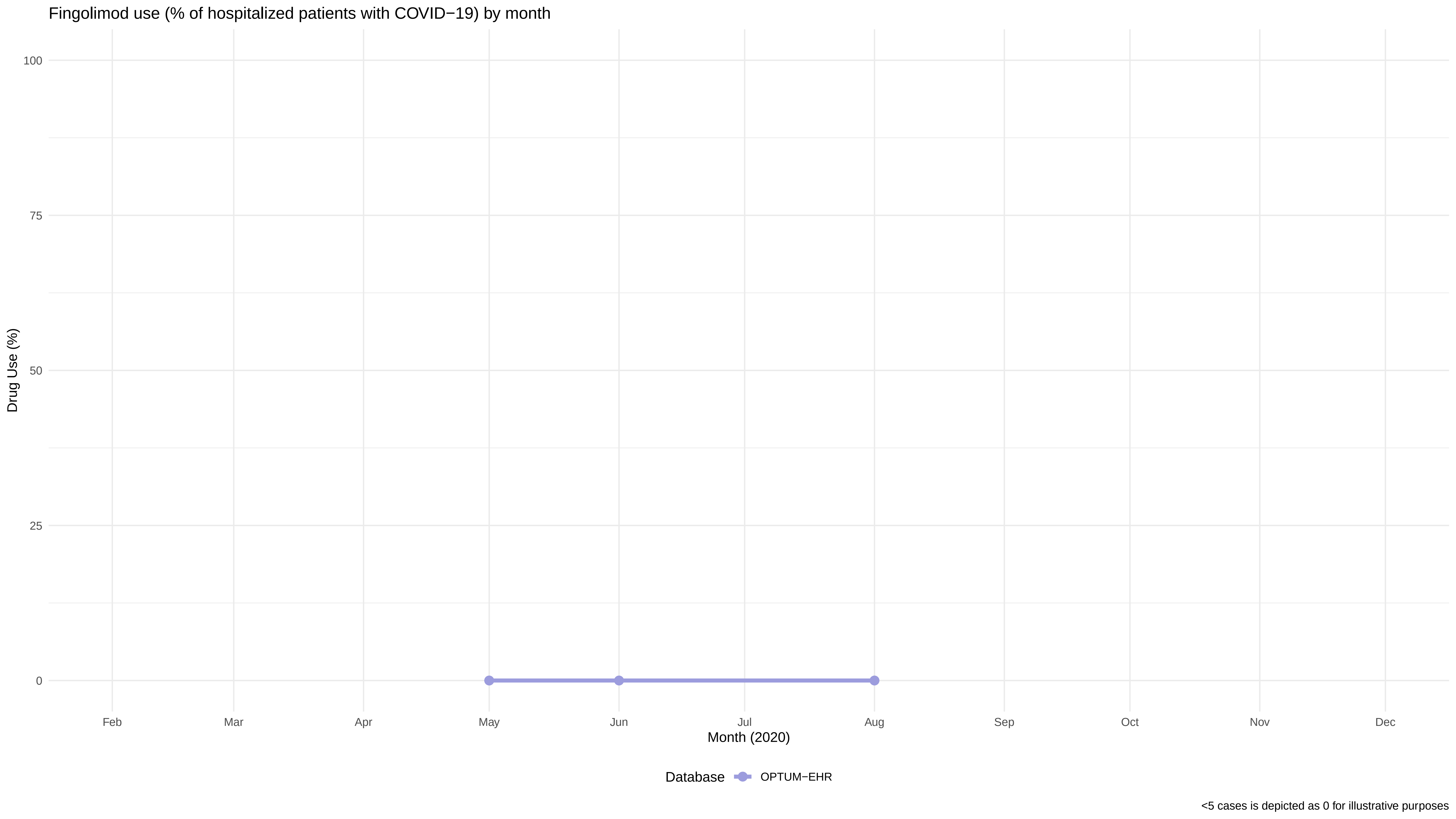
Fingolimod use (% of hospitalized patients with COVID−19) by month
100
75
50
25
0
Drug Use (%)
Feb
Mar
Apr
May
Jun
Jul
Aug
Sep
Oct
Nov
Dec
Month (2020)
Database
OPTUM−EHR
<5 cases is depicted as 0 for illustrative purposes

Fluoroquinolones use (% of hospitalized patients with COVID−19) by month

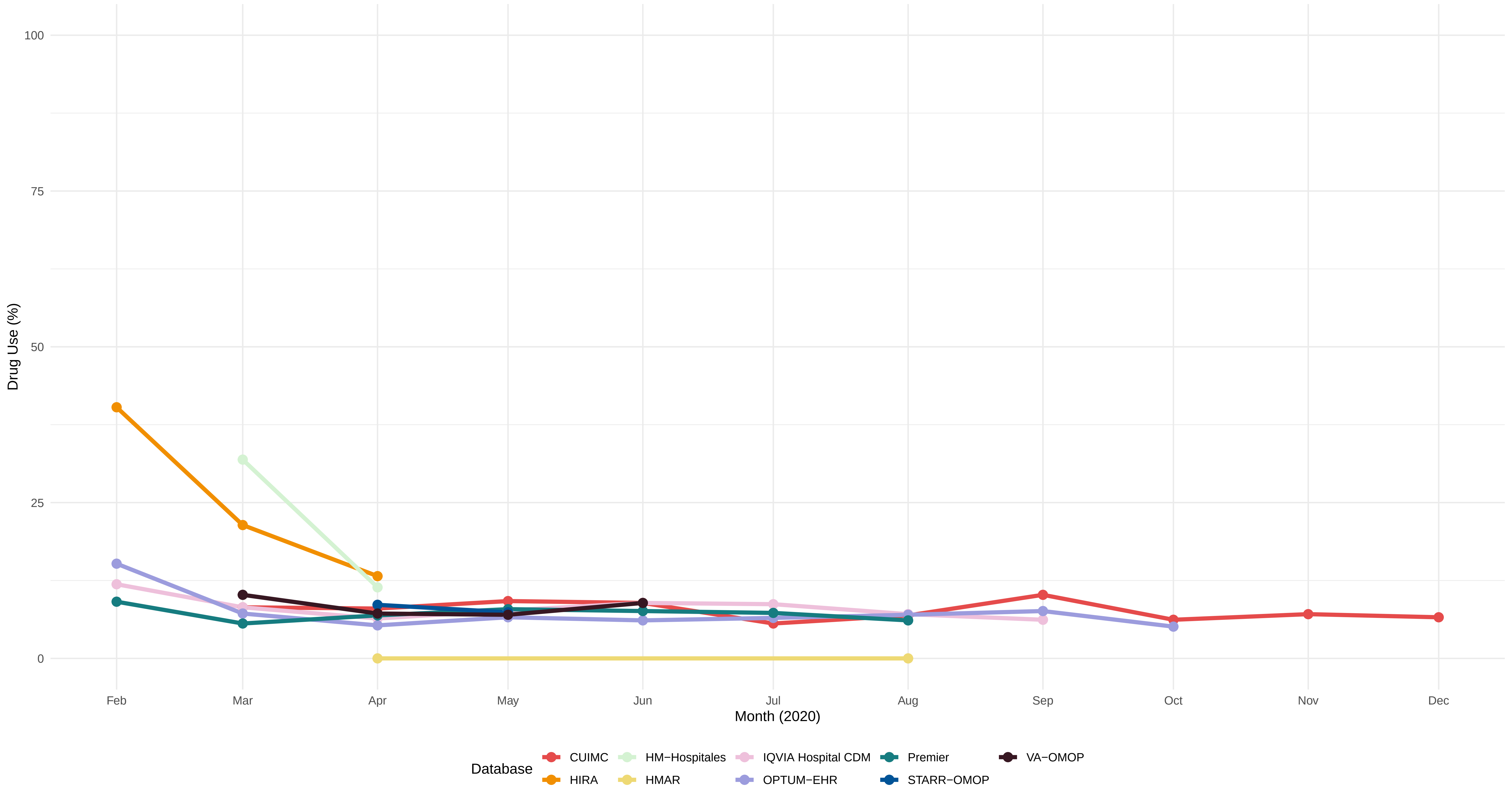

<5 cases is depicted as 0 for illustrative purposes

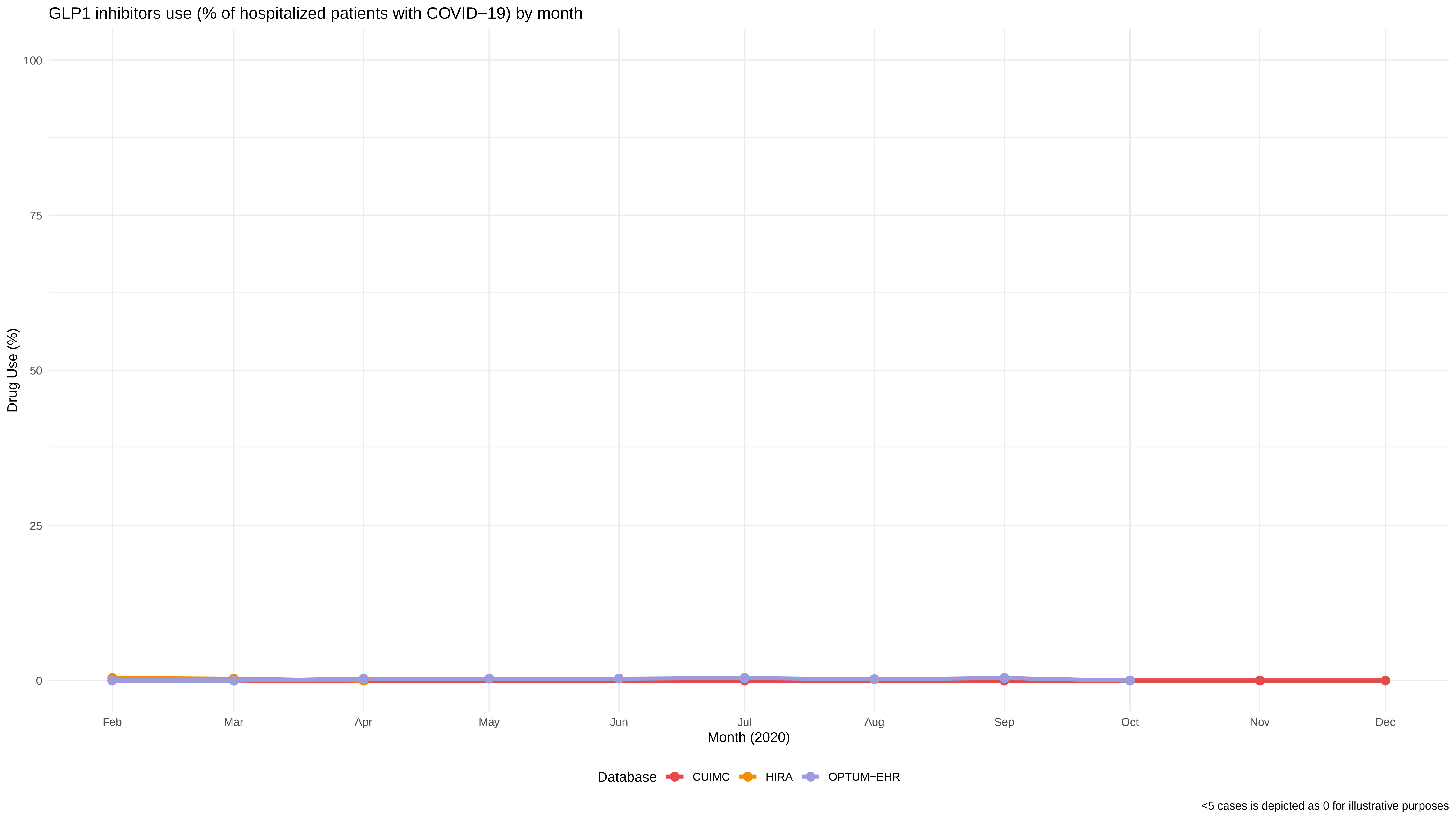

GLP1 inhibitors use (% of hospitalized patients with COVID−19) by month
Drug Use (%)
100
75
50
25
0
Feb
Mar
Apr
May
Jun
Jul
Aug
Sep
Oct
Nov
Dec
Month (2020)
Database
CUIMC
HIRA
OPTUM−EHR
<5 cases is depicted as 0 for illustrative purposes

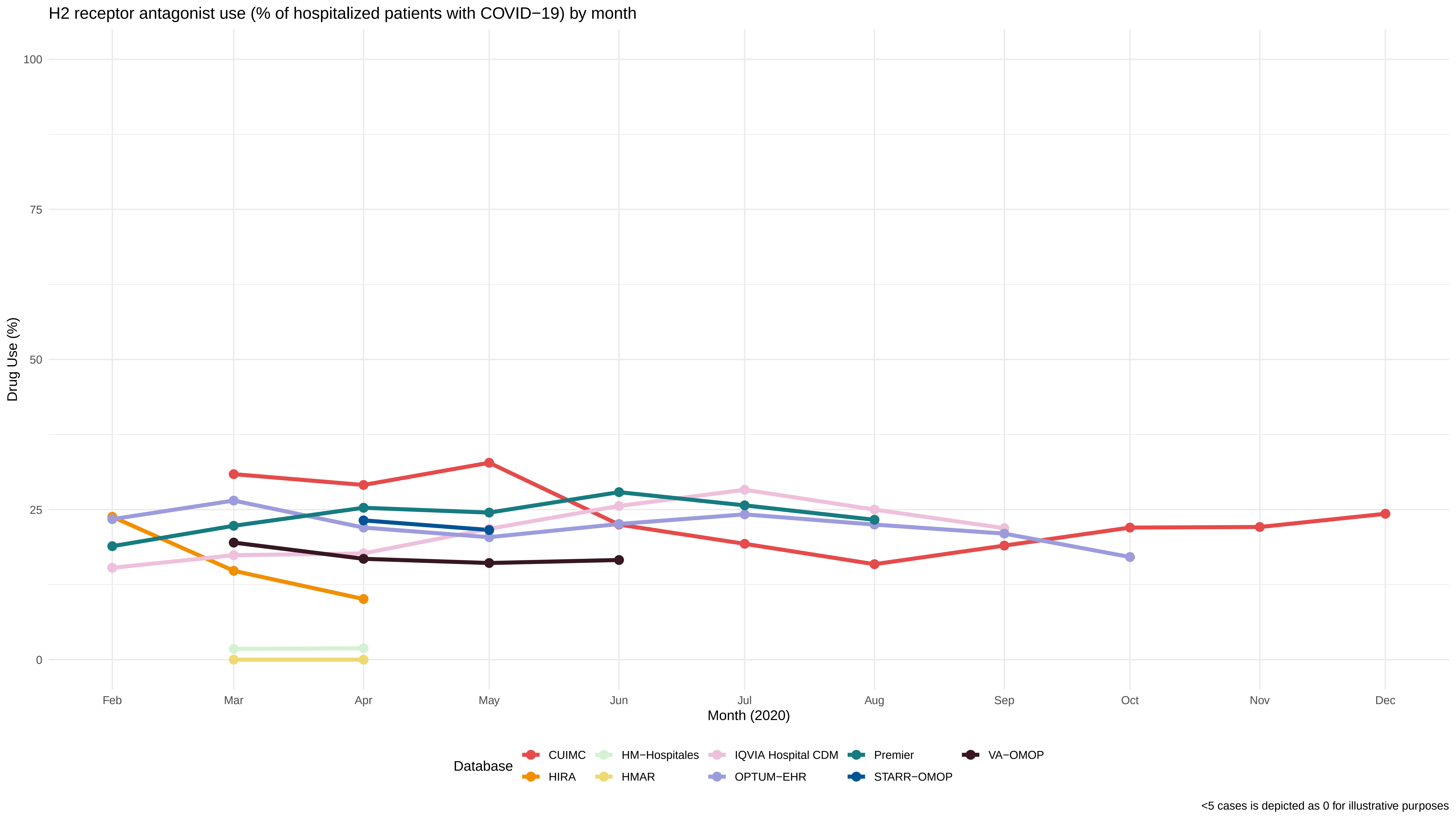

H2 receptor antagonist use (% of hospitalized patients with COVID−19) by month
Drug Use (%)
100
75
50
25
0
Feb
Mar
Apr
May
Jun
Jul
Aug
Sep
Oct
Nov
Dec
Month (2020)
Database
CUIMC
HIRA
HM−Hospitales
HMAR
IQVIA Hospital CDM
OPTUM−EHR
Premier
STARR−OMOP
VA−OMOP
<5 cases is depicted as 0 for illustrative purposes

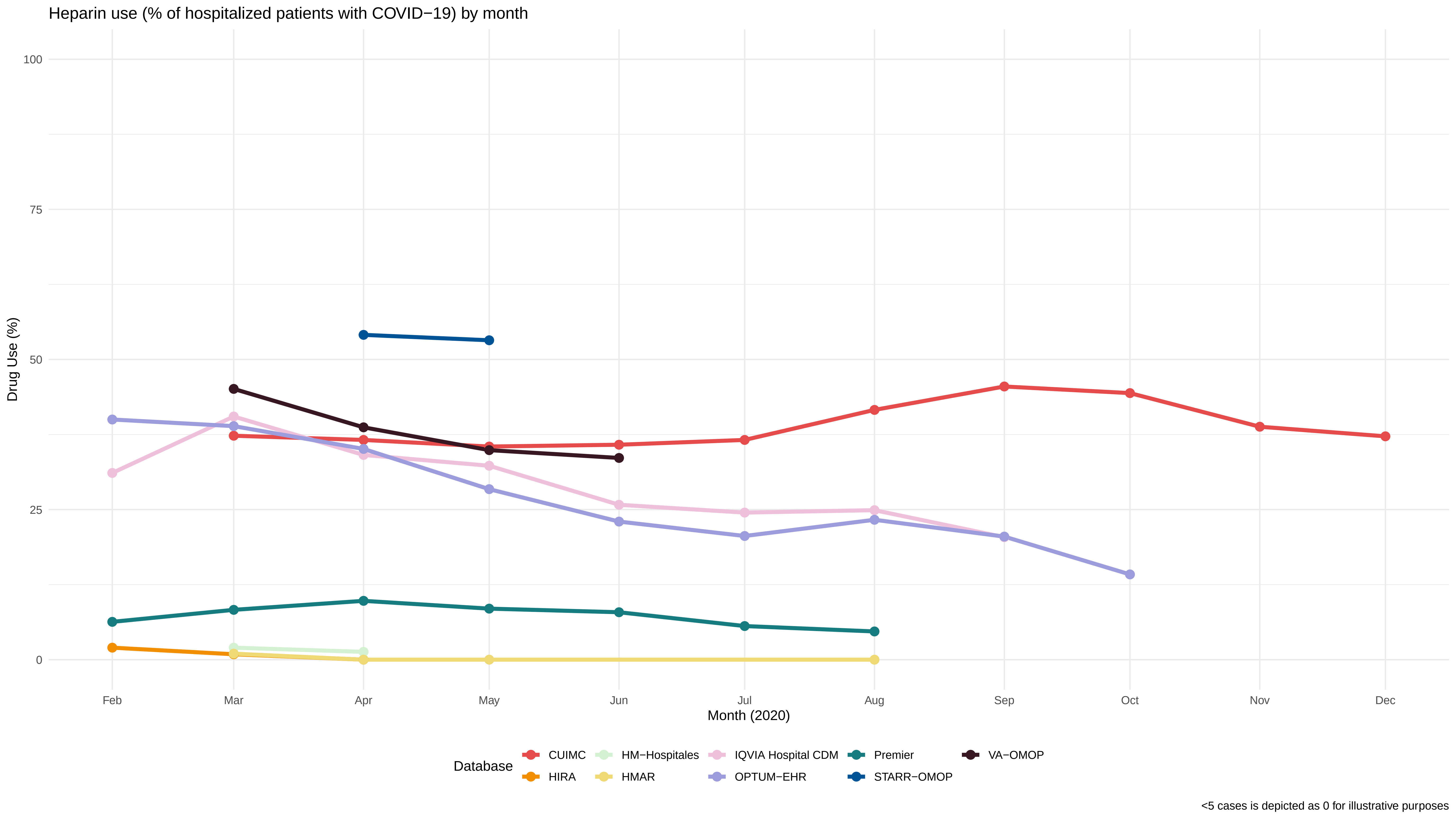

Heparin use (% of hospitalized patients with COVID−19) by month
Drug Use (%)
100
75
50
25
0
Feb
Mar
Apr
May
Jun
Jul
Aug
Sep
Oct
Nov
Dec
Month (2020)
Database
CUIMC
HIRA
HM−Hospitales
HMAR
IQVIA Hospital CDM
OPTUM−EHR
Premier
STARR−OMOP
VA−OMOP
<5 cases is depicted as 0 for illustrative purposes

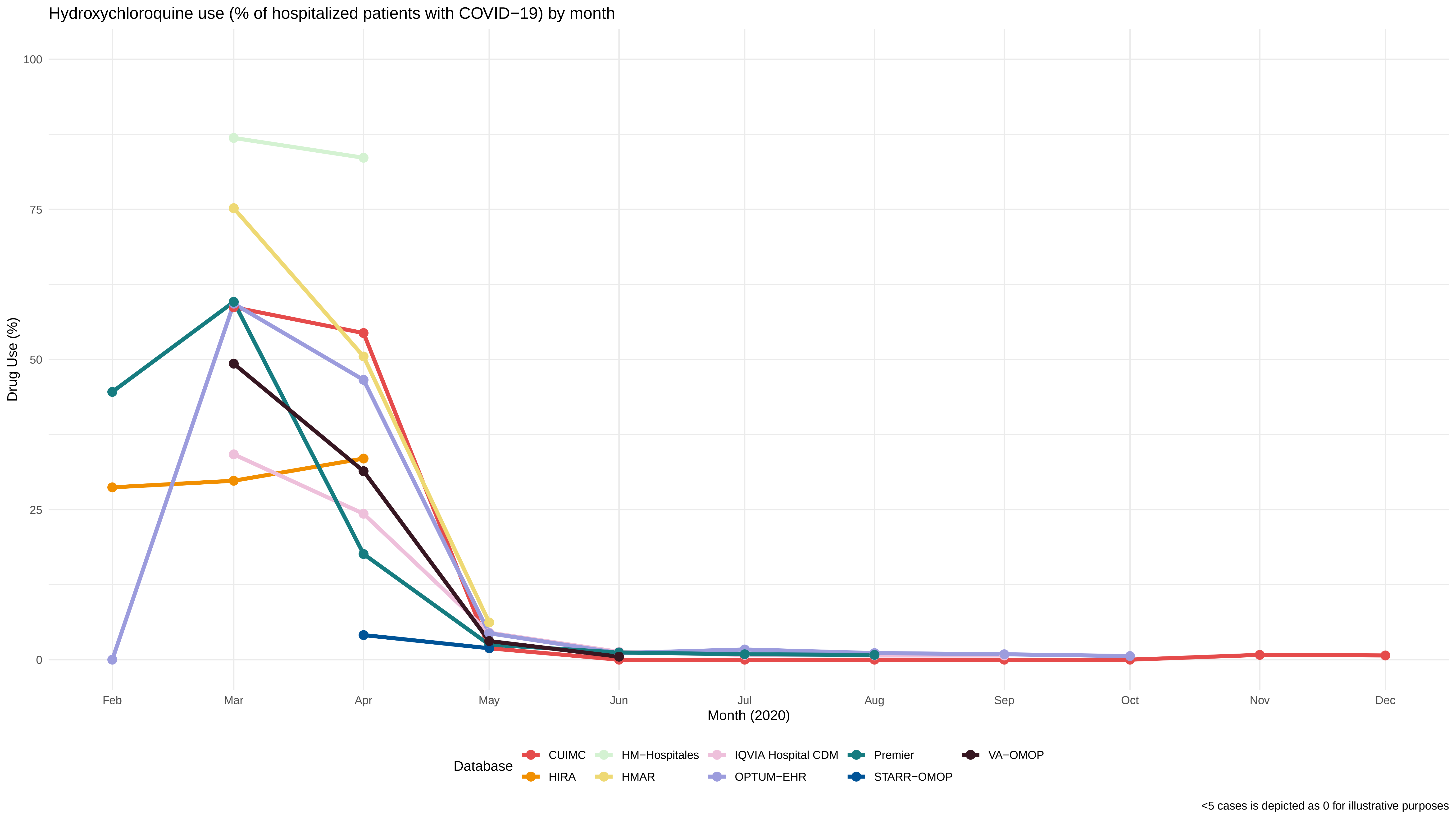
Hydroxychloroquine use (% of hospitalized patients with COVID−19) by month
100
75
50
25
0
Drug Use (%)
Feb
Mar
Apr
May
Jun
Jul
Aug
Sep
Oct
Nov
Dec
Month (2020)
Database
CUIMC
HIRA
HM−Hospitales
HMAR
IQVIA Hospital CDM
OPTUM−EHR
Premier
STARR−OMOP
VA−OMOP
<5 cases is depicted as 0 for illustrative purposes

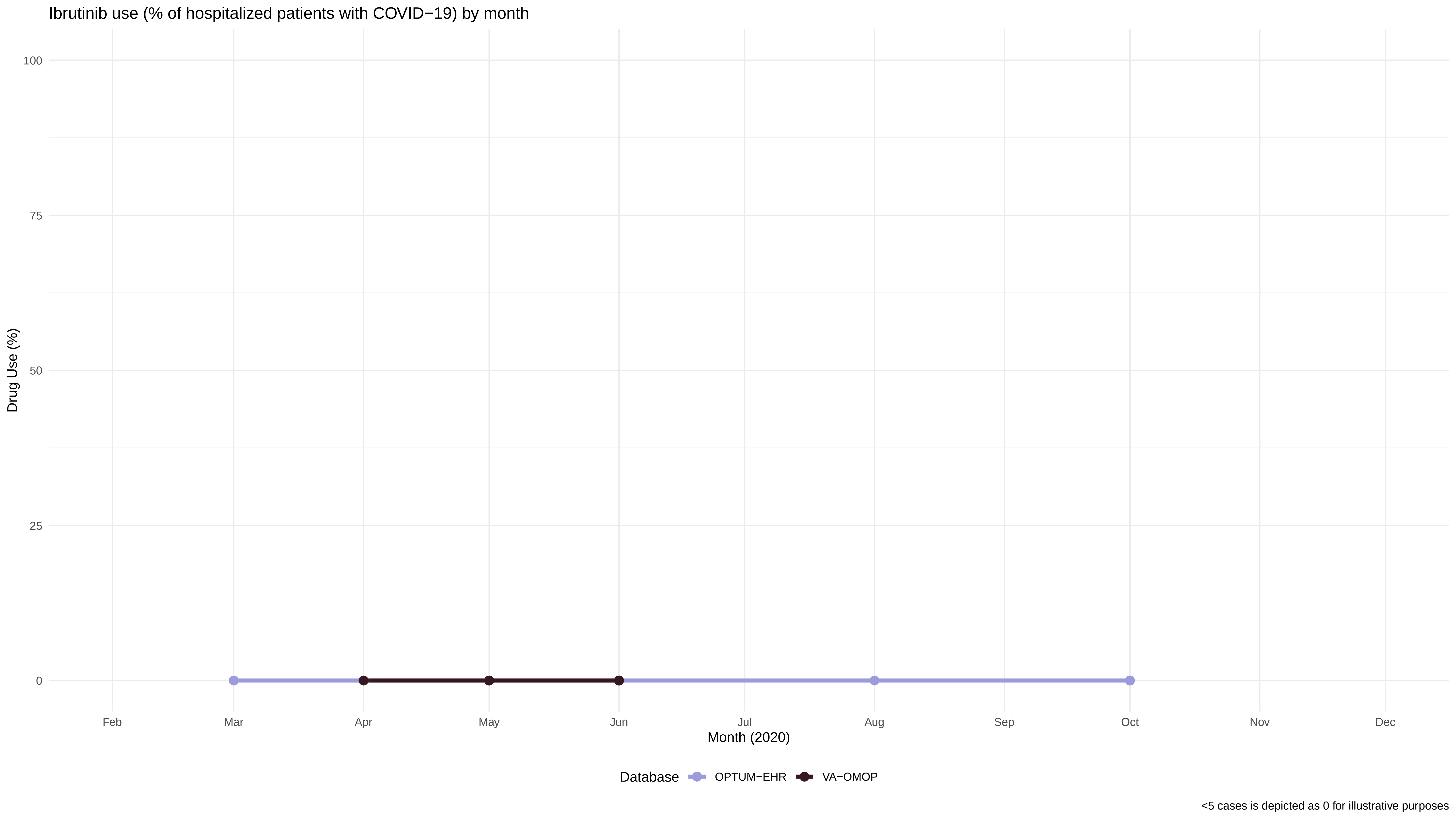
Ibrutinib use (% of hospitalized patients with COVID−19) by month
Drug Use (%)
100
75
50
25
0
Feb
Mar
Apr
May
Jun
Jul
Aug
Sep
Oct
Nov
Dec
Month (2020)
Database
OPTUM−EHR
VA−OMOP
<5 cases is depicted as 0 for illustrative purposes

Immunoglobulins use (% of hospitalized patients with COVID−19) by month

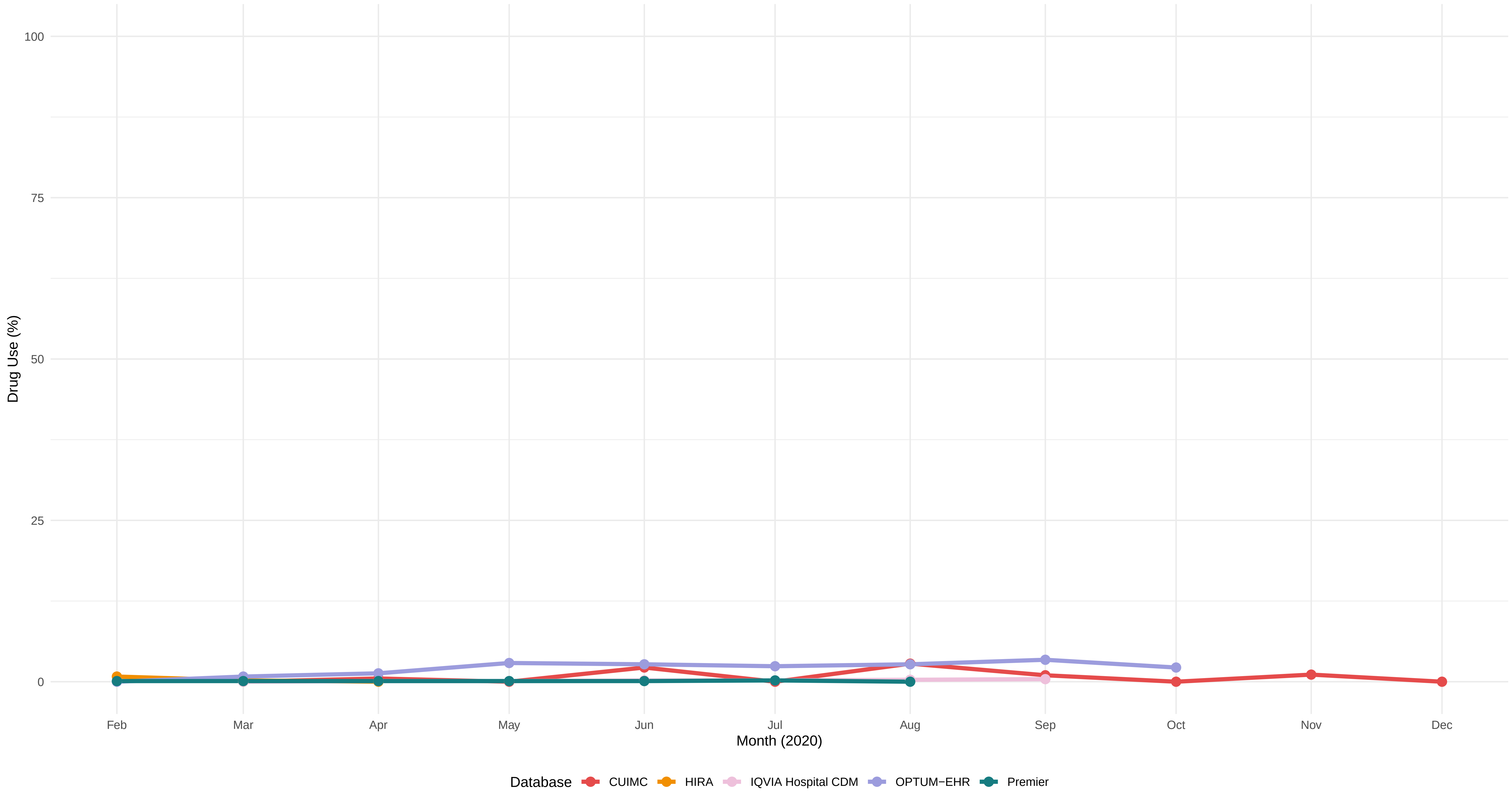

<5 cases is depicted as 0 for illustrative purposes

Infliximab use (% of hospitalized patients with COVID−19) by month

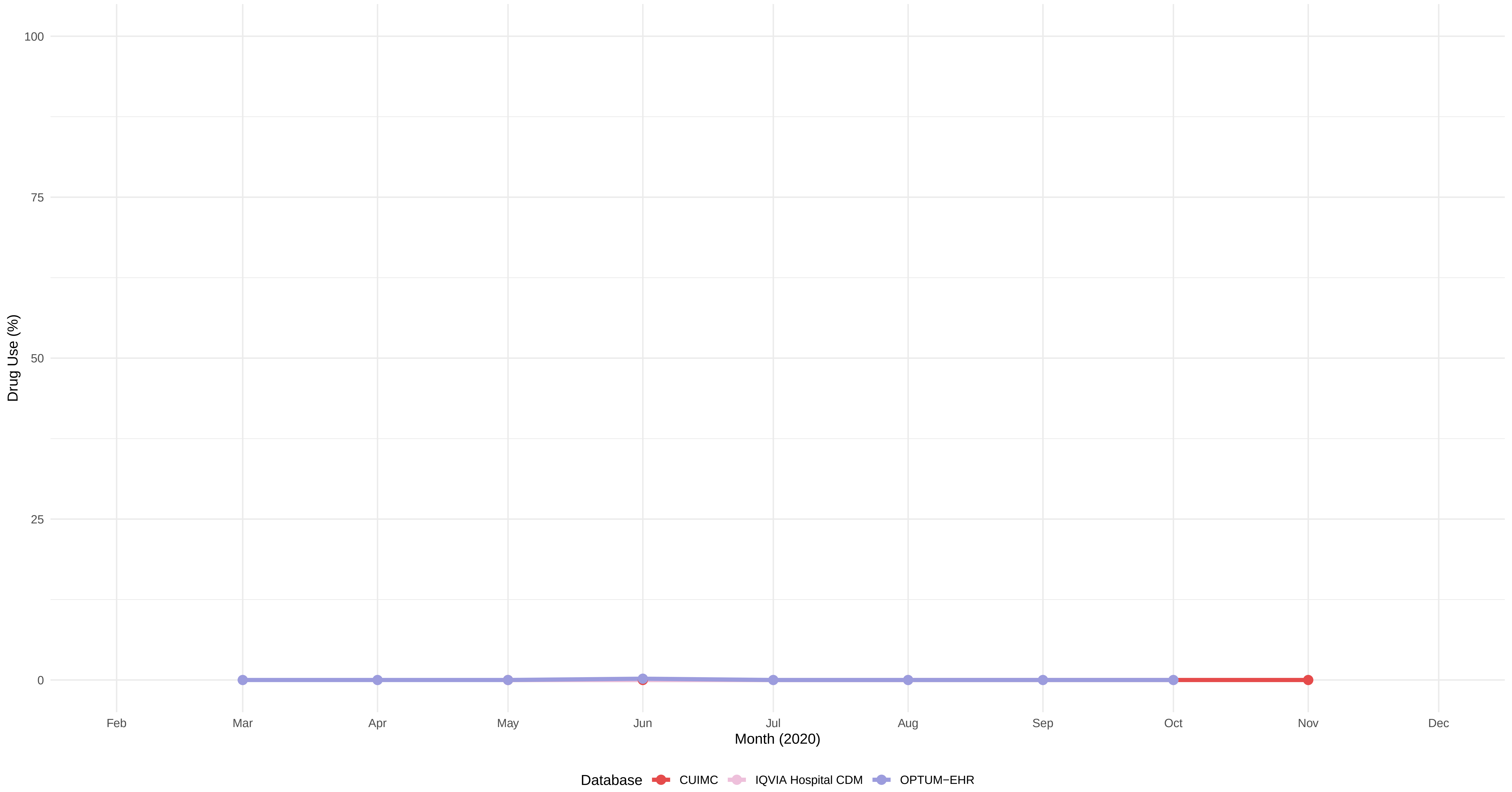

<5 cases is depicted as 0 for illustrative purposes

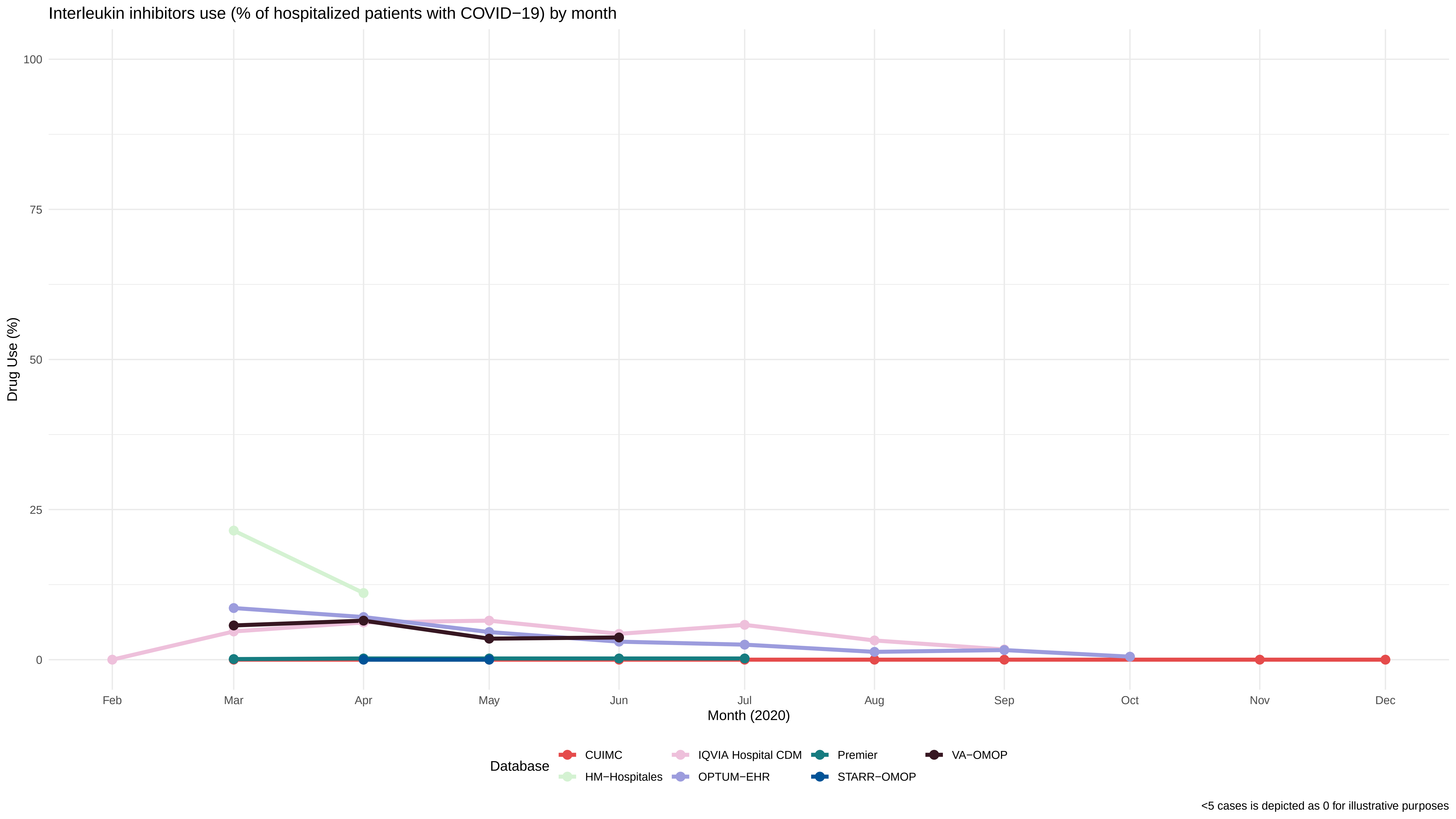
Interleukin inhibitors use (% of hospitalized patients with COVID−19) by month
Drug Use (%)
100
75
50
25
0
Feb
Mar
Apr
May
Jun
Jul
Aug
Sep
Oct
Nov
Dec
Month (2020)
Database
CUIMC
HM−Hospitales
IQVIA Hospital CDM
OPTUM−EHR
Premier
STARR−OMOP
VA−OMOP
<5 cases is depicted as 0 for illustrative purposes

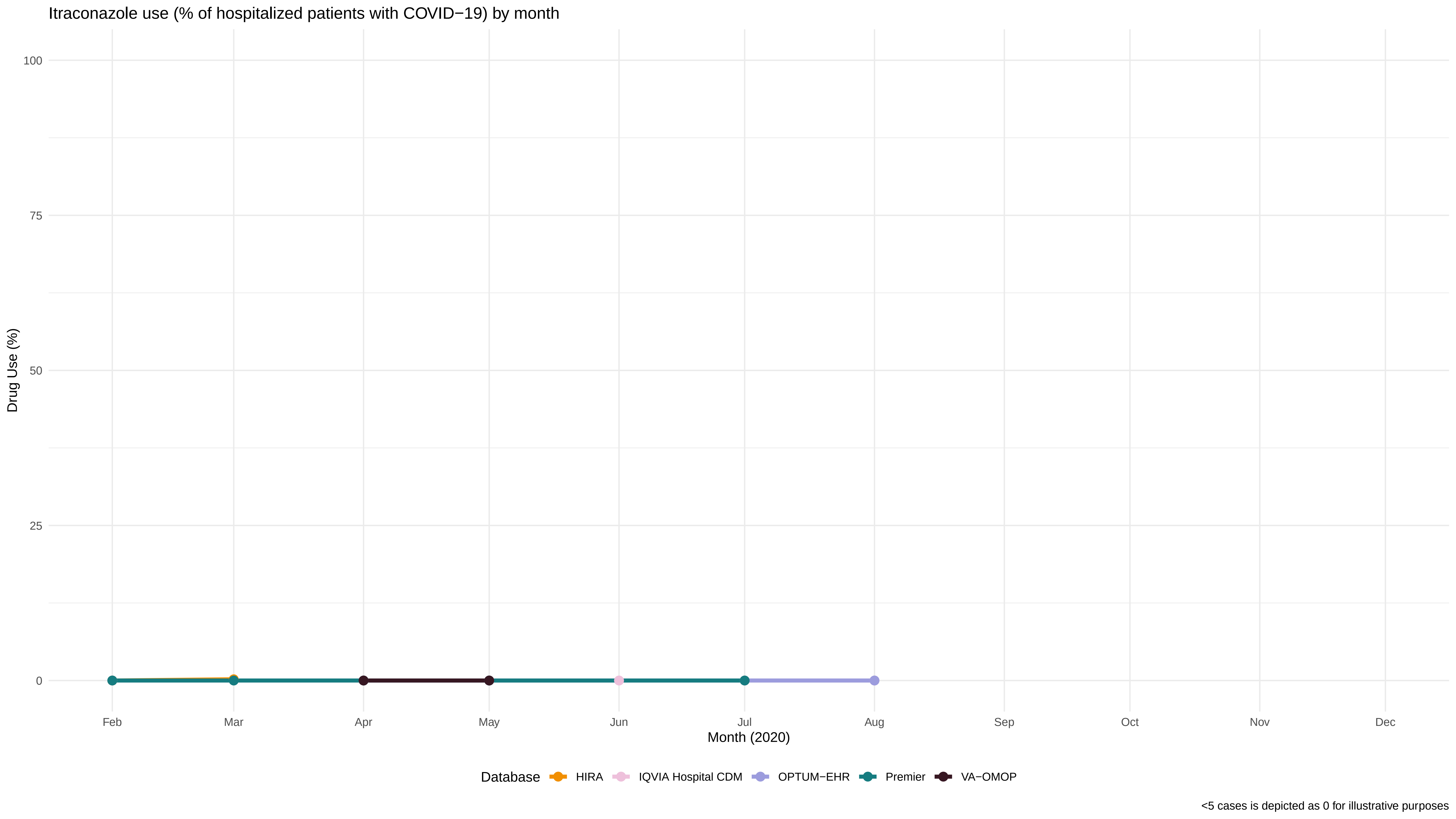
Itraconazole use (% of hospitalized patients with COVID−19) by month
Drug Use (%)
100
75
50
25
0
Feb
Mar
Apr
May
Jun
Jul
Aug
Sep
Oct
Nov
Dec
Month (2020)
Database
HIRA
IQVIA Hospital CDM
OPTUM−EHR
Premier
VA−OMOP
<5 cases is depicted as 0 for illustrative purposes

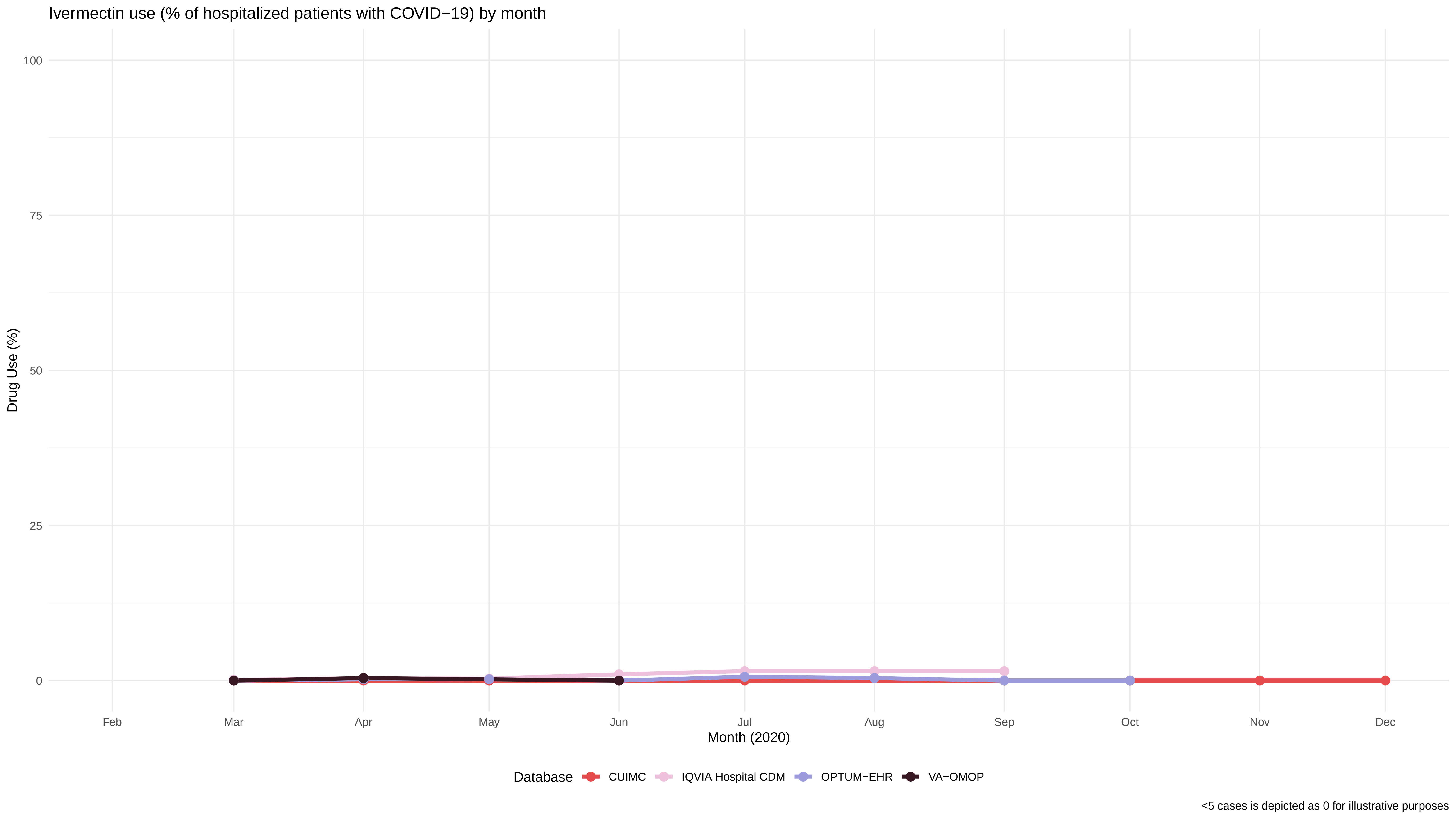
Ivermectin use (% of hospitalized patients with COVID−19) by month
100
75
50
25
0
Drug Use (%)
Feb
Mar
Apr
May
Jun
Jul
Aug
Sep
Oct
Nov
Dec
Month (2020)
Database
CUIMC
IQVIA Hospital CDM
OPTUM−EHR
VA−OMOP
<5 cases is depicted as 0 for illustrative purposes

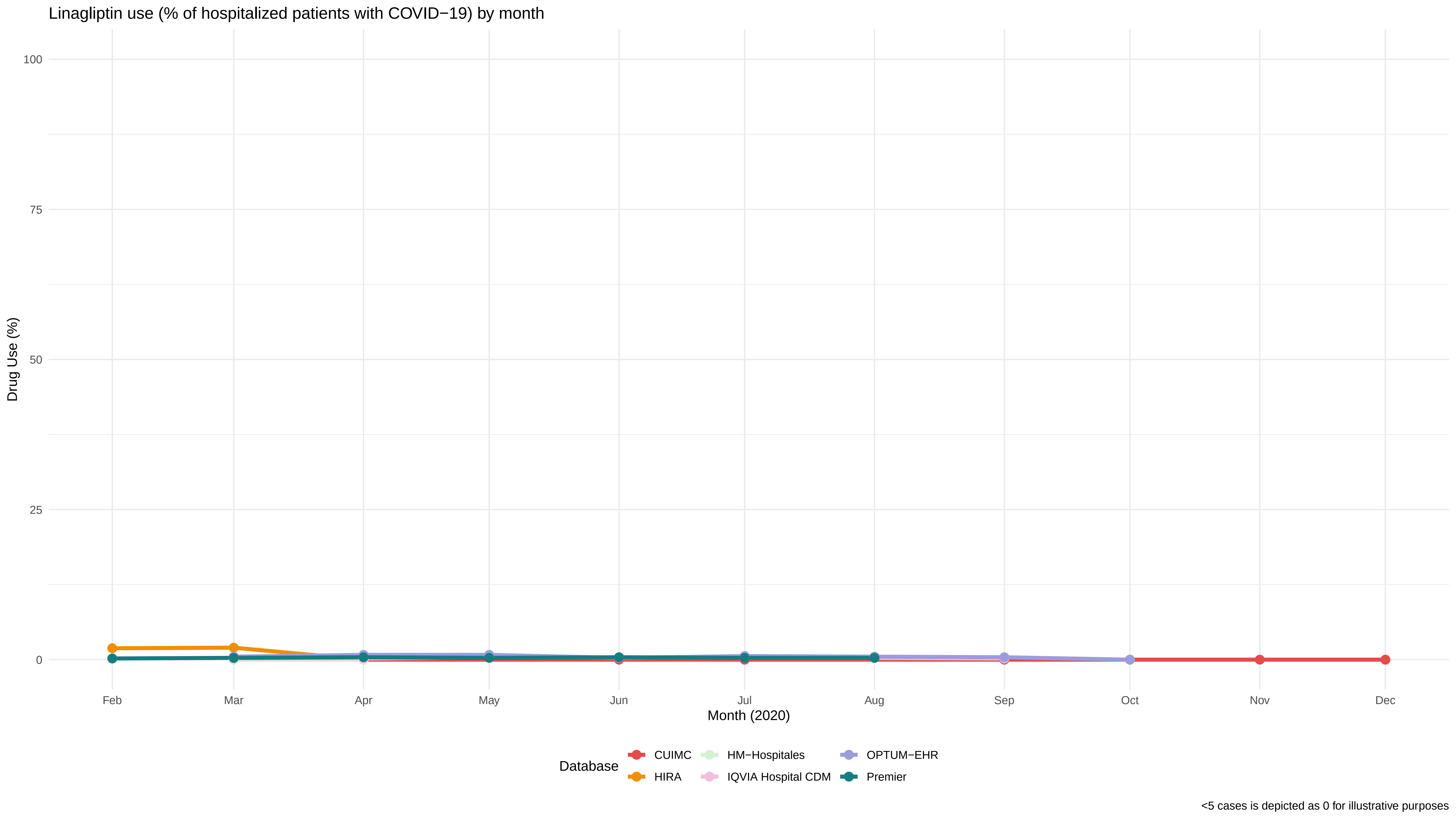

Linagliptin use (% of hospitalized patients with COVID−19) by month
Drug Use (%)
100
75
50
25
0
Feb
Mar
Apr
May
Jun
Jul
Aug
Sep
Oct
Nov
Dec
Month (2020)
Database
CUIMC
HIRA
HM−Hospitales
IQVIA Hospital CDM
OPTUM−EHR
Premier
<5 cases is depicted as 0 for illustrative purposes

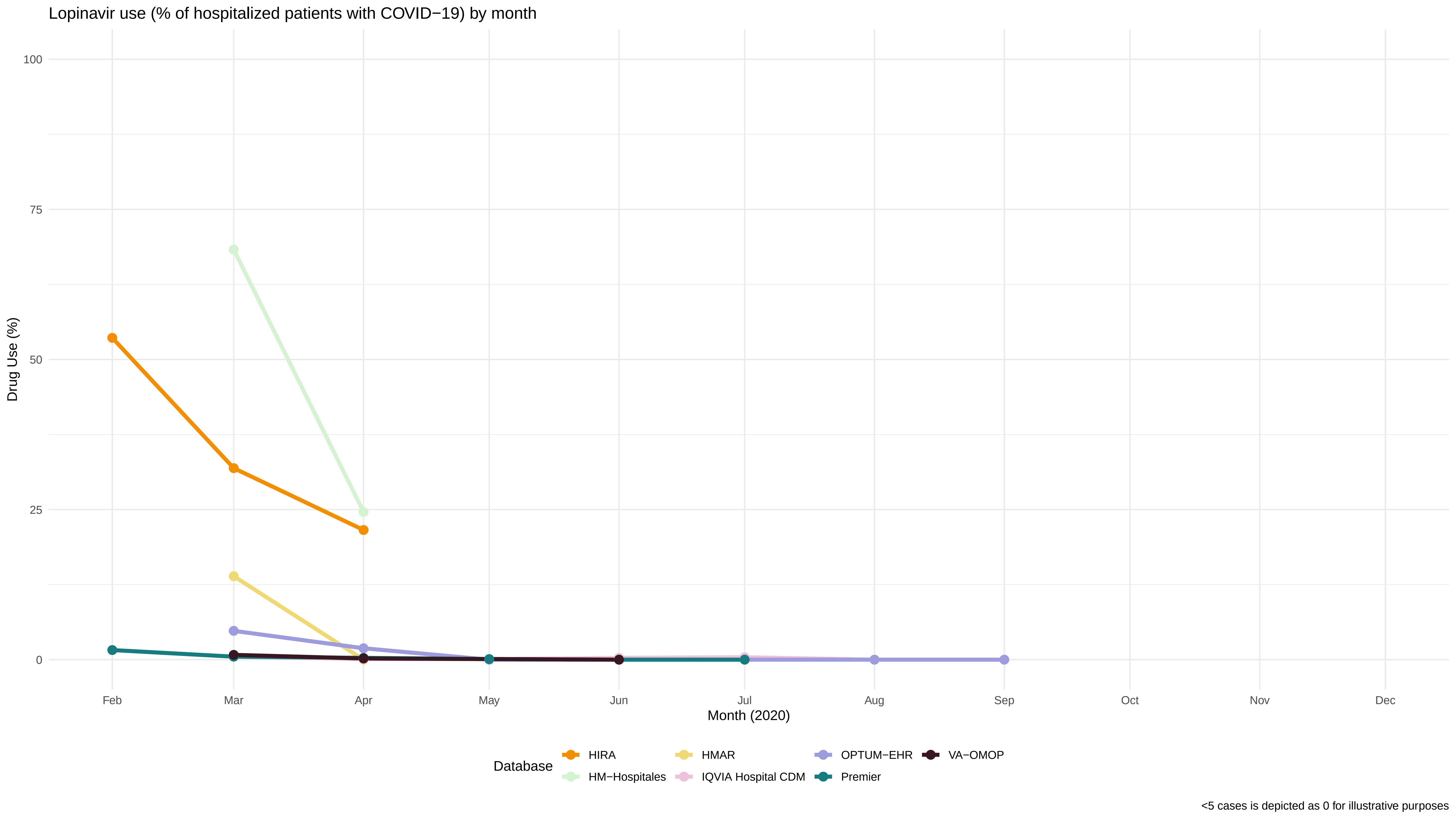

Lopinavir use (% of hospitalized patients with COVID−19) by month
Drug Use (%)
100
75
50
25
0
Feb
Mar
Apr
May
Jun
Jul
Aug
Sep
Oct
Nov
Dec
Month (2020)
Database
HIRA
HM−Hospitales
HMAR
IQVIA Hospital CDM
OPTUM−EHR
Premier
VA−OMOP
<5 cases is depicted as 0 for illustrative purposes

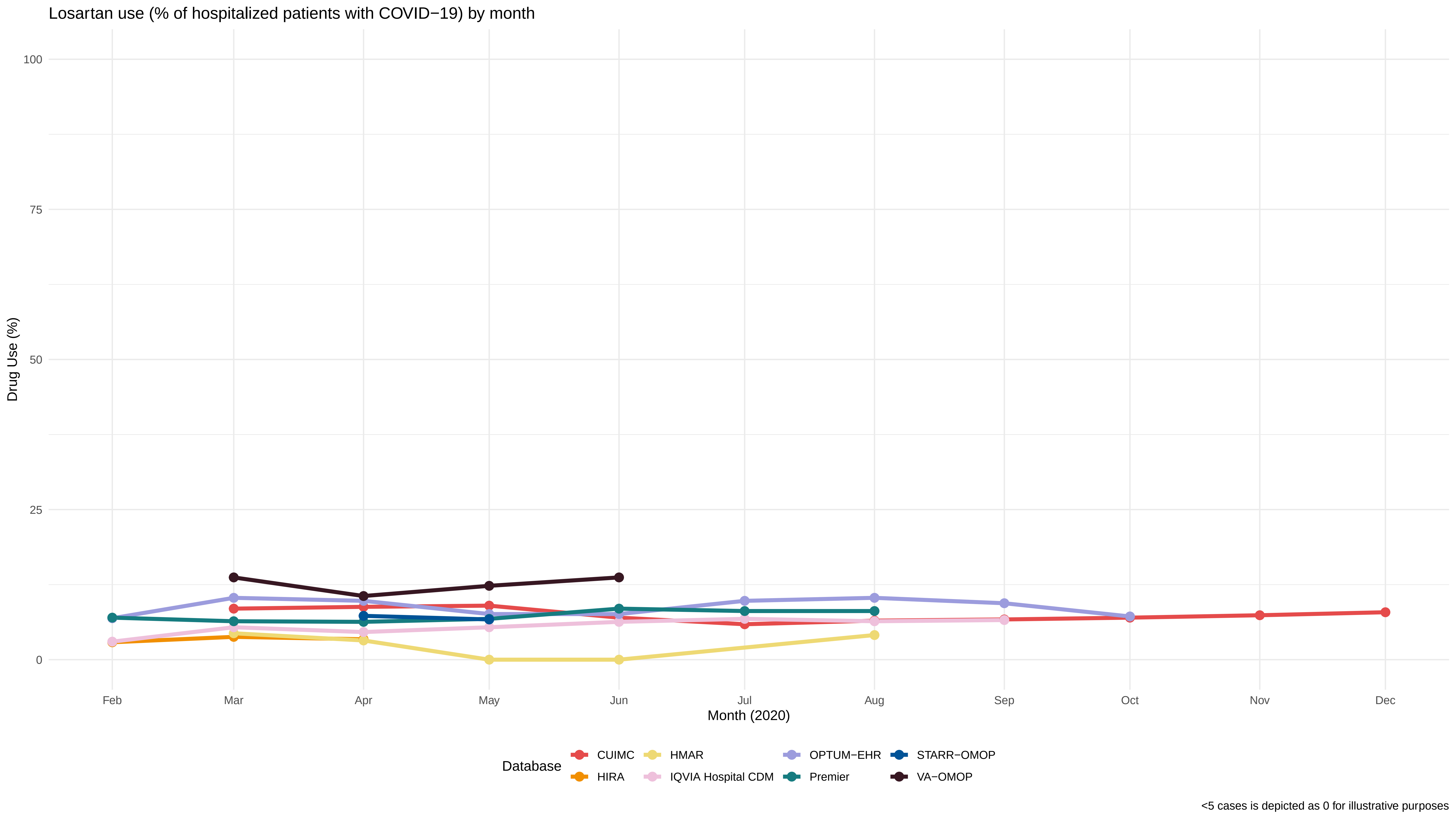
Losartan use (% of hospitalized patients with COVID−19) by month
100
75
50
25
0
Drug Use (%)
Feb
Mar
Apr
May
Jun
Jul
Aug
Sep
Oct
Nov
Dec
Month (2020)
Database
CUIMC
HIRA
HMAR
IQVIA Hospital CDM
OPTUM−EHR
Premier
STARR−OMOP
VA−OMOP
<5 cases is depicted as 0 for illustrative purposes

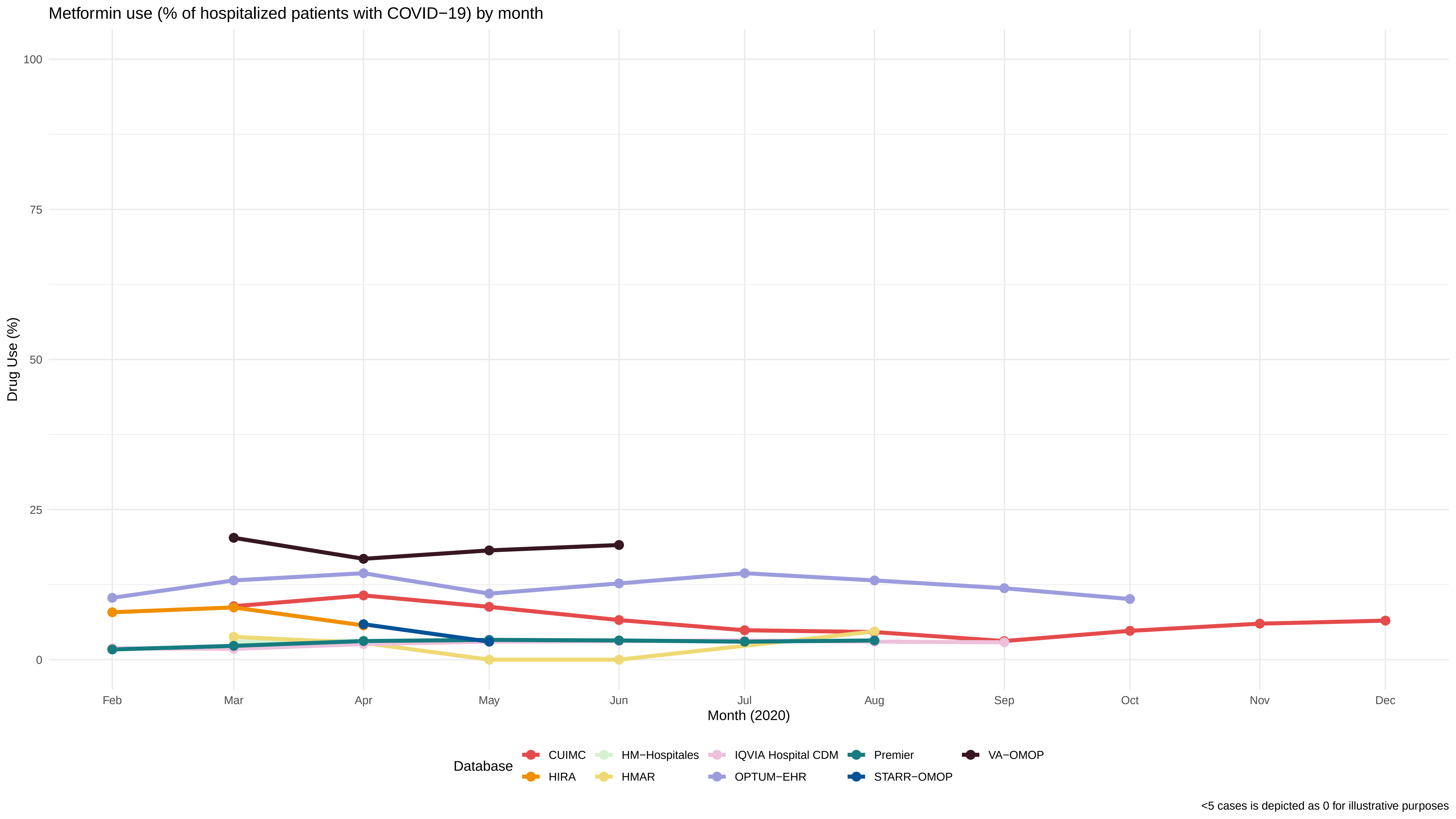
Metformin use (% of hospitalized patients with COVID−19) by month
Drug Use (%)
100
75
50
25
0
Feb
Mar
Apr
May
Jun
Jul
Aug
Sep
Oct
Nov
Dec
Month (2020)
Database
CUIMC
HIRA
HM−Hospitales
HMAR
IQVIA Hospital CDM
OPTUM−EHR
Premier
STARR−OMOP
VA−OMOP
<5 cases is depicted as 0 for illustrative purposes

Nitazoxanide use (% of hospitalized patients with COVID−19) by month

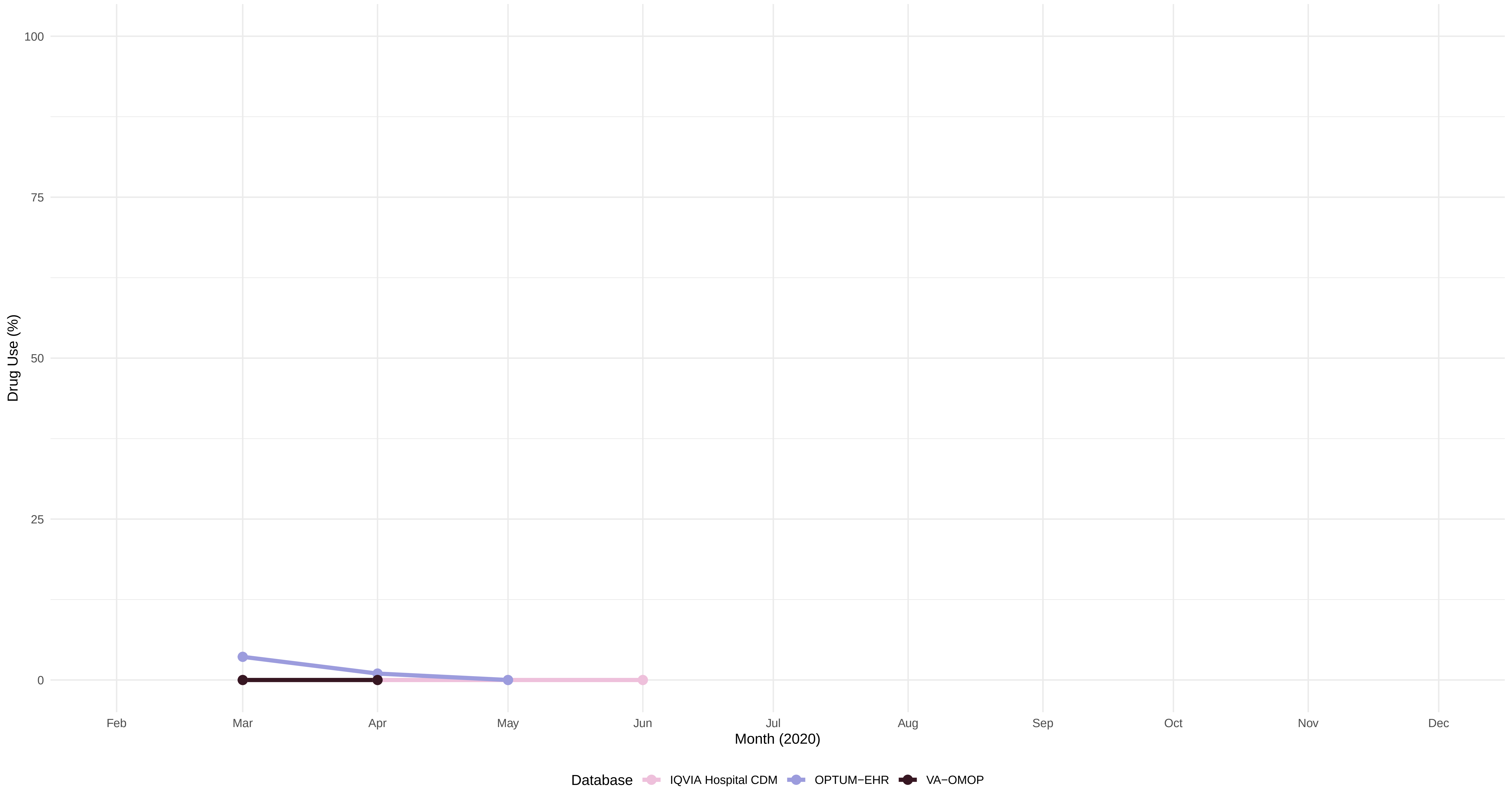

<5 cases is depicted as 0 for illustrative purposes

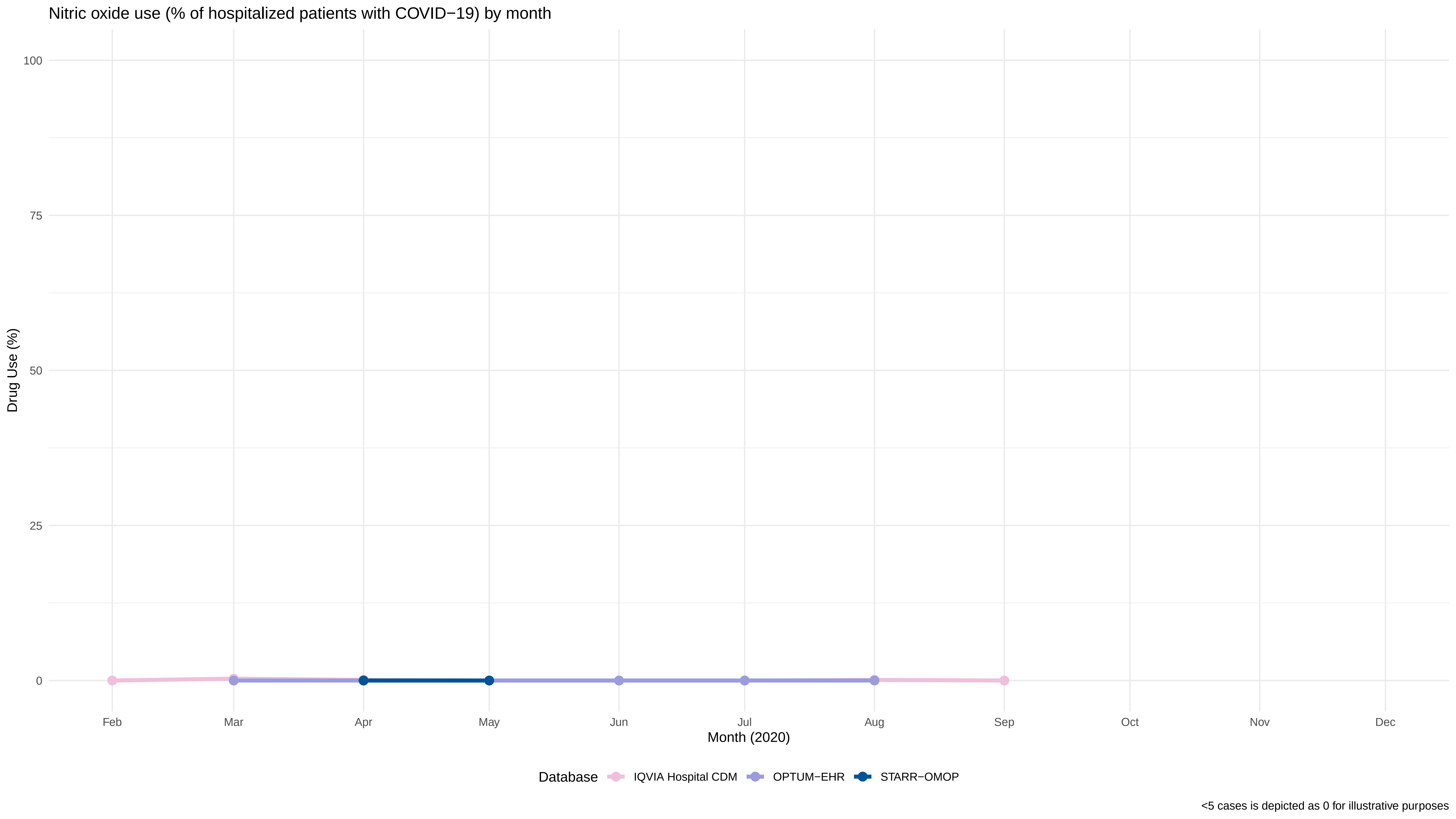

Nitric oxide use (% of hospitalized patients with COVID−19) by month
Drug Use (%)
100
75
50
25
0
Feb
Mar
Apr
May
Jun
Jul
Aug
Sep
Oct
Nov
Dec
Month (2020)
Database
IQVIA Hospital CDM
OPTUM−EHR
STARR−OMOP
<5 cases is depicted as 0 for illustrative purposes

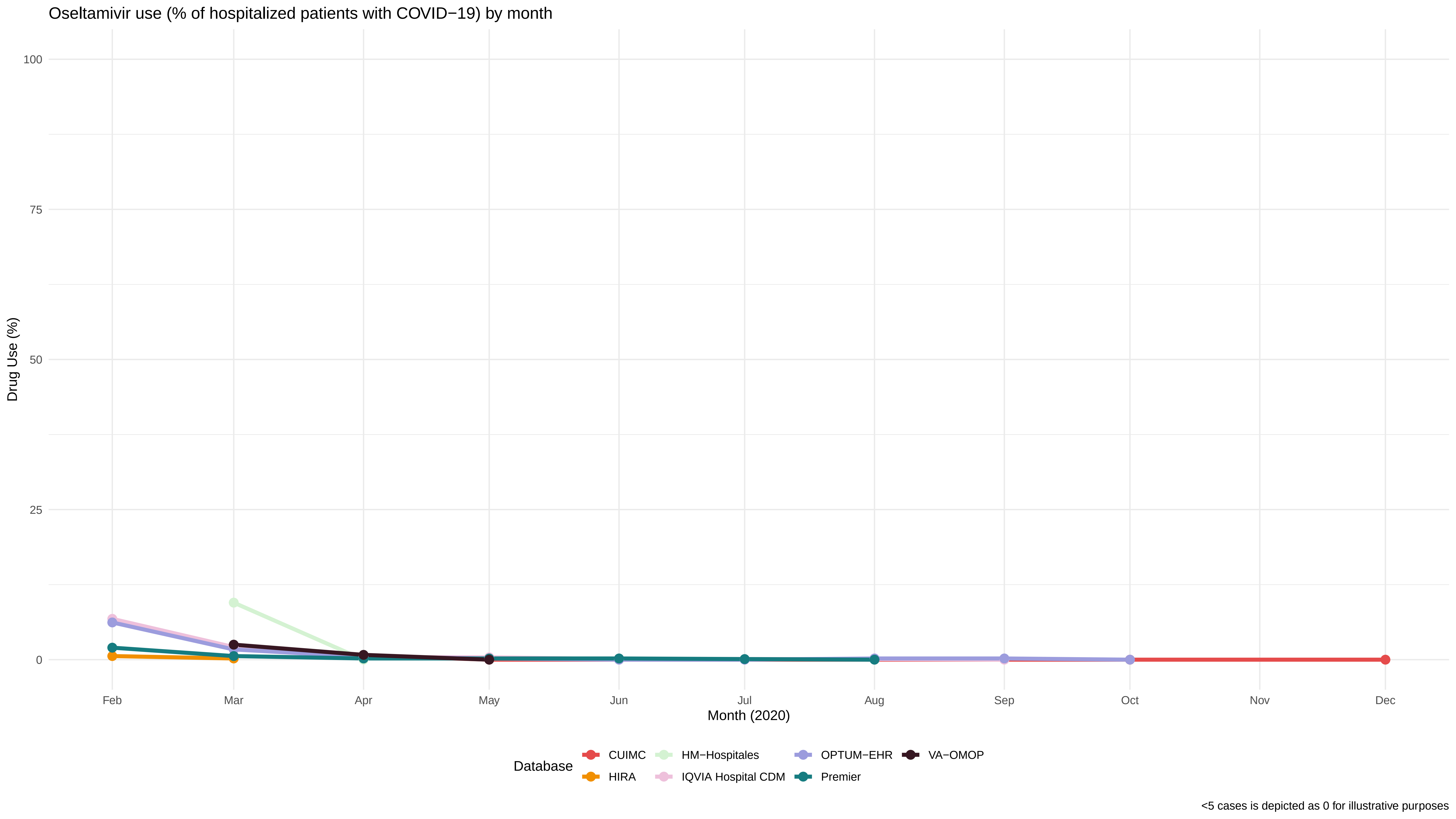
Oseltamivir use (% of hospitalized patients with COVID−19) by month
Drug Use (%)
100
75
50
25
0
Feb
Mar
Apr
May
Jun
Jul
Aug
Sep
Oct
Nov
Dec
Month (2020)
Database
CUIMC
HIRA
HM−Hospitales
IQVIA Hospital CDM
OPTUM−EHR
Premier
VA−OMOP
<5 cases is depicted as 0 for illustrative purposes

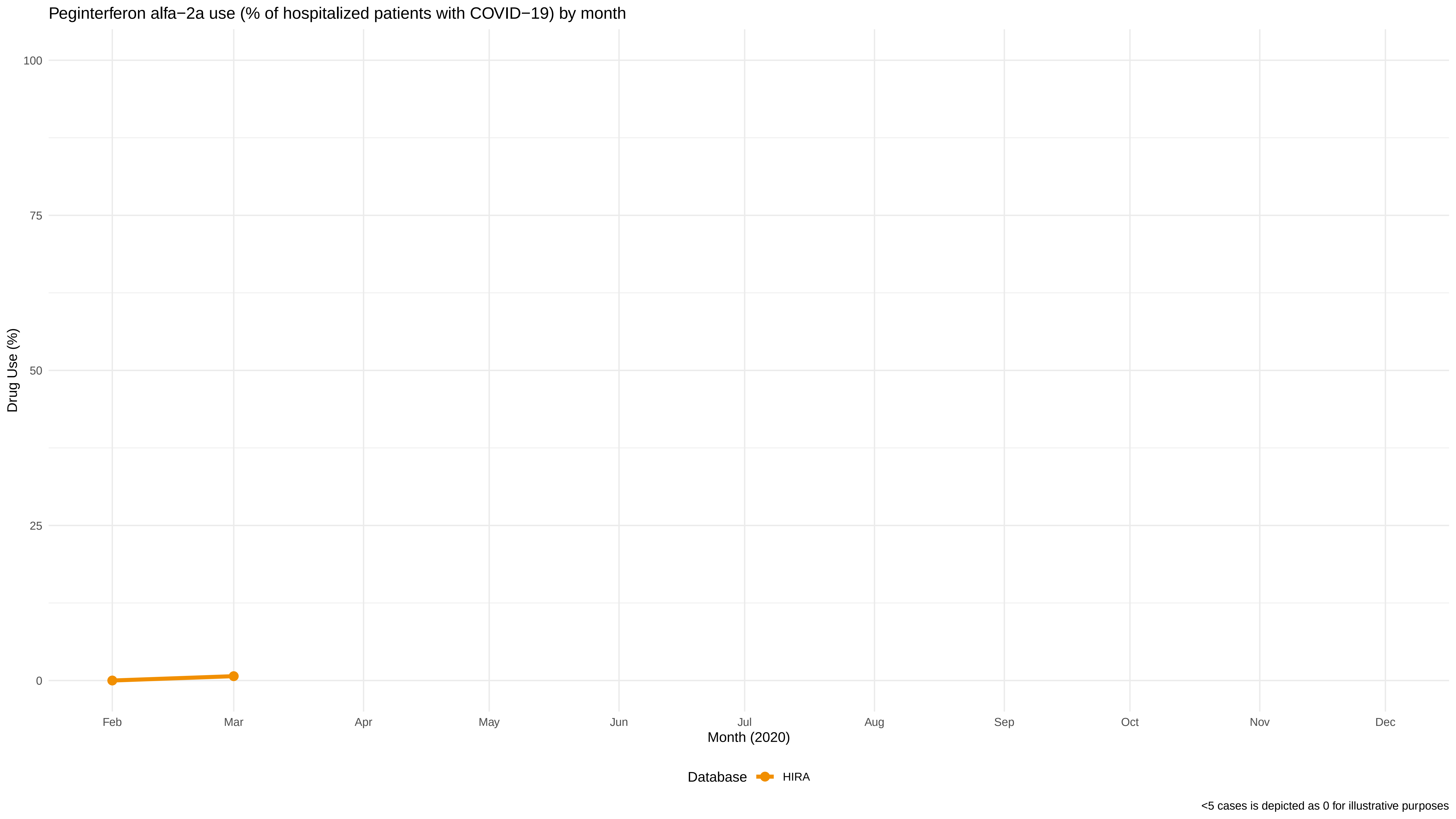

Peginterferon alfa−2a use (% of hospitalized patients with COVID−19) by month
Drug Use (%)
100
75
50
25
0
Feb
Mar
Apr
May
Jun
Jul
Aug
Sep
Oct
Nov
Dec
Month (2020)
Database
HIRA
<5 cases is depicted as 0 for illustrative purposes

Pirfenidone use (% of hospitalized patients with COVID−19) by month

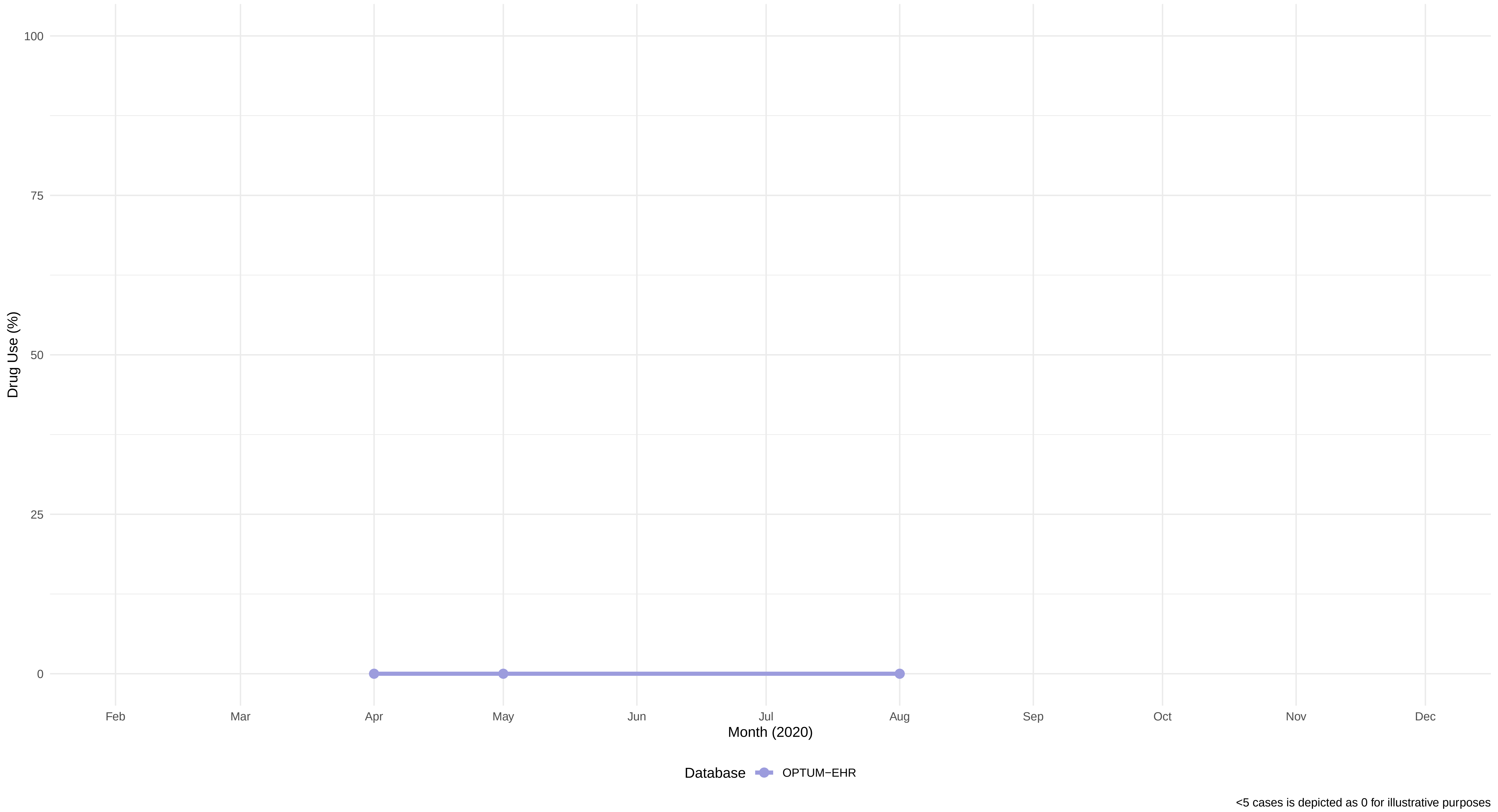

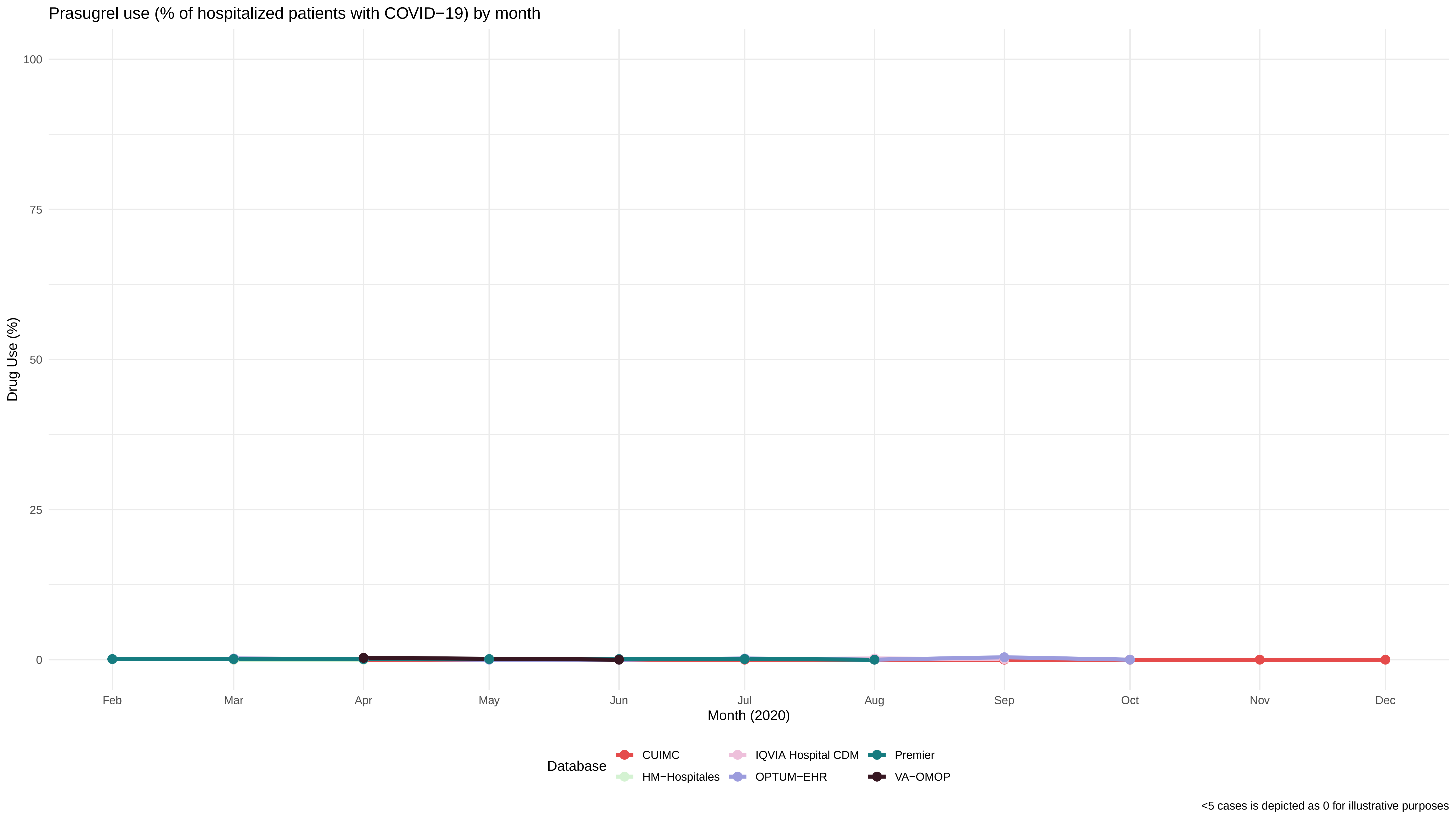

Prasugrel use (% of hospitalized patients with COVID−19) by month
Drug Use (%)
100
75
50
25
0
Feb
Mar
Apr
May
Jun
Jul
Aug
Sep
Oct
Nov
Dec
Month (2020)
Database
CUIMC
HM−Hospitales
IQVIA Hospital CDM
OPTUM−EHR
Premier
VA−OMOP
<5 cases is depicted as 0 for illustrative purposes

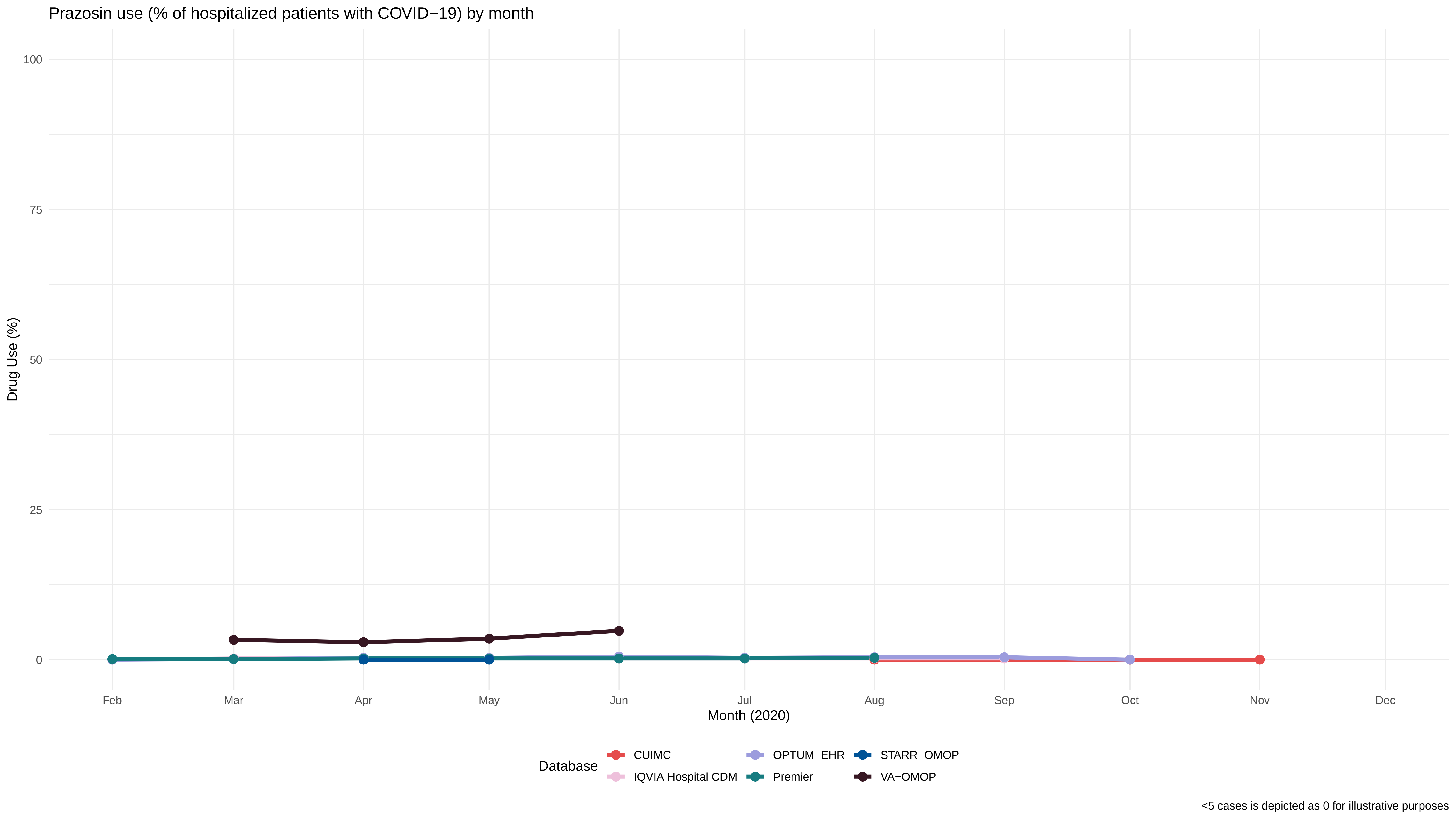
Prazosin use (% of hospitalized patients with COVID−19) by month
Drug Use (%)
100
75
50
25
0
Feb
Mar
Apr
May
Jun
Jul
Aug
Sep
Oct
Nov
Dec
Month (2020)
Database
CUIMC
IQVIA Hospital CDM
OPTUM−EHR
Premier
STARR−OMOP
VA−OMOP
<5 cases is depicted as 0 for illustrative purposes

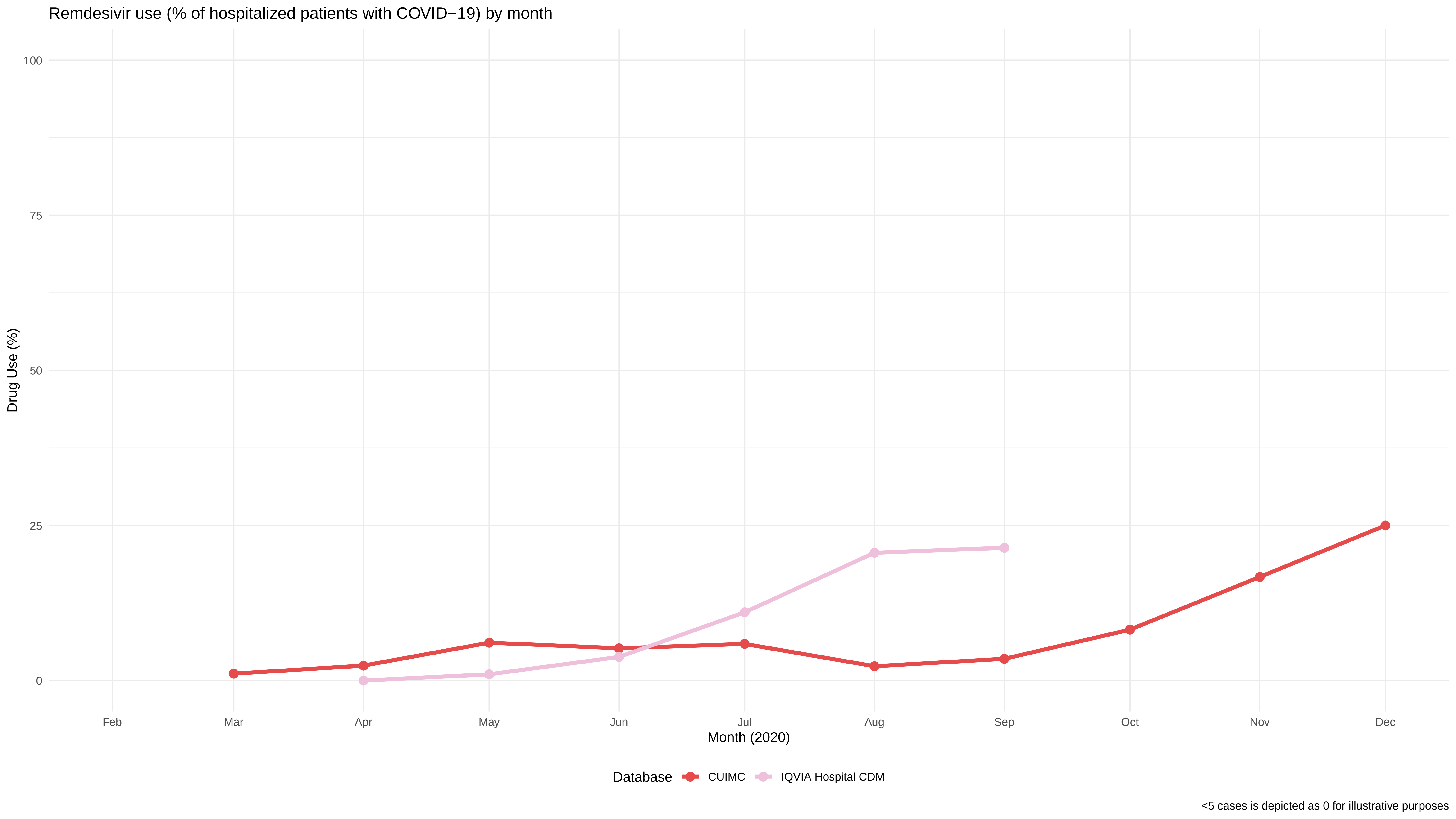

Remdesivir use (% of hospitalized patients with COVID−19) by month
Drug Use (%)
100
75
50
25
0
Feb
Mar
Apr
May
Jun
Jul
Aug
Sep
Oct
Nov
Dec
Month (2020)
Database
CUIMC
IQVIA Hospital CDM
<5 cases is depicted as 0 for illustrative purposes

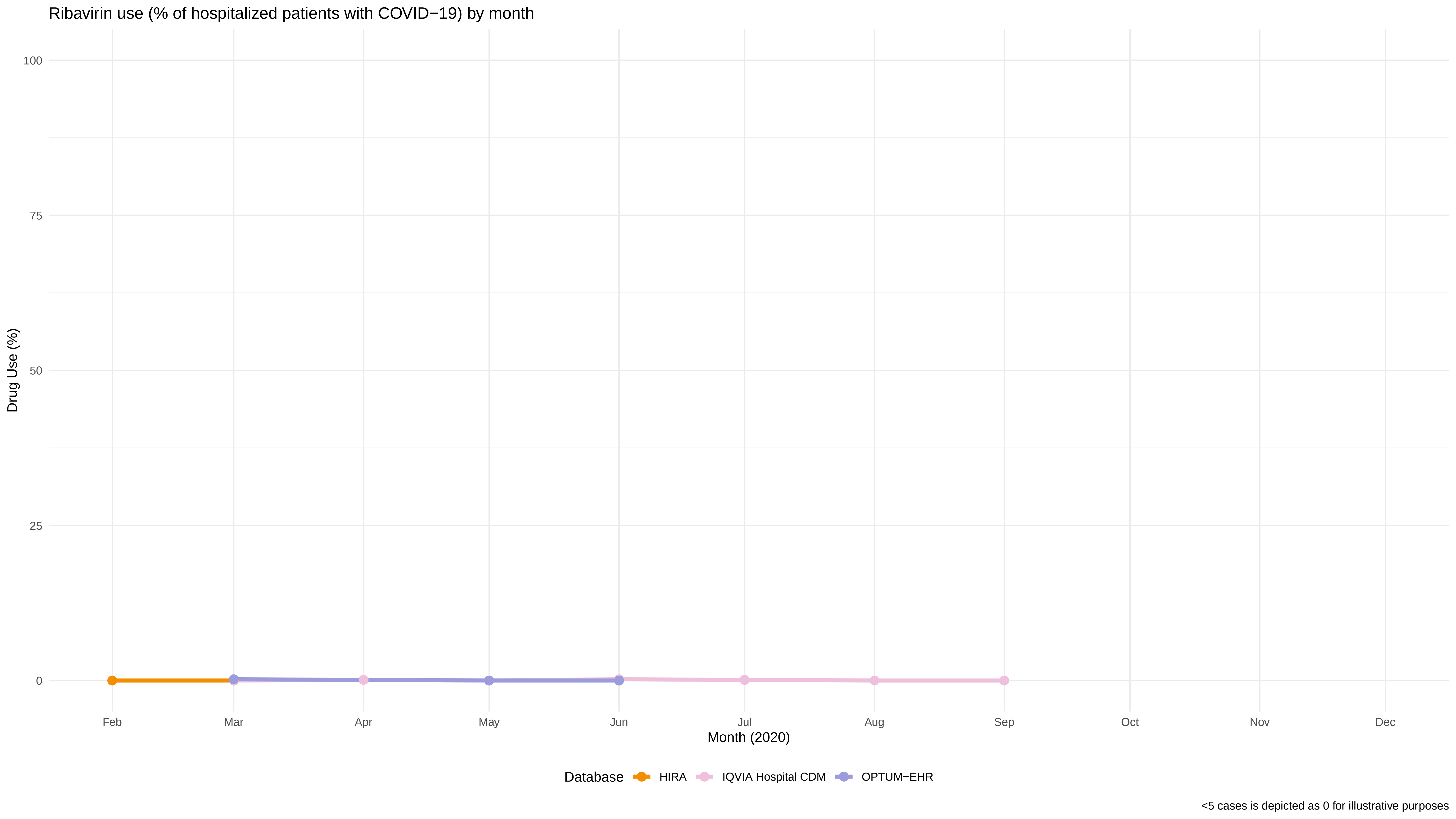
Ribavirin use (% of hospitalized patients with COVID−19) by month
100
75
50
25
0
Drug Use (%)
Feb
Mar
Apr
May
Jun
Jul
Aug
Sep
Oct
Nov
Dec
Month (2020)
Database
HIRA
IQVIA Hospital CDM
OPTUM−EHR
<5 cases is depicted as 0 for illustrative purposes

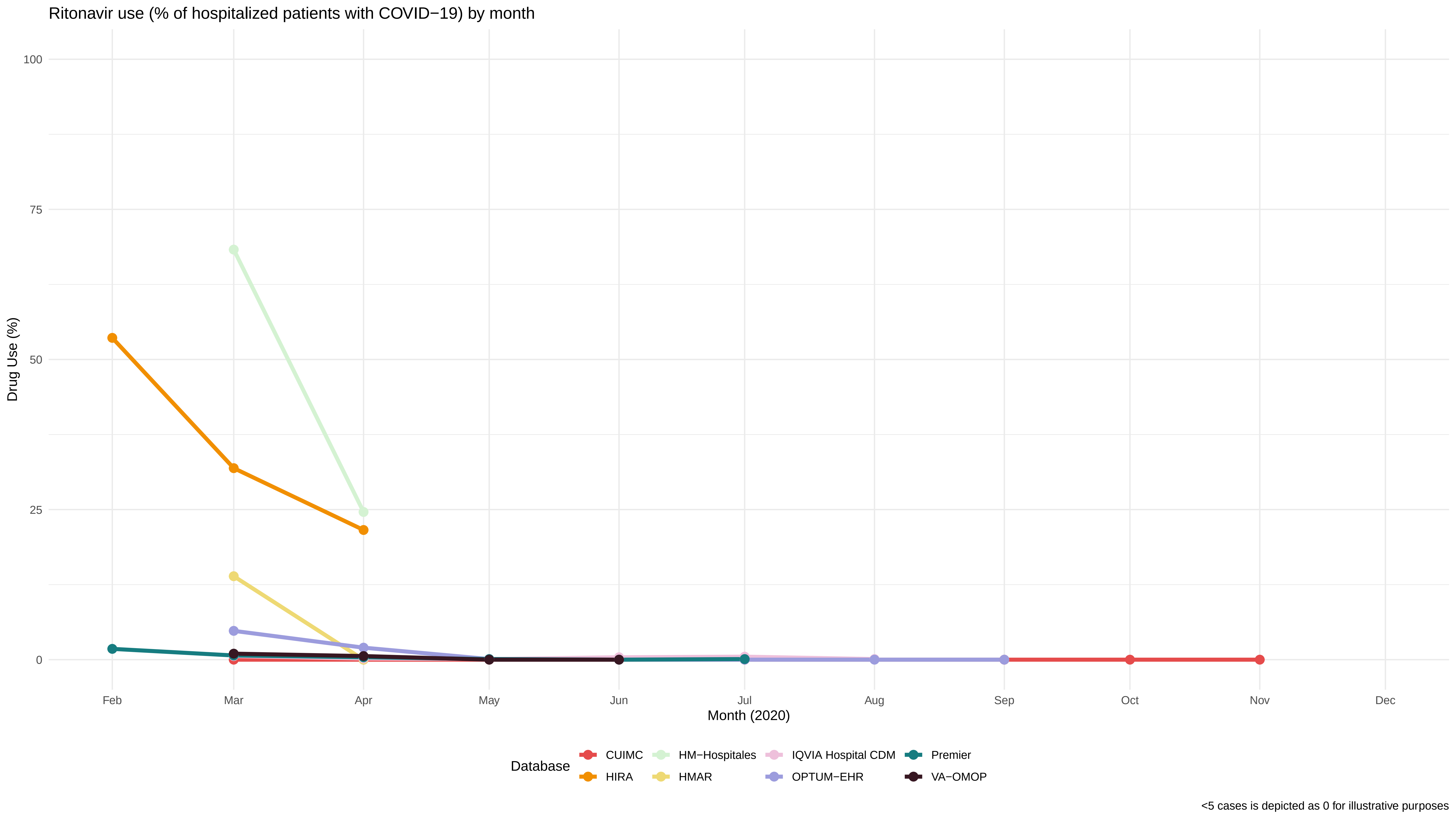
Ritonavir use (% of hospitalized patients with COVID−19) by month
Drug Use (%)
100
75
50
25
0
Feb
Mar
Apr
May
Jun
Jul
Aug
Sep
Oct
Nov
Dec
Month (2020)
Database
CUIMC
HIRA
HM−Hospitales
HMAR
IQVIA Hospital CDM
OPTUM−EHR
Premier
VA−OMOP
<5 cases is depicted as 0 for illustrative purposes

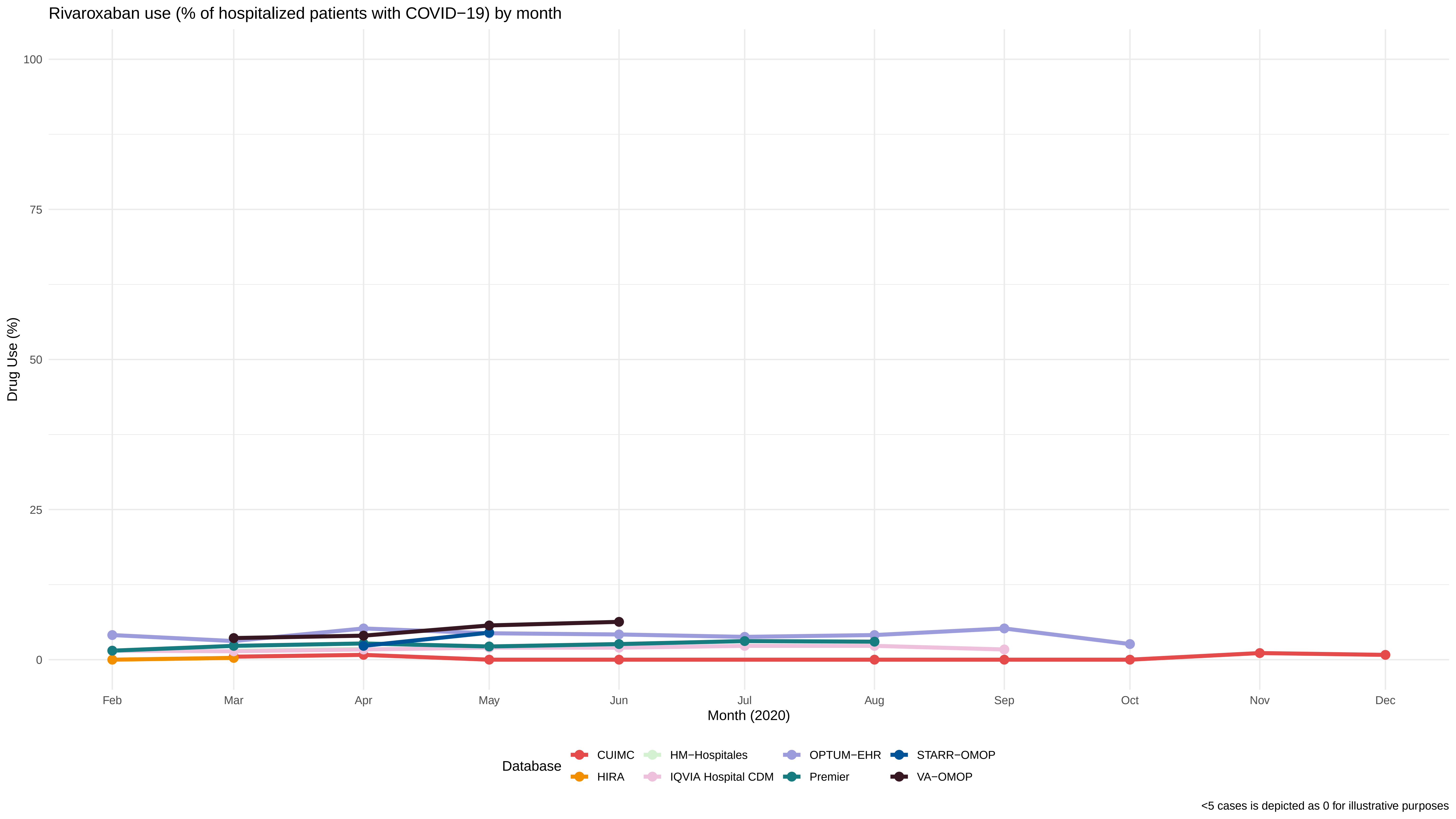
Rivaroxaban use (% of hospitalized patients with COVID−19) by month
Drug Use (%)
100
75
50
25
0
Feb
Mar
Apr
May
Jun
Jul
Aug
Sep
Oct
Nov
Dec
Month (2020)
Database
CUIMC
HIRA
HM−Hospitales
IQVIA Hospital CDM
OPTUM−EHR
Premier
STARR−OMOP
VA−OMOP
<5 cases is depicted as 0 for illustrative purposes

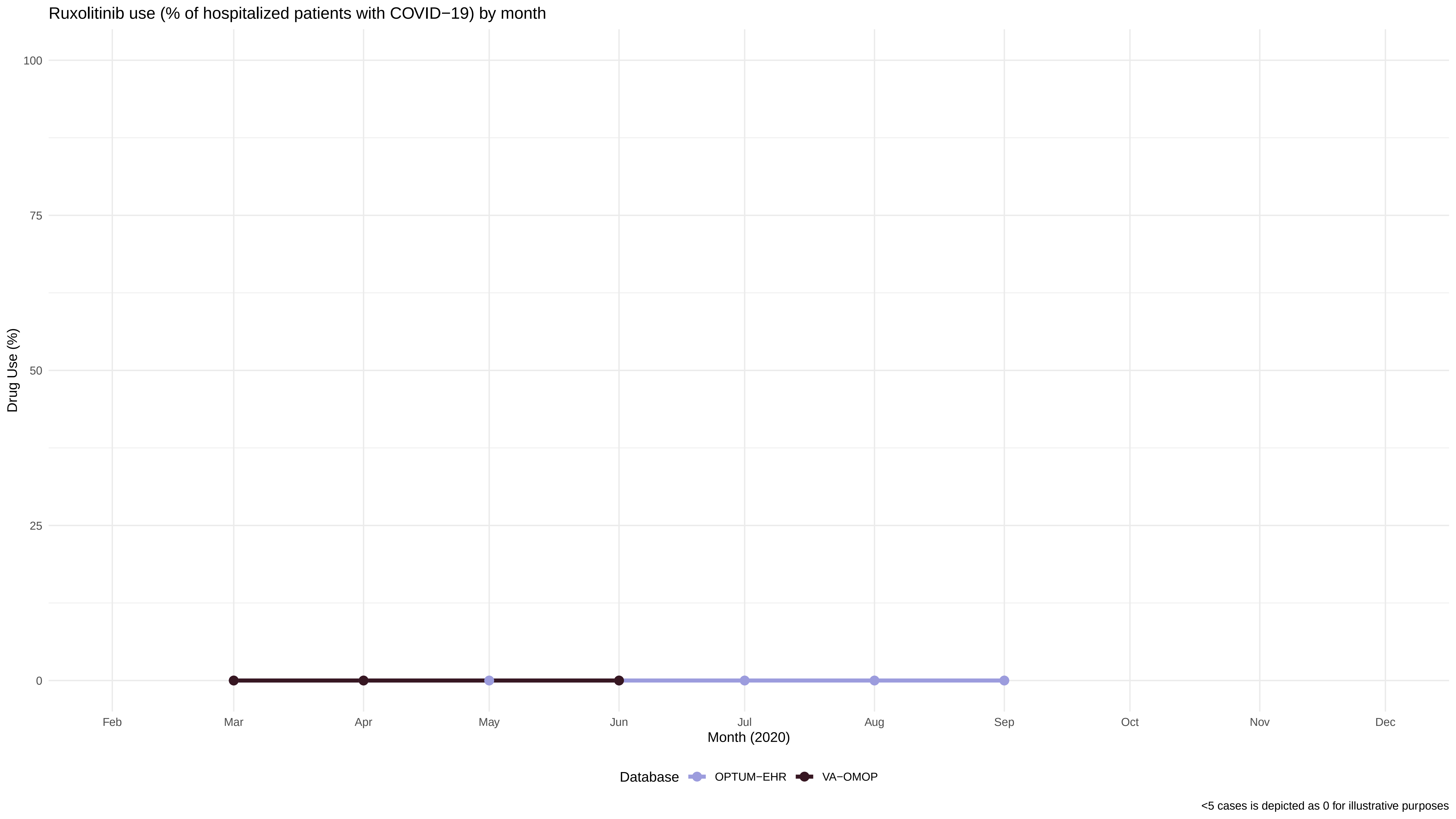
Ruxolitinib use (% of hospitalized patients with COVID−19) by month
100
75
50
25
0
Drug Use (%)
Feb
Mar
Apr
May
Jun
Jul
Aug
Sep
Oct
Nov
Dec
Month (2020)
Database
OPTUM−EHR
VA−OMOP
<5 cases is depicted as 0 for illustrative purposes

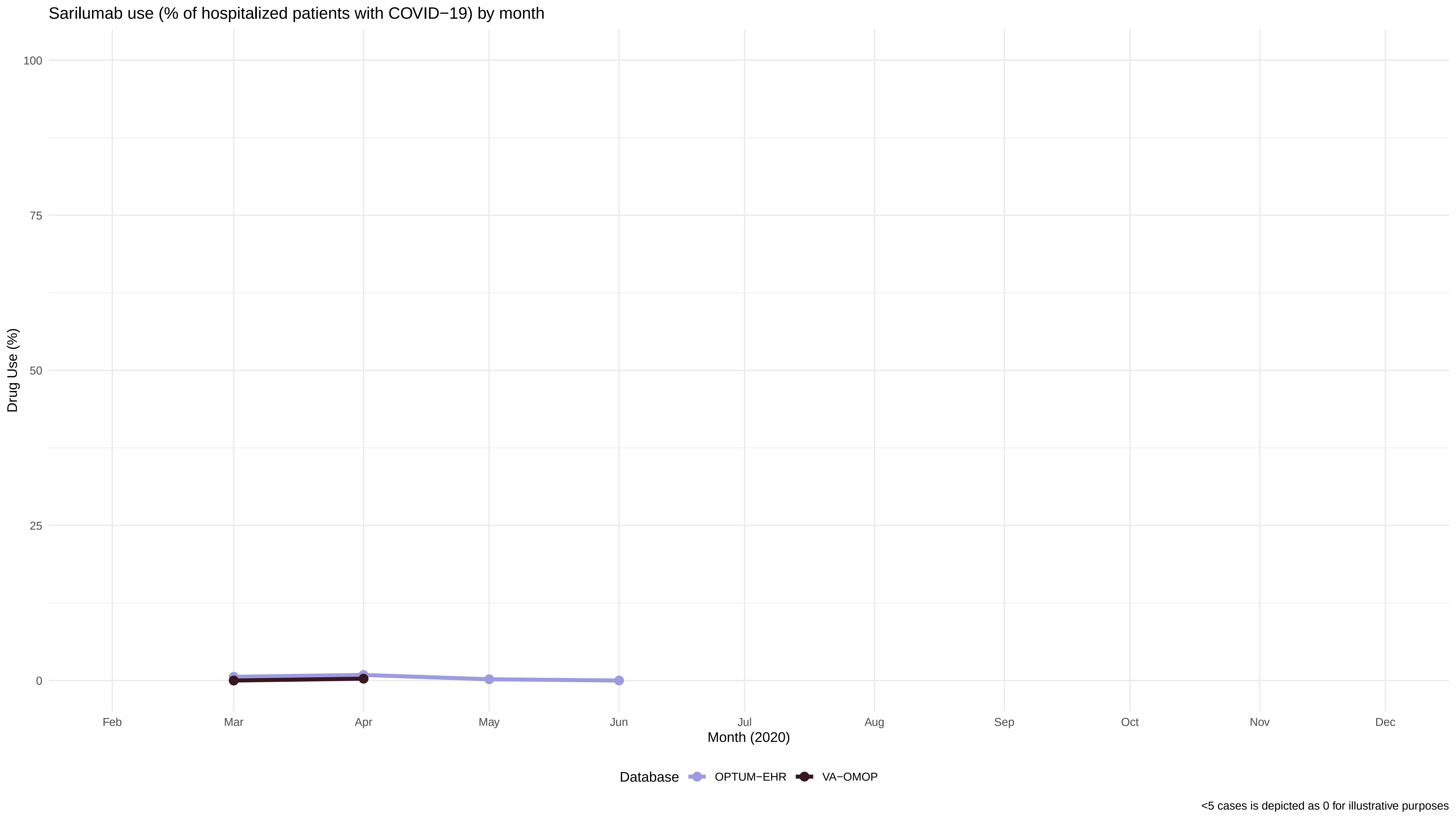
Sarilumab use (% of hospitalized patients with COVID−19) by month
100
75
50
25
0
Drug Use (%)
Feb
Mar
Apr
May
Jun
Jul
Aug
Sep
Oct
Nov
Dec
Month (2020)
Database
OPTUM−EHR
VA−OMOP
<5 cases is depicted as 0 for illustrative purposes

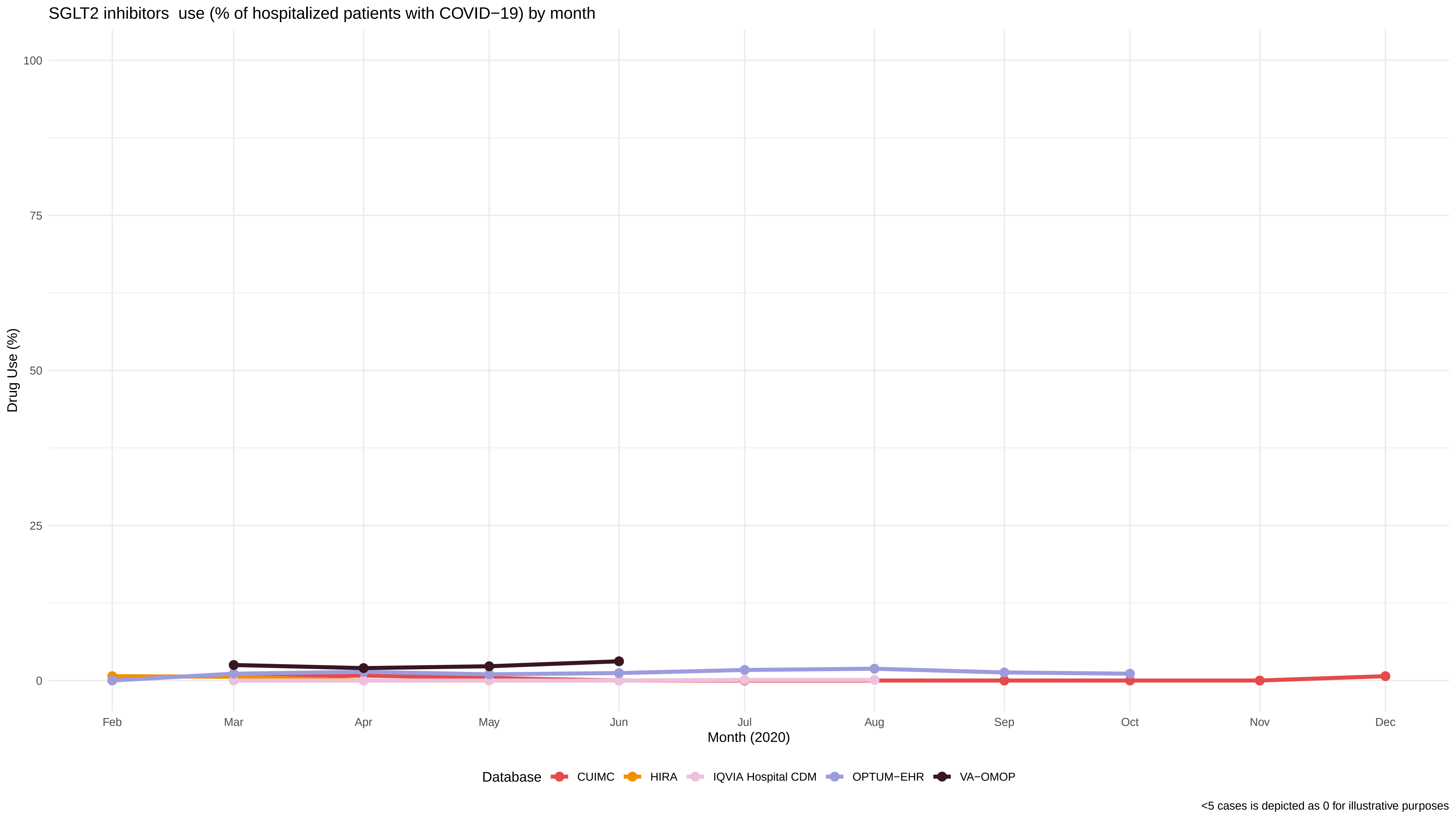
SGLT2 inhibitors use (% of hospitalized patients with COVID−19) by month
Drug Use (%)
100
75
50
25
0
Feb
Mar
Apr
May
Jun
Jul
Aug
Sep
Oct
Nov
Dec
Month (2020)
Database
CUIMC
HIRA
IQVIA Hospital CDM
OPTUM−EHR
VA−OMOP
<5 cases is depicted as 0 for illustrative purposes

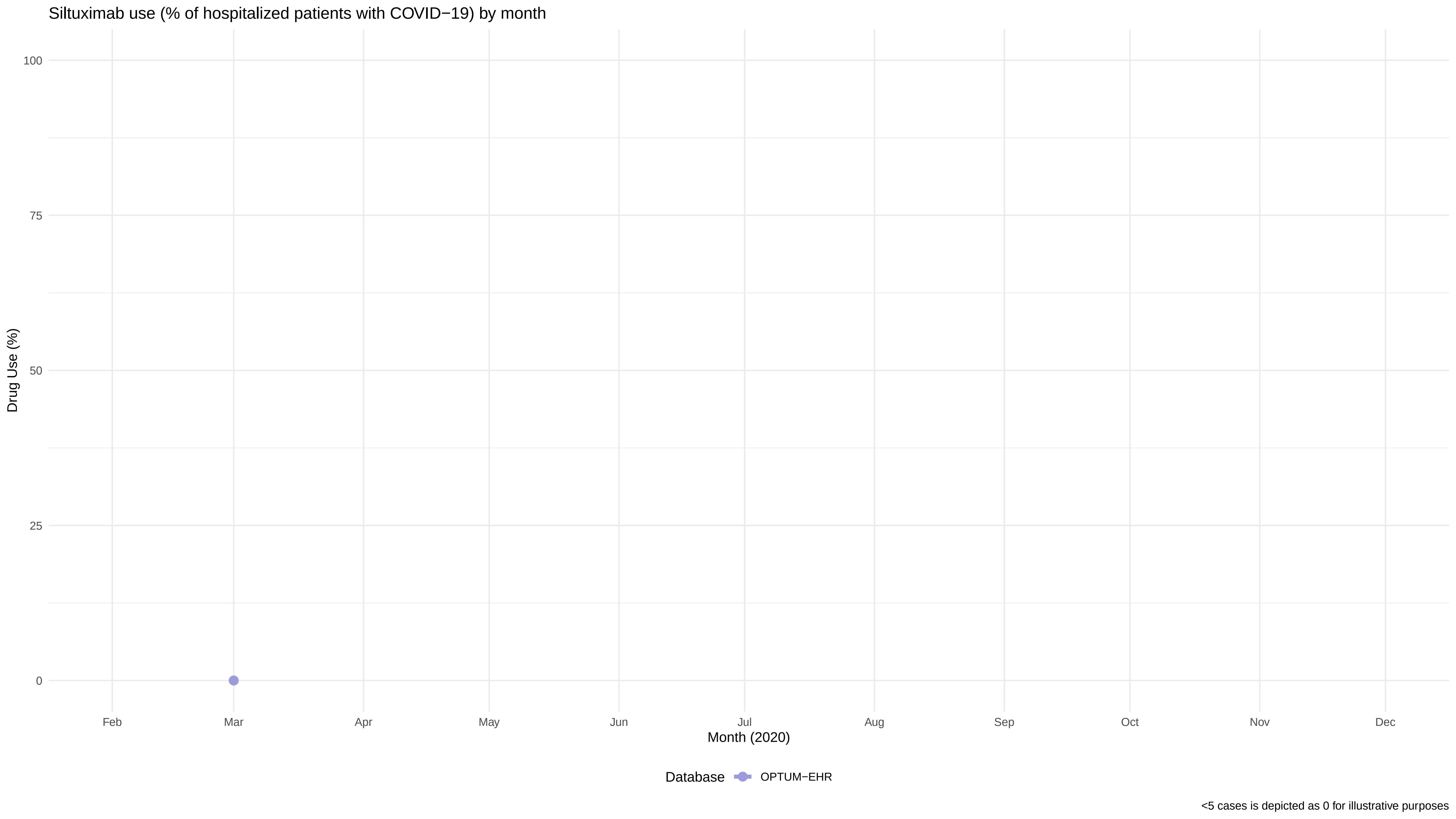
Siltuximab use (% of hospitalized patients with COVID−19) by month
100
75
50
25
0
Drug Use (%)
Feb
Mar
Apr
May
Jun
Jul
Aug
Sep
Oct
Nov
Dec
Month (2020)
Database
OPTUM−EHR
<5 cases is depicted as 0 for illustrative purposes

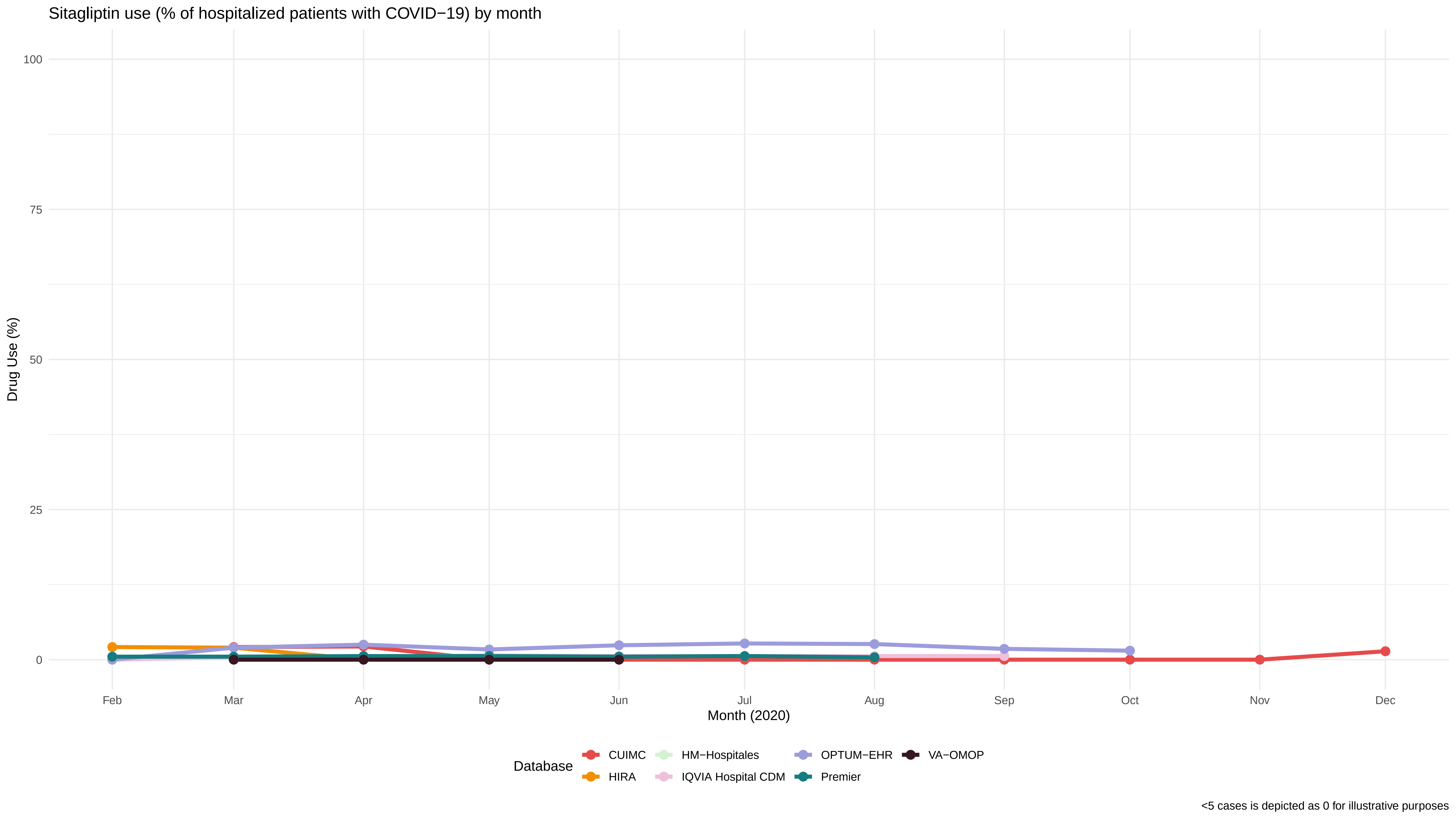

Sitagliptin use (% of hospitalized patients with COVID−19) by month
Drug Use (%)
100
75
50
25
0
Feb
Mar
Apr
May
Jun
Jul
Aug
Sep
Oct
Nov
Dec
Month (2020)
Database
CUIMC
HIRA
HM−Hospitales
IQVIA Hospital CDM
OPTUM−EHR
Premier
VA−OMOP
<5 cases is depicted as 0 for illustrative purposes

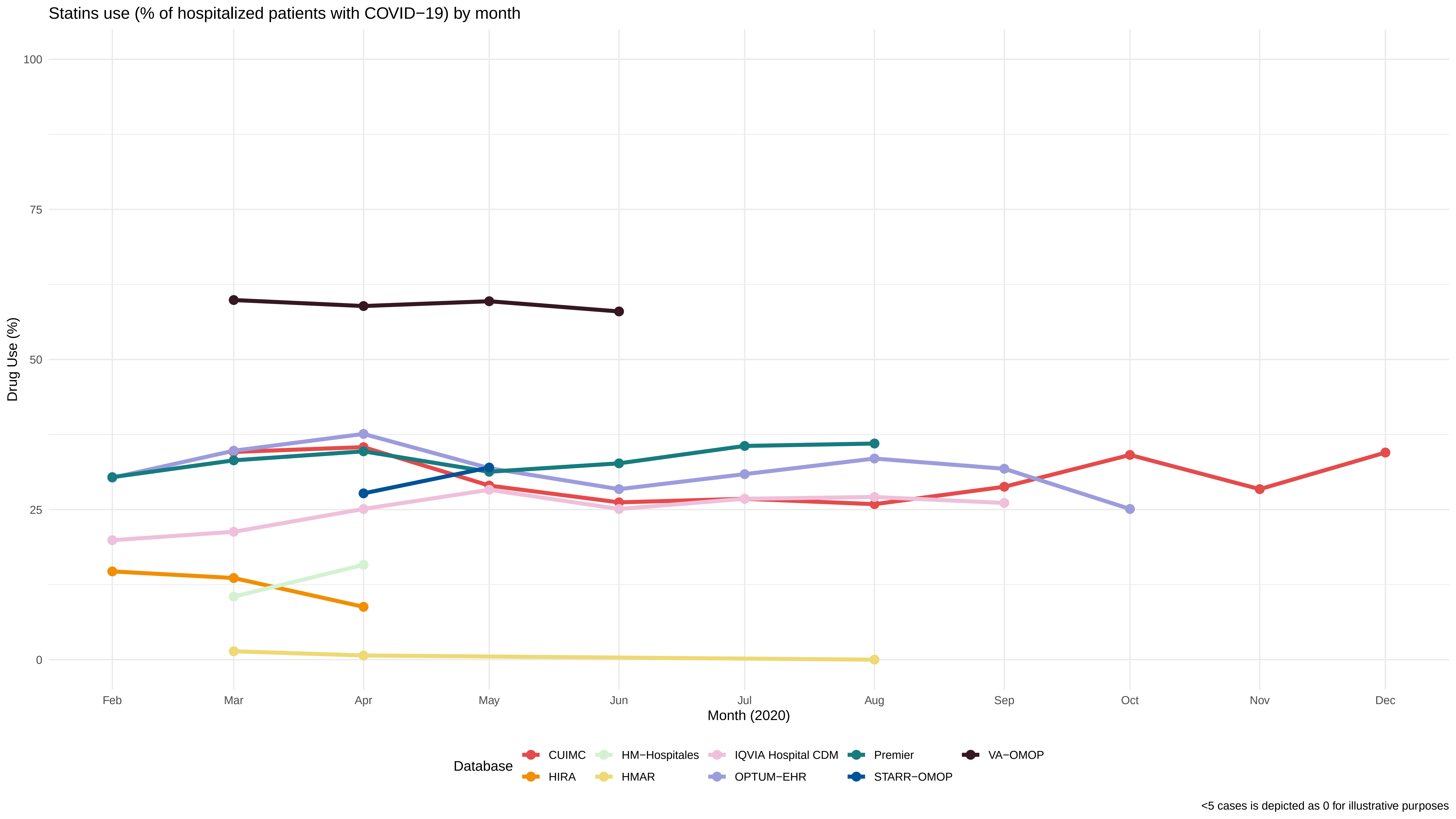

Statins use (% of hospitalized patients with COVID−19) by month
Drug Use (%)
100
75
50
25
0
Feb
Mar
Apr
May
Jun
Jul
Aug
Sep
Oct
Nov
Dec
Month (2020)
Database
CUIMC
HIRA
HM−Hospitales
HMAR
IQVIA Hospital CDM
OPTUM−EHR
Premier
STARR−OMOP
VA−OMOP
<5 cases is depicted as 0 for illustrative purposes

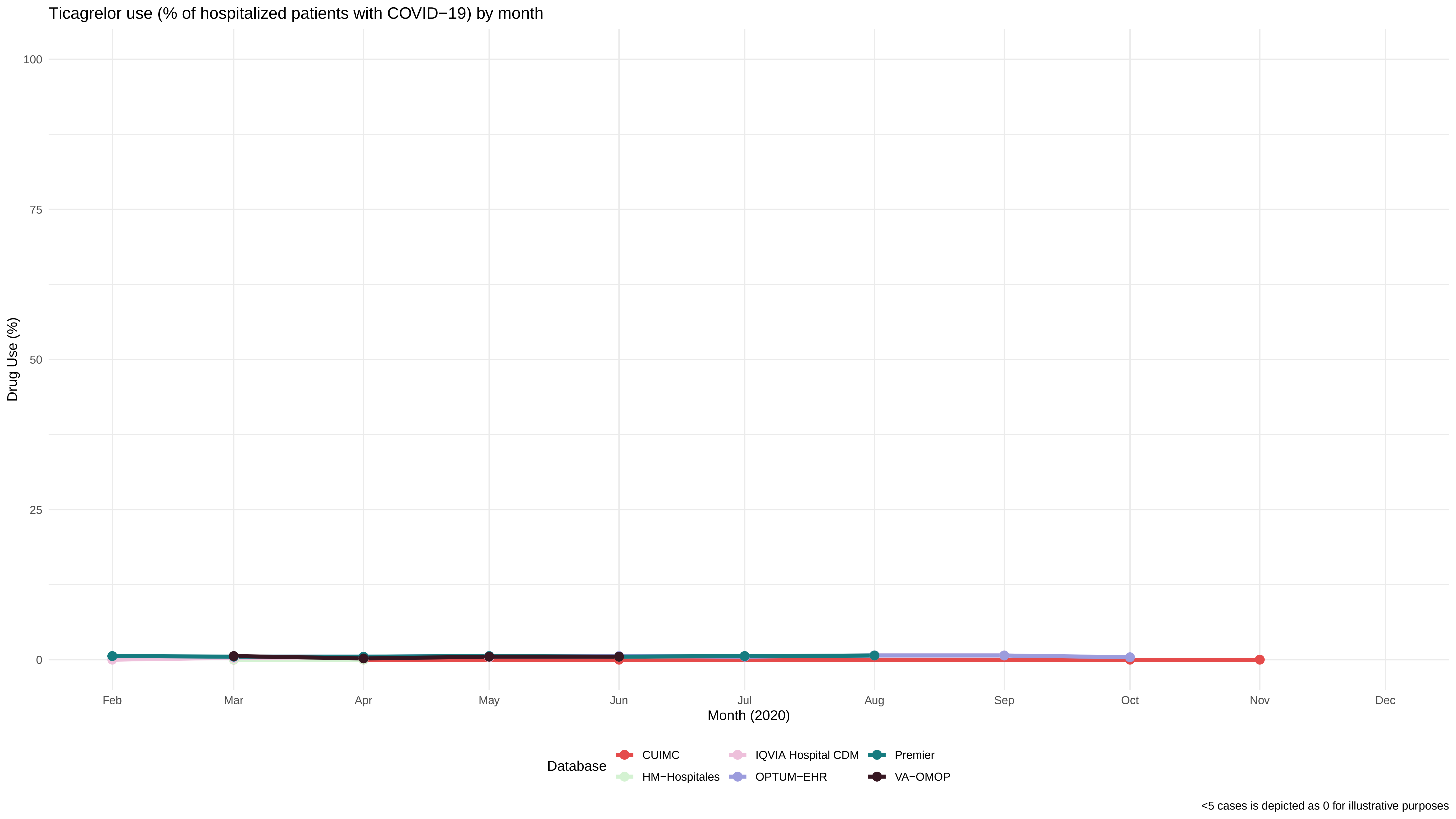
Ticagrelor use (% of hospitalized patients with COVID−19) by month
100
75
50
25
0
Drug Use (%)
Feb
Mar
Apr
May
Jun
Jul
Aug
Sep
Oct
Nov
Dec
Month (2020)
Database
CUIMC
HM−Hospitales
IQVIA Hospital CDM
OPTUM−EHR
Premier
VA−OMOP
<5 cases is depicted as 0 for illustrative purposes

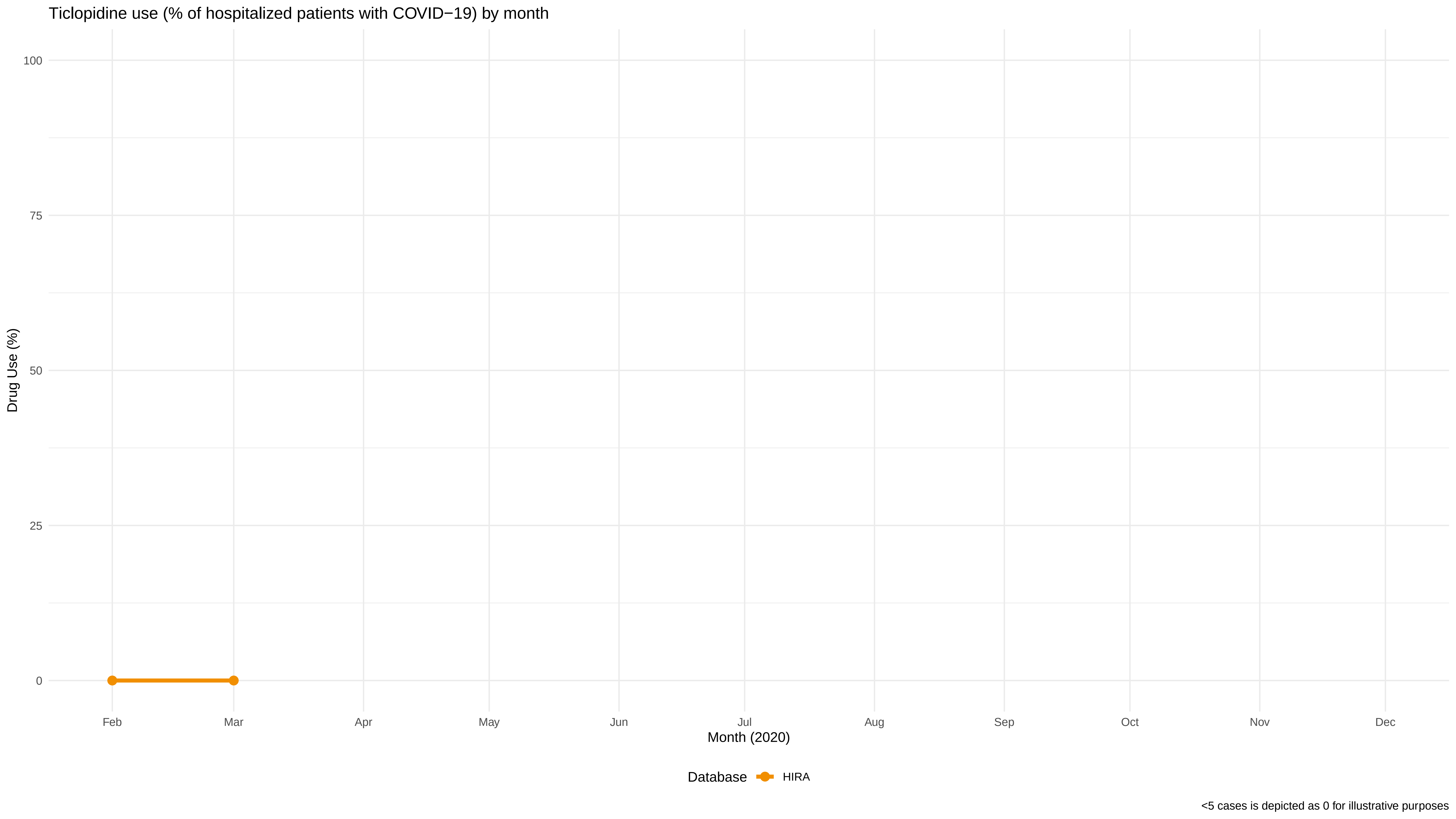
Ticlopidine use (% of hospitalized patients with COVID−19) by month
100
75
50
25
0
Drug Use (%)
Feb
Mar
Apr
May
Jun
Jul
Aug
Sep
Oct
Nov
Dec
Month (2020)
Database
HIRA
<5 cases is depicted as 0 for illustrative purposes

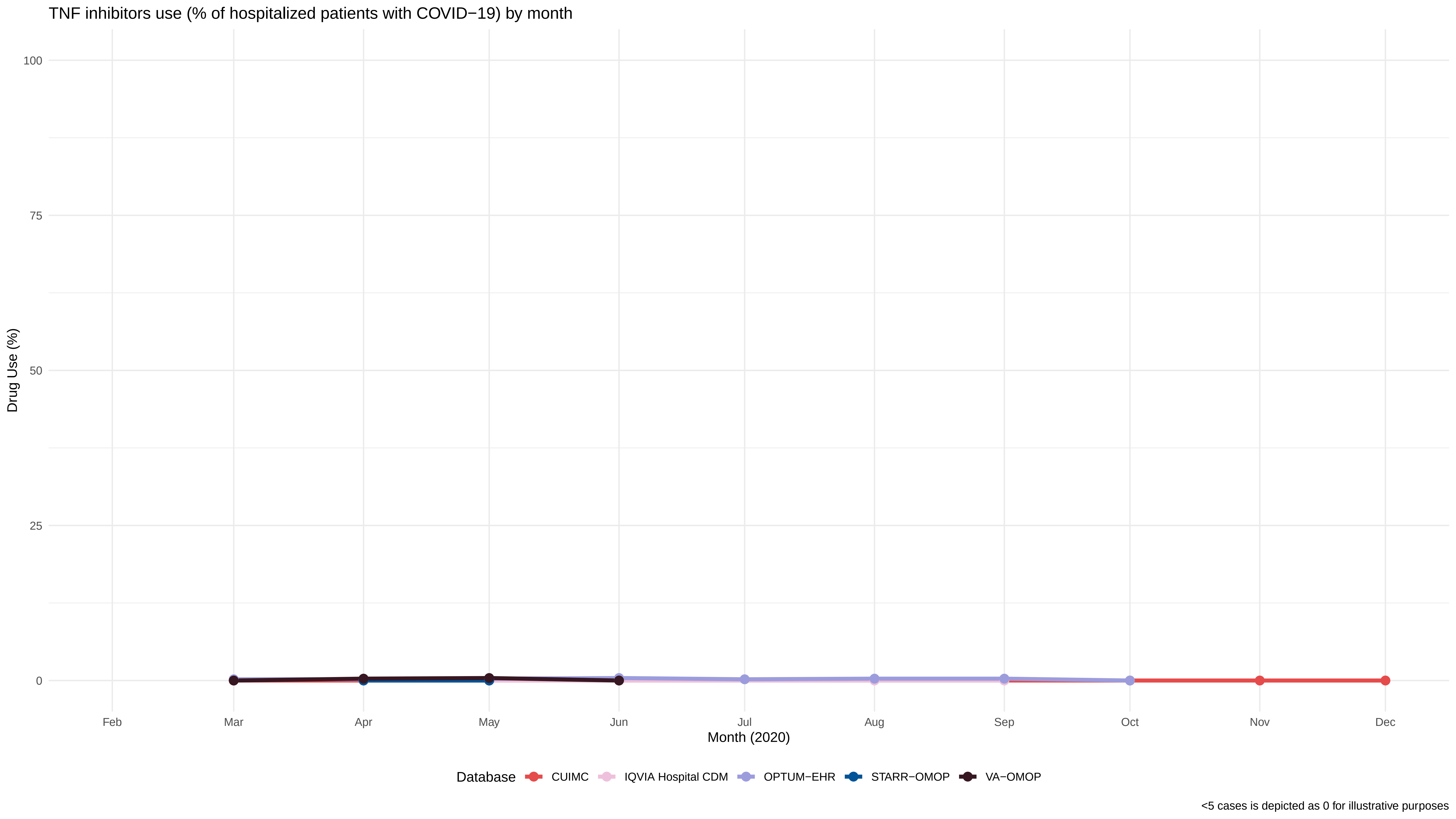
TNF inhibitors use (% of hospitalized patients with COVID−19) by month
Drug Use (%)
100
75
50
25
0
Feb
Mar
Apr
May
Jun
Jul
Aug
Sep
Oct
Nov
Dec
Month (2020)
Database
CUIMC
IQVIA Hospital CDM
OPTUM−EHR
STARR−OMOP
VA−OMOP
<5 cases is depicted as 0 for illustrative purposes

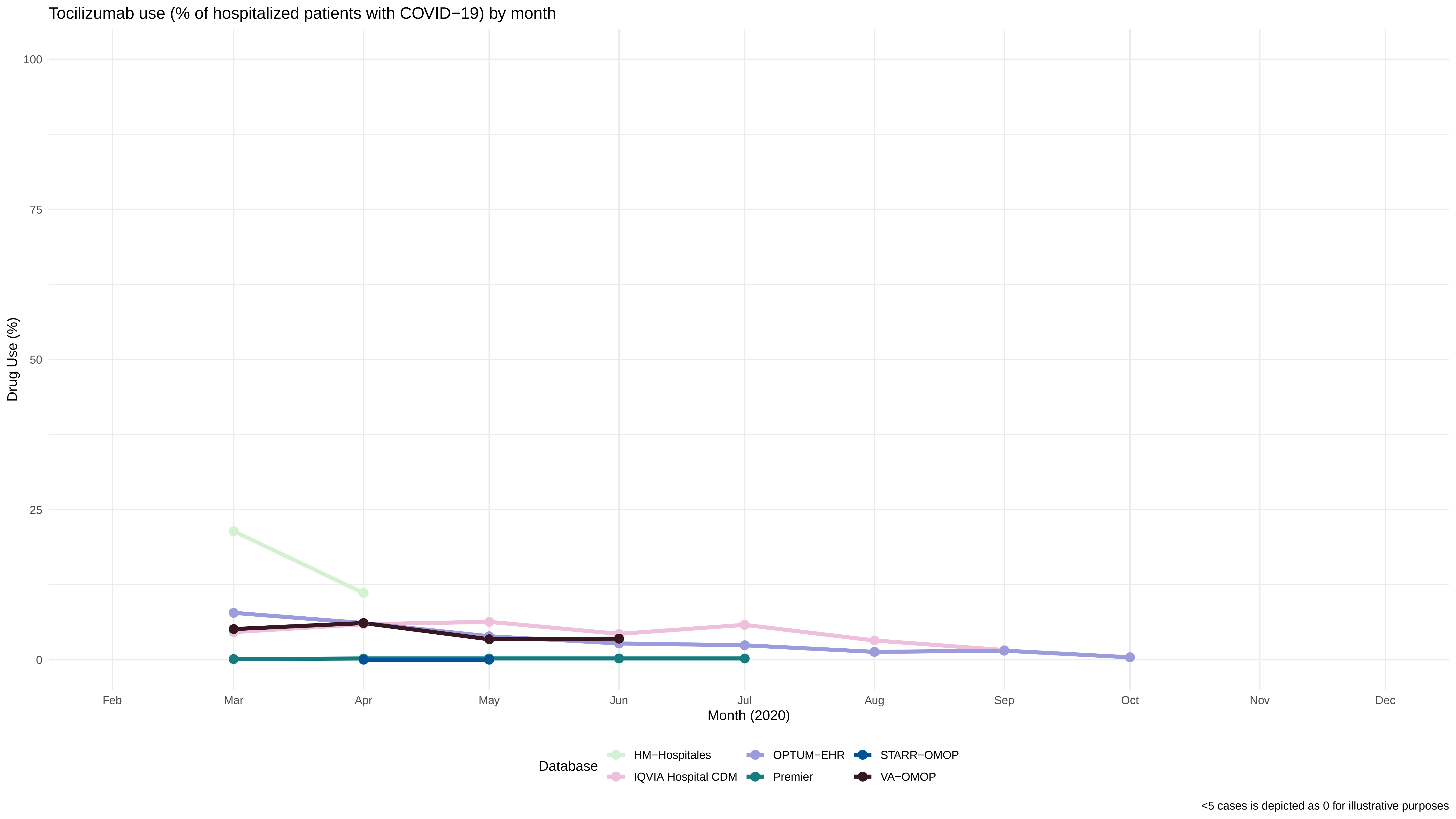
Tocilizumab use (% of hospitalized patients with COVID−19) by month
Drug Use (%)
100
75
50
25
0
Feb
Mar
Apr
May
Jun
Jul
Aug
Sep
Oct
Nov
Dec
Month (2020)
Database
HM−Hospitales
IQVIA Hospital CDM
OPTUM−EHR
Premier
STARR−OMOP
VA−OMOP
<5 cases is depicted as 0 for illustrative purposes

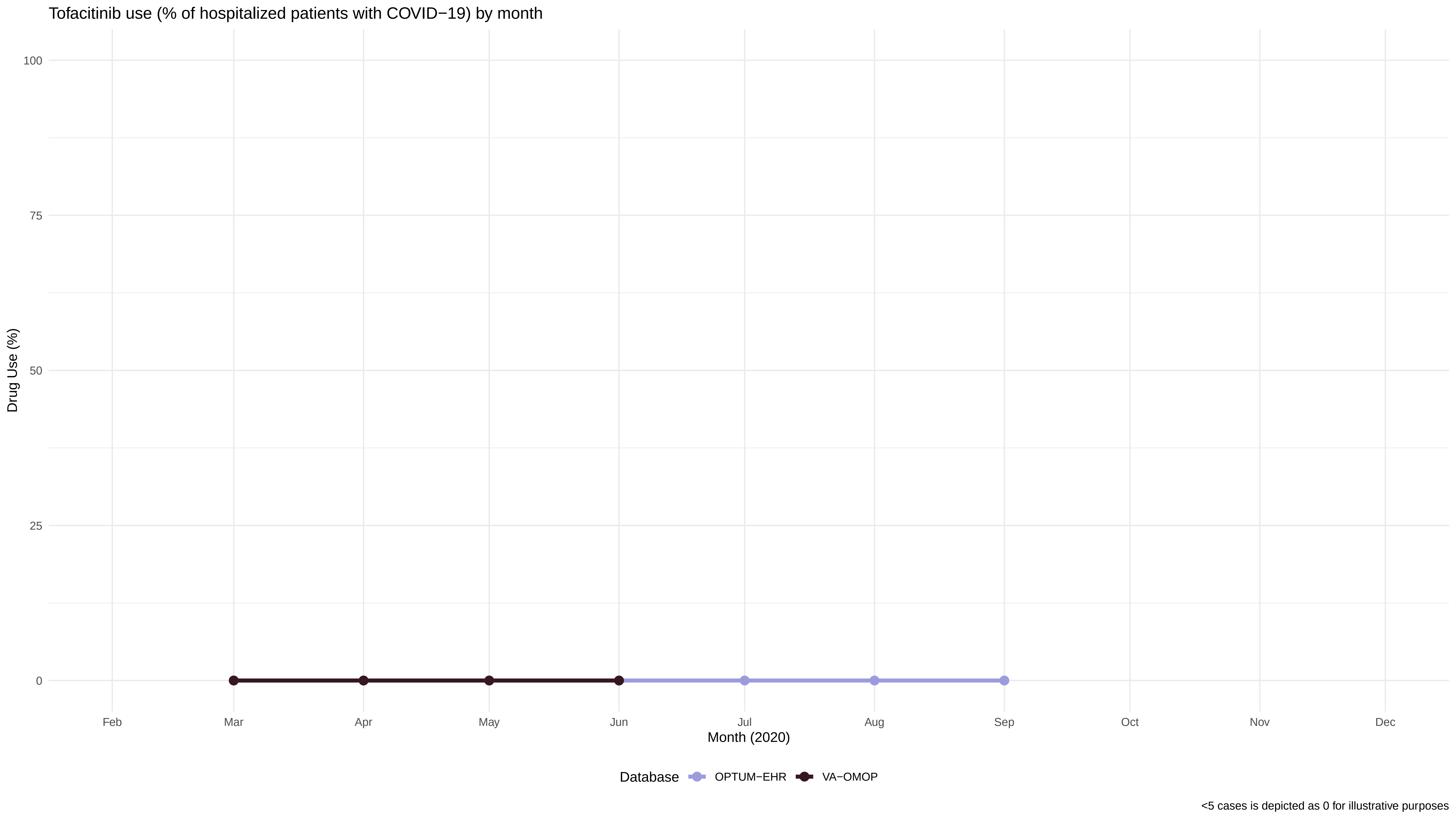
Tofacitinib use (% of hospitalized patients with COVID−19) by month
Drug Use (%)
100
75
50
25
0
Feb
Mar
Apr
May
Jun
Jul
Aug
Sep
Oct
Nov
Dec
Month (2020)
Database
OPTUM−EHR
VA−OMOP
<5 cases is depicted as 0 for illustrative purposes

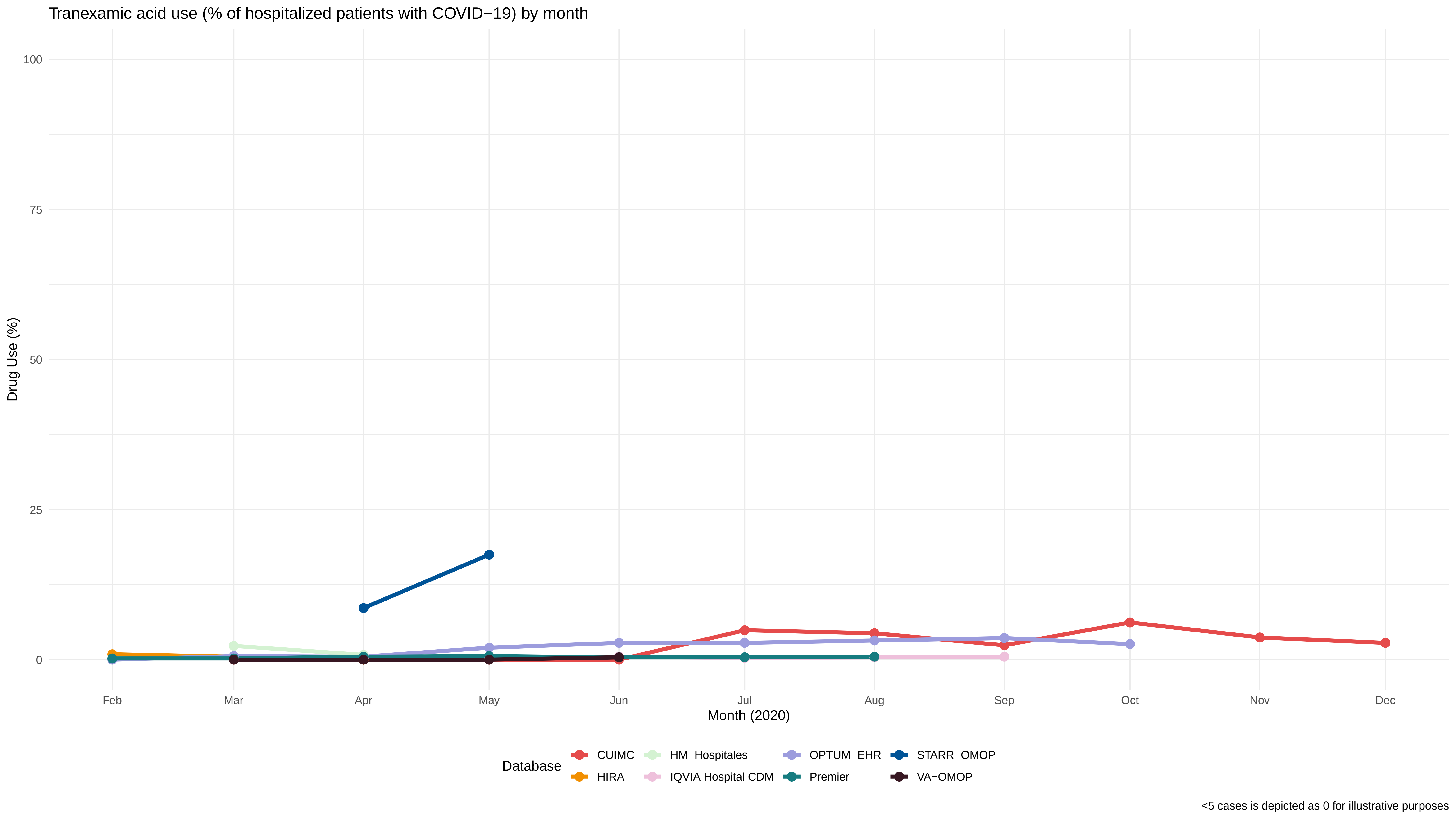
Tranexamic acid use (% of hospitalized patients with COVID−19) by month
Drug Use (%)
100
75
50
25
0
Feb
Mar
Apr
May
Jun
Jul
Aug
Sep
Oct
Nov
Dec
Month (2020)
Database
CUIMC
HIRA
HM−Hospitales
IQVIA Hospital CDM
OPTUM−EHR
Premier
STARR−OMOP
VA−OMOP
<5 cases is depicted as 0 for illustrative purposes

Triflusal use (% of hospitalized patients with COVID−19) by month

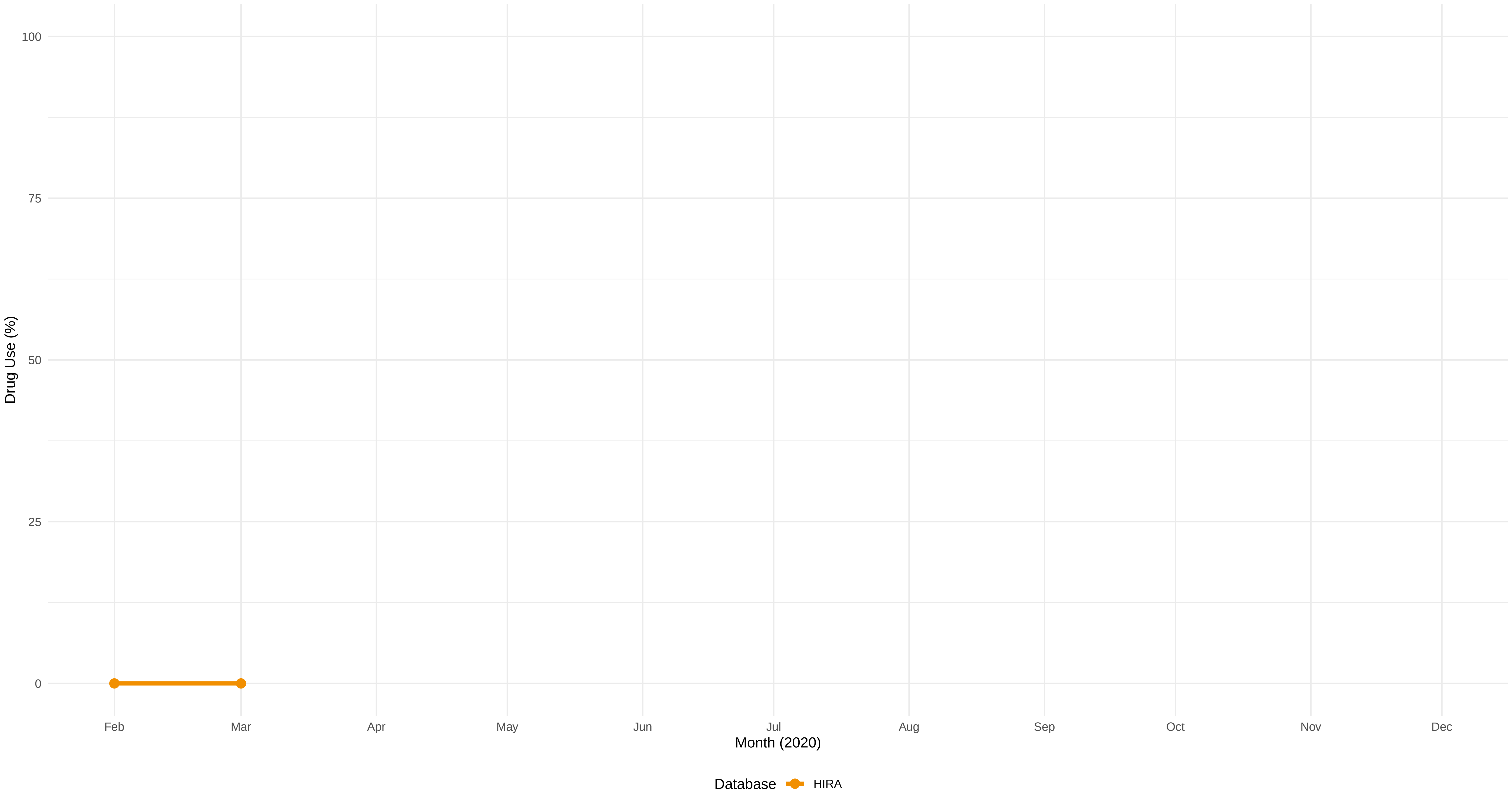

<5 cases is depicted as 0 for illustrative purposes

Ustekinumab use (% of hospitalized patients with COVID−19) by month

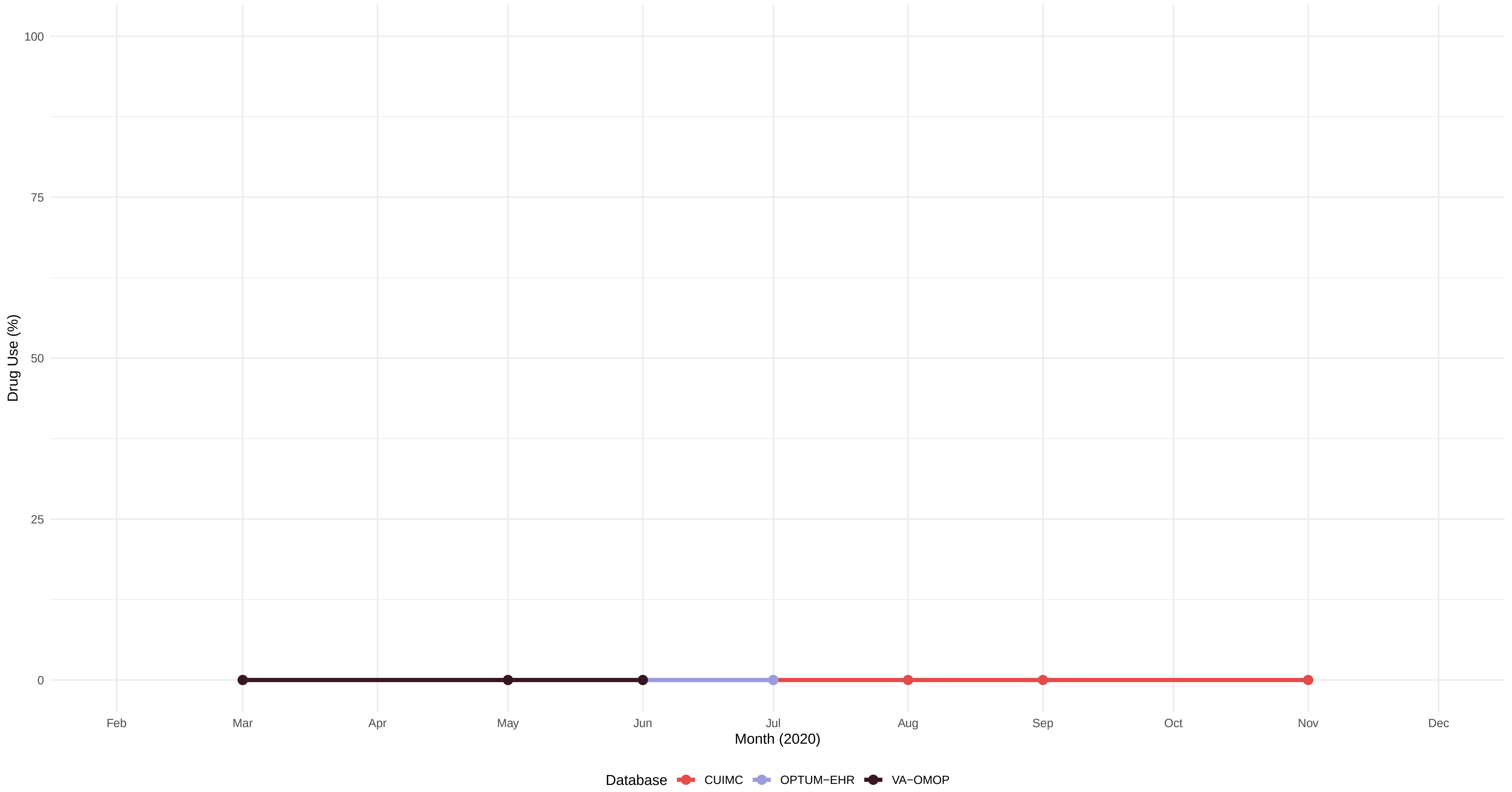

<5 cases is depicted as 0 for illustrative purposes

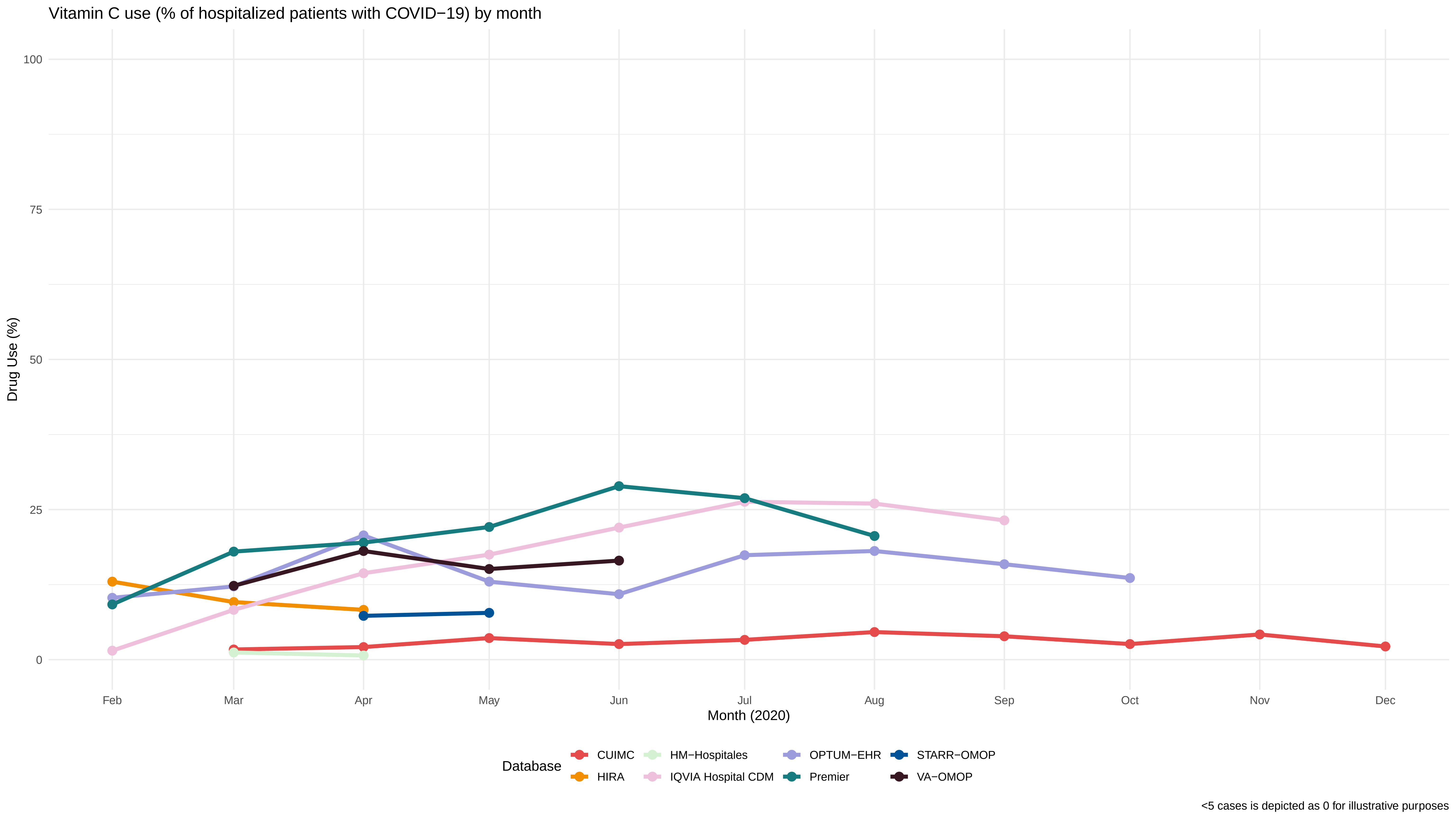

Vitamin C use (% of hospitalized patients with COVID−19) by month
Drug Use (%)
100
75
50
25
0
Feb
Mar
Apr
May
Jun
Jul
Aug
Sep
Oct
Nov
Dec
Month (2020)
Database
CUIMC
HIRA
HM−Hospitales
IQVIA Hospital CDM
OPTUM−EHR
Premier
STARR−OMOP
VA−OMOP
<5 cases is depicted as 0 for illustrative purposes

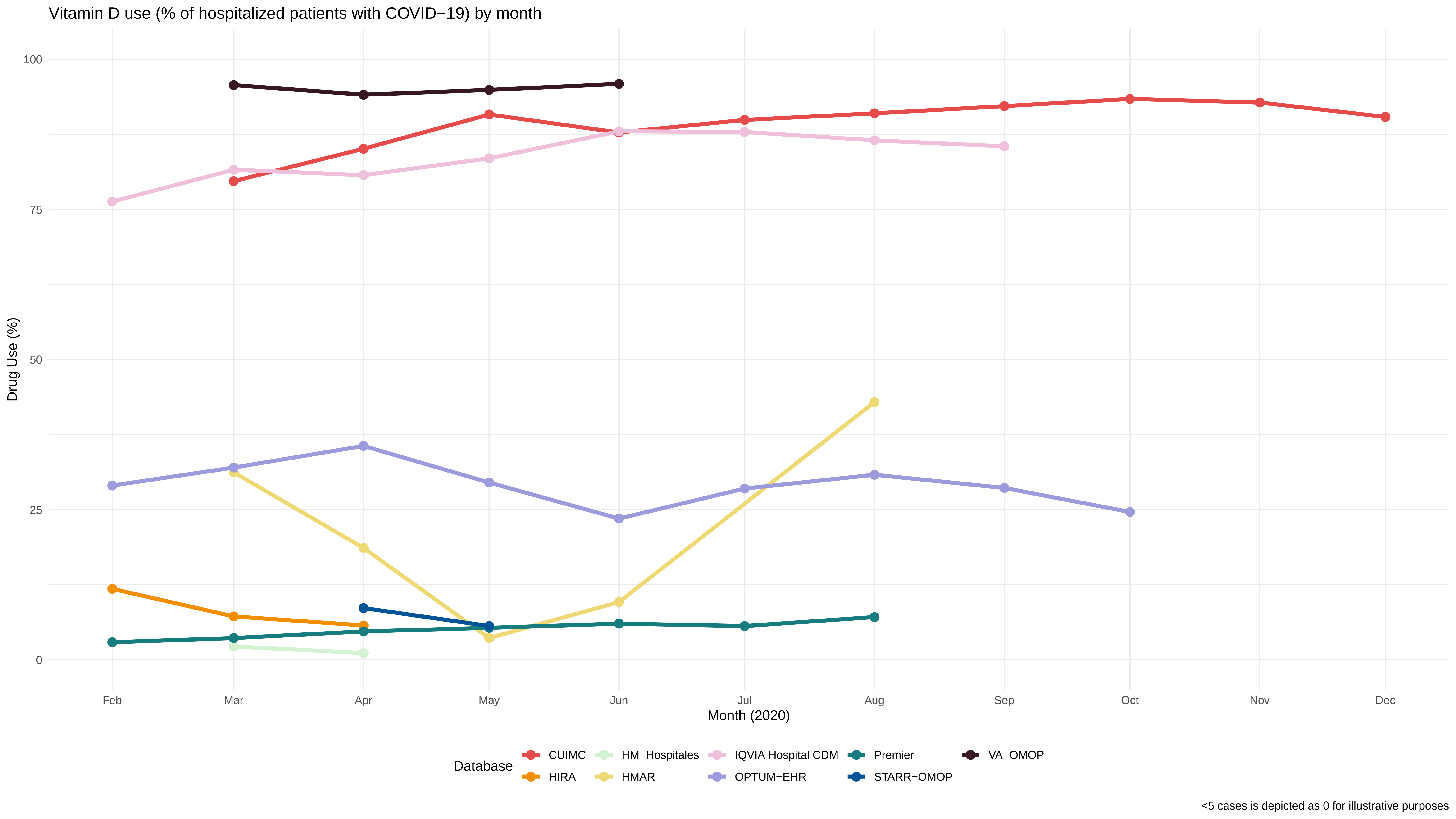
Vitamin D use (% of hospitalized patients with COVID−19) by month
Drug Use (%)
100
75
50
25
0
Feb
Mar
Apr
May
Jun
Jul
Aug
Sep
Oct
Nov
Dec
Month (2020)
Database
CUIMC
HIRA
HM−Hospitales
HMAR
IQVIA Hospital CDM
OPTUM−EHR
Premier
STARR−OMOP
VA−OMOP
<5 cases is depicted as 0 for illustrative purposes

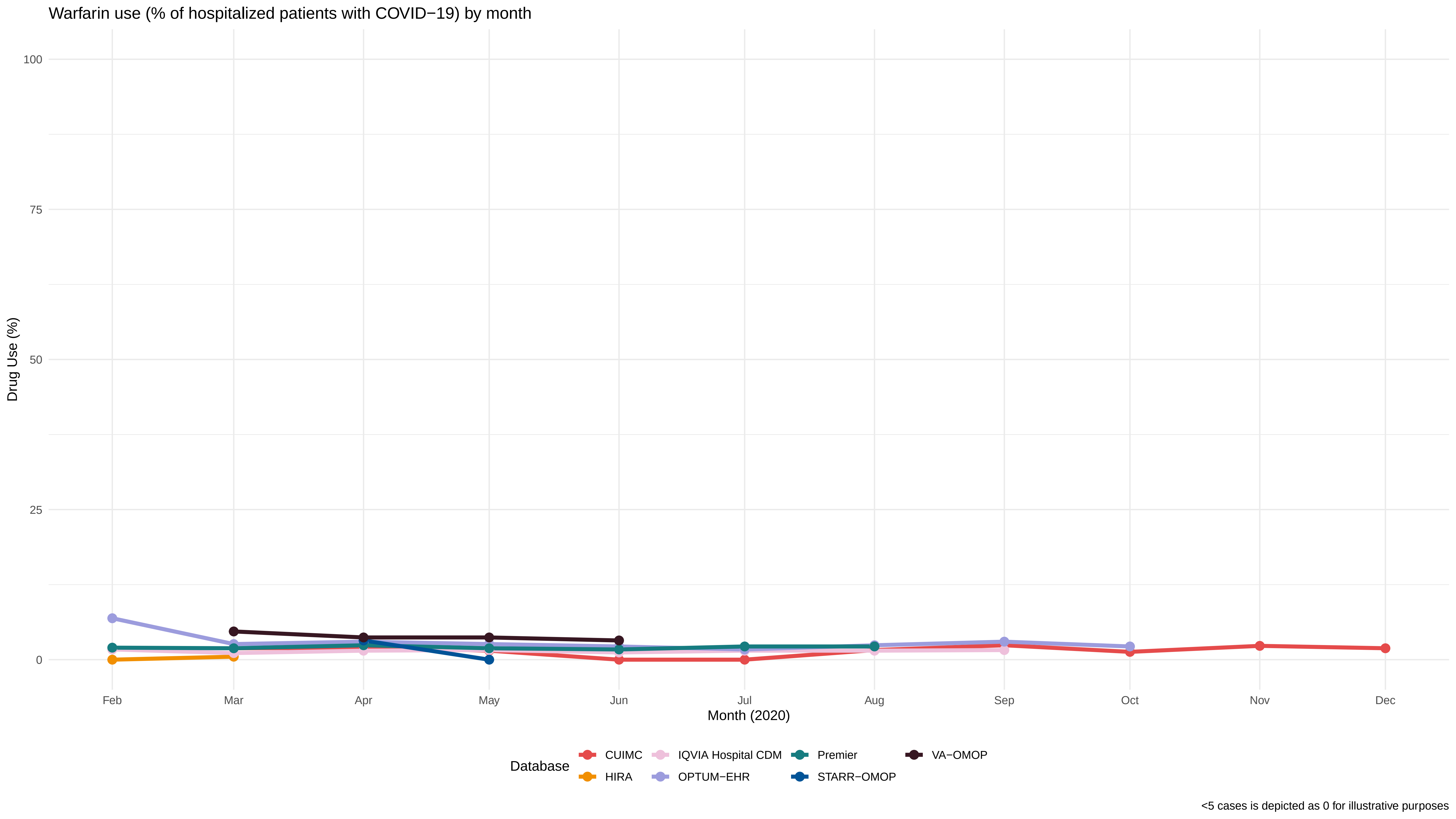

Warfarin use (% of hospitalized patients with COVID−19) by month
Drug Use (%)
100
75
50
25
0
Feb
Mar
Apr
May
Jun
Jul
Aug
Sep
Oct
Nov
Dec
Month (2020)
Database
CUIMC
HIRA
IQVIA Hospital CDM
OPTUM−EHR
Premier
STARR−OMOP
VA−OMOP
<5 cases is depicted as 0 for illustrative purposes
